# Effects of different doses of exercise and diet-induced weight loss on beta-cell function in type 2 diabetes (DOSE-EX): a randomized clinical trial

**DOI:** 10.1038/s42255-023-00799-7

**Published:** 2023-05-01

**Authors:** Grit E. Legaard, Mark P. P. Lyngbæk, Thomas P. Almdal, Kristian Karstoft, Sebastian L. Bennetsen, Camilla S. Feineis, Nina S. Nielsen, Cody G. Durrer, Benedikte Liebetrau, Ulrikke Nystrup, Martin Østergaard, Katja Thomsen, Beckey Trinh, Thomas P. J. Solomon, Gerrit Van Hall, Jan Christian Brønd, Jens J. Holst, Bolette Hartmann, Robin Christensen, Bente K. Pedersen, Mathias Ried-Larsen

**Affiliations:** 1 Centre for Physical Activity Research, Rigshospitalet, Copenhagen, Denmark; 2 Department of Endocrinology PE, Rigshospitalet, University of Copenhagen, Copenhagen, Denmark; 3 Department of Immunology & Microbiology, University of Copenhagen, Copenhagen, Denmark; 4 Department of Clinical Pharmacology, Bispebjerg-Frederiksberg Hospital, University of Copenhagen, Copenhagen, Denmark; 5 Blazon Scientific, London, UK; 6 Biomedical Sciences, Faculty of Health & Medical Science, University of Copenhagen, Rigshospitalet, Copenhagen, Denmark; 7 Clinical Metabolomics Core Facility, Clinical Biochemistry, University of Copenhagen, Rigshospitalet, Copenhagen, Denmark; 8 Department of Sports Science and Clinical Biomechanics, University of Southern Denmark, Odense, Denmark; 9 Department of Biomedical Sciences and the Novo Nordisk Foundation Center for Basic Metabolic Research, University of Copenhagen, Copenhagen, Denmark; 10 Section for Biostatistics and Evidence-Based Research, the Parker Institute, Bispebjerg and Frederiksberg Hospital, Copenhagen, Denmark; 11 Research Unit of Rheumatology, Department of Clinical Research, University of Southern Denmark, Odense, Denmark

**Keywords:** Translational research, Type 2 diabetes, Metabolism

## Abstract

Diet-induced weight loss is associated with improved beta-cell function in people with type 2 diabetes (T2D) with remaining secretory capacity. It is unknown if adding exercise to diet-induced weight loss improves beta-cell function and if exercise volume is important for improving beta-cell function in this context. Here, we carried out a four-armed randomized trial with a total of 82 persons (35% females, mean age (s.d.) of 58.2 years (9.8)) with newly diagnosed T2D (<7 years). Participants were randomly allocated to standard care (*n* = 20), calorie restriction (25% energy reduction; *n* = 21), calorie restriction and exercise three times per week (*n* = 20), or calorie restriction and exercise six times per week (*n* = 21) for 16 weeks. The primary outcome was beta-cell function as indicated by the late-phase disposition index (insulin secretion multiplied by insulin sensitivity) at steady-state hyperglycemia during a hyperglycemic clamp. Secondary outcomes included glucose-stimulated insulin secretion and sensitivity as well as the disposition, insulin sensitivity, and secretion indices derived from a liquid mixed meal tolerance test. We show that the late-phase disposition index during the clamp increases more in all three intervention groups than in standard care (diet control group, 58%; 95% confidence interval (CI), 16 to 116; moderate exercise dose group, 105%; 95% CI, 49 to 182; high exercise dose group, 137%; 95% CI, 73 to 225) and follows a linear dose–response relationship (*P* > 0.001 for trend). We report three serious adverse events (two in the control group and one in the diet control group), as well as adverse events in two participants in the diet control group, and five participants each in the moderate and high exercise dose groups. Overall, adding an exercise intervention to diet-induced weight loss improves glucose-stimulated beta-cell function in people with newly diagnosed T2D in an exercise dose-dependent manner (NCT03769883).

## Abstract (web summary)

Results from a randomized clinical trial show that adding exercise to diet-induced weight loss can improve beta-cell function, measured as late-phase disposition index, in people with newly diagnosed type 2 diabetes.

## Main

As the progressive deterioration of normal beta-cell function is regarded as a determining factor for the onset and subsequent progression of T2D, re-establishing beta-cell function is considered pivotal to improving the pathogenesis of T2D^1^.

Although a substantial diet-induced weight loss is consistent with improved beta-cell function^2–4^, the effects of exercise on beta-cell function in T2D are not well understood^5–9^. Inconsistent findings may relate to differences in concomitant pharmacological therapy, the participants’ pretrial insulin secretory capacity, or differences in exercise modality, intensity and/or volume^10–14^. The inconsistencies could also be related to a failure to correct for prevailing insulin sensitivity when assessing beta-cell function. As the normal physiological response to decreased insulin sensitivity is an increase in insulin secretion, the assessment of beta-cell function should incorporate both measures (that is, insulin sensitivity and secretion)^15^. A widely accepted measure of beta-cell function is the disposition index (DI), that is, the product of insulin sensitivity and insulin secretion^15^. Whereas there is evidence to suggest that exercise-induced improvements in DI are explained via improvements in insulin sensitivity and glucose disposal, the exercise-induced effects on insulin secretion in the context of prevailing insulin sensitivity remain to be clarified^5,10,16^.

Intensive structured weight management programs aiming for weight loss are recommended alongside pharmacological therapy to treat hyperglycemia^17^. Diet-induced weight loss is consistent with improvements in beta-cell function^2,18^, and glucose-lowering medications may increase insulin sensitivity, insulin secretion and incretin responses^19–21^. Therefore, potential interactions between these therapies and exercise should be considered when assessing the role of exercise on DI in people with T2D in a clinical setting. As such, there is a need to investigate the potential effects of exercise on DI in the context of standardized dietary weight loss and pharmacological therapy.

Accordingly, the primary objective of this study was to investigate the change in DI during the final 30 min of clamp-induced hyperglycemia (late-phase DI) after a 16-week intervention with different volumes of exercise in addition to diet-induced weight loss and algorithm-guided pharmacological management in people with newly diagnosed T2D. We hypothesized that late-phase DI would increase with increasing volumes of exercise in combination with diet-induced weight loss. Furthermore, we expected that both moderate and high volumes of exercise in combination with a diet-induced weight loss intervention would be superior to the control in improving late-phase DI^22^. The secondary objective was to assess the effects of the intervention on insulin sensitivity and secretion. Moreover, we aimed to explore the effects on cardiometabolic risk factors, postprandial glucose metabolism, glucose kinetics, glucagon-like peptide 1 (GLP-1) sensitivity and maximal insulin secretory capacity.

## Results

### Trial population and adherence to the intervention

**Fig. 1:**
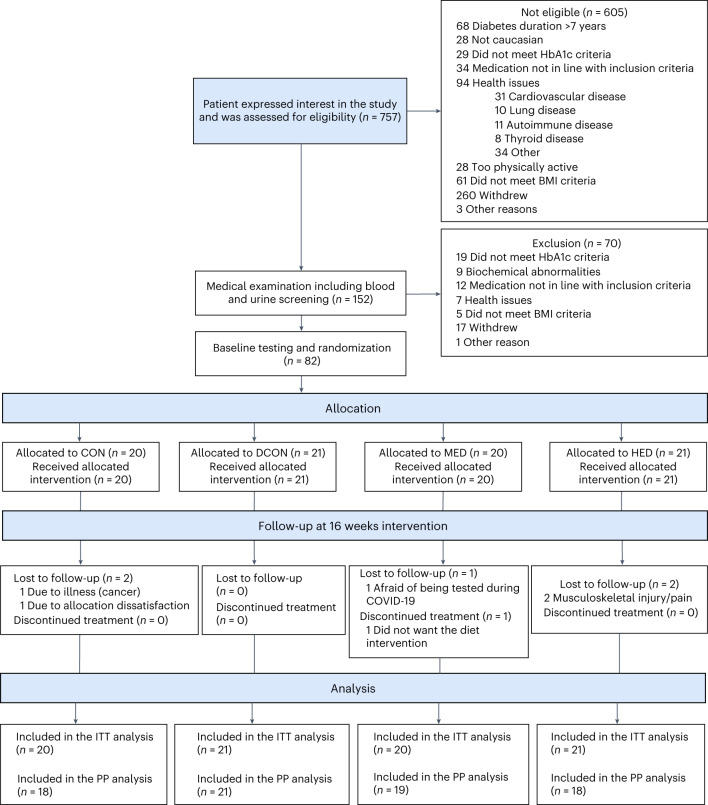
Flow of participants. CON, control group; DCON, diet control group; MED, moderate exercise dose group; HED, high exercise dose group; ITT, intention-to-treat; PP, per-protocol.

Eighty-two persons were included in the study (Fig. 1). Five participants were lost to follow-up: one was due to malignancy, one was dissatisfied with group allocation, one refrained from study testing due to COVID-19, and two were due to musculoskeletal injuries. The mean (s.d.) age was 58.2 years (9.8), body mass index (BMI) was 33 kg/m^2^ (3.7), and glycated hemoglobin (HbA1c) was 50.2 mmol/mol (6.6). Thirty-five percent (35%) of participants were females, and the median (interquartile range (IQR)) T2D duration was 4.0 years (1.9 to 5.5). Baseline characteristics are presented in Table 1. Mean (s.d.) adherence to the prescribed diet intervention (~25–30% energy deficit per day) was 92% (11) for the diet control group (DCON), 91% (18) for the moderate exercise dose group (MED), and 88% (13) for the high exercise dose group (HED) (Supplementary Table 1). Mean (s.d.) adherence to the prescribed exercise protocol was 86% (28) and 93% (18) for HED and MED, respectively (Supplementary Tables 2–7). No compensatory decrease in total free-living physical activity was seen in the intervention groups during the intervention period (Supplementary Table 8). In-study adherence to the predefined pharmacological treatment was similar among all groups (Supplementary Table 9).

**Table 1:** Baseline characteristics

	CON	DCON	MED	HED	Total
	Mean (s.d.)	Mean (s.d.)	Mean (s.d.)	Mean (s.d.)	Mean (s.d.)
	*n* = 20	*n* = 21	*n* = 20	*n* = 21	*n* = 82
**Clinical and cardiometabolic measurements**					
**General**					
Age, years	59.1 (9.2)	55.9 (10.0)	60.9 (7.6)	57.3 (11.8)	58.2 (9.8)
Sex, female, *n* (%)	7.0 (35.0)	7.0 (33.3)	7.0 (35.0)	8.0 (38.1)	29.0 (35.4)
T2D duration, median (IQR), years	3.5 (1.2; 5.4)	3.9 (2.9; 4.8)	4.1 (2.1; 5.9)	4.2 (3.4; 5.5)	4.0 (1.9; 5.5)
**Glycemic control**					
HbA1c, mmol/mol	52 (7)	49 (7)	51 (6)	49 (7)	50 (7)
HbA1c, %	6.9 (0.6)	6.7 (0.7)	6.8 (0.6)	6.7 (0.6)	6.7 (0.6)
Fasting glucose, median (IQR), mmol/l	9.1 (7.3; 10.3)	7.8 (7.3; 9.8)	7.7 (7.1; 9.6)	7.8 (7.0; 9.6)	7.8 (7.1; 10.0)
Fasting insulin, median (IQR), pmol/l	129 (95; 157)	149 (93; 198)	138 (88; 219)	127 (95; 166)	128 (91; 184)
Fasting C-peptide, pmol/l	1,206 (327)	1,247 (301)	1,387 (482)	1,248 (306)	1,271 (360)
**Lipids**					
LDL-C, mmol/l	3.2 (0.8)	2.9 (0.7)	2.9 (0.6)	2.6 (0.6)	2.9 (0.7)
Fasting triglycerides, mmol/l	1.5 (1.3)	1.5 (1.2)	1.8 (1.3)	1.4 (1.1)	1.5 (1.1)
**Blood pressure**					
Systolic, mmHg	128 (12)	127 (10.3*)	133 (11.2**)	129 (11)	129 (11)
Diastolic, mmHg	78 (6)	80 (6.7*)	81 (7.5**)	78 (7)	79 (7)
**Glucose-lowering medication,** ***n*** **(%)**					
None	5.0 (25.0)	8.0 (38.1)	5.0 (25.0)	5.0 (23.8)	23.0 (28.0)
Biguanide	9.0 (45.0)	7.0 (33.3)	11.0 (55.0)	9.0 (42.9)	36.0 (43.9)
Biguanide + SGLT2i or DPP4i	5.0 (25.0)	5.0 (23.8)	4.0 (20.0)	6.0 (28.6)	20.0 (24.4)
Biguanide + SGLT2i + DPP4i	1.0 (5.0)	1.0 (4.8)	0.0 (0.0)	1.0 (4.8)	3.0 (3.7)
**Lipid-lowering medication,** ***n*** **(%)**					
None	7.0 (35.0)	7.0 (33.3)	9.0 (45.0)	5.0 (23.8)	28.0 (34.1)
Statin	13.0 (65.0)	14.0 (66.7)	11.0 (55.0)	16.0 (76.2)	54.0 (65.9)
**Blood pressure-lowering medication,** ***n*** **(%)**					
None	11.0 (55.0)	9.0 (42.9)	11.0 (55.0)	6.0 (28.6)	37.0 (45.1)
ARB or ACEi	4.0 (20.0)	5.0 (23.8)	4.0 (20.0)	6.0 (28.6)	19.0 (23.2)
ARB or ACEi + thiazide or CCB	4.0 (20.0)	4.0 (19.0)	4.0 (20.0)	6.0 (28.6)	18.0 (22.0)
ARB or ACEi + thiazide + CCB	1.0 (5.0)	3.0 (14.3)	1.0 (5.0)	3.0 (14.3)	8.0 (9.8)
**Physical fitness**					
Absolute VO_2max_, ml/min	2,445.6 (413.2)	2,611.6 (618.2)	2,512.9 (634.5)	2,582.0 (714.4)	2,539.5 (599.2)
Relative VO_2max_, ml/kg/min	24.5 (3.7)	25.7 (3.6)	24.7 (4.6)	24.8 (4.3)	24.9 (4.0)
Watt max	192.8 (41.3)	204.8 (48.5)	189.7 (49.6)	204.8 (66.6)	198.2 (52.0)
1 RM chest press, median (IQR), kg	40.0 (35.0; 55.0**)	47.5 (35.0; 57.5)	45.0 (35.0; 57.5***)	52.5 (25.0; 65.0)	45.0 (35.0; 57.5)
1 RM leg extension, kg	68.0 (20.9*)	75.2 (24.4*)	63.6 (17.3)	68.0 (22.4)	68.7 (21.4)
**Body anthropometrics**					
Body weight, kg	100.3 (12.3)	100.8 (15.4)	101.6 (16.1)	102.8 (15.2)	101.4 (14.6)
BMI, kg/m^2^	32.4 (3.6)	33.2 (3.8)	33.2 (4.1)	33.4 (3.5)	33.1 (3.7)
**Diet**					
Energy intake, median (IQR), kcal/day	1,976.5 (1,772.0; 2,485.0)	1,990.0 (1,843.0; 2,247.0)	2,052.0 (1,800.0; 2,389.0**)	2,052.0 (1,571.0; 2,586.0)	1,995.0 (1,784.0; 2,465.0)
**Hyperglycemic clamp**					
**Basal**					
Mean ISR, median (IQR)	122 (72; 142)	110 (80; 175)	128 (82; 183)	100 (81; 167)	124 (78; 166)
Glucose *R*_a_, mg/kg/min	1.7 (0.2***)	1.7 (0.5**)	1.9 (0.5**)	1.8 (0.3*)	1.8 (0.4)
Glucose *R*_d_, mg/kg/min	1.6 (0.3***)	1.6 (0.5**)	1.8 (0.5**)	1.7 (0.3*)	1.7 (1.0)
EGP, mg/kg/min	1.7 (0.2***)	1.7 (0.5**)	1.8 (0.5**)	1.7 (0.3*)	1.7 (0.4)
**First-phase hyperglycemia (0–10** **min)**					
Mean GIR, mg/kg/min	10.7 (0.6)	10.6 (0.7)	10.6 (0.8)	10.6 (0.7)	10.6 (0.7)
Mean ISR, median (IQR), mmol/kg/min	106 (82; 143)	125 (77; 220)	123 (83; 190)	110 (83; 165)	116 (81; 173)
Peak ISR, median (IQR), mmol/kg/min	122 (92; 159)	132 (94; 229)	148 (103; 238)	136 (97; 173)	133 (94; 210)
**Late-phase hyperglycemia (90–120** **min)**					
Late-phase DI, a.u.	1.6 (0.7)	1.6 (0.8)	1.5 (0.9)	1.9 (1.4)	1.7 (1.0)
Late-phase ISI, mmol/kg/min	3.0 (1.9; 5.1)	3.1 (2.3; 5.2)	2.7 (2.0; 3.9)	3.2 (2.2; 5.4)	3.0 (2.0; 4.8)
Late-phase ISR, median (IQR), pmol/kg/min/mmol/l	239 (187; 459)	282 (185; 388)	317 (208; 532)	297 (211; 417)	282 (189; 446)
Mean GIR, mg/kg/min	2.5 (0.9)	2.4 (0.7)	2.6 (1.4)	2.8 (1.9)	2.6 (1.3)
Peak ISR, median (IQR)	253 (201; 525)	307 (198; 430)	355 (226; 563)	310 (234; 424)	309 (202; 503)
Glucose *R*_a_, mg/kg/min	4.1 (0.6***)	4.1 (0.7**)	4.4 (0.9**)	4.1 (1.1*)	4.2 (0.9)
Glucose *R*_d_, mg/kg/min	3.8 (0.7***)	4.3 (1.2**)	4.3 (0.9**)	4.7 (3.4*)	4.3 (1.9)
EGP, mg/kg/min	1.5 (0.8***)	1.6 (0.9**)	1.6 (1.2**)	1.4 (0.9*)	1.6 (0.9)
**Hyperglycemia and GLP-1 (120–180** **min)**					
Mean GIR, median (IQR), mg/kg/min	4.4 (3.3; 5.4)	4.0 (3.5; 6.2)	5.1 (3.4; 7.9)	4.6 (3.2; 8.8)	4.5 (3.3; 6.9)
Mean ISR, median (IQR)	506 (312; 1,009)	647 (423; 887)	783 (408; 1,343)	609 (411; 1,043)	629 (376; 1,048)
Peak ISR, median (IQR)	680 (420; 1,627)	914 (577; 1,402)	1274 (624; 2,177)	1049 (600; 1,778)	943 (534; 1,817)
**Hyperglycemia, GLP-1 and arginine (180–190** **min)**					
Mean ISR, median (IQR)	1,371 (1,027; 2,768.5***)	2,130 (1,504; 2,948.2***)	1,592 (1,228; 4,044.8****)	2,342 (1,151; 3,423.8***)	1,947 (1,163; 3,215)
Peak ISR, median (IQR)	1,937 (1,490; 3,579.0***)	3,227 (2,183; 3,775.0***)	2,256 (1,607; 5,048.0****)	3,011 (1,638; 4,746.0***)	2,818 (1,763; 4,050)
**Mixed meal tolerance test**					
**0–30** **min**					
tAUC glucose	5.2 (1.0)	5.1 (1.4)	4.8 (1.0)	4.9 (1.0)	5.0 (1.1)
tAUC C-peptide	838 (232)	880 (258)	868 (278)	888 (197)	869 (239)
tAUC insulin, median (IQR)	138 (110; 196)	171 (97; 210)	130 (84; 201)	177 (118; 209)	154 (92; 204)
tAUC GLP-1 total, median (IQR)	8 (7; 12)	8 (7; 11)	8 (7; 11)	9 (7; 11)	8 (7; 11)
tAUC GIP total	29 (10)	29 (9)	30 (12)	27 (10)	29 (10)
tAUC paracetamol, median (IQR)	0.0 (0.0; 0.0)	0.0 (0.0; 0.0)	0.0 (0.0; 0.0)	0.0 (0.0; 0.0)	0.0 (0.0; 0.0)
**0–120** **min**					
Oral DI (Matsuda × IGI), median (IQR)	391.9 (201.3)	273.5 (148.5)	338.5 (177.7)	389.3 (231.1)	340.0 (178.2)
Oral DI (Matsuda × AUC insulin/glucose), median (IQR)	149.8 (88.1)	193.8 (93.7)	168.6 (109.6)	182.3 (99.1)	168.6 (94.3)
AUC (insulin/glucose), median (IQR)	36.7 (27.4; 71.1)	43.1 (34.6; 63.3)	60.0 (23.1; 71.3)	42.9 (36.7; 85.6)	43.0 (28.2; 73.6)
Oral ISI, median (IQR)	3.3 (2.9; 5.2)	2.6 (2.2; 5.0)	3.8 (2.3; 5.4)	3.7 (2.8; 4.6)	3.6 (2.5; 5.0)
tAUC glucose	27.9 (5.3)	28.6 (7.0)	26.1 (6.1)	26.2 (6.3)	27.2 (6.2)
tAUC C-peptide	5,607 (1,611)	6,206 (2,292)	5,636 (1,544)	6,371 (2,309)	5,963 (1,974)
tAUC insulin, median (IQR)	1,081 (872; 1,584)	1,374 (921; 1,699)	1,221 (677; 1,576)	1,155 (935; 1,987)	1,167 (862; 1,646)
tAUC GLP-1 total, median (IQR)	40 (32; 52)	41 (37; 45)	39 (33; 46)	42 (33; 48)	41 (33; 46)
tAUC GIP total, median (IQR)	156 (122; 186)	157 (137; 186)	148 (114; 184)	146 (125; 189)	150 (123; 186)
tAUC paracetamol, median (IQR)	0.1 (0.1; 0.1)	0.1 (0.1; 0.1)	0.1 (0.1; 0.1)	0.1 (0.1; 0.1)	0.1 (0.1; 0.1)

Data are presented as means and standard deviations or medians with IQR (25^th^ to 75^th^ percentile).

Units for AUC glucose and paracetamol are presented in mmol/l × h, and units for AUC insulin, C-peptide, GLP-1 and GIP are presented in pmol/l × h. SGLT2i, sodium/glucose cotransporter 2 inhibitor; DPP4i, dipeptidyl peptidase 4 inhibitors; ARB, angiotensin ll receptor blocker; ACEi, angiotensin-converting enzyme inhibitor; CCB, calcium channel blocker; IGI, insulinogenic. *n = 20; **n = 19; ***n = 18; ****n = 16.

### Primary outcome

**Fig. 2:**
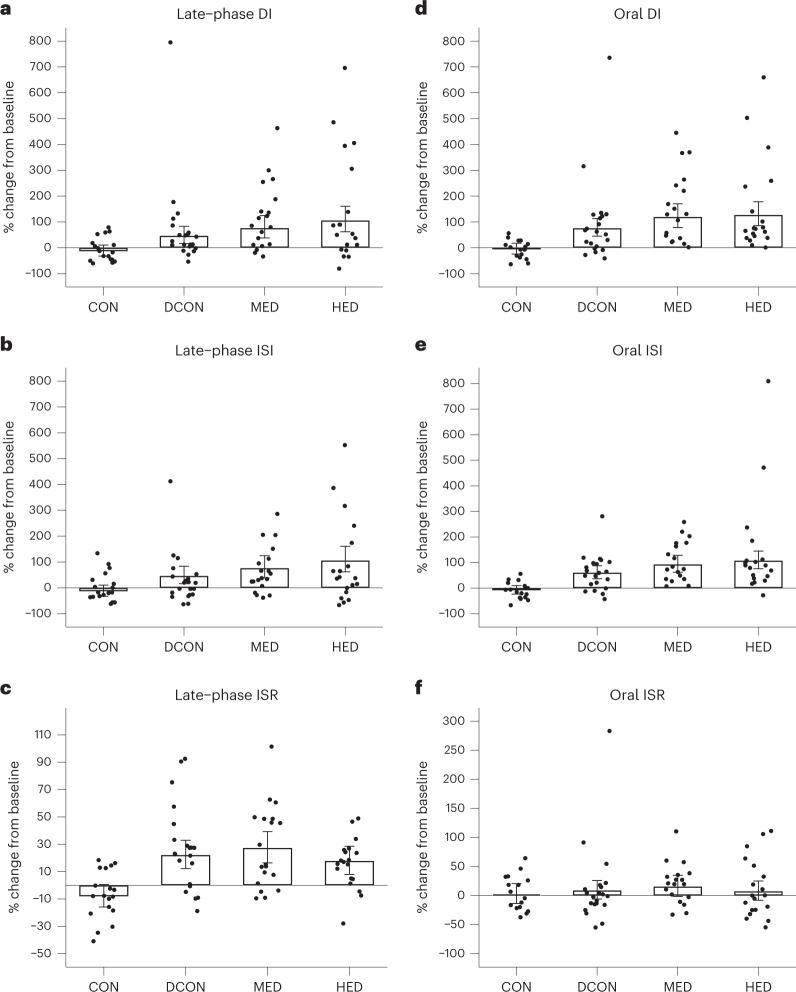
Changes from constrained baseline to 16-week follow-up in the primary and secondary outcomes. **a**–**f**, The bars represent estimated mean change from baseline for each intervention group from the *n* = 82 persons included in the study. Error bars represent 95% confidence intervals. Data were log(e)-transformed and back-transformed, and the results are presented as relative (percentage term) changes based on the ratio of geometric mean change from baseline to follow-up. Results were adjusted for sex. Data were analyzed using a constrained baseline longitudinal model. The dots represent the relative (percentage term) individual changes from baseline to follow-up. Left panel: Data are based on the final 30 min of the hyperglycemic clamp (stage 1). Right panel: Data are from 0 to 120 min of the MMTT. **a**, Change in late-phase DI by group. **b**, Change in late-phase ISI by group. **c**, Change in late-phase ISR by group. **d**, Change in oral DI of the MMTT by group. **e**, Change in oral ISI by group. **f**, Change in oral ISR by group. CON, *n* = 20; DCON, *n* = 21; MED, *n* = 20; HED, *n* = 21.

**Extended Data Fig. 1:**
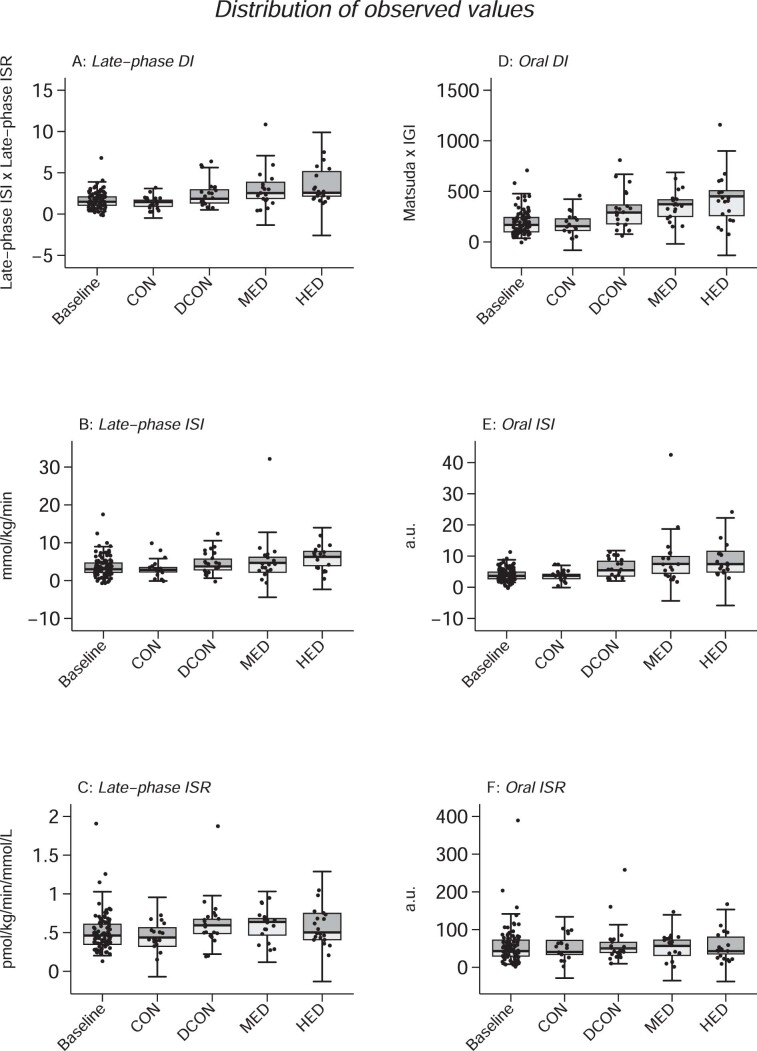
Box-plots for baseline values and by group at follow-up. As the primary analyses are performed using a constrained baseline model, where all groups are assumed to be similar a baseline, the baseline values are not depicted by group. Circles denotes the individual participant values. Center line is the median values, light grey area is the lower inter quartile range, dark grey area is the upper interquartile range, the whiskers show + /-1.5 x the interquartile range, CON: control group (N = 20 independent samples), DCON: Dietary control group (N = 21 independent samples), MED: Moderate volume exercise (N = 20 independent samples), HED: High volume exercise (N = 21 independent samples), DI: Disposition index, ISI: Insulin sensitivity index, ISR: Insulin secretion rate, pmol: pico mol, mmol: milli mol, a.u.: arbitrary units, kg: kilograms.

The late-phase DI increased in all intervention groups from baseline to 16-week follow-up with no change in the control group (CON) (Fig. 2a) in the intention-to-treat (ITT) analysis; as such, all intervention groups increased more than CON (*P* < 0.005 for all comparisons; Table 2). Compared with DCON, both MED and HED increased the late-phase DI (MED versus DCON, 29% (95% CI, −5 to 77), *P* = 0.11; HED versus DCON, 50% (95% CI, 10 to 104), *P* = 0.01) (Table 2). The magnitude of increases across groups was consistent with a linear dose–response relationship (*P* for trend <0.001). The per-protocol (PP) analysis set consisted of CON *n* = 18 (90%), DCON *n* = 21 (100%), MED *n* = 19 (95%) and HED *n* = 18 (86%), and followed the pattern observed in ITT (Table 2). The distribution of absolute values at baseline and follow-up are presented in Extended Data Fig. 1.

**Table 2:** Pairwise comparisons of the change in the primary outcome and secondary outcomes

	HED vs. CON		MED vs. CON		DCON vs. CON		HED vs. DCON		MED vs. DCON		HED vs. MED		Global *P*
	MD (95% CI)	*P*	MD (95% CI)	*P*	MD (95% CI)	*P*	MD (95% CI)	*P*	MD (95% CI)	*P*	MD (95% CI)	*P*	
**Primary outcome**													
Late-phase DI (ITT)	137 (73; 225)	˂0.001	105 (49; 182)	˂0.001	58 (16; 116)	0.004	50 (10; 104)	0.01	29 (−5; 77)	0.11	16 (−16; 59)	0.34	˂0.001
Late-phase DI (PP)	126 (62; 214)	<0.001	115 (54; 202)	<0.001	66 (21; 128)	0,002	36 (−1; 86)	0.058	30 (−7; 79)	0.12	5 (−25; 47)	0.79	˂0.001
**Secondary outcome**													
ISI	83 (35; 149)	˂0.001	50 (10; 104)	0.011	18 (−13; 60)	0.28	55 (15; 109)	0.004	27 (−7; 71)	0.13	23 (−10; 66)	0.20	˂0.001
ISR	28 (13; 45)	˂0.001	38 (22; 57)	˂0.001	33 (18; 50)	˂0.001	−4 (−15; 9)	0.56	4 (−8; 18)	0.51	−8 (−18; 5)	0.22	˂0.001
Oral DI	141 (80; 223)	˂0.001	133 (73; 213)	˂0.001	87 (40; 148)	˂0.001	29 (−2; 70)	0.065	25 (−5; 65)	0.12	4 (−22; 37)	0.81	˂0.001
Oral ISI	127 (78; 188)	˂0.001	110 (65; 168)	˂0.001	75 (38; 122)	˂0.001	29 (3; 62)	0.025	20 (−4; 51)	0.12	8 (−15; 36)	0.53	˂0.001
Oral ISR	5 (−16; 32)		13 (−10; 42)		7 (−14; 33)		−2 (−20; 22)		6 (−14; 31)		−7 (−25; 16)		0.76

Data were log(e)-transformed and back-transformed, and the results are presented as relative changes based on the ratio of estimated geometric mean (95% CI) change from baseline in one group versus the other. Results are adjusted for sex. Data were analyzed using a constrained baseline longitudinal model. *P* values are two-sided. No corrections for multiple comparisons were performed. If Global *P* is >0.1, *P* values are not reported for between-group comparisons. MD, mean difference.

### Secondary outcomes

The dose–response relationship observed for the late-phase DI was also reflected in the late-phase glucose-stimulated insulin sensitivity index (ISI) (*P* for trend <0.001), where both MED and HED increased more than CON, although the difference between DCON and CON was less pronounced (Fig. 2b). HED was associated with a greater increase in late-phase ISI compared with DCON (55% (95% CI, 15 to 109), *P* = 0.004). No differences were observed in late-phase ISI between DCON and CON. Late-phase glucose-stimulated insulin secretion rate (ISR) increased more in all intervention groups than in CON (Fig. 2c, Table 2 and Supplementary Tables 10 and 11), but no differences were observed among the remaining groups.

DI derived from the mixed meal tolerance test (MMTT) (oral DI) increased more in all intervention groups than in CON (Fig. 2d). The MED and HED groups increased more than DCON (MED versus DCON, 25% (95% CI, −5 to 65), *P* = 0.12; HED versus DCON, 29% (95% CI, −2 to 70), *P* = 0.065) with no signs of additional increases in HED versus MED (4% (95% CI, −22 to 37), *P* = 0.81).

All groups increased oral ISI compared with CON (P < 0.001) (Fig. 2e), with more pronounced increases in HED than DCON (29% (95% CI, 3 to 62), *P* = 0.025) (Table 2). No differences were observed in the oral ISR between the groups (Fig. 2f).

### Safety outcomes

Three serious adverse events were observed: one case of transient ischemic attack and one case of malignant melanoma in the CON group, and one case of prolactinoma in the DCON group (Table 3). Two participants in the DCON group, and five participants each in the MED and HED groups reported adverse events. Beyond musculoskeletal complaints and overuse injuries in MED and HED, the nature and frequency were similar between groups.

**Table 3:** Adverse events after randomization

	CON, *n* (%)	DCON, *n* (%)	MED, *n* (%)	HED, *n* (%)	All, *n* (%)
**Participants with** ≥**1 SAE or AE**^**a**^	2 (10.0)	3 (14.3)	5 (25.0)	5 (23.8)	15 (18.3)
**SAE**	2 (10.0)	1 (4.8)	0 (0.0)	0 (0.0)	3 (3.7)
**Infections (COVID-19)**	0 (0.0)	0 (0.0)	2 (10.0)	0 (0.0)	2 (2.4)
**Musculoskeletal pain and discomfort**					
Back pain	0 (0.0)	0 (0.0)	0 (0.0)	1 (4.8)	1 (1.2)
Lower extremities	0 (0.0)	0 (0.0)	0 (0.0)	0 (0.0)	0 (0.0)
Upper extremities	0 (0.0)	0 (0.0)	0 (0.0)	1 (4.8)	1 (1.2)
Other	0 (0.0)	0 (0.0)	0 (0.0)	2 (9.5)	2 (2.4)
**Musculoskeletal injury**					
Back pain	0 (0.0)	0 (0.0)	0 (0.0)	1 (4.8)	1 (1.2)
Lower extremities	0 (0.0)	0 (0.0)	1 (5.0)	1 (4.8)	2 (2.4)
Fatigue	0 (0.0)	0 (0.0)	0 (0.0)	1 (4.8)	1 (1.2)
Other	0 (0.0)	0 (0.0)	0 (0.0)	1 (4.8)	1 (1.2)
**Complications associated with clinical or experimental procedures**					
Allergic reactions to bandages and wound plasters	0 (0.0)	0 (0.0)	1 (5.0)	2 (9.5)	3 (3.7)
Felt uncomfortable during hyperglycemic clamp	0 (0.0)	1 (4.8)	1 (5.0)	0 (0.0)	2 (2.4)
Pain from muscle biopsy	0 (0.0)	1 (4.8)	1 (5.0)	0 (0.0)	2 (2.4)
Peripheral intravenous catheter went subcutaneous during hyperglycemic clamp	0 (0.0)	1 (4.8)	0 (0.0)	0 (0.0)	1 (1.2)
**Nutrition**	0 (0.0)	0 (0.0)	0 (0.0)	0 (0.0)	0 (0.0)

^a^(%)The numerator is the number of participants with at least one adverse event in each group, and the denominator is the total number of participants in the group.

SAE, serious adverse event; AE, adverse event. CON, *n* = 20; DCON, *n* = 21; MED, *n* = 20; HED, *n* = 21.

SAEs included one case of transient ischemic attack and one case of malignant melanoma in the CON group, and one case of prolactinoma in the DCON group.

Musculoskeletal pain and discomfort allows for modification of an exercise so that the prescribed exercise intervention can be performed.

Musculoskeletal injury is defined as pain or discomfort to an extent that precludes participating in at least one protocol-prescribed exercise for >7 days.

Nutrition is defined as events of increased or decreased hunger or satiety.

### Exploratory outcomes

#### Supporting clamp-derived indices of beta-cell function

**Fig. 3:**
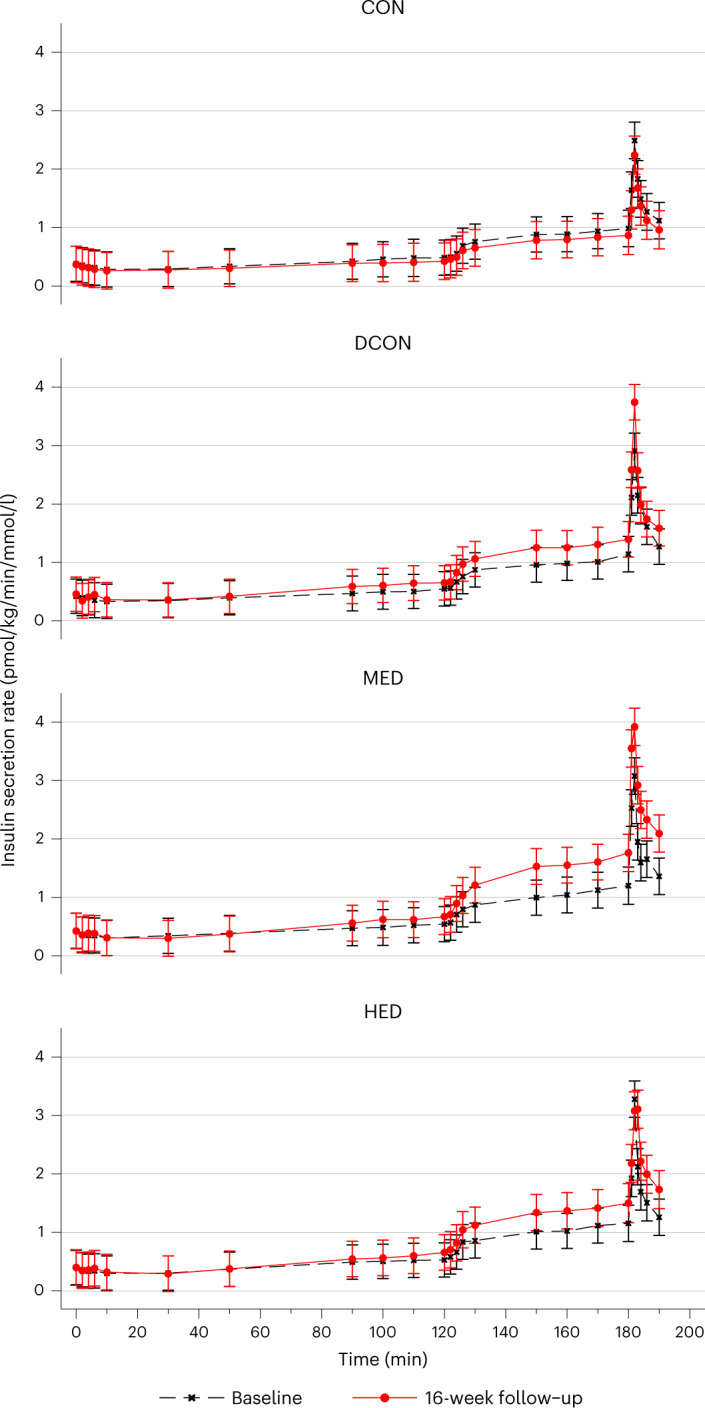
Insulin secretion rate across a three-phase hyperglycemic clamp. Data are represented as the marginal means with 95% confidence intervals. Results are adjusted for sex. Data were analyzed using a longitudinal mixed model. The black dashed line represents baseline values, and the red solid line represents the 16-week follow-up values.

The first-phase (0–10 min of clamp-induced hyperglycemia) DI increased in all intervention groups for all comparisons with CON (*P* ˂ 0.001; Supplementary Table 11). In addition, both HED and MED increased the first-phase DI more than DCON (HED versus DCON, 37% (95% CI, 6 to 77), *P* = 0.001; MED versus DCON, 58% (95% CI, 22 to 105), *P* = 0.017). No difference was observed between HED and MED (Supplementary Table 11). Peak and mean ISR in response to GLP-1 and GLP-1 + arginine infusion increased more from baseline to follow-up in all intervention groups compared with CON (Fig. 3 and Supplementary Tables 10 and 11). Whereas HED did not increase ISR in response to GLP-1 compared with DCON, MED was associated with increased ISR in response to GLP-1 compared with DCON (peak ISR, 0.2 (pmol/kg/min) × mM^−1^ (95% CI, 0.0 to 0.3), *P* = 0.019; mean ISR, 0.3 (pmol/kg/min) × mM^−1^ (95% CI, 0.05 to 0.6), *P* = 0.045). All intervention groups increased ISR in response to arginine, but no consistent differences were observed among the intervention groups (Supplementary Table 11).

#### Glucose kinetics

**Extended Data Fig. 2:**
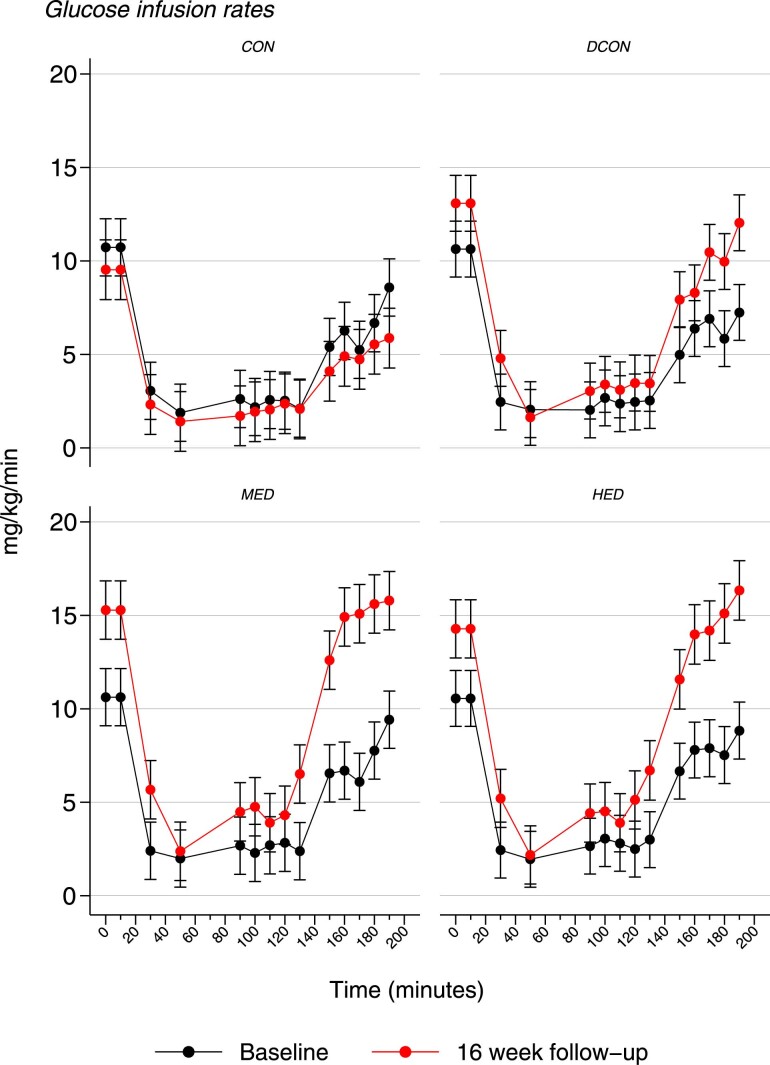
The black circles represent baseline values, and the red circles represents the 16-week follow-up values. Data are represented as the estimated means. Error bars are 95% confidence intervals. Results are adjusted for sex. Time 0-120 minutes is the hyperglycemic phase. Time 120-180 minutes is the hyperglycemic - and GLP-1 stimulation phase. Time 180-190 minutes is the hyperglycemic, GLP-1, and Arginine HCl stimulation phase. CON: control group (N = 20 independent samples), DCON: Dietary control group (N = 21 independent samples), MED: Moderate volume exercise (N = 20 independent samples), HED: High volume exercise (N = 21 independent samples).

The change in basal rate of glucose appearance (*R*_a_) and disappearance (*R*_d_), and thus the basal endogenous glucose production (EGP), was increased only in HED compared with CON, but no additional differences between the groups were observed. Late-phase *R*_d_ and *R*_a_ increased more in all intervention groups than in CON (*P* < 0.001 for all comparisons); HED increased more than DCON (difference in *R*_d_, 0.8 (95% CI, 0.2 to 1.4), *P* = 0.012; difference in *R*_a_, 0.7 (95% CI, 0.1 to 1.3), *P* = 0.022). Complete data on glucose infusion rate (GIR), EGP, *R*_d_ and *R*_a_ are presented in Extended Data Fig. 2 and Supplementary Tables 10 and 11.

#### Postprandial glucose metabolism

**Extended Data Fig. 3:**
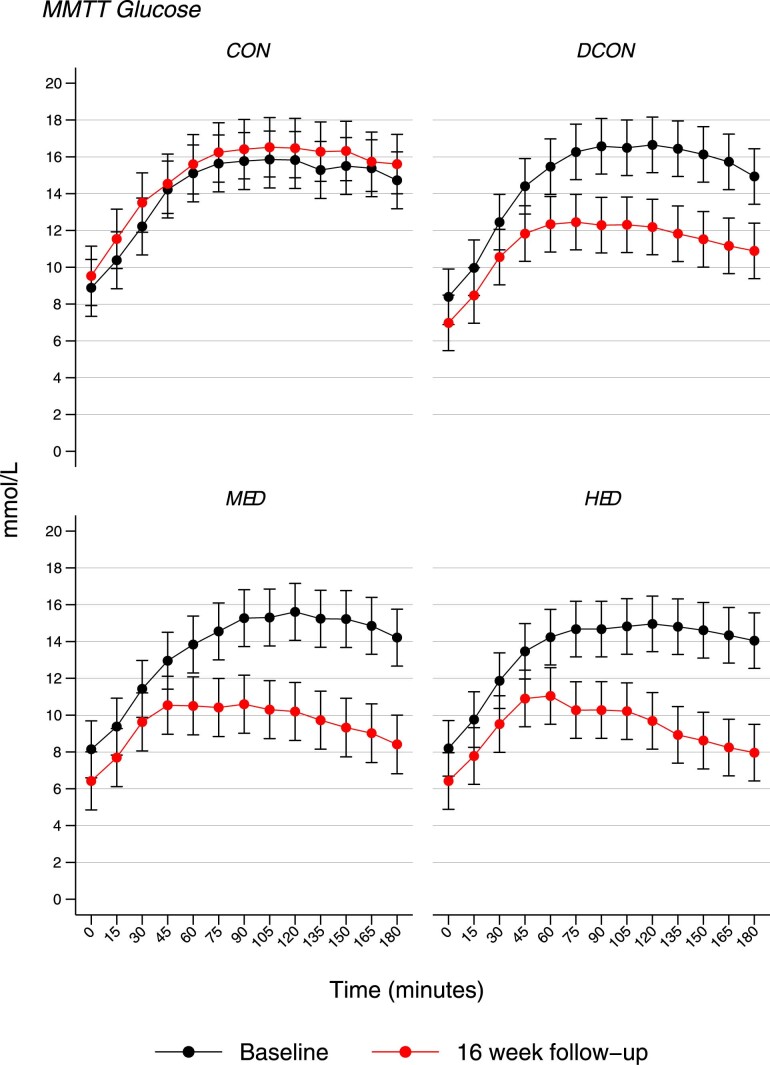
The black circles represent baseline values, and the red circles represents the 16-week follow-up values. Data are represented as the estimated means. Error bars are 95% confidence intervals. Results are adjusted for sex. CON: control group (N = 20 independent samples), DCON: Dietary control group (N = 21 independent samples), MED: Moderate volume exercise (N = 20 independent samples), HED: High volume exercise (N = 21 independent samples).

**Extended Data Fig. 4:**
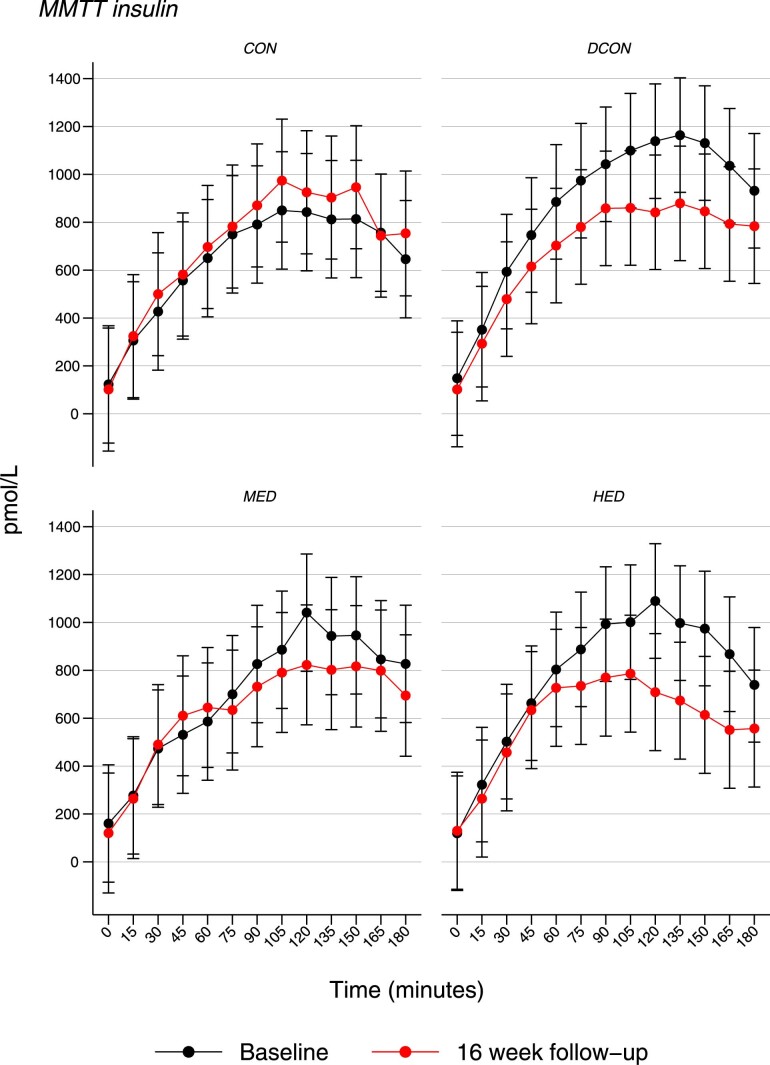
The black circles represent baseline values, and the red circles represents the 16-week follow-up values. Data are represented as the estimated means. Error bars are 95% confidence intervals. Results are adjusted for sex. CON: control group (N = 20 independent samples), DCON: Dietary control group (N = 21 independent samples), MED: Moderate volume exercise (N = 20 independent samples), HED: High volume exercise (N = 21 independent samples).

**Extended Data Fig. 5:**
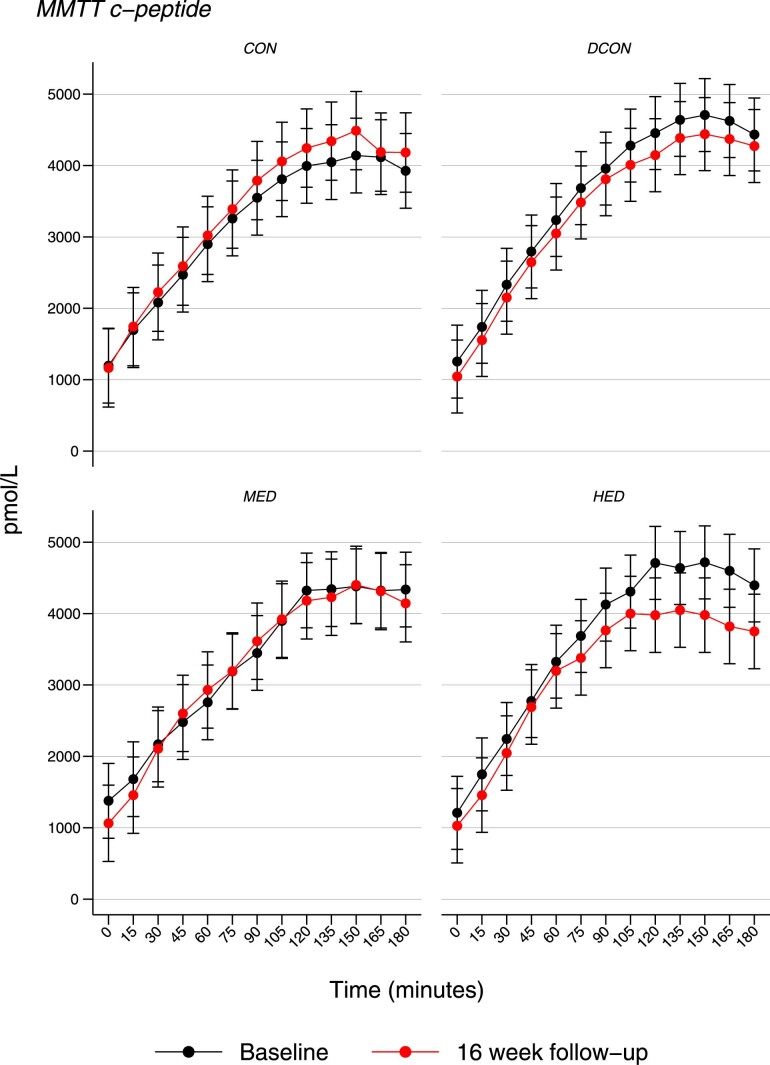
The black circles represent baseline values, and the red circles represents the 16-week follow-up values. Data are represented as the estimated means. Error bars are 95% confidence intervals. Results are adjusted for sex. CON: control group (N = 20 independent samples), DCON: Dietary control group (N = 21 independent samples), MED: Moderate volume exercise (N = 20 independent samples), HED: High volume exercise (N = 21 independent samples).

**Extended Data Fig. 6:**
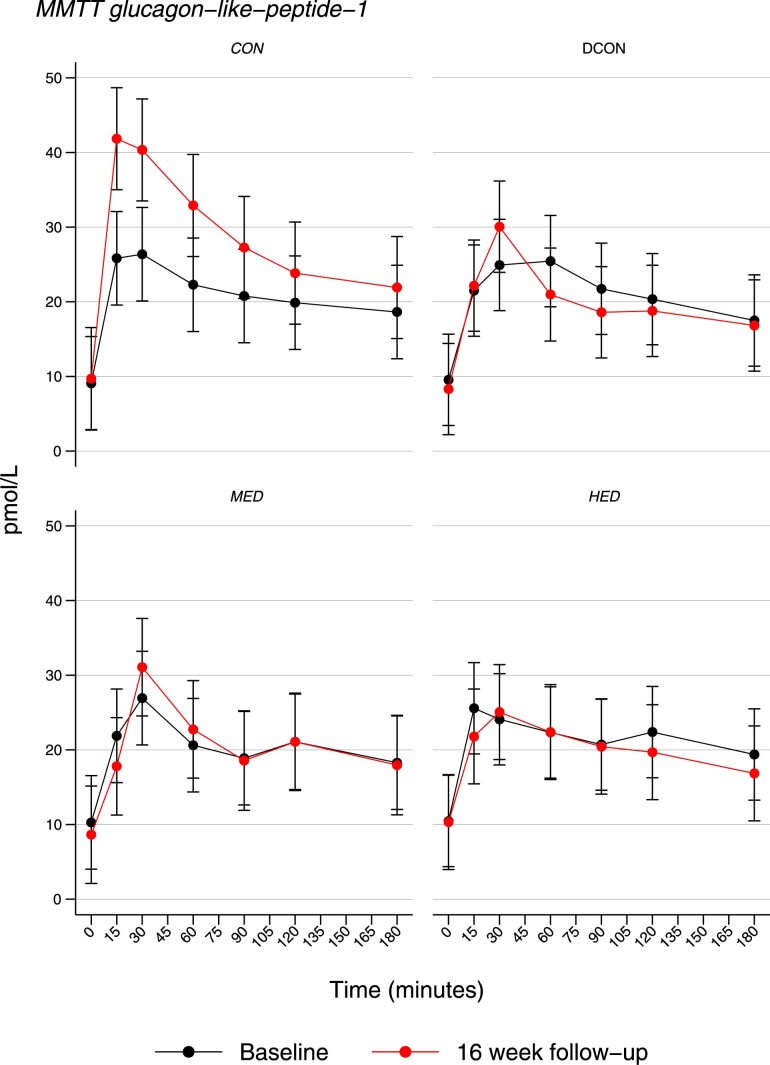
Represented as the estimated means. Error bars are 95% confidence intervals. Results are adjusted for sex. CON: control group (N = 20 independent samples), DCON: Dietary control group (N = 21 independent samples), MED: Moderate volume exercise (N = 20 independent samples), HED: High volume exercise (N = 21 independent samples).

**Extended Data Fig. 7:**
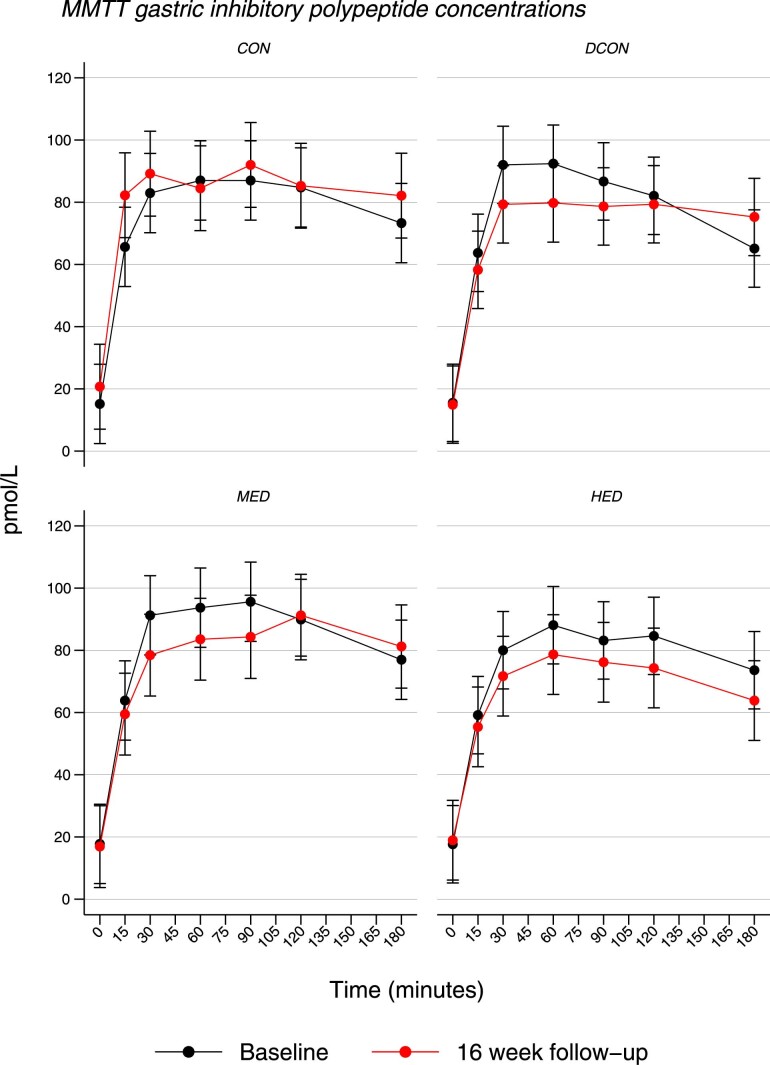
The black circles represent baseline values, and the red circles represents the 16-week follow-up values. Data are represented as the estimated means. Error bars are 95% confidence intervals. Results are adjusted for sex. CON: control group (N = 20 independent samples), DCON: Dietary control group (N = 21 independent samples), MED: Moderate volume exercise (N = 20 independent samples), HED: High volume exercise (N = 21 independent samples).

**Extended Data Fig. 8:**
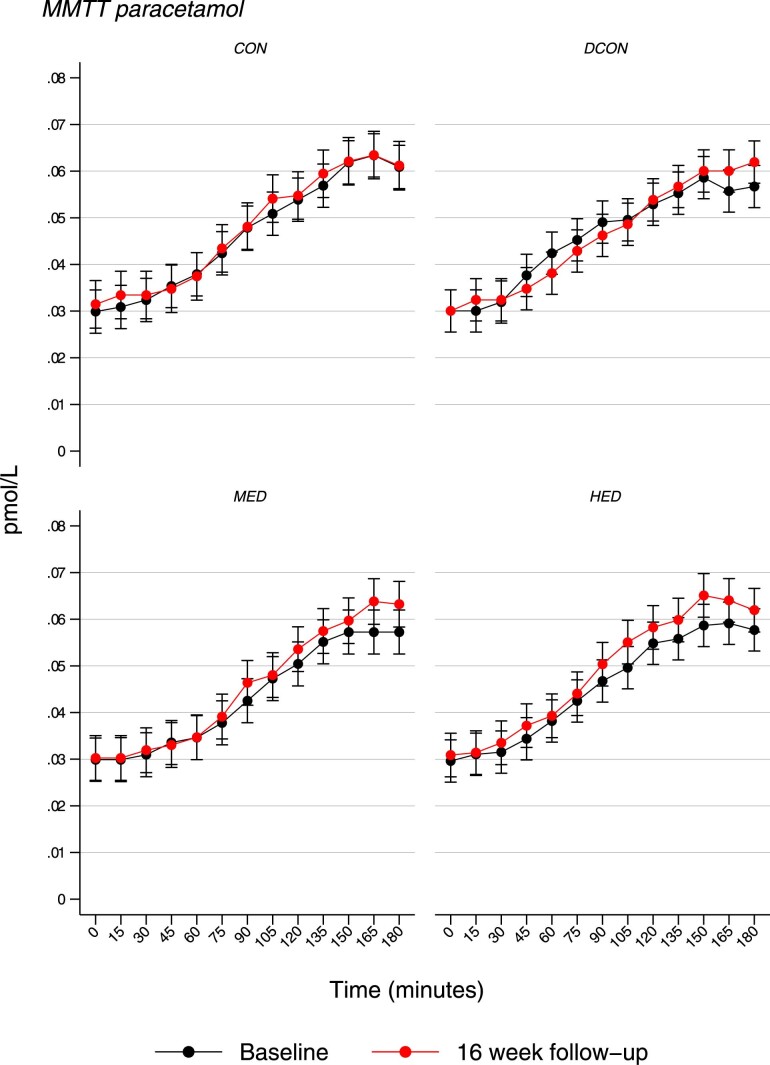
The black circles represent baseline values, and the red circles represents the 16-week follow-up values. Data are represented as the estimated means. Error bars are 95% confidence intervals. Results are adjusted for sex. CON: control group (N = 20 independent samples), DCON: Dietary control group (N = 21 independent samples), MED: Moderate volume exercise (N = 20 independent samples), HED: High volume exercise (N = 21 independent samples).

Postprandial plasma glucose and insulin decreased more in all intervention groups than in CON (total area under the curve (tAUC), *t* = 0–120 min, *P* < 0.001). No differences were observed between intervention groups (Supplementary Tables 10 and 11; incremental AUC (iAUC) is shown in Supplementary Tables 14 and 15). The tAUC for GLP-1 and gastric inhibitory polypeptide (GIP) secretion increased more in CON than in the intervention groups from baseline to follow-up (Supplementary Tables 10 and 11). No differences were observed between the intervention groups (Supplementary Tables 10 and 11 and iAUC in Supplementary Tables 14 and 15). Postprandial responses are presented in Extended Data Figs. 3–8.

#### Body weight

Body weight decreased by 0%, 7%, 10% and 12% from baseline in CON, DCON, MED and HED, respectively, and decreased more in all intervention groups than in CON (Tables 4 and 5). Both MED and HED reduced the body weight by 3.2 kg (*P* = 0.043) and 4.5 kg (*P* = 0.004) more than DCON, respectively, with no difference in changes between the exercising groups. The same pattern was observed for BMI (Tables 4 and 5).

**Table 4:** Within-group changes (0–16 weeks) in cardiometabolic outcomes

	CON	DCON	MED	HED
	Change (95% CI)	Change (95% CI)	Change (95% CI)	Change (95% CI)
**Glycemic control**				
HbA1c, mmol/mol	2 (0; 4)	−5 (−6; −3)	−5 (−7; −3)	−5 (−7; −3)
HbA1c, %	0.1 (0.1; 0.1)	0.1 (−1.1; 0.1)	0.1 (−1.1; 0.1)	0.1 (−1.1; 0.1)
Fasting glucose, mmol/l	0.1 (0.1; 1.1)	−1.1 (−2.1; −1.1)	−2.1 (−3.1; −1.1)	−2.1 (−3.1; −1.1)
Fasting insulin, pmol/l	1 (−23; 25)	−46 (−67; −26)	−60 (−82; −38)	−64 (−86; −43)
Fasting C-peptide, pmol/l	35 (−83; 153)	−216 (−319; −112)	−321 (−432; −209)	−363 (−472; −254)
**Glucose-lowering medication**				
Reduction, no (%), *n* = 65^a^	2.0 (11.1; 0.0)	12.0 (92.3; 0.0)	13.0 (81.3; 0.0)	16.0 (88.9; 0.0)
Discontinuation, no (%), *n* = 65^a^	5.0 (27.8; 0.0)	5.0 (38.5; 0.0)	11.0 (68.8; 0.0)	15.0 (83.3; 0.0)
Intensification, no (%), *n* = 82^b^	9.0 (45.0; 0.0)	0.0 (0.0; 0.0)	2.0 (10.0; 0.0)	2.0 (9.5; 0.0)
**Lipid-lowering medication**				
Intensification, no (%), *n* = 82^b^	5.0 (25.0; 0.0)	1.0 (4.8; 0.0)	1.0 (5.0; 0.0)	0.0 (0.0; 0.0)
**Blood pressure-lowering medication**				
Reduction, no (%), *n* = 47^a^	1.0 (9.1; 0.0)	3.0 (27.3; 0.0)	1.0 (10.0; 0.0)	2.0 (13.3; 0.0)
Discontinuation, no (%), *n* = 47^a^	0.0 (0.0; 0.0)	1.0 (9.1; 0.0)	0.0 (0.0; 0.0)	0.0 (0.0; 0.0)
Intensification, no (%), *n* = 82^b^	1.0 (5.0; 0.0)	1.0 (4.8; 0.0)	0.0 (0.0; 0.0)	1.0 (4.8; 0.0)
**Lipids**				
LDL cholesterol, mmol/l	−1 (−1; 0)	−1 (−1; 0)	−1 (−1; 0)	−1 (−1; 0)
Fasting triglycerides, % change from baseline	−17 (−28; −6)	−41 (−48; −33)	−37 (−45; −28)	−38 (−46; −30)
**Blood pressure**				
Systolic, mmHg	−3 (−6; 0)	−7 (−9; −4)	−9 (−12; −6)	−7 (−10; −5)
Diastolic, mmHg	−4 (−5; −2)	−4 (−6; −3)	−7 (−8; −5)	−7 (−8; −5)
**Physical fitness**				
Absolute VO_2max_, ml/min, *n* = 78	−109.1 (−248.7; 30.7)	−25.1 (−138.1; 87.9)	209.7 (81.3; 338.0)	540.9 (411.6; 670.1)
Relative VO_2max_, ml/kg/min, *n* = 78	−0.8 (−2.3; 0.7)	2.0 (0.8; 3.2)	5.6 (4.2; 6.9)	9.5 (8.2; 10.9)
Watt max, W/kg, *n* = 78	−0.1 (−0.2; 0.1)	0.2 (0.1; 0.3)	0.7 (0.5; 0.8)	0.9 (0.8; 1.1)
1 RM chest press, kg, *n* = 79	0.2 (−3.4; 3.9)	−1.5 (−4.4; 1.4)	−1.7 (−4.6; 1.2)	5.5 (2.7; 8.4)
1 RM chest press, kg/kg body weight, *n* = 79	0.0 (0.0; 0.1)	0.0 (0.0; 0.1)	0.0 (0.0; 0.1)	0.1 (0.1; 0.2)
1 RM leg extension, kg, *n* = 81	4.2 (0.1; 8.3)	−1.2 (−4.8; 2.3)	2.1 (−1.6; 5.9)	2.1 (−1.6; 5.7)
1 RM leg extension, kg/kg body weight, *n* = 81	0.1 (0.0; 0.1)	0.1 (0.0; 0.1)	0.1 (0.1; 0.1)	0.1 (0.1; 0.2)
**Body anthropometrics**				
Body weight, kg	−0.3 (−2.6; 2.0)	−7.4 (−9.5; −5.3)	−10.6 (−12.8; −8.3)	−11.9 (−14.1; −9.7)
BMI, kg/m^2^	−0.1 (−0.8; 0.6)	−2.4 (−3.1; −1.7)	−3.4 (−4.1; −2.7)	−3.8 (−4.5; −3.1)

Data are estimated means with 95% confidence intervals. Data were analyzed using a constrained baseline longitudinal model (two-sided). No corrections for multiple comparisons were performed. Reduction is defined as at least one step down on the predefined algorithm. Discontinuation is defined as discontinuation of all drugs when therapeutic target was met. Intensification is defined as at least one step up on the predefined algorithm. ^a^Participants on medication at baseline (denominator). ^b^All participants (denominator).

**Table 5:** Pairwise comparisons of the change in cardiometabolic outcomes

	HED vs. CON^a^		MED vs. CON^a^		DCON vs. CON^a^		HED vs. DCON^a^		MED vs. DCON^a^		HED vs. MED^a^		Global *P*
	MD (95% CI)	*P*	MD (95% CI)	*P*	MD (95% CI)	*P*	MD (95% CI)	*P*	MD (95% CI)	*P*	MD (95% CI)	*P*	
**Glycemic control**													
HbA1c, mmol/mol	−7 (−9; −5)	<0.001	−7 (−9; −5)	<0.001	−7 (−9; −5)	<0.001	0 (−3; 2)	0.75	0 (−2; 2)	0.93	0 (−2; 2)	0.82	<0.001
HbA1c, %	−1.1 (−1.1; 0.1)	<0.001	−1.1 (−1.1; 0.1)	<0.001	−1.1 (−1.1; 0.1)	<0.001	0.1 (0.1; 0.1)	0.76	0.1 (0.1; 0.1)	0.97	0.1 (0.1; 0.1)	0.79	<0.001
Fasting glucose, mmol/l	−2.1 (−3.1; −1.1)	<0.001	−2.1 (−3.1; −1.1)	<0.001	−2.1 (−3.1; −1.1)	<0.001	0.1 (−1.1; 0.1)	0.30	0.1 (−1.1; 0.1)	0.31	0.1 (−1.1; 1.1)	1.00	<0.001
Fasting insulin, pmol/l	−65 (−97; −34)	<0.001	−61 (−93; −29)	<0.001	−47 (−78; −17)	0.003	−18 (−47; 11)	0.23	−13 (−43; 16)	0.38	−5 (−35; 26)	0.77	<0.001
Fasting C-peptide, pmol/l	−398 (−555; −240)	<0.001	−356 (−515; −196)	<0.001	−250 (−404; −96)	0.001	−147 (−295; 0)	0.050	−105 (−255; 44)	0.17	−42 (−195; 111)	0.59	<0.001
**Glucose-lowering medication**													
Reduction, OR	3.7 (1.9; 9.4)	<0.001	5.4 (2.0; 20.1)	<0.001	71.5 (6.2; 4341.4)	<0.001	0.8 (0.1; 3.8)	1.000	0.4 (0.0; 5.4)	0.77	1.8 (0.2; 24.8)	0.88	<0.001
Discontinuation, OR	2.3 (1.3; 4.5)	0.002	2.3 (1.0; 5.7)	0.039	1.6 (0.3; 9.6)	0.81	2.7 (1.1; 7.9)	0.028	3.4 (0.6; 21.6)	0.21	2.2 (0.3; 17.4)	0.55	0.003
Intensification, OR	0.5 (0.2; 0.9)	0.025	0.4 (0.1; 0.9)	0.031	0.1 (0.0; 0.34^b^)	0.001	1.6 (0.4; ∞^b^)	0.49	2.6 (0.2; ∞^b^)	0.46	1.0 (0.1; 14.4)	1.000	0.001
**Lipid-lowering medication**													
Intensification, OR	0.5 (0.0; 0.98^b^)	0.041	0.4 (0.1; 1.3)	0.18	0.2 (0.0; 1.6)	0.16	1.0 (0.0; 6.24^b^)	1.00	1.1 (0.0; 86.7)	1.00	1.0 (0.0; 37.14^b^)	0.98	0.031
**Blood pressure-lowering medication**													
Reduction, OR	1.2 (0.4; 4.6)		1.1 (0.1; 9.8)		3.5 (0.2; 216.2)		0.7 (0.2; 2.1)		1.1 (0.1; 9.8)		1.4 (0.1; 90.6)		0.70
Discontinuation, OR	NE (0.0; 0.0)	NE	NE	NE	NE	NE	NE	NE	NE	NE	NE	NE	NE
Intensification, OR	1.0 (0.2; 4.3)		1.0 (0.0; 6.24^b^)		1.0 (0.0; 78.5)		1.0 (0.1; 9.1)		1.1 (0.0; 40.95^b^)		1.0 (0.0; ∞^b^)		1.00
**Lipids**													
LDL cholesterol, mmol/l	0 (0; 0)		0 (0; 0)		0 (0; 0)		0 (0; 0)		0 (0; 0)		0 (0; 0)		0.99
Fasting triglycerides, % change from baseline	−25 (−37; −12)	0.001	−24 (−35; −10)	0.002	−29 (−39; −16)	<0.001	5 (−11; 23)	0.41	7 (−9; 25)	0.57	−2 (−17; 15)	0.80	<0.001
**Blood pressure**													
Systolic, mmHg	−4 (−7; −1)	0.021	−6 (−9; −2)	0.001	−4 (−7; 0)	0.039	0 (−4; 3)	0.79	−2 (−6; 1)	0.18	2 (−2; 5)	0.29	0.010
Diastolic, mmHg	−3 (−5; −1)	0.011	−3 (−5; −1)	0.011	−1 (−3; 1)	0.46	−2 (−4; 0)	0.063	−2 (−4; 0)	0.059	0 (−2; 2)	0.95	0.017
**Physical fitness**													
Absolute VO_2max_, ml/min, *n* = 78	649.9 (461.2; 838.7)	<0.001	318.7 (130.7; 506.7)	0.001	84.0 (−94.3; 262.2)	0.36	566.0 (396.0; 736.0)	<0.001	234.7 (65.6; 403.9)	0.007	331.2 (151.0; 511.4)	<0.001	<0.001
Relative VO_2max_, ml/kg/min, *n* = 78	10.3 (8.3; 12.3)	<0.001	6.4 (4.4; 8.4)	<0.001	2.8 (0.9; 4.7)	0.004	7.5 (5.7; 9.3)	<0.001	3.6 (1.8; 5.3)	<0.001	4.0 (2.1; 5.9)	<0.001	<0.001
Watt max, W/kg, *n* = 78	1.0 (0.8; 1.2)	<0.001	0.7 (0.5; 0.9)	<0.001	0.2 (0.1; 0.4)	0.008	0.8 (0.6; 0.9)	<0.001	0.5 (0.3; 0.6)	<0.001	0.3 (0.1; 0.5)	0.002	<0.001
1 RM chest press, kg, *n* = 79	5.3 (0.7; 9.9)	0.025	−2.0 (−6.6; 2.7)	0.41	−1.7 (−6.4; 2.9)	0.46	7.0 (3.0; 11.1)	0.001	−0.2 (−4.3; 3.8)	0.91	7.3 (3.2; 11.3)	<0.001	0.001
1 RM chest press, kg/kg body weight, *n* = 79	0.1 (0.1; 0.2)	<0.001	0.0 (0.0; 0.1)	0.31	0.0 (0.0; 0.1)	0.53	0.1 (0.1; 0.2)	<0.001	0.0 (0.0; 0.1)	0.66	0.1 (0.1; 0.1)	<0.001	<0.001
1 RM leg extension, kg, *n* = 81	−2.1 (−7.6; 3.3)		−2.1 (−7.6; 3.4)		−5.5 (−10.9; −0.1)		3.3 (−1.7; 8.4)		3.4 (−1.8; 8.5)		0.0 (−5.2; 5.2)		0.241
1 RM leg extension, kg/kg body weight, *n* = 81	0.1 (0.0; 0.1)	0.028	0.0 (0.0; 0.1)	0.12	0.0 (−0.1; 0.1)	0.91	0.1 (0.0; 0.1)	0.013	0.1 (0.0; 0.1)	0.074	0.0 (0.0; 0.1)	0.52	0.035
**Body anthropometrics**													
Body weight, kg	−11.6 (−14.8; −8.4)	<0.001	−10.3 (−13.5; −7.1)	<0.001	−7.1 (−10.2; −4.0)	<0.001	−4.5 (−7.5; −1.4)	0.004	−3.2 (−6.2; −0.1)	0.043	−1.3 (−4.5; 1.8)	0.40	<0.001
BMI, kg/m^2^	−3.7 (−4.7; −2.7)	<0.001	−3.3 (−4.3; −2.3)	<0.001	−2.3 (−3.3; −1.3)	<0.001	−1.4 (−2.4; −0.5)	0.004	−1.0 (−1.9; 0.0)	0.044	−0.4 (−1.4; 0.5)	0.38	<0.001

Data are estimated means with 95% confidence intervals unless stated otherwise. Reduction, Discontinuation and Intensification are defined as in Table 4. NE, not estimable. If Global *P* is >0.1, *P* values are not reported for between-group comparisons. Continuous data were analyzed using a constrained baseline longitudinal model, and ordinal variables were analyzed using exact logistic regression. *P* values are two-sided. No corrections for multiple comparisons were performed because of sparse data in medication changes. ^a^Reference category for the logistic regression analyses. When zero-event data were observed, a continuity correction was used that was inversely proportional to the relative size of the opposite group.

#### Other cardiometabolic markers

HbA1c decreased 0.6% (7 mmol/mol) more in all intervention groups than in CON (*P* < 0.001), but no differences were observed between intervention groups (Tables 4 and 5). The same pattern was observed for fasting glucose, fasting insulin, fasting C-peptide, fasting triglycerides and systolic blood pressure (Tables 4 and 5). All intervention groups had reduced diastolic blood pressure, and the reduction was greater in HED and MED than in DCON. No reductions in low-density lipoprotein cholesterol (LDL-C) were observed. Physical fitness defined as maximum oxygen consumption in ml O_2_/min (VO_2max_) increased in MED and HED compared with CON and DCON, and HED improved more than MED. VO_2max_ relative to body weight defined as ml O_2_/min/kg (relative VO_2max_) changed by −3%, 8%, 23% and 39% in CON, DCON, MED and HED, respectively (Table 4). HED improved absolute and relative-to-body-weight 1 repetition maximum (RM) chest press compared with all of the other groups, whereas 1 RM leg extensions relative to body weight improved in HED only when compared with CON and DCON (Table 5).

### Sensitivity analyses

The multiple imputation analyses on the primary and secondary outcomes agreed with the primary analyses (Supplementary Table 13).

### Post hoc analyses

As a post hoc outcome, the need for medication, after completion of follow-up testing, was calculated based on the prespecified algorithm. Reductions of glucose-lowering medication were 11%, 92%, 81% and 89% in CON, DCON, MED and HED, respectively (Table 4). The corresponding numbers for discontinuations of glucose-lowering medication were 28%, 39%, 69% and 83%, respectively (Table 4). The odds of reductions and discontinuations were higher in all intervention groups than in CON (*P* < 0.05). No differences were observed in the odds of reductions of glucose-lowering medication between the intervention groups, but the odds of discontinuations were higher for HED than for CON (odds ratio (OR), 2.7 (95% CI, 1.1; 7.9); Table 5). Although the odds of discontinuation were higher in MED than in DCON (OR, 3.4 (95% CI, 0.6; 21.6)), it did not reach statistical significance (*P* = 0.2; Table 5). The odds of discontinuations were similar between HED and MED, and no differences were observed in any group comparison for other medications (Table 5).

The role of weight loss on the primary and secondary outcomes was explored in a post hoc statistical mediation analysis (Supplementary Table 16). It revealed that the treatment effect mediated by weight loss on late-phase DI was similar across the exercising groups and accounted for around 50–60% of the total effect, whereas 70% of the treatment effect was mediated by weight loss in DCON. Regarding late-phase ISI, the pattern was similar for the exercising groups, but for DCON, the weight loss was entirely responsible for the treatment effect. Weight loss did not explain the increase in late-phase ISR.

## Discussion

One of our main findings is that all intervention groups improved beta-cell function, as expressed by late-phase DI, more than standard care. Furthermore, adding exercise to diet-induced weight loss improved beta-cell function more than diet-induced weight loss or standard care alone. This seemed to be achieved by additional increases in insulin sensitivity induced by exercise in a dose-dependent manner. However, the secondary and exploratory outcomes did not uniformly support the linear dose–response relationship observed for the primary outcome.

There is a paucity of studies investigating the role of exercise and exercise volume in conjunction with diet-induced weight loss, but our observations are in line with previous findings suggesting that high volumes of exercise without a concomitant dietary intervention improve first-phase and/or late-phase DI in people with prediabetes and T2D^5,12,23^. In line with other studies, our data support that the exercise component increases DI due to increases in insulin sensitivity rather than increased insulin secretion^5,12,23,24^. In contrast, other studies have shown that exercise may increase insulin secretion and not insulin sensitivity in people with dysglycemia^6,8^. One reason for the discrepancy may be the higher glucose clamp levels (25 mmol/l) used in ref. ^6^ compared with the ~13 mmol/l plasma glucose used in our study. At clamp levels closer to our target (~13 mmol/l plasma glucose), the researchers in ref. ^6^ also did not observe an increase in insulin secretion. The differences could also relate to the exercise intensities in the studies wherein, for a given increase in insulin sensitivity, it has been shown that high-intensity exercise results in a larger reduction in insulin secretion than low-intensity to moderate-intensity exercise^12^.

Although we did not observe a difference in late-phase ISR with increased exercise volume compared with diet-induced weight loss alone, all intervention groups exhibited similar increases in late-phase ISR. Therefore, it could be speculated that diet-induced weight loss alone might explain this observation and that a weight loss of ~7.5% body weight may be sufficient to re-establish late-phase ISR in this study population. Supporting this, a previous study from our group, using the same intervention protocol as for HED, also increased DI. Consistent with our current findings, only the improvement in insulin sensitivity index, and not the small increase in insulin secretion, was associated with exercise volume in that study^10^. Taken together, these findings suggest that changes in insulin sensitivity are more exercise driven, whereas changes in insulin secretion are primarily driven by weight loss. In further support of this, previous studies have shown an increased insulin secretory capacity after caloric restriction with no or marginal improvement in peripheral insulin sensitivity; thus, first-phase, late-phase and total-phase DI are explained mainly by increases in insulin secretion^3,18,25^. The increased first-phase ISR following diet-induced weight loss is consistent with findings of other studies^3,26^ and may relate to a decrease in fasting plasma glucose (which was comparable between the intervention groups in our study)^27^. However, it was surprising that exercise was associated with an attenuation of the increase in first-phase ISR induced by diet-induced weight loss. When first-phase DI was calculated (correcting the ISR for insulin sensitivity), the increase was larger in the exercising groups than with diet-induced weight loss alone. Although there seems to be a consensus that regaining first-phase insulin secretion is characteristic of T2D remission, we found a reduction when exercise was added to a diet-induced weight loss intervention. An explanation may relate to both improved insulin sensitivity and glucose effectiveness observed with exercise^5,16^, or could be ascribed to a blunted insulin secretion during hyperglycemia, GIP stimulation and arginine stimulation after high volumes of exercise^28–30^. The HED group may have experienced an exercise-induced blunting of insulin secretion whereas MED did not, which could also explain why there were no further increases in ISR, first-phase DI, GLP-1 stimulation and arginine stimulation despite the largest late-phase ISI being in HED. The metabolic consequences of a slightly blunted insulin secretion in people with T2D are unknown. Nevertheless, exercise-induced decreases in insulin secretion during concomitant increases in insulin sensitivity are consistently found in individuals with prediabetes and/or obesity, as well as in healthy people^12,23,28^. Given that insulin secretion physiologically counterbalances insulin sensitivity as a homeostatic response^15,31^, an exercise-induced increase in insulin sensitivity added to diet-induced weight loss may reduce the demand on beta cells, offering beta-cell rest and therefore preserving beta-cell health.

In this study, both late-phase DI and late-phase ISI increased linearly with increasing treatment intensity. However, a similar relationship was not observed during the MMTT nor in first-phase DI or the GLP-1 and arginine stimulations. Although speculative, this may relate to the route of glucose administration. Recently, it was described that the increased insulin secretion observed after oral administration of glucose compared with intravenous administration might be accompanied by a compensatory decrease in insulin sensitivity^32^. Furthermore, the incretin response during the MMTT might activate a GIP-induced vasoconstriction in the microvasculature of skeletal muscles^33^, which could dampen a difference in insulin action between the exercise groups achieved by exercise-induced skeletal muscle capillarization^34^.

These findings suggest that there is only a limited effect of increasing exercise volume from three to six sessions weekly in the context of diet-induced weight loss. Likewise, although weight loss was larger in both exercise groups compared with diet-induced weight loss alone, there was no apparent additional weight loss when doubling the exercise volume from three to six sessions per week. In contrast, the increase in both absolute and relative VO_2max_ was positively associated with exercise dose, with only marginal differences in maximal strength.

Although a weight loss of ≥5% may increase beta-cell function slightly, a weight loss of ≥11% may be necessary to maximize an increase in peripheral insulin sensitivity in people with obesity^2^. However, diet-induced weight losses of ≥15 kg resulting in significant improvements in beta-cell function have been reported in people with T2D without concomitant increases in peripheral insulin sensitivity^3^.

Still, diet-induced weight loss may primarily improve hepatic (central) insulin sensitivity and beta-cell insulin secretory capacity through reductions in visceral and ectopic fat (that is, in liver and pancreas) that confer a dose-dependent increase in beta-cell function^3,4^. This might explain why the post hoc statistical mediation analysis suggested that the role of weight loss on the intervention effect mediated the entire effect of late-phase ISI in DCON. In contrast, exercise mainly improves peripheral insulin sensitivity^35^ and may explain why only 50–60% of the intervention effect was mediated by weight loss on late-phase ISI when exercise was added to the diet. As such, the small additional weight loss observed in the exercise groups compared with DCON most likely does not alone explain the add-on effect on late-phase ISI. These findings support that exercise may improve beta-cell function by increasing peripheral insulin sensitivity beyond the effects of weight reduction alone^34–36^.

Interestingly, weight loss completely mediated the intervention effects in the oral DI and oral ISI, suggesting that weight loss becomes the most important signal in the context of an oral mixed meal and normal homeostatic postprandial regulation. Although speculative, this may relate not only to the complex neuronal and endocrine organ crosstalk, but also to the meal composition wherein certain amino acids and fatty acids regulate insulin secretion, as well as skeletal muscle microvascular blood flow.

### Limitations

Our findings must be interpreted in the context of the limitations of the study. First, the sample size was based on a previous study including up to five aerobic exercise sessions per week. Thus, the lack of differences between MED and HED or DCON and MED could be a type 2 error due to low statistical power. However, given the consistent signal across most beta-cell indices, the results can be interpreted with confidence. Second, we assessed beta-cell DI with a hyperglycemic clamp. Although the hyperglycemic clamp is the gold standard for beta-cell function^15^, it is an unphysiological assessment that limits physiological translation. However, we clamped the glucose level at only 5.4 mmol/l above the fasting glucose level, attempting to mimic postprandial glucose levels. Furthermore, we assessed beta-cell indices during an MMTT to compare the supraphysiological hyperglycemic clamp (glucose levels ~13 mmol/l at baseline and follow-up) to the physiological conditions during the MMTT (peak glucose levels ~16 mmol/l during MMTT at baseline). Because we observed a consistent pattern between the hyperglycemic clamp and MMTT, this allows for translating the findings from the hyperglycemic clamp to a physiological context. Third, we applied pharmacological constraints, and the participants were all relatively newly diagnosed and pharmacologically well regulated before randomization as well as throughout the study. Therefore, we cannot directly translate the results to people with longer T2D duration, treated with other pharmacological agents, or who have poor glycemic control. However, as our findings are consistent with pharmacological weight-loss trials^37^, they may still have clinical implications. Fourth, the intervention was only 16 weeks. Diminished benefits concerning beta-cell indices when going from three to six exercise sessions per week could be due to ceiling effects for the time course of the intervention. Hence, three exercise sessions per week combined with a 25% energy deficit may almost fully saturate the rate of adaptation for the mechanisms influencing beta-cell function. Furthermore, organ-specific changes in response to chronic exercise may occur on different time courses and will also reflect individual responses to exercise^35,38^. Thus, 16 weeks may have been too short to see significant deviations between MED and HED or even from DCON. Fifth, we did not use the gold standard hyperinsulinemic-euglycemic clamp to assess insulin sensitivity; however, the hyperglycemic clamp provides a reliable measure of glucose disposal^15,39^. Moreover, we observed that late-phase EGP did not change while *R*_d_ increased compared with CON, suggesting an increased peripheral insulin sensitivity and not hepatic insulin sensitivity. Sixth, we assessed dietary adherence using self-reporting, which may include information bias^40^. Seventh, although DI is considered the most accurate assessment of beta-cell function^15^, the relationship between insulin secretion and insulin sensitivity is not consistently hyperbolic across levels of glucose tolerance, BMI or measurements of DI^31^. Moreover, an increased beta-cell DI does not necessarily imply improved beta-cell health. This is because an increase in DI via an increased demand of insulin secretion to compensate for decreased insulin sensitivity (that is, higher allostatic load) has been associated with deterioration of beta-cell function compared with increasing DI via improved insulin sensitivity^31,41,42^. Therefore, we evaluated both insulin secretion and insulin sensitivity for calculating beta-cell DI, and our results are in line with preclinical and clinical studies suggesting that increasing beta-cell DI through insulin sensitivity is beneficial for beta-cell health.

## Conclusion and perspectives

Among adults with T2D within 7 years of diagnosis, exercise in addition to diet-induced weight loss increases late-phase DI across a 16-week intervention. The most pronounced benefits were observed with exercise six times per week.

The direction of the exercise effects on the oral DI and oral ISI are consistent with an additional benefit when added to diet-induced weight loss. In contrast to the linear dose–response relationship observed with glucose stimulation only, the oral DI and oral ISI displayed a curve-linear relationship with diminished returns when comparing three and six exercise sessions per week. Hence, data from the meal stimulation suggest that increasing the exercise dose beyond three times per week may be redundant to gain additional benefits of exercise on beta-cell function when performed in conjunction with diet-induced weight loss. Further research is needed to confirm this.

## Methods

### Study design

The study was a 16-week, parallel-group, four-arm, assessor-blinded, randomized clinical trial conducted between February 2019 and October 2021 at the Centre for Physical Activity Research (CFAS), Rigshospitalet, Copenhagen, Denmark. The study was preregistered at ClinicalTrials.gov (NCT03769883) and was approved by the Scientific Ethical Committee of the Capital Region of Denmark (approval number H-18038298) before the commencement of any study procedures. Guidelines from the Helsinki Declaration were followed, and the data are reported following the CONSORT guideline for multi-arm trials^43^ and the REPORT standards^43^. The study protocol for this clinical trial is available in the Supplementary Information and has been published previously^22^. The prespecified full statistical analysis plan (SAP) was completed and uploaded to our website before commencing any statistical analyses (https://aktivsundhed.dk/images/docs/SAP_doseex_nov21.pdf).

### Participants and eligibility criteria

Participants were recruited through the media, municipalities and the Danish Health Data Authorities. The potential participants contacted the study nurse and completed the screening process before the medical examination. The main inclusion criteria were (1) men and women aged 18–80 years, (2) diagnosed with T2D within <7 years, (3) no current treatment with insulin and (4) BMI > 27 kg/m^2^ and <40 kg/m^2^. All participants provided written and oral informed consent before any testing.

### Interventions

CON received standard care and was encouraged to maintain habitual physical activity and dietary habits throughout the study. DCON received standard care and dietary intervention. MED received standard care, dietary intervention and an exercise intervention with two aerobic training sessions per week and one combined aerobic and resistance training session per week, totaling 150–165 min of exercise training per week. HED received the standard care and dietary interventions as described above but had twice as much exercise as MED, with a total of four aerobic training sessions per week and two combined aerobic and resistance training sessions per week, totaling 300–330 min of exercise training per week.

#### Standard care component

Standard care included pharmacological management of blood glucose, blood lipids and blood pressure according to a prespecified algorithm and was managed by an endocrinologist who was blinded for participant allocation^22^. To minimize an influence on the findings of poor glucose control upon study entry, medical standardization was introduced according to the prespecified treat-to-target algorithm for 6 weeks before the baseline measurements. Furthermore, the pharmacological treatment was evaluated according to the algorithm following baseline measurements and at week 12 of the intervention. The treatment targets were in line with current guidelines. In adjunct to the algorithm, pharmacological treatment was adapted to mitigate subjective signs of hypotension or hypoglycemia. Blood lipids, blood pressure and blood glucose were measured before the intervention and 4, 12 and 16 weeks into the intervention. In case of any adverse events, the participants were advised to contact the study nurse. At each visit, the study nurse interviewed all participants about potential adverse events. The adverse events definition followed ICH E2A guidelines^44^.

#### Dietary component

Daily energy requirements were estimated using the age-adjusted Oxford equation^45^. The dietary intervention aimed at ~25–30% energy deficit per day with a macronutrient distribution within the range of 45–60 energy percent (E%) carbohydrate, 15–20E% protein and 20–35E% fat (<7E% saturated fat). The intervention consisted of individualized recommendations and recipes. A clinical dietician implemented the plan at three sessions during the intervention, and adjustments were performed based on self-reported, 3-day food records.

#### Exercise component

The exercise intervention consisted of both aerobic and resistance training, and the first 2 weeks served as a familiarization period. The aerobic training sessions of 30-min duration had a target intensity of 60–100% of maximal heart rate (HR_max_). Throughout the intervention, the relative time spent exercising in intensity zone 80–100% of HR_max_ was increased, and the relative time spent in the intensity zone 60–79% of HR_max_ was reduced accordingly. Resistance training was added in combined sessions with 30 min of aerobic training and 30–45 min of resistance training. The resistance training consisted of three sets in the main muscle groups, for example, chest press, leg press, back row, and leg extension. The 8–12 repetitions aimed at a resistance consistent with 0–3 repetitions in reserve^46^. All heart-rate profiles were recorded during the exercise interventions (Polar V800), and all training sessions were supervised by educated trainers.

### Experimental days

Two experimental days were conducted at baseline and repeated at 16-week follow-up. Forty-eight hours before the experimental days, the participants were instructed to discontinue glucose-lowering medication use and refrain from any exercise. Moreover, no alcohol or caffeine was permitted 24 h before the visits, and the participants were instructed to maintain their habitual diet. The participants arrived at the testing facilities at 07:30 am after an overnight fast (≥10 h fasting). Experimental days 1 and 2 were planned to be separated by 1 week.

#### Experimental day 1

The participants completed a 3-h MMTT. The liquid meal was prepared using 400 ml of Nestlé Resource with an additional 36 g of dextrose (total energy content, 735 kcal; E%, 64/24/12 carbohydrate/fat/protein). Paracetamol (1.5 g) was added to assess gastric emptying. Body weight was measured with an electronic scale, and height was measured with a Holtain stadiometer according to standard procedures. VO_2max_ was assessed using indirect calorimetry (Quark CPET, Cosmed) on a Monark LC4 bicycle (Monark Exercise). The test was performed with a 5-min warm-up followed by increases of 20 watts/min until exhaustion. Maximum muscle strength was assessed by two exercises performed in resistance training machines (chest press, leg extension) via estimating the maximum weight (kg) that could be lifted once with a full range of motion with proper form (that is, 1 RM).

#### Experimental day 2

A three-stage hyperglycemic clamp was performed. After baseline blood sampling, a priming bolus of [6,6-^2^H_2_]glucose was injected intravenously and a continuous tracer infusion was initiated. The bolus dose and infusion rate of the tracer depended on the participant’s fasting glucose level and body weight as described elsewhere^5^. After 2 h of tracer infusion, hyperglycemia was introduced by clamping glucose at 5.4 mM above fasting glucose (whereas the absolute postintervention clamp glucose level was equal to the preintervention clamp level). An initial increase in blood glucose was brought about by a square-wave glucose infusion lasting 15 min. After this, the glucose concentration was kept constant by adjusting GIRs based on blood glucose measurements (ABL 8 series, Radiometer) performed every 5 min according to an automated algorithm^5^. After 2 h of hyperglycemia, a continuous GLP-1 infusion was initiated at a rate of 0.5 pmol/kg/min, and after 1 h of hyperglycemia + GLP-1 infusion, an intravenous bolus of arginine hydrochloride (5 g given over 30 s) was administered to provide a maximal stimulus to the beta cells, leading to secretion of remaining intracellular vesicles of insulin. Before baseline sampling, the participant voided. Every time the participant voided during the clamp, the urine was accumulated, and urinary glucose concentration was measured at the end of the procedure.

#### Free-living measurements

Assessments of free-living physical activity and blood pressure were recorded by the participants between the 2 study days. Physical activity was also assessed with physical activity monitors (AX3, Axivity) for 7 consecutive days. Blood pressure was assessed with home-based resting measurements across 3 days, including three measurements morning and evening. Furthermore, a 3-day record of total dietary intake was completed at baseline, during the intervention period (at weeks 4 and 12), and during the 3 days leading up to follow-up testing.

#### Blood sample analyses

Blood samples (plasma insulin, C-peptide, glucose, HbA1c, LDL-C, triglycerides and paracetamol) were analyzed at the Department of Clinical Biochemistry, Rigshospitalet, using standard procedures. GLP-1 and GIP were analyzed using in-house carboxy-terminal radioimmunoassays. The total GLP-1 assay (codename 89390) is based on the amidated COOH terminus and therefore measures GLP-1(7–36)NH_2_ and GLP-1(9–36)NH_2_. The assay results, therefore, reflect the secretion rate of GLP-1 (refs. ^47,48^). The total GIP assay (codename 80867) reacts fully with intact GIP and amino-terminally truncated forms^49^. The glucose tracer [6,6-^2^H_2_]glucose was used for whole-body measurements of *R*_a_ and *R*_d_ of glucose during steady-state hyperglycemia and was calculated using non-steady-state equations^50^ adapted for stable isotopes^51,52^.

### Participant compensation

All participants received up to DKK 6,000 (€800) in total to cover lost earnings, transport and discomfort. The transaction was completed upon completion of the study (all four full laboratory days (V1, V2, V6 and V7) or upon withdrawal). For every completed day of laboratory testing, participants received DKK 1,000. Moreover, DKK 500 in compensation was added per biopsy (up to four in total). To prevent loss to follow-up in the CON group, we offered three supervised training sessions and a free 16-week membership in a fitness center following final testing.

### Outcomes

#### Primary outcome

The primary outcome was the change in late-phase DI from baseline to the 16-week follow-up, reflecting the beta-cell response during the last 30 min of the hyperglycemic stage^15^. DI was calculated as the product of late-phase ISR and late-phase ISI (designated secondary outcomes, see below).

#### Secondary outcomes

Secondary outcomes were prespecified in the SAP (designated ‘Major secondary outcomes’ in the SAP) and included the late-phase ISR, late-phase ISI derived during the last 30 min of the hyperglycemic stage, and the oral DI, oral ISI and oral ISR derived from the MMTT^53^. Late-phase ISR was calculated from the deconvoluted C-peptide measurements^54^ and subsequently normalized to ambient blood glucose concentrations. Late-phase ISI was calculated as the GIR divided by the product of insulin and glucose^39^. Oral DI was calculated as the product of oral ISI and oral ISR. Oral ISI (the Matsuda index) was calculated as 10,000/√(fasting glucose × fasting insulin) × (mean glucose_0–120min_ × mean insulin_0–120min_), and oral ISR was calculated as the tAUC for glucose divided by the tAUC for insulin from time 0 to 120 min during the MMTT^53^.

#### Exploratory outcomes

The exploratory outcomes (designated ‘Other secondary outcomes’ in the SAP) included the change (baseline to 16-week follow-up) in first-phase ISR, EGP, first-phase DI, ISI and ISR, as well as HbA1c, LDL-C, fasting glucose, fasting insulin, fasting C-peptide, fasting triglycerides, systolic blood pressure, diastolic blood pressure, body weight, absolute VO2_max_, relative VO2_max_, 1 RM for chest press and leg extension (both absolute and relative to body weight), and tAUC and iAUC in glucose, insulin, C-peptide, GLP-1, GIP and paracetamol from the MMTT. AUCs for the different time periods were calculated using the trapezoidal rule. *R*_a_ and *R*_d_ were calculated from glucose tracers during clamp-induced steady-state hyperglycemia. Adverse events were self-reported.

#### Post hoc outcomes

Post hoc outcomes included intensification (yes or no), reduction (yes or no) and discontinuation (yes or no) for glucose-lowering and blood pressure-lowering medications. Due to restrictions in our pharmacological treatment algorithm regarding lipid-lowering medications, only intensifications were assessed for this outcome.

### Randomization and blinding

The participants were randomly allocated to the four intervention arms upon successful completion of the baseline measurements. An independent statistician (author R.C.) prepared a computer-generated randomization schedule in a ratio of 1:1:1:1, stratified by sex. To ensure concealment, the (permuted) block sizes were not disclosed. The schedule was forwarded to a secretary who was not involved in any study procedures and stored on a password-protected computer. Sequentially numbered, opaque, sealed envelopes were prepared and stored in a locked cabinet before commencing the recruitment. The envelopes were lined with aluminum foil to render the envelope impermeable to intense light. Following the conclusion of the hyperglycemic clamp, the appropriate envelope was opened by a study nurse, and the participant was informed about the allocation stated on the card inside the envelope. The participant received the allocation in a closed room. As such, the participants were blinded for treatment allocation until after the completion of the hyperglycemic clamp. Following the baseline assessment, blinding of the participants was no longer possible. Both study personnel involved with the data collection and the study endocrinologist managing pharmacological treatment and safety were blinded to allocation. The clinical results used for pharmacological management and safety assessment were presented to the endocrinologist by the study nurse without disclosing participant allocation.

### Sample size and power considerations

We expected that an exercise intervention would increase the late-phase DI by 1.5 arbitrary units (a.u.) more than the control group, with a standard deviation of 1.5 a.u. of the change in the exercise and 1.0 a.u. in the control group^5^. For a contrast in a one-way analysis of variance (ANOVA) with four means (1.5, 1.0, 0.5, 0.0) and contrast coefficients (1, 0, 0, −1) using a two-sided significance level of 0.05, assuming an error standard deviation of 1.5 and a balanced design, a total sample size of 80 participants in the PP population (approximately 20 participants in each group) would yield statistical power of 87.7%.

### Statistical analysis

According to the protocol and the SAP, the analysis of the primary outcome was based on the as-observed population (missing data were not imputed in the primary analysis)^55,56^, as well as the PP population. The ‘Full Analysis Set’ for the ITT population included all randomized participants irrespective of their compliance with the interventions. The PP population criteria included (1) completion of the primary outcome assessment (all groups), (2) compliance with the diet protocol defined as being within ±30% of the prescribed energy intake (DCON, MED and HED), and (3) compliance with the exercise training protocol defined as completing ≥70% of the prescribed exercise volume across the intervention period (from weeks 2 to 16) (MED and HED). Missing data were assumed to be missing at random. Continuous data, including the primary, secondary and exploratory outcomes, were analyzed using constrained baseline longitudinal analysis via a linear mixed model^57^. As the baseline value is a part of the outcome vector, all participants with at least one measurement (baseline or follow-up) were included in the analyses^57^. The model included fixed effects for time (two levels), treatment (coded 0 for all groups at baseline and coded 0, 1, 2 or 3 at follow-up for CON, DCON, MED and HED, respectively) and sex (two levels), as well as the unique patient identifier as a random effect. The potentially biased PP population analysis was further adjusted for putative confounders: diabetes duration and baseline maximal oxygen consumption (ml O_2_/kg/min). Data are presented as the difference in the mean changes with 95% confidence intervals unless stated otherwise. The adequacy of the models was investigated via the predicted values and residuals. If the model assumptions were violated, the analyses were conducted using the log-transformed data and subsequently exponentiated for interpretation. Back-transformed data were expressed as the ratio of the geometric mean and interpreted as either percent change from baseline (within group) or difference in change between groups. A linear trend (interpreted as a linear dose–response relationship) was examined by treating each treatment category as a continuous variable in the main model and tested using a Wald test (*P* value reported). Linearity was inspected visually, and the *P* for trend was calculated only for the primary and secondary outcomes to the extent that the relationship was linear (that is, for late-phase DI and late-phase ISI). Sensitivity analyses were performed using multiple linear imputation procedures with the change in outcomes (post-pre values)^55^. The model included all covariates included in the main model, and beta coefficient and standard errors were based on 30 imputed data sets and adjusted for between-imputation variability^58^. Dichotomous outcomes were analyzed using logistic regression analyses. As sparsity of dichotomous outcomes (as expected for medications) invalidates the confidence intervals, exact logistic regression (exlogistic in Stata) was used when cases were <5^59,60^. A post hoc statistical mediation analysis was performed to examine the extent to which the observed treatment effect (in the intervention groups) on the primary and secondary outcomes was mediated by the change in body weight. An exploratory statistical mediation analysis was performed in R^61^ to examine the extent to which the observed treatment effect (in the intervention groups) on the primary and key secondary outcomes was mediated by the change in body weight. The lme4 package was used to construct the linear mixed models for the analysis^62^. This simple mediation analysis partitions the total causal effect into average direct effects (ADE) and average causal mediation effects (ACME; otherwise known as indirect effects). Bias-corrected and accelerated 95% confidence intervals were generated via nonparametric bootstrap analysis (2,000 resamples with replacement).

All non-hypothesis-based comparisons (that is, on the secondary and exploratory outcomes) are per definition considered exploratory and supportive of the interpretation of the primary outcome. If the global test of significance indicated between-group differences (*P* < 0.1)^63^, all outcomes (primary, secondary, exploratory and post hoc) on pairwise comparisons were explored. Although no corrections for multiplicity were performed, family-wise type 1 error rate on the primary outcome was retained by using a hierarchical analytic approach^63^. In accordance with our prespecified SAP, the six prespecified hierarchical hypotheses (based on a superiority assumption) were tested using the prespecified sequence: (1) CON versus HED, (2) CON versus MED, (3) CON versus DCON, (4) DCON versus HED, (5) DCON versus MED, (6) MED versus HED. If we failed to progress from any of the prior between-group comparisons (*P* > 0.05), the subsequent *P* values and confidence intervals were regarded as indicators of associations rather than causality. The statistical significance level (for superiority) was set at α < 0.05 (two-sided). The statistical analyses were performed using Stata/SE (StataCorp), version 17.1.

### Reporting summary

Further information on research design is available in the Nature Portfolio Reporting Summary linked to this article.

## Supplementary information

Supplementary Information CONSORT checklist, published protocol, overview of preplanned statistical analysis and approved study protocol.

Reporting Summary

Supplementary Tables Supplementary table overview and Supplementary Tables 1–16.

## Data Availability

Data are not available for download owing to privacy and ethical restrictions under the European Union’s General Data Protection Regulation (EU GDPR). Specific requests for access to the trial and individual-level and unique biological data included in this article may be sent to mathias.ried-larsen@regionh.dk. Based on the request, access may be provided to a named individual in agreement with the rules and regulations of the Danish Data Protection Agency and the National Committee on Health Research Ethics. Requests will be considered from the date of publication of this article.

## Code availability

The syntax files (Stata) are available upon request to mathias.ried-larsen@regionh.dk.
