# Supplementary material for: Effects of different doses of exercise and diet-induced weight loss on beta-cell function in type 2 diabetes (DOSE-EX): a randomized clinical trial
Source: Nat Metab. 2023 May 1;5(5):880–95. doi: 10.1038/s42255-023-00799-7 (PMC10229430; doi:10.1038/s42255-023-00799-7)
Supplement: Supplementary file 1 — CONSORT checklist, published protocol, overview of preplanned statistical analysis and approved study protocol. [file 42255_2023_799_MOESM1_ESM.pdf]

nature metabolism

Article https://doi.org/10.1038/s42255-023-00799-7

Effects of different doses of exercise and diet-induced weight loss on beta-cell function in type 2 diabetes (DOSE-EX): a randomized clinical trial

In the format provided by the authors and unedited

CONSORT 2010 checklist of information to include when reporting a randomised trial*

Section/Topic	Item No	Checklist item	Reported on page No
**Title and abstract**			
	1a	Identification as a randomised trial in the title	1
	1b	Structured summary of trial design, methods, results, and conclusions (for specific guidance see CONSORT for abstracts)	2
**Introduction**			
Background and objectives	2a	Scientific background and explanation of rationale	3, 4
	2b	Specific objectives or hypotheses	4
**Methods**			
Trial design	3a	Description of trial design (such as parallel, factorial) including allocation ratio	14, 19
	3b	Important changes to methods after trial commencement (such as eligibility criteria), with reasons	Statistical analyses plan
Participants	4a	Eligibility criteria for participants	14
	4b	Settings and locations where the data were collected	14
Interventions	5	The interventions for each group with sufficient details to allow replication, including how and when they were actually administered	14, 15, 16, and in protocol
Outcomes	6a	Completely defined pre-specified primary and secondary outcome measures, including how and when they were assessed	18, 19
	6b	Any changes to trial outcomes after the trial commenced, with reasons	N/A
Sample size	7a	How sample size was determined	20
	7b	When applicable, explanation of any interim analyses and stopping guidelines	N/A
Randomisation:			
Sequence generation	8a	Method used to generate the random allocation sequence	19, 20
	8b	Type of randomisation; details of any restriction (such as blocking and block size)	19
Allocation concealment mechanism	9	Mechanism used to implement the random allocation sequence (such as sequentially numbered containers), describing any steps taken to conceal the sequence until interventions were assigned	19, 20
Implementation	10	Who generated the random allocation sequence, who enrolled participants, and who assigned participants to	19

		interventions	
Blinding	11a	If done, who was blinded after assignment to interventions (for example, participants, care providers, those assessing outcomes) and how	19, 20
	11b	If relevant, description of the similarity of interventions	N/A
Statistical methods	12a	Statistical methods used to compare groups for primary and secondary outcomes	20, 21, 22
	12b	Methods for additional analyses, such as subgroup analyses and adjusted analyses	20, 21, 22
**Results**			
Participant flow (a diagram is strongly recommended)	13a	For each group, the numbers of participants who were randomly assigned, received intended treatment, and were analysed for the primary outcome	Figure 1
	13b	For each group, losses and exclusions after randomisation, together with reasons	4<br>Figure 1
Recruitment	14a	Dates defining the periods of recruitment and follow-up	14
	14b	Why the trial ended or was stopped	N/A
Baseline data	15	A table showing baseline demographic and clinical characteristics for each group	Table 1
Numbers analysed	16	For each group, number of participants (denominator) included in each analysis and whether the analysis was by original assigned groups	Table 1<br>Figure 1<br>And page 5
Outcomes and estimation	17a	For each primary and secondary outcome, results for each group, and the estimated effect size and its precision (such as 95% confidence interval)	Table 2<br>Table 4
	17b	For binary outcomes, presentation of both absolute and relative effect sizes is recommended	N/A
Ancillary analyses	18	Results of any other analyses performed, including subgroup analyses and adjusted analyses, distinguishing pre-specified from exploratory	Supp. Tables 10, 11, 14, 15, 16
Harms	19	All important harms or unintended effects in each group (for specific guidance see CONSORT for harms)	Table 3 and page 5,6
**Discussion**			
Limitations	20	Trial limitations, addressing sources of potential bias, imprecision, and, if relevant, multiplicity of analyses	11, 12, 13
Generalisability	21	Generalisability (external validity, applicability) of the trial findings	11, 12, 13
Interpretation	22	Interpretation consistent with results, balancing benefits and harms, and considering other relevant evidence	11, 12, 13
**Other information**			
Registration	23	Registration number and name of trial registry	14
Protocol	24	Where the full trial protocol can be accessed, if available	With

			submission
Funding	25	Sources of funding and other support (such as supply of drugs), role of funders	22, 23

*We strongly recommend reading this statement in conjunction with the CONSORT 2010 Explanation and Elaboration for important clarifications on all the items. If relevant, we also recommend reading CONSORT extensions for cluster randomised trials, non-inferiority and equivalence trials, non-pharmacological treatments, herbal interventions, and pragmatic trials. Additional extensions are forthcoming: for those and for up to date references relevant to this checklist, see www.consort-statement.org.

Lyngbaek et al. Trials (2021) 22:244
https://doi.org/10.1186/s13063-021-05207-7

Trials

STUDY PROTOCOL Open Access

The effects of different doses of exercise on pancreatic β-cell function in patients with newly diagnosed type 2 diabetes: study protocol for and rationale behind the "DOSE-EX" multi-arm parallel-group randomised clinical trial

Mark P. P. Lyngbaek[1†], Grit E. Legaard[1†], Sebastian L. Bennetsen[1], Camilla S. Feineis[1], Villads Rasmussen[1], Nana Moegelberg[1], Cecilie F. Brinkløv[1], Anette B. Nielsen[1], Katja S. Kofoed[1], Carsten A. Lauridsen[2,3], Caroline Ewertsen[2], Henrik E. Poulsen[4,5], Robin Christensen[6,7], Gerrit Van Hall[8], Kristian Karstoft[1,4], Thomas P. J. Solomon[9], Helga Ellingsgaard[1], Thomas P. Almdal[10,11], Bente K. Pedersen[1,5] and Mathias Ried-Larsen[1*]

Abstract

Background: Lifestyle intervention, i.e. diet and physical activity, forms the basis for care of type 2 diabetes (T2D). The current physical activity recommendation for T2D is aerobic training for 150 min/week of moderate to vigorous intensity, supplemented with resistance training 2–3 days/week, with no more than two consecutive days without physical activity. The rationale for the recommendations is based on studies showing a reduction in glycated haemoglobin (HbA1c). This reduction is supposed to be caused by increased insulin sensitivity in muscle and adipose tissue, whereas knowledge about effects on abnormalities in the liver and pancreas are scarce, with the majority of evidence stemming from in vitro and animal studies. The aim of this study is to investigate the role of the volume of exercise training as an adjunct to dietary therapy in order to improve the pancreatic β-cell function in T2D patients less than 7 years from diagnosis. The objective of this protocol for the DOSE-EX trial is to describe the scientific rationale in detail and to provide explicit information about study procedures and planned analyses.

(Continued on next page)

* Correspondence: mathias.ried-larsen@regionh.dk
†Mark P. P. Lyngbaek and Grit E. Legaard contributed equally to this work.
[1]Centre for Physical Activity Research, Copenhagen University Hospital - Rigshospitalet, Copenhagen, Denmark
Full list of author information is available at the end of the article

© The Author(s). 2021 **Open Access** This article is licensed under a Creative Commons Attribution 4.0 International License, which permits use, sharing, adaptation, distribution and reproduction in any medium or format, as long as you give appropriate credit to the original author(s) and the source, provide a link to the Creative Commons licence, and indicate if changes were made. The images or other third party material in this article are included in the article's Creative Commons licence, unless indicated otherwise in a credit line to the material. If material is not included in the article's Creative Commons licence and your intended use is not permitted by statutory regulation or exceeds the permitted use, you will need to obtain permission directly from the copyright holder. To view a copy of this licence, visit http://creativecommons.org/licenses/by/4.0/. The Creative Commons Public Domain Dedication waiver (http://creativecommons.org/publicdomain/zero/1.0/) applies to the data made available in this article, unless otherwise stated in a credit line to the data.

(Continued from previous page)
Methods/design: In a parallel-group, 4-arm assessor-blinded randomised clinical trial, 80 patients with T2D will be randomly allocated (1:1:1:1, stratified by sex) to 16 weeks in either of the following groups: (1) no intervention (CON), (2) dietary intervention (DCON), (3) dietary intervention and supervised moderate volume exercise (MED), or (4) dietary intervention and supervised high volume exercise (HED). Enrolment was initiated December 15th, 2018, and will continue until $N = 80$ or December 1st, 2021. Primary outcome is pancreatic beta-cell function assessed as change in late-phase disposition index (DI) from baseline to follow-up assessed by hyperglycaemic clamp. Secondary outcomes include measures of cardiometabolic risk factors and the effect on subsequent complications related to T2D. The study was approved by The Scientific Ethical Committee at the Capital Region of Denmark (H-18038298). Trial registration: The Effects of Different Doses of Exercise on Pancreatic β-cell Function in Patients With Newly Diagnosed Type 2 Diabetes (DOSE-EX), NCT03769883, registered 10 December 2018 https://clinicaltrials.gov/ct2/show/NCT03769883). Any modification to the protocol, study design, and changes in written participant information will be approved by The Scientific Ethical Committee at the Capital Region of Denmark before effectuation.

Discussion: The data from this study will add knowledge to which volume of exercise training in combination with a dietary intervention is needed to improve β-cell function in T2D. Secondarily, our results will elucidate mechanisms of physical activity mitigating the development of micro- and macrovascular complications correlated with T2D.

Keywords: Randomised controlled trial, Randomised clinical trial, Type 2 diabetes mellitus, Insulin resistance, β-cell function, Lifestyle intervention, Exercise, Inflammation, Oxidative stress

Introduction and rationale

Historically, type 2 diabetes (T2D) has been regarded as a treatable, yet chronic, condition. Glycated haemoglobin (HbA1c) is a diagnostic tool as well as an important indicator of long-term glycaemic control with the ability to reflect the average blood glucose level during the preceding 2 to 3 months [1]. Lifestyle intervention including physical activity forms the basis of clinical care of T2D. The current exercise recommendation for T2D is aerobic exercise for 150 min/week of moderate to vigorous intensity, supplemented with resistance training 2–3 days/week, with no more than two consecutive days without physical activity [2, 3]. The rationale for the exercise recommendations for T2D relies heavily on the consistent evidence supporting the efficacy of exercise in reducing HbA1c in T2D patients [2–6] . However, evidence suggests that targeting HbA1c, i.e. the mean reduction in glucose, as the main marker for glycaemic control is not sufficient in order to minimise micro- and macrovascular complications. This is underpinned by a randomised, clinical study of 10,251 T2D patients with established cardiovascular disease or cardiovascular risk factors. The study compared standard therapy with intensive therapy targeting HbA1c and found no significant reduction in nonfatal myocardial infarction, nonfatal stroke, or death from cardiovascular causes. However, a marginal benefit was observed for microvascular complications, i.e. microalbuminuria [7]. Variables such as plasma glucose fluctuations (i.e. glycaemic variability (GV)) [8–10] have been associated with poorer cardiovascular outcome as compared to sustained hyperglycaemia. Thus, GV might be taken into account when evaluating glucose control. A resent systematic review and meta-analysis found a dose-response relationship between physical activity and all-cause mortality in patients with T2D [11]. In essence, most clinical exercise interventions targeting T2D base their conclusions on HbA1c, but to fully uncover the efficacy of exercise on T2D, we aim to look at β-cell function and other markers of diabetes pathophysiology.

β-cell dysfunction

Although insulin resistance is the earliest detectable abnormality in T2D [12], dysfunction in the insulin secretory capacity is the major determinant of hyperglycaemia and onset of T2D [13]. By the time of T2D diagnosis, the insulin secretory capacity of the β-cell may be reduced by > 50% [14, 15]. This reduction was assessed by disposition index (DI) which is recognised as the most sensitive marker of β-cell function [15]. The detrimental effects of obesity on β-cell function are well-recognised [16, 17]. A genetic predisposition along with a chronic positive energy balance and pre-existing peripheral insulin resistance may lead to hepatic fat accumulation and subsequent hepatic insulin resistance [17, 18]. This leads to an increase in plasma glucose, which stimulates insulin secretion further enhancing the accumulation of liver fat and failure of insulin-induced suppression of hepatic gluconeogenesis.

The molecular mechanism leading to hepatic insulin resistance is suggested to be that fatty acids within hepatocytes may be oxidised for energy or are combined with glycerol to form mono-, di-, and then triacylglycerols (MAGs, DAGs, and TAGs). Excess intracellular diacylglycerol (DAG) activates protein kinase C epsilon type (PKCε) that inhibits the insulin receptor signalling

pathway, thus resulting in inhibition of glycogen synthesis and activation of gluconeogenesis [16]. The chronic excess energy availability, hyperinsulinemia, and hepatic insulin resistance promote hepatic de novo *lipogenesis* that increases delivery of lipids from the liver to the circulation, tissues, and organs [19], including the pancreas where they will accumulate [17]. Due to peripheral (muscle and adipose tissue) and central (hepatic) insulin resistance, increased levels of portal insulin develop and may further stimulate hepatic de novo *lipogenesis*. This augments the storage of lipids in β-cells [17]. Intracellular lipid accumulation in the β-cells eventually leads to secretory dysfunction. This self-reinforcing cycle between the liver and the pancreas, known as the *twin cycle hypothesis* [20], may compromise β-cell insulin secretion. Consequently, the β-cell can no longer compensate for the peripheral insulin resistance in response to ingested glucose and promotes the onset of hyperglycaemia.

According to the β-cell centric hypothesis proposed by Schwartz et al. [21], the β-cell dysfunction is the sole common denominator for diabetes aetiology. The β-cell dysfunction and subsequent hyperglycaemia is the culprit for the generation of excess reactive oxygen species (ROS) and for the subsequent oxidative stress (OS). This OS induced by hyperglycaemia is suggested to be the unifying complication impetus in T2D. Thus, in supplement to HbA1c, it may be beneficial to focus on mechanisms alleviating β-cell dysfunction and subsequent vascular complications, when evaluating the significance of exercise in the clinical care of prevalent T2D [5, 6, 22, 23].

Influence of the toxic diabetic milieu on the β-cell

The mechanism behind β-cell dysfunction may include an abundance of excess energy, consisting of fatty acids and glucose, escalating the production of ROS and causing inflammation [18, 21, 24, 25]. Chronic hyperglycaemia (i.e. glucotoxicity) has been shown to induce β-cell apoptosis by increasing proapoptotic gene expression while antiapoptotic gene expression remains unaffected [18]. Also, glucotoxicity increases malonyl-CoA levels, which leads to inhibition of carnitine palmitoyl transferase-1 and a subsequent decrease in fatty acid oxidation. For β-cells to secrete insulin in response to glucose, adenosine triphosphate (ATP) production must take place, but excess of fatty acids and TAGs are thought to inhibit this process. Under physiological conditions, when glucose enters the β-cell through glucose transporter 2, glucose undergoes glycolysis and the tricarboxylic acid cycle to generate ATP. However, increased fatty acid availability (i.e. lipotoxicity) inhibits both pyruvate cycling and pyruvate dehydrogenase activity, inhibiting the ATP synthesis and thereby diminishing insulin secretion [17]. Moreover, endoplasmic reticulum stress caused by gluco- and lipotoxicity may cause a depletion of Ca^{2+} stores and further prevent the release of insulin [26, 27]. Lipotoxicity activates the unfolded protein response in endoplasmic reticulum (ER) and increases both OS and transcriptional factors, e.g. nuclear factor κappaB (NFκB) [18]. In addition, lipotoxicity and increased glucose concentration inhibit β-cell proliferation [17]. Thus, the pancreatic β-cell in the diabetes milieu is subject to several detrimental incidents such as OS, mitochondrial dysfunction, ER stress, and islet inflammation and epigenetic modification [18, 21, 28].

Systemic inflammatory signals as well as islet inflammation may also cause oxidative stress and activation of infiltrated macrophages [26]. It has been suggested that prolonged exposure of pancreatic islet to chronic glucolipotoxicity and ROS might trigger the intracellular production of the inflammatory cytokines specifically IL-1β and TNF-α and trigger signal transduction pathways, such as NFκB resulting in ER stress, to induce expression of proinflammatory genes, mitochondrial dysfunction, secretory dysfunction, and apoptosis. Additionally, this may also be triggered by proinflammatory signals from other organs, e.g. adipose tissue [18]. Indeed, 13 weeks of pharmacological inhibition of IL1-β in T2D patients by subcutaneous injections of the IL-1 receptor antagonist (IL-1RA) did increase β-cell secretory function [29]. This supports the hypothesis that inflammation plays an important role in the aetiology of T2D and may be causally related to β-cell dysfunction in T2D.

The aetiology, pathophysiology, and treatment of T2D are undeniably multifactorial and the understanding of T2D is increasing rapidly, but reducing obesity remains essential to improve β-cell function. However, a residue β-cell capacity appears to be essential for remission emphasising the need for lifestyle intervention early in the clinical management [20]. While exercise is less recognised as an efficient therapy for weight loss, dietary therapy is [30]. With the recent advantages in the role of very low-calorie diets on β-cell function [20, 31], it is important to study the role of exercise therapy in combination with dietary-induced weight loss to fully understand the implications for patient care. An outline of the current understanding of the effects of exercise training on the β-cell and the mechanisms leading to improved β-cell function is discussed in the following section.

Exercise and inflammatory factors in β-cell dysfunction

It has previously been suggested that the anti-inflammatory effects of exercise may partly be linked to improved β-cell function in T2D. This may be due to mechanisms that are different from diet-induced weight loss [32]. Such an example is IL-6 secreted from contracting skeletal muscle, inducing an increase in the production of IL-1RA and IL-10, thus exerting a systemic anti-inflammatory effect. Moreover, IL-6 regulates visceral fat lipolysis [33], which potentially reduces systemic

inflammation, while also contributing to non-insulin-dependent glucose uptake in skeletal muscle during acute exercise [34]. Also, IL-6 increases the incretin glucagon-like peptide-1 (GLP-1) that may protect β-cell from apoptosis, promote β-cell growth, and delay gastric emptying [35–37], thereby indirectly decreasing post-prandial insulin demand. Hence, exercise-induced anti-inflammatory effects and myokine secretion could indirectly contribute to β-cell rest.

It is evident that hyperglycaemia induces overproduction and expression of advanced glycation end-products (AGEs) and the receptor for AGE (RAGE). Activation of the AGE-RAGE-axis has been associated with the development of diabetic complications [38, 39]. A common feature and diagnostic marker for T2D is postprandial hyperglycaemia, and a large portion of patients with T2D express a high degree of glycaemic variability (GV) in response to a meal. GV has been suggested to be more strongly associated with OS and vascular endothelial dysfunction than sustained chronic hyperglycaemia [8–10]. A proposed mechanism is that an acute increase in blood glucose (e.g. postprandial hyperglycaemia) activates the AGE-RAGE-axis via reactive oxygen species and signal transduction pathways (i.e. NFκB pathway), producing OS and inflammation [38]. The increased production of ROS will further enhance the production of AGEs, generating a feed-forward cycle. Endogenous soluble forms of RAGE (esRAGE and sRAGE) are found in the circulation and are suggested to modulate the AGE-RAGE response, acting as decoy ligands [38]. Soluble RAGE might increase in response to elevated AGE-RAGE levels and act as a marker for cardiovascular disease. However, it has been shown that exercise training increases sRAGE while markers of OS decrease [40]. Inhibition of the AGE-RAGE axis thus seems imperative in reducing the risk of vascular complications and might also mitigate the production of systemic OS, offering an indirect manner to decrease the inflammatory exposure on the β-cell. GV may be reduced by exercise in patient with T2D [41–43]; still, the effect of exercise on the AGE-RAGE-axis is not fully understood, nor is the role of GV in this context.

β-cell function in response to exercise

In a recent study from Heiskanen et al., it was observed that only 14 weeks of exercise decreased pancreatic ectopic lipid accumulation and improved β-cell function in both participants with and without T2D [44].

Evidence from human, animal, and in vitro models, as shown in a recent review, supports that exercise may increase β-cell mass (i.e. β-cell proliferation, β-cell apoptosis, and β-cell viability) and improve β-cell function (i.e. glucose sensing, insulin secretion, and insulin content) [45, 46]. However, human studies investigating exercise duration, intensity, frequency, volume, and dose dependency are few, but important for understanding the link between exercise and β-cell health [45].

Hepatic response to exercise in relation to β-cell dysfunction

Exercise may, independently of even a minimal weight loss, relieve hepatic insulin resistance [47] and decrease hepatic fat content and de novo *lipogenesis* [48–50]. In addition, exercise improves liver fatty acid metabolism and might prevent mitochondrial and hepatocellular damage [51]. Exercise is evident as a therapeutic strategy to improve fatty liver disease and, given the crosstalk between liver and pancreas, might further potentiate the benefits of a diet-induced weight loss intervention on the β-cell.

Skeletal muscle and glucolipotoxicity in relation to exercise and β-cell dysfunction

When plasma glucose reaches the insulin receptor, it promotes the docking and fusion of glucose transporter type (GLUT) 4, containing vesicles to the plasma membrane. Peripheral insulin resistance has been suggested to be partly mediated by an inability to oxidise excess lipid delivery/storage or convert DAG to TAG [16]. It is well established that exercise improves insulin sensitivity in peripheral tissue [34, 52], which potentially induces pancreatic β-cell rest. During an acute bout of exercise training, the exercising muscle increases glucose uptake insulin-independently via multiple mechanisms [53]. Two to 72 h post exercise, there is an increase in GLUT4 translocation to the cell membrane, increasing insulin sensitivity [54]. Also, an acute bout of exercise has been shown to induce increased diacylglycerol acyl transferase 1 expression in skeletal muscle, promoting the conversion from DAG to TAG [16]. Furthermore, an increase in muscle mass appears to be beneficial, and the combination of resistance training and aerobic training has been shown to be superior to either of the two [54]. In skeletal muscle, chronic exercise increases GLUT4 concentration and enhances capillarisation, mitochondrial function, and content, all major factors for insulin sensitivity [53]. In summary, exercise-induced effects on peripheral insulin sensitivity may therefore act as a “drain” for glucose disposal, indirectly reducing gluco- and lipotoxic exposure to the β-cell.

Muscular changes in relation to exercise and T2D

Only little attention has been given to the negative impact of T2D on skeletal muscle. T2D, like other chronic diseases coinciding with low-grade inflammation, may lead to loss of muscle mass, resulting in reduced physical capacity and strength, a condition called diabetic myopathy [55]. Loss of muscle mass and strength is strongly

associated with poor mobility and physical function [56], which subsequently leads to sarcopenia, frailty, and loss of autonomy [57]. Patients with T2D have been observed to have a 2–3-fold higher risk of sarcopenia [58]. Muscle progenitor cells (here referring to satellite cells (SC)) are essential for maintenance of muscle homeostasis and regeneration [59]. SC and endothelial cells are suggested to interact in the capillarisation of skeletal muscle [60, 61]. The microvasculature is essential for the delivery of oxygen, cytokines, growth factors, waste removal, and physical capacity of the muscle. An increased capillarisation is associated with increased SC function, and muscle perfusion may be a critical factor in repair and recovery of damaged muscles [62]. It has been demonstrated in vitro that endothelial cells in a high glucose environment secrete factors that dysregulate SC growth and differentiation [63]. In addition, autophagy is imperative for muscular regenerative capacity and function [64]. Autophagy is shown to be dysregulated in T2D, affecting e.g. myogenesis and negatively affecting the regenerative capacity of muscle [65]. However, autophagy markers are increased following exercise in humans [66] and this has been shown in both acute and chronic exposure to exercise [67]. Macrophages play an essential role in regulating muscle stem cells [68], and intramuscular inflammation has been associated with T2D. Muscle expression of macrophage genes has been linked to hyperglycaemia, whereas anti-inflammatory markers have been associated with low glycaemia, exercise, and high glucose disposal rate [69]. There is a reason to speculate that an exercise training intervention combined with diet-induced weight loss may mitigate the detrimental effects of T2D on skeletal muscle, thus maintaining muscular function and further contributing to β-cell rest. The role of exercise on autophagy in human diabetic muscle cells has to our knowledge not yet been investigated.

Investigating the dose of exercise training in addition to diet-induced weight loss

There is evidence that higher levels of physical activity are associated with a lower mortality risk in patients with T2D [70]. However, only a few studies have focused on the effects of exercise on pancreatic β-cell function in T2D and discrepancies regarding the effect exist [46, 71–74]. The discrepancies may relate to the assessment of β-cell function [75], failure to correct for the change in peripheral insulin sensitivity, concomitant pharmacological therapy, and the pre-trial insulin secretory capacity. Moreover, exercise intensity, volume, and modality may play an essential role in the reduction of HbA1c [4, 6, 76–78]. Thus, current evidence suggests that physical activity may *directly* improve β-cell mass and β-cell function [45], and may also *indirectly* improve β-cell function and mass by inducing β-cell rest via reductions in systemic inflammation and metabolic stress (i.e. gluco- and lipotoxicity). However, evidence is limited from human studies investigating the relationship of exercise volume, intensity, frequency, and dose dependency on β-cell function [45]. As a consequence, knowledge about the exercise training dose needed to reduce micro- and macrovascular complications in T2D is almost non-existing [4, 5, 7, 79–84]. As most clinical exercise interventions in T2D base their conclusions on HbA1c, the significance of exercise training in the clinical care of prevalent T2D is challenged [5, 6, 22, 23] and investigating β-cell function with different volumes of exercise in addition to a diet-induced weight loss is of clinical relevance. We propose that combining a moderate diet-induced weight loss with exercise training may dose-dependently improve pancreatic β-cell function.

Study objectives and hypotheses

Objectives

Primary objective To investigate the dose dependency of exercise training on pancreatic β-cell function after 16 weeks in patients with short standing T2D.

Secondary objectives To investigate the dosing effect of exercise training on mechanisms mitigating pancreatic β-cell dysfunction and markers of cardiovascular complications.

Research hypotheses

Primary The effect of exercise training on pancreatic β-cell function (assessed as late-phase disposition index) increases with increasing volumes of exercise in combination with a diet across a 16-week intervention in patients with T2D of short duration. It is expected that both moderate volume and high volumes of exercise in combination with a dietary intervention are superior to the control intervention in improving pancreatic β-cell function.

Secondary Exercise training decreases low-grade inflammation in a dose-dependent manner. The intervention-induced reductions in low-grade inflammation are associated with intervention-induced reductions in glycaemic variability, ROS, and alterations in the AGE/RAGE axis. It is expected that both moderate volume and high volumes of exercise training in combination with a dietary intervention are superior to the control or dietary intervention alone in improving low-grade inflammation, ROS, glycaemic variability, and alterations in the AGE/RAGE axis.

Methods

Study design and setting

The study is designed as a parallel-group, 4-arm assessor-blinded, randomised, clinical trial where the primary outcome is β-cell function as measured by a 3-stage hyperglycaemic clamp before and after 16 weeks of intervention. Participants will be randomly allocated (1,1:1:1, stratified by sex) to four groups; (1) no intervention, (2) dietary intervention, (3) dietary intervention + moderate volume exercise, (4) dietary intervention + high volume exercise. The flow of participants is described in Fig. 1. Exercise training in combination with dietary recommendations is a cornerstone in the treatment of T2D, but the isolated effect in addition to dietary changes is poorly understood. Furthermore, knowledge is scarce on volume of exercise training needed to induce clinically significant effects. Thus, as the trial is designed to investigate the additive dose-response effects of exercise in conjunction with a dietary intervention, a multi-arm design is employed.

Participants will be recruited within the Capital Region of Denmark. The study is registered at www.clinicaltrials.gov (NCT03769883) and approved by the Scientific Ethical Committee of the Capital Region of Denmark (approval number H-18038298) prior to commencement of any study procedures. Primary place of study execution and data collection will be Centre for Physical Activity Research (CFAS), Rigshospitalet, section 7641, Tagensvej 20, DK-2200 Copenhagen (visiting address); Blegdamsvej 9, DK-2100 Copenhagen (postal address), Telephone: (+ 45) 3545 7641. Magnetic resonance imagining and magnetic resonance spectroscopy will be collected at Radiologisk Klinik X, section 3032, Rigshospitalet. All data will be collected and analysed in Denmark.

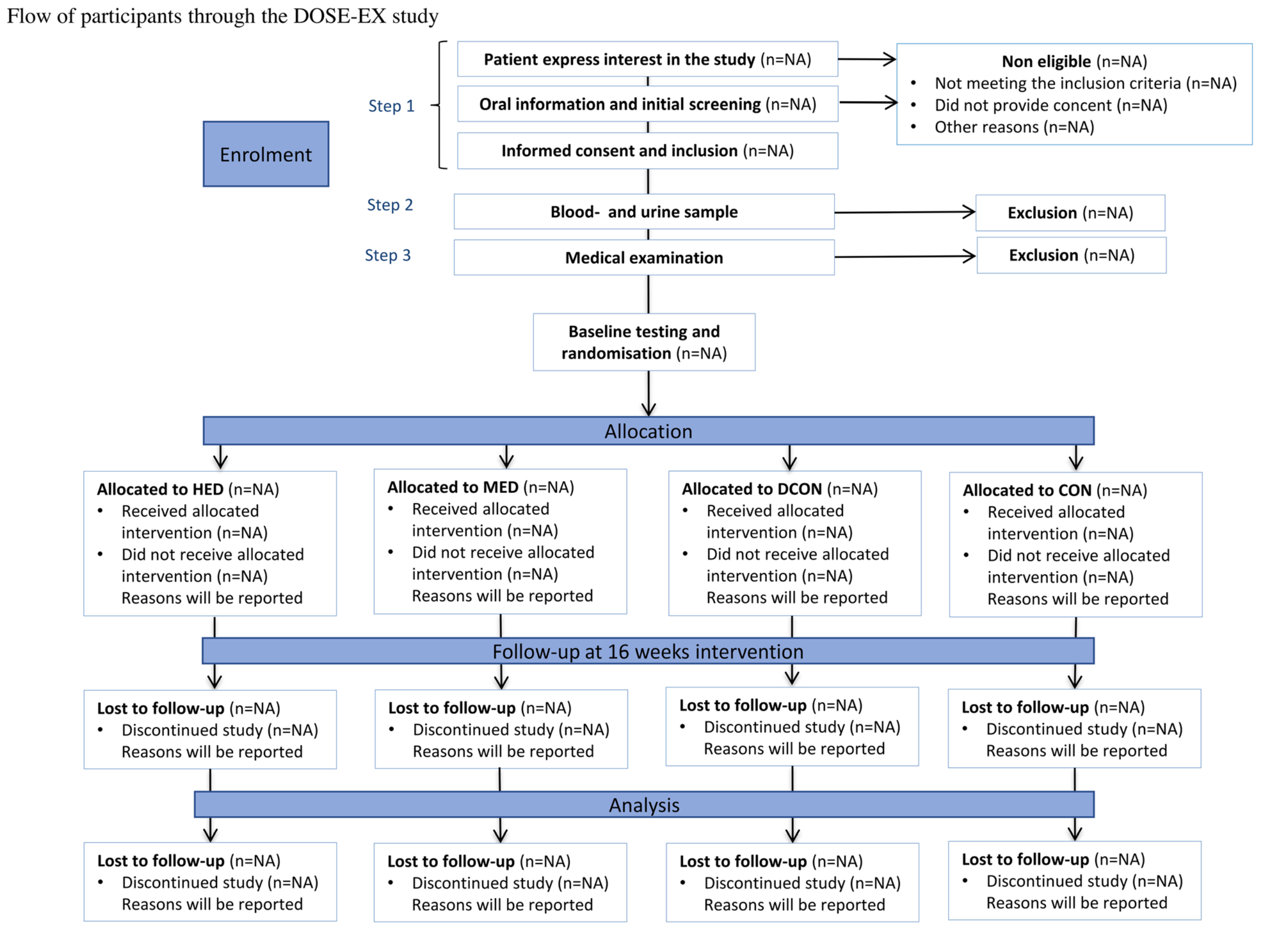

Fig. 1 Flow of participants through the DOSE-EX study. HED, high exercise dose; MED, moderate exercise dose; DCON, dietary intervention; CON, control group. Steps 1, 2, and 3 refer to the recruitment procedure. Step 1 being the initial contact with possible participants, screening by phone and obtaining informed consent if inclusion criteria are met. In step 2, eligibility is assessed based on blood and urine samples and finally step 3 constitutes a medical examination (see text for details). If no exclusion criteria are identified the participant proceeds to baseline testing and randomisation. Participants are randomly allocated to HED, MED, DCON, or CON in a ratio of 1:1:1:1 stratified by sex

Participants and eligibility

Poor glycaemic control and poor β-cell function prior to training predict less benefit from exercise training [85, 86]. Indeed, Dela et al. showed that T2D patients with remaining insulin secretory function improved their insulin secretory capacity with exercise, whereas participants with low secretory capacity prior to the intervention did not [46]. This is in line with observations from other lifestyle interventions in T2D [87, 88]. Moreover, to avoid any risk of drug-induced signs of hypoglycaemia or hypotension, previous trials with an expected decrease in body weight in other populations of T2D patients have adjusted the concomitant glucose- and blood pressure (BP)-lowering medications according to symptomatology and/or standard care guidelines without any adverse effects [88].

Thus, to use lifestyle as a first-line monotherapy, it is sensible to focus on T2D patients with remaining β-cell function prior to the intervention, and so, patients with a diabetes duration of < 7 years is the primary target population. Eligibility criteria are described in Table 1.

Interventions

The general intervention is based on a previous trial and adapted to the aim of this study [89, 90]. The active interventions will consist of two main components; (1) structured supervised exercise and/or (2) a dietary intervention aiming at a weight loss. Whereas there will be no difference in the dietary intervention between the lifestyle groups, the volume of physical activity and structured exercise vary according to the frequencies of the structured exercise sessions. The anticipated flow of participants is described in Fig. 1 and an overview of the intervention is depicted in Fig. 2 while a detailed description of the weekly training sessions is found in Fig. 3.

1) Control group (CON): no intervention. Participants will be encouraged to maintain habitual exercise and dietary habits throughout the study.
2) Dietary control (DCON): dietary intervention. Participants will be encouraged to maintain habitual exercise habits throughout the study.
3) Moderate exercise dose (MED): two aerobic training sessions per week of 45–60 min duration and one session per week with combined aerobic (30–35 min) and resistance (30 min) training, and a dietary intervention.
4) High exercise dose (HED): four aerobic training sessions per week of 45–60 min duration and two sessions per week with combined aerobic (30–35 min) and resistance (30 min) training, and a dietary intervention.

Dietary intervention

Overall, the dietary intervention aims for weight loss and improved glycaemic control, while mimicking the general dietary recommendations for persons with T2D. The macro-nutrient distributions are in line with the current guidelines from the national Diabetes Association and Canadian guidelines, where individualisation in macro-nutrient distribution is within the range of 45–60E% from carbohydrate, 15–20E% from protein, and 20–35E% from fat [91]. The diet will include food items with a low glycaemic index and load, as these are associated with improved glycaemic control in contrast to food items with a high glycaemic index and high glycaemic load [92–95]. Saturated fat intake is related to cardiovascular disease risk [96]; thus, the dietary plan will aim at reducing saturated fat intake < 7E% [97] in accordance to national guidelines. Both prevention and a successful management of type 2 diabetes are highly related to diets rich in whole grains, fruit, vegetables, nuts and legumes, and lower on refined grains, red or processed meat, and sugar-sweetened beverages [96]. Therefore, a range of macro-nutrient composition will allow for individual preferences to be included in the dietary plan.

Dietary procedure

A registered clinical dietician will prepare the individual meal plan with proposed recipes based on individual counselling (3 sessions during the intervention). The implementation and potential adjustments will be performed continuously based on self-reported, 3-day, dietary records (for details, please refer to patient-reported outcomes) that are discussed at the individual sessions. The meal plan will cover three main meals and three snack meals per day. The content of the recipes may be adapted to individual participant preferences. Energy requirement will be based on the age-adjusted Oxford equations as described by Henry 2005 [98], aiming at a weight loss, Table 2. The participants' body weight will be used for calculation of the energy requirement if the body mass index is < 30 kg/m^2. If the BMI is > 30 kg/m^2, the body weight in the equation will be adjusted to corresponding to a BMI = 25 kg/m^2. In order to reduce the risk of mild hypoglycaemia (e.g. trembling, heart racing, feeling uncomfortable, nausea) and hunger on days with training sessions, 100–200 kcal snack meal will be added to the energy intake just before and after training (MED and HED). Furthermore, a main meal 2–3 h before a training session will be advised. In case of subjective signs of low blood glucose, the participants will be instructed to either eat one piece of fruit or to drink a glass of juice in combination with a piece of rye or crisp bread. To facilitate adherence, the dietician will contact the participants by text, phone, or e-mail (depending on their preferences) in between the individual

Table 1 Eligibility of study participants

Inclusion criteria	Exclusion criteria
Men and women aged 18–80 years	HbA1c: ≥ 75 mmol/mol with no glucose-lowering medications
Diagnosed with diabetes type 2 and/or HbA1c ≥ 48 mmol/mol if no treatment with anti-diabetic medication and/or use of anti-diabetic medication	HbA1c: ≥ 64 mmol/mol with mono glucose-lowering therapy (if compliant with the prescription)
Caucasian	HbA1c: ≥ 57 mmol/mol with ≥ dual glucose-lowering therapy (if compliant with the prescription)
No diagnose of type 1 diabetes, MODY-diabetes, type 1½ diabetes or LADA-diabetes	eGFR < 60 mL/min
T2D duration < 7 years	Macroalbuminuria at pre-screening
No treatment with insulin	Clinical or biochemical signs of hypothyroid disease
No use of sulphonylurea-based drugs	Biochemical sign of other major diseases
Body Mass Index (BMI) > 27 kg/m^2 and < 40 kg/m^2	Presence of circulating glutamatdecarboxylase anti body (GAD) 65
No known or signs of intermediate or severe microvascular complications to diabetes (retino-, neuro- or nephropathy)	Objective findings that contraindicates participation in intensive exercise (Pedersen and Saltin 2006)
No known cancer	Anamnestic findings that contraindicates participation in the study (Pedersen and Saltin 2006)
No lung disease, other than asthma that can be managed with beta2-agonists and does not exhibit seasonal variation.	Unable to allocate the needed time to fulfil the intervention
No known cardiovascular disease	Language barrier, mental incapacity, unwillingness or inability to understand and be able to complete the interventions
No known hyperthyroid disease	HbA1c: ≥ 75 mmol/mol with no glucose-lowering medications
No changes in hypothyroid disease treatment within the last 3 months prior to enrolment	HbA1c: ≥ 64 mmol/mol with mono glucose-lowering therapy (if compliant with the prescription)
No known liver disease—defined as ALAT or ASAT elevated three times above upper limit.	HbA1c: ≥ 57 mmol/mol with ≥ dual glucose-lowering therapy (if compliant with the prescription)
No known autoimmune disease	eGFR < 60 mL/min
No psoriasis disease requiring systemic treatment or cutaneous elements bigger than a total area of 25 cm2	Macroalbuminuria at pre-screening
No other endocrine disorder causing obesity	Clinical or biochemical signs of hypothyroid disease
No current treatment with anti-obesity medication	Biochemical sign of other major diseases
No current treatment with anti-inflammatory medication	Presence of circulating glutamatdecarboxylase anti body (GAD) 65
No weight loss of > 5 kg within the last 6 months	Objective findings that contraindicates participation in intensive exercise (Pedersen and Saltin 2006)
No changes in symptoms or anti-depressive medication 3 months prior to enrolment.	Anamnestic findings that contraindicates participation in the study (Pedersen and Saltin 2006)
No diagnose of psychiatric disorder or treatment with anti-psychotic medication	Unable to allocate the needed time to fulfil the intervention
No history of suicidal behaviour or ideations within the last 3 months **prior** enrolment	Language barrier, mental incapacity, unwillingness or inability to understand and be able to complete the interventions
No previous surgical treatment for obesity (excluding liposuction > 1 year prior to enrolment)	
Not pregnant/considering pregnancy	
No functional impairments that prevents the performance of intensive exercise	
Accept of medical regulation by the study endocrinologist	
Inactivity, defined as < 1,5 h of structured physical activity per week at moderate intensity or cycling < 30 min/5 km per day at moderate intensity (moderate intensity = out of breath but able to speak)	
No participation in other research intervention studies	

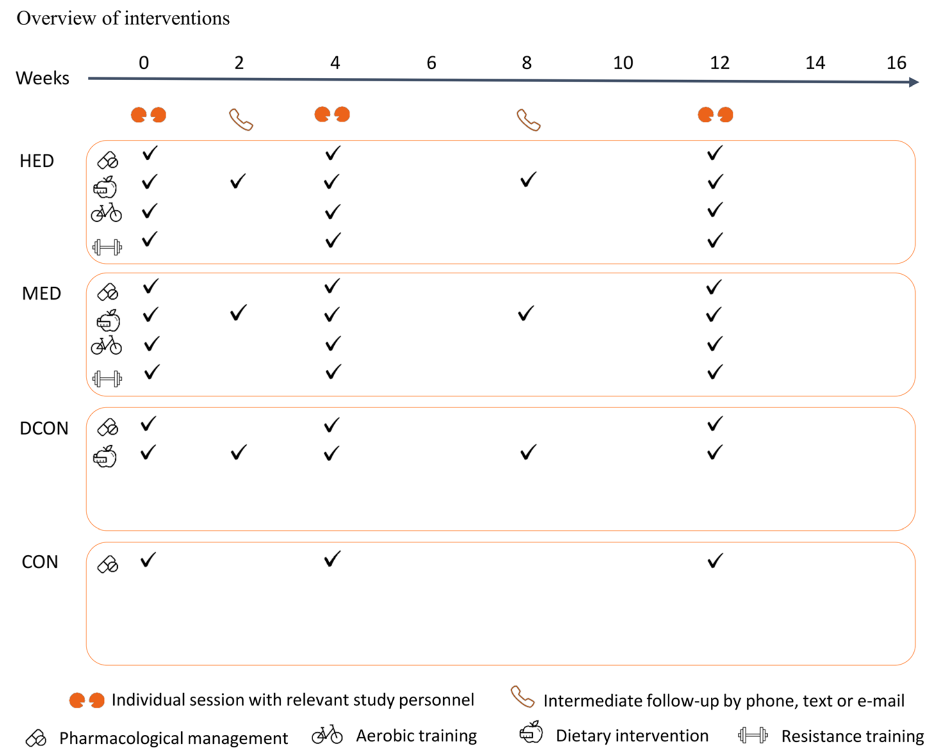

Fig. 2 Overview of interventions. HED, high exercise dose; MED, moderate exercise dose; DCON, dietary intervention; CON, control group. The intervention runs over a full 16 weeks period. During the 16 weeks, adherence and individual adjustment in the different parts of the intervention (exercise, diet, and pharmacological management) will be evaluated by relevant study personnel. Dietary consulting is provided by the dietician. The coordinating exercise trainer handles consults regarding the exercise intervention and pharmacological management is provided by the study nurse in close collaboration with the blinded endocrinologist. Prior to the dietary consults, each participant will be asked to complete a 3-day dietary record to frame the conversation. Pharmacological adjustments will be based on resent results from blood samples and blood pressure measured at home

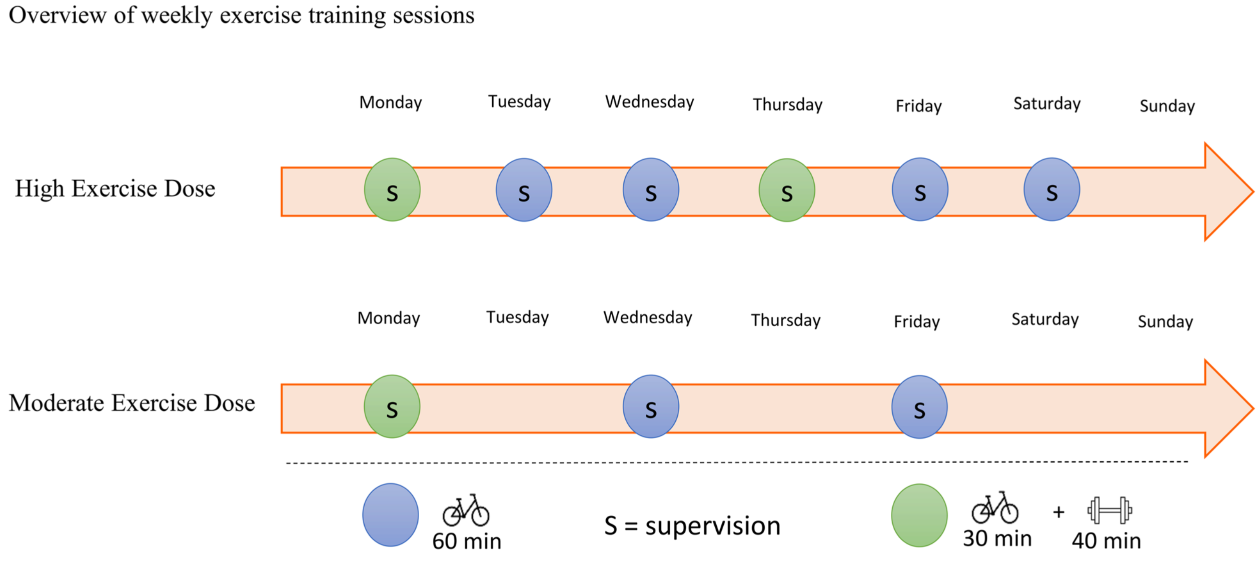

Fig. 3 Overview of weekly exercise sessions. HED, high exercise dose; MED, moderate exercise dose; DCON, dietary intervention; CON, control group

Table 2 Daily energy requirement

Calculated BMR	Energy requirement per day
< 1200–1249 kcal	1200 kcal
1250–1349 kcal	1300 kcal
1350–1449 kcal	1400 kcal
1450–1549 kcal	1500 kcal
1550–1649 kcal	1600 kcal
1650–1749 kcal	1700 kcal
1750–1849 kcal	1800 kcal
1850–1949 kcal	1900 kcal
1950–2049 kcal	2000 kcal
2050–2149 kcal	2100 kcal
2150–2249 kcal	2200 kcal

Basic metabolic rate (BMR) is calculated from the age-adjusted Oxford equation as described by Henry 2005 (NNR, 2012). Participants current body weight is used if body mass index (BMI) < 30 kg/m^2 and in case of BMI > 30, the body weight in the equation is adjusted to equal BMI = 25 kg/m^2. Daily energy requirement is based on the level of kilocalories closest to the calculated BMR

In addition, participants in the high exercise dose (HED) and moderate exercise dose (MED) groups receive 200 kcal for restitution on days with training sessions

sessions. Furthermore, participants will be allowed to contact the dietician by e-mail once a week if they have issues regarding implementation of or concerns about the meal plan.

Rescue procedure for the dietary intervention

If a participant exceeds ± 30% of the prescribed energy intake as assessed by the dietary records, the procedures below will be initiated. Moreover, if the participant expresses concerns about satiety, food preferences, or food preparation techniques, the procedures, described below, will be initiated. A 1-week pause will be allowed for, e.g. vacation or illness, where the participants will receive dietary guidance that will be feasible to follow. Moreover, the participant will be asked to complete at dietary record during the pause, in order to adjust the following dietary programme to reach the pre-specified energy intake.

The procedures for the dietary intervention include;

(1) An interview regarding compliance to the meal plan will be performed, and the participant will be provided with specific guidelines to practical changes in the plan by the clinical dietician. This is done to augment adherence to food items, increase satiety, or exchange some food items to match preferences.
(2) If action 1 proves insufficient, the energy intake will be increased in steps of 100 kcal/day until the level of satiety is acceptable to the participant.

Increased structured exercise

The training protocol will be adapted based on a previous study where the T2D participants were prescribed 4 weekly sessions of 60 min aerobic training alone and 2 combined aerobic and resistance training sessions (averaging 360–420 min of exercise per week) [89]. Although mean adherence was high (82% of the planned sessions were completed), variation was high and imperfect [90]. Since the variation was high in adherence in the previous study, a lower dose of exercise training may be expected in a subset of participant in the Dose-Ex study. Because of this expectation, and because of previous analyses suggesting that there may be an inverse dose-response relationship between reductions in HbA1c and aerobic training volume [4, 6], a group of moderate dose exercise training is formed (i.e. MED group). Furthermore, high intensity during both aerobic and resistance training has been shown to have a superior effect on reduction in HbA1c compared to moderate intensity [99–101]. Thus, high-intensity training is implemented in the exercise training protocol. As the effect of exercise training on glycaemic control is more closely related to the number of training sessions [6], we will reduce the number of sessions by 50% to three sessions/week in the MED group and maintain the original session frequency in the HED group (six sessions/week).

Exercise procedures

In the first 2 weeks of the intervention, a familiarisation to the specific exercises will be prioritised to facilitate the training quality (i.e. to meet the prescribed training intensity) in the remaining part of the intervention. During this period, the participants will be thoroughly introduced to a heart rate monitor, training programmes, and the concept of *repetitions in reserve* (RIR) [102, 103]. The target aerobic training intensity span will be 60–100% of maximal heart rate (HR_{max}), which is in line with current guidelines [2, 89, 101]. A correction of HR_{max} will be performed if a higher measurement is found during the aerobic training [104]. Throughout the intervention, the time spend exercising in intensity zone 80–100% of HR_{max} will increase and consequently the time spend exercising in intensity zone 60–79% of HR_{max} will be reduced (Fig. 4). The aerobic training programmes are found in Additional file 1 and will be programmed on the Polar HR watch (Polar V800, Polar, Holte, DK). The Polar HR watch will show the participant when it is time to modify the intensity in order to reach the target intensity zone during each training session. Replacement of the aerobic training programmes throughout the intervention will permit progression in minutes spend in intensity zone 80–100% of HR_{max}. Aerobic training programme 1, 2, and 3 will be used during the initial 2 weeks. The volume and intensity of

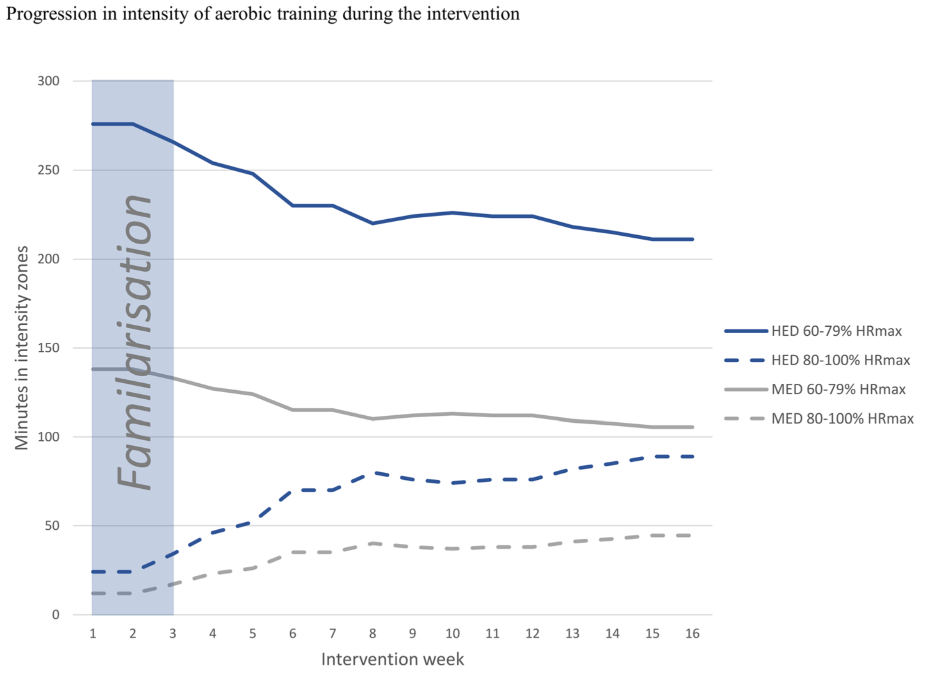

Fig. 4 Progression in intensity of aerobic training during the intervention. During the 16 weeks, time spend in intensity zone 80–100% of maximum heart rate (HR_{max}) increases gradually as time spend in intensity zone 60–79% of HR_{max} decreases. Total aerobic training volume for high exercise dose and moderate exercise dose are 300 and 150 min per intervention week, respectively. Volume remains unchanged throughout the intervention period

the resistance training will also be in line with current guidelines, e.g. 3–6 bouts of 8–12 repetitions per muscle group with intensities varying between 9 and 15 repetition maximum (1–3 RIR) [2]. The resistance training periodisation is presented in Fig. 5. Specific supersets with resistance training exercises can also be found in Additional file 1. If a participant can complete 3 repetitions more than prescribed (12, 10 or 8 repetitions), the load (i.e. resistance) will systematically increase in the next resistance training session (Table 3). The resistance

Resistance training periodisation

	Resistance training periodisation															
**Period**	**Fam**		**Block 1**				**Block 2**						**Block 3**			
**Week no.**	**1**	**2**	**3**	**4**	**5**	**6**	**7**	**8**	**9**	**10**	**11**	**12**	**13**	**14**	**15**	**16**
**Set**	3	3	3	3	3	3	3	3	3	3	3	3	3	3	3	3
**Repetitions**	12	12	12	12	12	12	10	10	10	10	10	10	8	8	8	8
**Rest (s)**	30	30	30	30	30	30	30	30	30	30	30	30	30	30	30	30
**RIR**	2-3	2-3	1-2	1-2	1-2	1-2	1-2	1-2	1-2	1-2	1-2	1-2	1-2	1-2	1-2	1-2
**RIR test**			x					x			x			x		

Fig. 5 Resistance training periodisation. Fam, familiarisation period; RIR, repetitions in reserve. The initial 2 weeks of the intervention constitutes a familiarisation period with thorough instruction to each exercise and reduced intensity. The remaining intervention period is divided into blocks ensuring progression towards less repetitions with higher loads. The resistance training frequency for high exercise dose (HED) and moderate exercise dose (MED) are two and one time per week, respectively. RIR tests will be performed four times during the intervention to ensure that participants train with the prescribed intensity

Table 3 Progression of resistance training

RIR	Increase in load (%)	Equation
**1–2**	No change	–
**3**	7*	Current load (kg) × 1.07
**4**	10	Current load (kg) × 1.10
**5**	13	Current load (kg) × 1.13
**6**	16	Current load (kg) × 1.16
**7**	19	Current load (kg) × 1.19
**8**	22	Current load (kg) × 1.22
**9**	25	Current load (kg) × 1.25
**10**	28	Current load (kg) × 1.28

RIR, repetitions in reserve; *kg*, kilogrammes of weight plates on the exercise machine
The participants will take as many repetitions as possible until muscular failure in the third set during the RIR test. If the participant can complete three repetitions more than prescribed (12, 10 or 8 repetitions), the increase in load will follow the equations above and the new load will be initiated at the following resistance training session. The percentage increase in load is adapted from previous studies [102]
*Minimum increase with one weight plate corresponding to 2.5–10 kg depending on the specific exercise machine

of the exercise will be adjusted if the systematic increase in load does not fit perfectly in practise, due to the limitations regarding the different relationship between upper-/lower-body exercises and single-/multi-joint exercises [105]. To ascertain compliance to the intervention and quality of the training, all exercise sessions will be supervised by educated trainers (see below). On a weekly basis, the educated and experienced trainers will adjust the individual exercise plans to accommodate individual participant preferences in terms of exercise modality and to avoid potential overuse injuries. The aerobic training modality is individualised based on preferences, however, to minimise the risk of injuries associated with running [105], only walking, cycling, and cross-training will be allowed. In terms of resistance training, if participants report any pain or discomfort associated with a given exercise, other exercises focusing on the same muscle groups will be selected while intensity (1–3 RIR) will be assured.

Modifications and strategy to maintain and improve adherence

Exercise intervention

Compliance will be monitored by the trainers continuously through the study. During the training sessions, the trainers will investigate whether the participants need help to handle barriers regarding the intervention initiatives. If a participant completes less than 80% of the training volume prescribed over a 1-week period, procedures to prevent drop-out will be initiated. A 1-week vacation will be allowed, where the participants will receive exercise programmes that will be feasible to complete at the vacation location. The programmes will closely mimic the assigned intervention. In case that the exercise volume is unfeasible during vacation, the volume will be reduced. Subsequently, a specific plan to reach the overall exercise volume (across 16 weeks) will be drafted in collaboration with the participant, e.g. increasing some of the existing exercise sessions by 15–30 min over a period until the target volume has been reached.

The drop-out prevention procedures for the exercise intervention groups include

1. The participant will be offered a consultation with an exercise trainer to help handling the worries and to help manage the time. If the lacking compliance relates to injuries, pain or resistance to exercise modality, the exercise modality may be altered, whereas the exercise intensity will be maintained.
2. If compliance is not corrected/maintained within a week based on action 1, a temporary adjusted plan will be made in collaboration between the trainers and participant with the aim of maintaining the weekly training volume by reducing the number of sessions of exercise per week, but increasing duration (unchanged intensity).
3. If this is not sufficient to correct/maintain compliance, the training volume will be reduced for a short period of time by retracting 1/3 of the exercise sessions per week for 2 weeks. During this process, a plan to restore the training volume will be formed in collaboration between the trainer and the participant.

Adherence assessment

Posture allocation and physical activity behaviour will be measured using three-axial accelerometer-based physical activity monitors (Axivity AX3, Newcastle, UK). All heart rate profiles will be recorded during the exercise interventions (Polar V800, Polar, Holte, DK). As multiple modalities will be allowed to target the muscle groups described in the training plan, all participants in the active group will receive a sheet with pictograms of possible exercises available in the training centre (see Additional file 1 for an example). The final sheets will be formed when final training locations are identified. The participant will note down the resistance and RIR after each exercise modality. Moreover, if the participant does not complete or only partially completes the session as prescribed, the proportion of completion and reasons for not full completion will be noted on the sheet. Structured and systematic RIR testing will be performed to ascertain the planned resistance training intensity (see Fig. 5). In periods between RIR testing, the trainers will randomly perform RIR tests to check the validity of the

self-reported RIR during the sessions and adjust the load if needed.

Education of intervention personnel

Trainers who study sports science or physiotherapy will be recruited for the training intervention and a registered clinical dietician will be recruited for the dietary intervention. The primary working tasks of the trainers and the clinical dietician will be to assure compliance with the protocol and to prevent loss-to-follow-up. The trainers will also be responsible for motivating participants and making individual adjustments to the participants' exercise training programmes in order to reduce the risk of injuries. All intervention personnel will partake in meetings, presentations, and discussions led by the principal investigators and clinicians, which will cover the following:

– The research protocol
– Disease pathophysiology (type 2 diabetes mellitus)
– The intervention: organisations and collaborators, exercise selections, physical activity, and diet
– Motivation
– Potential medical issues during the intervention (including a course in cardiopulmonary resuscitation)

Concomitant care (all participants)

Pharmacological management procedures and algorithm

At visits 1, 4, and 5, biochemical markers of glucose control, lipids, and blood pressure will be assessed by the study endocrinologist, who will be blinded for subject allocation. Pharmacological management will be conducted in conjunction with self-reported symptoms of hypotension (e.g. dizziness, especially at standing up from a sitting/laying position, confusion, and fatigue) or subjected signs of hypoglycaemia (e.g. hunger, trembling, heart racing, nausea, and sweating). Pharmacological management will follow the trial algorithm for treatment targets and pharmacological titration (Additional file 2). Lipid-lowering treatment will continue at any given dosage if LDL cholesterol is < 2,5 mmol/L at inclusion. The study endocrinologist will manage the medication in accordance with the pre-defined algorithms. All changes in pharmacological treatment and adherence will be logged for later analysis. The participants will moreover be informed about side effects as well as subjective signs of increased hypo- or hyperglycaemia (thirst, fatigue, polyuria, confusion) or hypotension and urged to contact the study nurse in case of any adverse symptoms. Safety criteria will include adverse events, health-related outcomes (e.g. symptoms resembling episodes of angina pectoris or signs of cardiac arrhythmias) and participant-reported hypoglycaemic episodes (plasma glucose < 4 mmol/l, see below). Minor hypoglycaemic episodes will be defined as those that can be self-treated; major episodes will be defined as plasma glucose < 3 mmol/l or episodes requiring third-party assistance or medical intervention. In case of unacceptable adverse effects, medication will be changed according to titration described earlier. In case of participant-reported (see above) symptoms of hypo- or hyperglycaemia or hypotension, the participant will be asked to measure blood glucose using a blood glucose metre (for 3 consecutive days: morning (fasting), before evening meal, and 2 h after evening meal (postprandial)) and blood pressure profiles (18 home-based resting measurements across 3 days with 3 measurements in the morning and evening). The blood glucose and blood pressure profiles will be assessed by the nurse and presented to the study endocrinologist in a blinded manner, and the endocrinologist will manage the pharmacological treatment based on the algorithm.

Study endpoints

An overview of outcome assessment is found in Table 4.

Primary outcome

The between groups differences for change in the late-phase disposition index (DI) from baseline (0 weeks) to follow-up (16 weeks) during the final 30 min of the hyperglycaemic phase of the hyperglycaemic clamp.

Secondary outcomes

Secondary measurements of pancreatic β- and α-cell function, post prandial glycaemic control, visceral and organ-specific fat content, body anthropometrics, blood glucose control, blood lipids, blood pressure, gastric emptying, physical fitness, and incretin response. All outcomes designated for the primary article on β-cell function are listed in Table 5.

Other outcomes

Other outcomes designated to investigate the effects on glycaemic variability, markers of systemic oxidative stress, low-grade inflammation, and markers of glycation are depicted in Additional file 3. These outcomes will be included in the article, assessing systemic oxidative stress and diabetic complications.

Data collection methods

Precautions prior to testing

In order to avoid any interferences of drugs, physical activity, etc. on the various measurements, the participants will be informed about the following restrictions:

Visits 1, 2, 6, and 7

- All glucose-lowering drugs and cholesterol-lowering drugs must be discontinued for 48 h

Table 4 Outcome assessment during the intervention (SPIRIT figure)

**Participant timeline**	**Week**	**− 8 >**	**− 2**	**− 1**	**0**	**4**	**12**	**17**	**18**	**18**
	**Domain**	**Visit 0**	**Visit 1**	**Visit 2**	**Visit 3**	**Visit 4**	**Visit 5**	**Visit 6**	**Visit 7**	**Visit 8**
**Intervention period**					X	X	X			
**Primary outcome**										
Hyperglycaemic clamp	β-cell function			X					X	
**Secondary and exploratory outcomes**										
Clinical blood sampling	Clinical, functional, metabolic markers of mechanisms[1]	X	X			X	X	X		
Urine sampling	Systemic markers of oxidative stress[2]		X			X	X	X		
Mixed meal tolerance test	Glycaemic control during mixed meal tolerance test[3]		X					X		
Continuous glucose monitoring	24-h glucose profile		X					X		
Muscle and fat biopsies	Muscle and fat profiling[4]			X					X	
Cardiovascular procedures										
Home blood pressure	Resting systolic and diastolic blood pressure		X			X	X	X		
Body composition										
Magnetic resonance imaging	Visceral fat mass				X					X
Magnetic resonance spectroscopy	Pancreatic and hepatic fat deposition				X					X
Dual-energy X-ray absorptiometry	BW, BMI, LBM, FM		X					X		
Physical function										
Cardiorespiratory fitness	Maximal aerobic capacity		X					X		
Muscular strength	1RM		X					X		
Physical activity behaviour	Accelerometer-based physical activity monitors		X			X	X	X		
Patient-reported outcomes										
Mental and physical well-being	SF36 questionnaire			X					X	
Quality of life	SF36 questionnaire			X					X	
Satiety	VAS		X					X		
Dietary records	3-day record			X		X	X	X		

BW, body weight; *BMI*, body mass index; *LBM*, lean body mass; *FM*, fat mass; *1RM*, one-repetition maximum; *VAS*, visual analogue scale
1. Total cholesterol, low- and high-density lipoprotein, HbA1c, interferon-ɣ, interleukin 10, interleukin 8, interleukin 6, interleukin 1, tumour necrosis factor α, Advanced Glycation End-products (AGE), receptor for AGE (RAGE).
2. 8-oxoGuo and 8-oxodG
3. iAUC, tAUC of glucose, insulin, glucagon and C-peptide, circulation markers of appetite regulation and gastric emptying.
4. Muscle progenitor cell isolation, snap freeze and tissue-tek.

- Fasting must be initiated at least 10 h prior to testing
- No exercise 36–48 h prior to testing
- No caffeine 24 h prior to testing
- No alcohol 48 h prior to testing
- No smoking 8 h prior to testing
- No Antacida, NSAIDs, paracetamol, or PPIs 24 h prior to testing

Visits 4 and 5

- All exercise must be discontinued 36 h prior to testing
- No caffeine 24 h prior to testing
- No alcohol 48 h prior to testing
- No smoking 8 h prior to testing

Training and certification plans

All testing except the MRI and MRS will take place at the Centre for Physical Activity Research, Rigshospitalet Section 7641, Blegdamsvej 9, DK-2100 Copenhagen. All test personal will receive extensive training in all relevant standard operating procedures. Moreover, relevant staff will receive training and certification in DXA scanning.

Table 5 Overview of outcomes and variables designated article 1 concerning β-cell function

Outcome	Time frame	Domain	Measurements
Primary	Baseline and after 16 weeks	β-cell function	Late-phase DI from hyperglycaemic phase
Secondary	Baseline and after 16 weeks	Secondary measures of pancreatic α- and β-cell function.	Hyperglycaemic clamp<br>• GLP-1 stimulated insulin, C-peptide and glucagon secretion<br>• Arginine stimulated insulin, C-peptide and glucagon secretion<br>• 1st phase C-peptide and insulin secretion defined as the peak concentration during the initial 10 min of the hyperglycaemic clamp<br>• Late-phase S_I (mean Glucose infusion rate over last 30 min of clamp phase/ (mean insulin × glucose))<br>• Early phase DI (DI from 0 to 30 min)<br>• Rate of glucose disappearance (R_d)<br>• Rate of glucose appearance (R_a)
Secondary	Baseline and after 16 weeks	Post prandial glycaemic control	Mixed meal tolerance test (MMTT)<br>• iAUC of glucose, insulin, glucagon and C-peptide<br>• tAUC of glucose, insulin, glucagon and C-peptide<br>• Indices of insulin secretion, insulin sensitivity and β-cell function
Secondary	Baseline and after 16 weeks	Visceral and organ-specific fat content	Magnet Resonance (MR)
Secondary	Baseline and after 16 weeks	Body anthropometrics	• Body weight<br>• Body mass index<br>• Lean body mass<br>• Total fat mass
Secondary	Baseline, 4 weeks, 12 weeks and after 16 weeks	Blood glucose control	• HbA1c<br>• Fasting glucose<br>• Fasting C-peptide and insulin
Secondary	Baseline, 4 weeks, 12 weeks and after 16 weeks	Blood lipids	• Total cholesterol<br>• Triglyceride<br>• Low- and high-density lipoprotein
Secondary	Baseline, 4 weeks, 12 weeks and after 16 weeks	Blood pressure	• Resting systolic and diastolic blood pressure
Secondary	Baseline and after 16 weeks	Gastric emptying	Mixed meal tolerance test (MMTT)<br>• Rate of appearance of paracetamol
Secondary	Baseline and after 16 weeks	Physical fitness	• Maximal aerobic capacity (VO_2 peak)<br>• One-repetition maximum (RM) strength
Secondary	Baseline and after 16 weeks	Incretin response	Mixed meal tolerance test (MMTT)<br>• Blood levels of incretins

iAUC, incremental area under the curve; *tAUC*, total area under the curve; *DI*, disposition index

Primary outcome and key secondary outcome

Hyperglycaemic clamp

For the 3-stage hyperglycaemic clamp, an antecubital venous catheter will be placed for infusion and another line will be placed in the opposite arm for blood sampling. After baseline blood sampling ($t = -120$ min) tracer primer will be injected and infusions will be initiated as presented in Fig. 6. Glucose and glycerol will be used as tracers in order to assess rate of appearance and disappearance of glucose, and glycerol kinetics will allow us to estimate lipolysis. Glucose clamp level will be 5.4 mM above fasting glucose (post-intervention clamp level will be equal to the pre-intervention clamp level). Increase in blood glucose will be initiated by a square-wave glucose infusion lasting 15 min. After this, glucose concentration will be kept constant and glucose infusion rates will be adjusted based on blood glucose measurements (ABL 8 series, Radiometer, Denmark), obtained every 5th min according to an automated algorithm [71]. At $t = 120$ min, the hyperglycaemic + GLP-1 stage will be commenced by infusing a primed (0.5 pmol/kg), continuous GLP-1 infusion, mimicking postprandial levels in healthy individuals. At $t = 180$ min, the arginine stage will be commenced with intravenous injection of arginine hydrochloride (5 g given over 30 s) to assess maximal insulin secretory capacity. At $t = 190$ min, the clamp will be terminated. At the start of the study day and prior to baseline sampling, participants will be asked to empty their bladder and subsequently all urine will be collected throughout the clamp. To ensure collection of the total urine volume, the participants will be asked to empty their bladder one final time just after the last blood sample ($t = 190$ min). Furthermore, spot urine samples will be collected when the participant goes to the bathroom and the time will be noted on the samples. Participants will be encouraged to keep as calm as possible during the clamp session and, if possible, refrain from bathroom breaks at times critical to outcome assessment (e.g. last

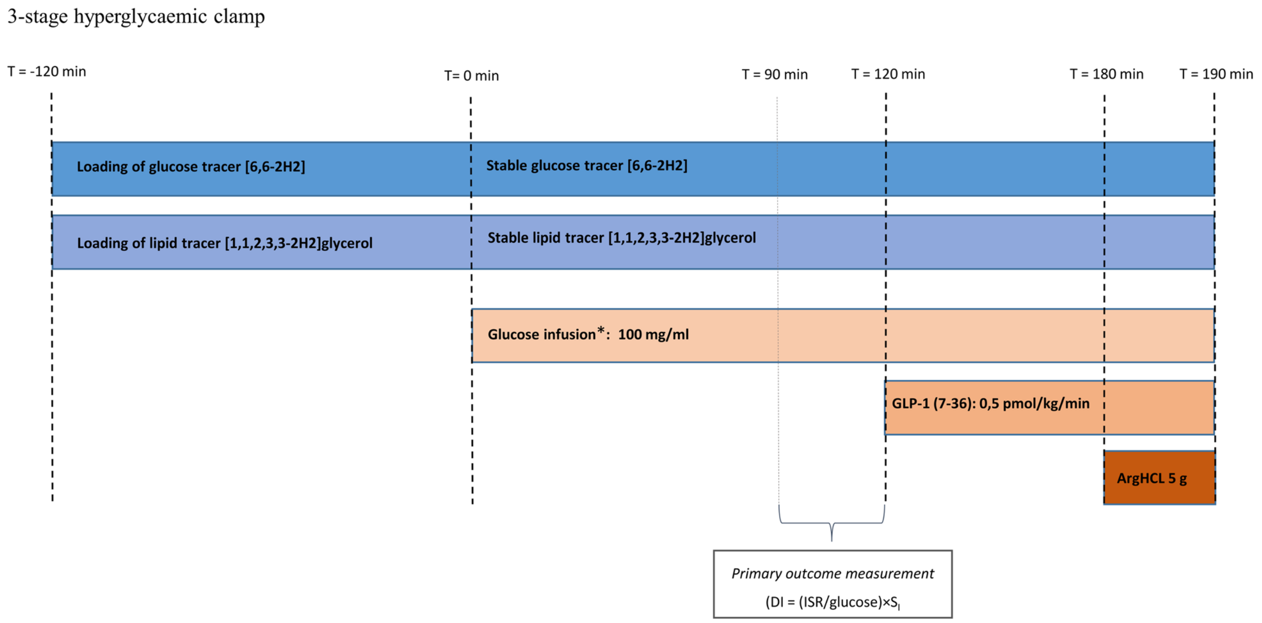

Fig. 6 Three-stage hyperglycaemic clamp. *Glucose infusion aiming at blood glucose = 5.4 mmol/l above basal. T = time. The hyperglycaemic clamp is used to evaluate beta-cell function. Participants will meet fasting in the laboratory in the morning. After placement of an antecubital venous catheter in both arms, a tracer prime dose (concentration calculated from fasting glucose and body weight) will be injected and infusion of tracers will commence at T = – 120 min. Tracer infusion will continue for 2 h to reach saturation in the circulation. During the 2-h tracer loading period, the muscle and adipose tissue biopsies will be collected. At baseline (time 0), the glucose infusion will be initiated aiming to reach blood glucose concentration at 5.4 mM above fasting. Simultaneously, the tracer infusion rate will be adjusted accordingly. Blood glucose will be measured every 5th minute throughout the remaining clamp and glucose infusion rate (GIR) will be adjusted according to the algorithm. At T = 120 min, the GLP-1 infusion is added. After another hour (T = 180), the arginine bolus is administered, and blood samples are taken continuously for 10 min before ending the clamp

30 min of in the hyperglycaemic phase). Endogenous glucose control will be ensured before removing the catheters and allowing the participant to leave the study site. For a detailed description of blood sampling throughout the clamp, please see Additional file 4.

Secondary and explorative outcomes

Clinical examination, clinical blood, and urine sampling

A medical history and examination (stethoscopy of heart and lungs, foot exam including peripheral pulse conditions, body weight, height, and electrocardiography) will be performed by standard procedures. Blood sampling will be conducted at all visits by standard procedures. The blood samples (25 ml) will be analysed for cholesterols, triglycerides, glucose, C-peptide, insulin, HbA1c, haematology, electrolytes, liver and renal status, and endocrinology (including human chorionic gonadotropin, if relevant) at the Department of Clinical Biochemistry, section 3011, Rigshospitalet. Spot urine will be collected throughout the study at the beginning of study days and frozen immediately after collection. Urine will be used to measure 8-oxo-7,8-dihydroguanosine (8-oxoGuo) and 8-oxo-7,8-dihydro-2′-deoxyguanosine (8-oxodG), using a validated method of ultra-performance liquid chromatography and tandem mass spectrometry [106].

Blood pressure will be monitored by standard procedure for home blood pressure measurements using a calibrated Microlife BP A3 Plus blood pressure monitor (Microlife AG Swiss Corporation Espenstrasse 139, CH-*9443 Widnau/ Switzerland).*

Mixed meal tolerance test

To evaluate the postprandial glucose metabolism and gastric emptying time, a standard 3-h mixed meal tolerance test (MMTT) will be performed at baseline and after the 16-week intervention (Additional file 5). After an overnight fast, serial blood samples will be drawn at baseline, 0, 15, 30, 60, 90, 120, 150, and 180 min after intake of a 420 ml liquid meal (total energy 735 kcal) consisting of 400 ml Nestlé Resource (E%: 55/30/15, carbohydrate/fat/protein respectively) with the addition of 36 g dextrose, diluted in 20 ml of water. Furthermore, 1.5 g paracetamol will be added to the MMTT for the purpose of measurement of gastric emptying time. Participants will be asked to ingest the mixed meal in less than 2 min. Blood samples will be collected in relevant tubes and analysed for, e.g. glucagon-like peptide-1 (GLP-1) (active and total), glucagon, gastric inhibitory polypeptide (GIP), insulin, proinsulin, C-peptide, glucose, and rate of gastric emptying.

The rate of gastric emptying will be calculated as previously reported [107].

Continuous glucose monitoring
Fourteen days of continuous glucose monitoring (CGM) will be performed using a blinded CGM sensor (FreeStyle Libre Pro, Abbot Diabetes Care Ltd., UK) inserted in the subcutaneous adipose tissue on the upper arm.

Biopsies
A muscle biopsy (approx. 2–300 mg) will be obtained from m. vastus lateralis using a Bergström needle. Five millilitres of lidocaine (20 mg/ml) will be administrated as local anaesthetic before the biopsy is taken. Immediately after the biopsy, skeletal muscle progenitor cells will be isolated, and the remaining muscle tissue will be divided in two. One part will immediately be frozen in liquid nitrogen and then stored at − 80 °C for protein and RNA analysis. The second muscle part will be imbedded in tissue-tek and stored at − 80 °C for histological procedures. Abdominal subcutaneous adipose tissue biopsies (up to 200 mg) will also be obtained with a Bergström needle after administrating 2 ml Lidocaine (20 mg/ml) as local anaesthetic. The adipose tissue will be divided in two. One part will be frozen in liquid nitrogen and stored at − 80 °C and imbedded in tissue-tek. The other part will be used for isolation of adipose tissue cells.

Body composition
Abdominal magnetic resonance imaging (MRI) and magnetic resonance spectroscopy (MRS)
MRI and MRS will be performed using a Siemens Magnetom Prisma 3 Tesla matrix magnetic resonance scanner (Erlangen, Germany) at 3-mm intervals. All adipose tissue located from the diaphragm to the pelvic floor inside the peritoneum will be traced manually as the visceral fat region of interest. MRS to assess liver and pancreatic fat will be performed based on the MRI and analysed as described elsewhere [108]. All MR scans will be analysed by investigators blinded for subject allocation. This will be achieved via the investigators not being unblinded until the end of analysis.

Dual X-ray absorptiometry (DXA)
A DXA scan (Prodigy Advance, GE Medical Systems – Lunar, Madison, WI, USA) will be used to assess body fat and lean mass before and after the intervention. Participants will be in a fasting state and asked to empty their bladder prior to the scan.

Physical function
Maximal aerobic capacity
The participants will undergo a maximal graded exercise test on a bicycle ergometer for evaluation of their peak oxygen consumption (VO_{2peak}, ml oxygen per min per kg). The test will start with a 5-min warm up at 60 or 80 W for women and men, respectively. Warm up will be immediately followed by a 20 W increase every minute until volitional exhaustion. Subjects will be given strong verbal encouragement during the test. Oxygen consumption will be assessed using continuous indirect calorimetric measurements (CPET, Cosmed, Italy). The highest 20 s breath by breath average will be taken as the VO_{2peak} provided that standard criteria are fulfilled [109]. Maximal heart rate (HR_{max}, beats per min) will be determined using a Garmin Premium heart rate monitor.

Muscle strength
Maximum muscle strength will be assessed in two functional exercises performed in resistance training machines (chest press, leg extension) by 1-repetition maximum (1-RM). Four warm up sets including 10, 6, 3, and 1 repetitions with very light, light, moderate, and heavy load, respectively, will be performed. 1-RM attempts will then take place. If a lift is successful, the participant will rest for 3 min before attempting the next lift with a heavier load. The investigator will use the participant's feedback from the RIR-scale along with average velocity of each attempt to determine the subsequent attempt [110]. The load of a successful repetition will be recorded as 1-RM if an attempt with a load increase of 2.5 kg fails [103]. A failed attempt will be defined as a participant being unable to complete a lift using the proper technique through the full range of motion.

Physical activity
To evaluate leisure time physical activity all participants will be equipped with two accelerometers (AX3, Axivity, Newcastle, UK) for 4–6 consecutive days. One accelerometer will be placed on the right thigh, and the other will be placed on the right side of the lower back. Both accelerometers will be attached to the participant with a patch (Fixomull stretch, BSN medical, Germany).

Patient-reported outcomes
Mental and physical well-being from the Short Form 36 (SF-36)
The SF-36 is a short form health survey with 36 questions. It yields an 8-scale profile of functional health and well-being scores as well as psychometrically based physical and mental health summary measures and a preference-based health utility index [111]. Moreover, socio-demographic information about education, age,

ethnicity, civil status, occupation, smoking status, recreational drugs, current use or prior use of anabolic androgenic steroids, and alcohol consumption will be collected.

Diet record

A self-reported 3-day record of the participants' total dietary intake will be obtained at baseline, during the intervention period (at weeks 0, 4, and 12), and after the intervention. Digital kitchen scales (Day Kitchen Scale Digital, Schou Company A/S, Nordager 31, DK-6000 Kolding) are handed out at visit 1 and the participants are asked to weigh and note down every food item in the 3-day record. Individual adjustments to the dietary intervention will be assessed according to the trial's guidelines.

Satiety

Satiety will be recorded during the MMTT (immediately before, after 60 min, after 120 min, and immediately after the test) using a 5-item visual analogue scale (VAS).

Sample size considerations

Based on a previous study with an aerobic training volume similar to the guidelines (i.e. MED group) in a population with short T2D duration, it is expected that an exercise intervention will increase late-phase disposition index derived from a hyperglycaemic clamp by 1.5 (au.) more than the control group, with a standard deviation of 1.5 (au.) of the change in the exercise and 1.0 (au.) in the control group [71]. For a contrast in a one-way ANOVA with four means (1.5, 1.0, 0.5, 0.0) and contrast coefficients (1, 0, 0, − 1) using a two-sided significance level of 0.05, assuming an error standard deviation of 1.5 and a balanced design, a total sample size of 80 participants corresponds to an approximate statistical power of 87.7%. Thus, up to 20 participants will be recruited per group ($N = 80$ in total).

Randomisation, sequence generation, and allocation concealment

Participants will be randomly allocated, following successful completion of the baseline measurements. An independent statistician generates a computer-generated randomisation schedule in a ratio of 1:1:1:1, stratified by sex. In order to ascertain concealment, the (permuted) block sizes will not be disclosed. The schedule will be forwarded to a secretary not involved in any study procedures and stored on a password-protected computer. Sequentially numbered (according to the sequence) opaque, sealed envelopes will be prepared and stored in a locked cabinet. The envelopes will be lined with aluminium foil to render the envelope impermeable to intense light. Following the conclusion of visit 2 (V2), i.e. after the termination of the hyperglycaemic clamp, the appropriate envelope will be opened by a study nurse and the participant will be told the allocation stated on the card inside the envelope.

Blinding

Participants will be blinded for treatment allocation until group assignment at the end of the tests on V2. However, following the baseline assessment, blinding of the participants will no longer be possible. All study personal responsible for data collection will be blinded throughout the study. The participant will be informed about group allocation by the study nurse in a closed room. The study endocrinologist managing pharmacological treatment and safety will be blinded to allocation. The clinical results will be presented to the endocrinologist by the study nurse without disclosing subject allocation. As all necessary information about intervention, medical history, and adverse events can be provided to the endocrinologist by the study nurse, the blinding of the study endocrinologist will only be repealed if considered necessary, e.g. based on symptoms of severe hypoglycaemia in relation to training or serious adverse events.

Statistical analysis methods

Analysis of the primary outcome

The primary analysis will be based on the family of the intention-to-treat population, defined as the *as-observed population* (missing data will not be imputed in the primary analysis) [112, 113], and the set of participants who are as close as possible to the intended intervention protocol, i.e. per-protocol. The "Full Analysis Set" for the intention-to-treat will thus be derived from the set of all randomised participants by minimal and justified elimination of participants. Therefore, all participants allocated to an active treatment group (DCON, MED or HED) will be followed up, assessed, and analysed as members of that group irrespective of their compliance to the planned course of treatment. Sensitivity analyses will be performed using the potentially biased but conservative non-responder imputation (*baseline observation carried forward* technique) as well as the current best practice multiple imputation procedure [112]. Patterns of missing data will be investigated. A priori, the less restrictive missing at random (MAR) assumption is considered more reasonable than data missing completely at random (MCAR). Assuming that the data on potential dropouts are MAR, multiple imputation procedures will be applicable to handle missing data for all participants with baseline measurements.

The analyses of the primary outcome will be performed using a repeated measures analysis of covariance applied using mixed linear modelling [113, 114]. Mean

change score of DI will be applied as the dependent outcome variable, whereas group (4 levels), time (2 levels), the interaction between time and group, sex (2 levels), and the baseline value of DI are included as independent variables and participant identifier as random effect. The potentially biased *per-protocol* population analysis will be adjusted for putative confounders: XXX, YYY, and ZZZ. The assumptions for using the linear models will be checked to confirm normal distribution of the residuals and the homogeneity of the variance (standardised residuals vs. the predicted values).

If the global test indicates between-group differences ($H_{0,\mathrm{DCON}} = H_{0,\mathrm{MED}} = H_{0,\mathrm{HED}} = H_{0,\mathrm{CON}};\ p < 0.1$), pairwise between-group differences will be explored. To maintain the family-wise type 1 error rate, a hierarchical analytic approach is engaged [115]; if we fail to progress from any of the subsequent steps ($p > 0.05$), we will interpret p values and CIs numerically as indicators of associations. Between-group comparisons for effect size estimation (difference in change from 0 to 16 weeks, based on a superiority assumption) will be completed in the following order:

1) CON vs. HED. If a difference is present ($p < 0.05$, 2-sided), then the next between-group comparison is performed. If not, then sequence is terminated.
2) CON vs. MED. If a difference is present ($p < 0.05$, 2-sided), then the next between-group comparison is performed. If not, then sequence is terminated.
3) CON vs. DCON. If a difference is present ($p < 0.05$, 2-sided), then the next between-group comparison is performed. If not, then sequence is terminated.
4) DCON vs. HED. If a difference is present ($p < 0.05$, 2-sided), then the next between-group comparison is performed. If not, then sequence is terminated.
5) DCON vs. MED. If a difference is present ($p < 0.05$, 2-sided), then the next between-group comparison is performed. If not, then sequence is terminated.
6) MED vs. HED.

The per-protocol population will be defined as participants (all criteria present):

1. CON
   - The primary outcome is assessed at both baseline and after 16 weeks follow-up (i.e. complete case).
2. DCON
   - The primary outcome is assessed at both baseline and after 16 weeks follow-up (i.e. complete case).
   - Do not exceed ± 30% of the prescribed energy intake as assessed by their dietary records (assessed as the mean energy intake across that latter 16 weeks, excluding 1-week vacation administered following week 2 of the intervention)
3. MED and HED
   - The primary outcome is assessed at both baseline and after 16 weeks follow-up (i.e. complete case).
   - ≥ 70% of the prescribed exercise volume across the intervention period (excluding weeks 1, 2 + 1-week vacation administered following week 2 of the intervention).
   - Do not exceed ± 30% of the prescribed energy intake as assessed by their dietary records (assessed as the mean energy intake across that latter 16 weeks, excluding 1-week vacation administered following week 2 of the intervention)

Analyses of the secondary outcomes

Other continuous secondary outcomes, assessed before and after the intervention period, will be analysed by analysis of covariance (ANCOVA) with the mean change score of the variable as dependent variable and group (4 levels), sex (2 levels), and the baseline value of the variable as independent variables. Continuous variables, additionally assessed during the intervention period, will be analysed within the framework of repeated measures linear mixed models. The model includes treatment (4 levels), time (2 levels), sex (2 levels), and the possible interaction between treatment (group) and time (weeks) as fixed effects, with the baseline value of the relevant variable as a covariate and participant ID as random effect. The assumptions will be investigated as described above. Variables not meeting the model assumptions will be transformed using appropriate transformations. If no suitable transformation is identified, the median change with interquartile ranges will be reported and testing will be performed using suitable non-parametric statistical tests (e.g. Wilcoxon signed rank tests). Binary outcomes will be reported as numbers and proportions and compared using a X^2 test or Fisher's exact statistics.

Retention

All participants will receive up to DKK 6000 (€800) to cover lost earnings, transport, and discomfort. The transaction will be completed upon completion of the study (all four full laboratory days (V1, V2, V6, and V7) or upon withdrawal). For every completed full day of laboratory testing, participants will receive 1.000 DKK. Moreover, DKK 500 in compensation will be added per biopsy (up to 4 in total). To prevent loss-to-follow-up amongst participants in the CON, we will offer three supervised training sessions and free membership in a fitness centre for 16 weeks following final testing.

Data management

The web-based Clinical Trial Management System EasyTrial will be used for data entry and management (EasyTrial ApS). EasyTrial has been approved by the Danish Data Protection Board. Electronic case report forms (eCRF) and questionnaires will be generated by the sponsor in EasyTrial. Fields have been programmed with acceptable ranges for data entry. All paper material (CRF, blood screen results, questionnaires, and dietary records) will be collected and stored in a locked cabinet at CFAS, Rigshospitalet Denmark. All information from the paper material will be entered twice by in non-consecutive order into the electronic back-end system. In case of discrepancies between the entries, the original paper record will be consulted. Upon completion of the study, all paper material will be scanned and stored on the secured hospital server in an electronic form. All paper material, except for the consent form, will subsequently be destroyed. Data management will be performed using appropriate statistical software.

To enable pseudonymised data, all participants will be ascribed a unique participant identification (ID) number. The identification key (ID number to personal information) will be stored on a password-protected computer, separate from the unique ID number and the database. Printed data will be kept in a separate locked area with limited access. All patient-related information obtained during the study will be handled in accordance with the Danish law for protection of personal data ("lov om behandling af personoplysninger") and the Danish health law ("sundhedsloven"). The blood samples will be registered from the hospital blood sample portal (Labka) and para-clinical observations will be obtained through "Sundhedsportalen". The study has been reported to the Danish Data Protection Agency ("Region H's paraplyanmeldelse") VD-2018-516/ I-suite no. 6768.

Harms, risks, and discomforts

Adverse events (AE) and safety evaluation

In this study, we have adopted the ICH definition of adverse event (AE) (E2A).

An AE is thus defined as; "An adverse event (AE) can therefore be any unfavourable and unintended sign (including an abnormal laboratory finding, for example), symptom, or disease temporally associated with the use of a medicinal product, whether or not considered related to the medicinal product" [116].

Serious AE (SAE) is defined as; "[...] any untoward medical occurrence that at any dose: * results in death, * is life-threatening, NOTE: The term "life-threatening" in the definition of "serious" refers to an event in which the patient was at risk of death at the time of the event; it does not refer to an event which hypothetically might have caused death if it were more severe. * requires inpatient hospitalisation or prolongation of existing hospitalisation, * results in persistent or significant disability/incapacity, or * is a congenital anomaly/birth defect" [116].

AEs/SAEs (anticipated and unanticipated) will be recorded on adverse event forms. These forms will include a description and classification of the event, date of onset, date resolved, whether the event was serious or not (ICH criteria), relationship of the event to the study (1 = none, 2 = unlikely, 3 = possible, 4 = probable, 5 = definitely), action taken, and whether the study was suspended or not. All SAEs will be reported to the Regional Ethical Committee. AEs observed by any investigator and/or reported by the participant will be reported in the source data and case report form from the first (signature of informed consent) to the last protocol-specific procedure allocation [117].

VO_2-max test and 1RM Physical fitness and strength tests, where subjects must put in maximal effort. The tests can cause some degree of breathlessness and exhaustion, but both are standard methods used for scientific purposes at the CFAS laboratory.

DXA scan Is not expected to cause any discomfort and involves very little radiation (0.0004 mSv), corresponding to approximately 1/10 of the radiation acquired for a thoracic X-ray. The dose is smaller than the radiation received when flying in a commercial jet from (11–12 h) SST.dk—Strålingsguiden.

Hyperglycaemic clamp The hyperglycaemic clamp combined with GLP-1 infusion and arginine bolus may cause hypoglycaemic symptoms (dizziness, headache, and fatigue), following the trial. However, blood glucose will be monitored for up to 1 h after the test and a meal will be provided when testing has finished. Furthermore, there is a minor risk of infection or haematoma due to blood lines being placed. All researchers are experts in these procedures, so the risks are minimal. The hyperglycaemic clamp with GLP-1 and arginine has previously been used for scientific purposes in our laboratory. The introduction of arginine may give the participants a transient metallic taste that is short and fully reversible.

Stable isotope tracers will be used to obtain knowledge about glucose and lipid distribution. The isotopes are not radioactive and are considered safe to use.

Hyperglycaemia can lead to low blood levels of potassium. Supplementary potassium will be administered if potassium levels are low at the beginning of the clamp.

Blood sampling A small peripheral venous catheter (PVC) will be placed and may cause slight discomfort

and involves a small risk of local infection and oedema. The blood volume collected (maximum 965 ml/4 months) is considered too small to cause any symptoms.

Biopsies The local anaesthesia can be associated with short-lasting discomfort and subjects might experience some degree of muscle pain after the biopsy. Paracetamol (1000 mg) max. Four times a day will be recommended for pain management. Generally, the fat biopsy causes much less discomfort. The procedures can leave small bruises, but normally they heal nicely. Temporary, decreased sensation at the incision area or where the local anaesthetic has been injected can be seen and heals within months. Infection occurs in 1 out of 25,000 times and may in some cases require treatment with antibiotics. Participants will be informed to contact a project physician in case of any signs of infection (heat, redness, swelling, or fever).

MRI/MRS The measurements are pain free and based on radio waves and, thus, the participant is not exposed to X-rays or other sources of radiation. The scans will be performed in a tight cylinder that may cause claustrophobia. As the scans are not performed with a specific clinical purpose but rather for quantification of site-specific ectopic adipose tissue, they cannot and will not be used for diagnostic purposes. However, all scans will be screened by a trained radiologist at least 4 weeks after each scan at which occasion unexpected abnormalities may be detected. If deemed necessary and the participant so wishes (according to the consent form), the Department of Radiology, section 3024, Rigshospitalet will perform further warranted diagnostics.

Continuous glucose monitoring (CGM) The method is safe and routinely used by patients with diabetes to continuously monitor the blood glucose level. Penetration of the skin involves a small risk of infection and the study participants will be informed and instructed to act in case of symptoms of infection.

Discussion

The study is expected to result in minimal discomfort and risk for the study participants. The study examines the effects of various volumes of exercise training on pancreatic β-cell function and glucose levels in participants with short standing T2D, i.e. < 7 years. Both a rapid weight loss through diet and diet/exercise training may induce subjective signs of hypoglycaemia or hypotensive episodes. Procedures to manage medications in both the Look AHEAD and the DIRECT studies are described [87, 88]. It is thus reasonable to manage glucose- and blood pressure-lowering agents with a safety mechanism in a blinded standardised algorithm in all groups. Based on a previous study, a large proportion of the exercise training/diet groups are expected to discontinue their glucose and blood pressure-lowering medications within the study period without any adverse events [118]. Immediately following the study, the clinical parameters will be reviewed by the research physicians, and the participants will be asked to contact their general practitioner with the purpose of continuing their treatment based on the clinical guidelines [119]. Also following each participant's final study visit, the project endocrinologist will write a summary of the participant's clinical values and intervention and send this information to his/her general practitioner in a data-secure manner. The participants within the DCON/MED/HED groups will benefit from the study in terms of a thorough medical examination, increased physical capacity and increased T2D management. Based on previous research from our group, it is expected that a large proportion will maintain or even improve T2D control with this intervention [90]. Moreover, in contrast to the previous study, all exercise sessions are fully supervised; thus, it is expected that compliance to the lifestyle intervention will be even higher than previously reported (82%) [90]. The control group will also benefit from an extensive health check-up and achieve insight to basic anti-diabetic lifestyle alterations. After the project has finished, all participants will be re-referred to the various activities for patients with T2D in their local municipalities. Furthermore, participants in the CON group will be offered 16 weeks of supervised training after the intervention. If participants in the CON group do not wish or are unable to attend the rehabilitation programme, then a dietary plan and an extensive individualised training programme (based on the intervention provided in the DCON/MED/HED groups) will be provided.

We consider this a valuable and sound study that will contribute to the essential knowledge of possible T2D remission induced by non-surgical and non-pharmacological lifestyle intervention. Specifically, it will improve our current knowledge about if and how exercise training intervention, when administered in concert with diet-induced weight loss, affects pancreatic β-cell function in patients with T2D and possibly provides a sound alternative to conventional high-risk procedures. Moreover, the study will elucidate the time dependency and causality between the pathophysiological processes of cardiovascular damage and add to the body of evidence about how and if exercise training intervention may decrease the risk of the micro- and macrovascular complications induced by T2D. The vast amount of data collected leaves room for exploratory outcomes and, thus, hypotheses-generating studies on e.g. mitochondrial function and density, metabolomics and proteomics from urine and blood samples, circulating biomarkers of organ and/or arterial function.

In the end, the project will be an important stepping-stone in the process of developing efficient lifestyle interventions with both curative and secondary prevention purposes in the clinical care of T2D.

Trial status

The enrolment period began on 15-12-2019 and is open until $N = 80$ T2D or until 01-12-2021, whichever is reached first. However, given the present COVID19 pandemic, the time period may be extended further in order to gather $N = 80$ as the inclusion of new participants was suspended on March 13th, 2020, until May 11th, 2020 (see Additional file 6). This is protocol version number 4.

Abbreviations

AE: Adverse event; AGEs: Advanced glycation end-products; ATP: Adenosine triphosphate; CGM: Continuous glucose monitoring; CON: Control group; DAG: Diacylglycerol; DCON: Dietary control group; DXA: Dual X-ray absorptiometry; esRAGE: Endogenous soluble receptor for advanced glycation end-products; ER: Endoplasmic reticulum; GLP1: Glucagon-like peptide-1; GLUT: Glucose transporter type; GV: Glycaemic variability; HbA1c: Glycated haemoglobin; HED: High exercise dose group; IL: Interleukin; MAR: Missing at random; MCAR: Missing completely at random; MED: Moderate exercise dose group; MMTT: Mixed meal tolerance test; MRI: Magnetic resonance imaging; MRS: Magnetic resonance spectroscopy; OS: Oxidative stress; PKCε: Protein kinase C epsilon type; PVC: Peripheral venous catheter; RAGE: Receptor for advanced glycation end-products; ROS: Reactive oxygen species; SAE: Serious adverse event; SC: Satellite cell; sRAGE: Soluble receptor for advanced glycation end-products; TAG: Triacylglycerol; T2D: Type 2 diabetes mellitus

Supplementary Information

The online version contains supplementary material available at https://doi.org/10.1186/s13063-021-05207-7.

Additional file 1. Aerobic training programmes and resistance training exercises completed during the intervention period.

Additional file 2. Algorithm for pharmacological management.

Additional file 3. Outcome Table Article 2.

Additional file 4. Hyperglycaemic Clamp Blood Sampling Schedule.

Additional file 5. Mixed Meal Tolerance Test.

Additional file 6. Suspension Note.

Additional file 7. Informed Consent.

Acknowledgements

We would like to acknowledge Else Danielsen and Karen Kettless for advice and support on MRI and MRS imaging. Furthermore, we would like to acknowledge Søren Nielsen and Anne-Kristine Meinild Lundby for guidance and advice on laboratory procedures.

Authors' contributions

BKP, KK, TPA, and MRL conceived the study. MPPL, GEL, and MRL led the proposal. MPPL, GEL, MRL, TPA, KK, TS, HEP, ELL, RC, HE, and GVH contributed to protocol development. MRL is the Chief Investigator. MPPL, GEL, MRL, TPA, KK, TS, HEP, ELL, RC, HE, GVH, CSF, SLB, VR, NM, CF, KSK, ABN, CE, and CAL all participated in designing the study. Statistical advisor: RC. Methodological advisors: TS, GVH, HEP. DOSE-EX steering committee: MRL, TPA, BKP. All authors read and approved the final manuscript.

Funding

The Centre for Physical Activity Research (CFAS) is supported by TrygFonden (info@trygfonden.dk). The DOSE-EX study is supported by an additional grant from TrygFonden (ID: 124708) and Svend Andersen Fonden. Robin Christensen from the Parker Institute, Bispebjerg and Frederiksberg Hospital is supported by a core grant from the Oak Foundation (OCAY-18-774-OFIL). MPPL is funded by The Danish Diabetes Academy.
None of the funding agencies has taken part in protocol drafting and they will not take part in completion of the study, data collection, nor interpretation or publishing of the data from this trial.

Availability of data and materials

The data from the DOSE-EX study will be published in international peer-reviewed journals. All results will be reported according to the CONSORT guidelines [120]. Positive, negative, and inconclusive data will all be disseminated and published. All authors must comply with the "Uniform Requirements for Manuscripts Submitted to Biomedical Journals" [121].
All data are the property of CFAS and access to data will be overseen by the DOSE-EX steering committee. All members of the DOSE-EX study group will have access to the anonymised, cleaned data set upon completion of the final post-intervention testing after approval from the steering committee (based on an approved proposal or with specific reason provided). Following publication of the primary outcome, other researchers may request access to the data following an approved (by the steering committee) research proposal.

Declarations

Ethics approval and consent to participate

The study has been approved by the Scientific Ethical Committee at the Capital Region of Denmark (Approval number: H-18038298) and the study will be conducted in accordance with the Declaration of Helsinki (1964) with its subsequent revisions. Written, informed consent to participate will be obtained from all participants (see Additional file 7).

Consent for publication

Not applicable.

Competing interests

The authors declare that they have no competing interests.

Author details

[1]Centre for Physical Activity Research, Copenhagen University Hospital - Rigshospitalet, Copenhagen, Denmark. [2]Department of Radiology, Copenhagen University Hospital - Rigshospitalet, Copenhagen, Denmark. [3]Bachelor's Degree Programme in Radiography, Copenhagen University College, Copenhagen, Denmark. [4]Department of Clinical Pharmacology, Bispebjerg-Frederiksberg Hospital, University of Copenhagen, Copenhagen, Denmark. [5]Department of Clinical Medicine, University of Copenhagen, Copenhagen, Denmark. [6]Musculoskeletal Statistics Unit, The Parker Institute, Bispebjerg and Frederiksberg Hospital, Copenhagen, Denmark. [7]Department of Clinical Research, Research Unit of Rheumatology, University of Southern Denmark, Odense University Hospital, Odense, Denmark. [8]Biomedical Sciences, Faculty of Health & Medical Science, University of Copenhagen & Clinical Metabolomics Core Facility, Clinical Biochemistry, Rigshospitalet, Copenhagen, Denmark. [9]Blazon Scientific, London, UK. [10]Department of Endocrinology PE, Rigshospitalet, University of Copenhagen, Copenhagen, Denmark. [11]Department of Immunology & Microbiology, University of Copenhagen, Copenhagen, Denmark.

Received: 30 November 2020 Accepted: 18 March 2021
Published online: 01 April 2021

References

1. Sherwani SI, et al. Significance of HbA1c test in diagnosis and prognosis of diabetic patients. Biomark Insights. 2016;11:95–104.
2. Colberg SR, Sigal RJ, Fernhall B, Regensteiner JG, Blissmer BJ, Rubin RR, Chasan-Taber L, Albright AL, Braun B. Exercise and type 2 diabetes: the American College of Sports Medicine and the American Diabetes Association: joint position statement. Diabetes Care. 2010;33(12):e147–67. https://doi.org/10.2337/dc10-9990.
3. Colberg SR, Sigal RJ, Yardley JE, Riddell MC, Dunstan DW, Dempsey PC, Horton ES, Castorino K, Tate DF. Physical activity/exercise and diabetes: a position statement of the American Diabetes Association. Diabetes Care. 2016;39(11):2065–79. https://doi.org/10.2337/dc16-1728.

4. Boule NG, et al. Meta-analysis of the effect of structured exercise training on cardiorespiratory fitness in type 2 diabetes mellitus. Diabetologia. 2003;46(8):1071–81.
5. Umpierre D, et al. Physical activity advice only or structured exercise training and association with HbA1c levels in type 2 diabetes: a systematic review and meta-analysis. JAMA. 2011;305(17):1790–9. https://doi.org/10.1001/jama.2011.576.
6. Umpierre D, Ribeiro PAB, Schaan BD, Ribeiro JP. Volume of supervised exercise training impacts glycaemic control in patients with type 2 diabetes: a systematic review with meta-regression analysis. Diabetologia. 2013;56(2):242–51. https://doi.org/10.1007/s00125-012-2774-z.
7. Action to Control Cardiovascular Risk in Diabetes Study, G, et al. Effects of intensive glucose lowering in type 2 diabetes. N Engl J Med. 2008;358(24):2545–59.
8. Torimoto K, Okada Y, Mori H, Tanaka Y. Relationship between fluctuations in glucose levels measured by continuous glucose monitoring and vascular endothelial dysfunction in type 2 diabetes mellitus. Cardiovasc Diabetol. 2013;12(1):1–1. https://doi.org/10.1186/1475-2840-12-1.
9. Hirsch IB, Brownlee M. Should minimal blood glucose variability become the gold standard of glycemic control? J Diabetes Complicat. 2005;19(3):178–81. https://doi.org/10.1016/j.jdiacomp.2004.10.001.
10. Monnier L, Mas E, Ginet C, Michel F, Villon L, Cristol JP, Colette C. Activation of oxidative stress by acute glucose fluctuations compared with sustained chronic hyperglycemia in patients with type 2 diabetes. Jama. 2006;295(14):1681–7. https://doi.org/10.1001/jama.295.14.1681.
11. Geidl W, Schlesinger S, Mino E, Miranda L, Pfeifer K. Dose-response relationship between physical activity and mortality in adults with noncommunicable diseases: a systematic review and meta-analysis of prospective observational studies. Int J Behav Nutr Phys Act. 2020;17(1):109. https://doi.org/10.1186/s12966-020-01007-5.
12. Taylor R. Insulin resistance and type 2 diabetes. Diabetes. 2012;61(4):778–9. https://doi.org/10.2337/db12-0073.
13. Defronzo RA. Banting lecture. From the triumvirate to the ominous octet: a new paradigm for the treatment of type 2 diabetes mellitus. Diabetes. 2009;58(4):773–95. https://doi.org/10.2337/db09-9028.
14. Rudenski AS, Hadden DR, Atkinson AB, Kennedy L, Matthews DR, Merrett JD, Pockaj B, Turner RC. Natural history of pancreatic islet B-cell function in type 2 diabetes mellitus studied over six years by homeostasis model assessment. Diabet Med. 1988;5(1):36–41. https://doi.org/10.1111/j.1464-5491.1988.tb00938.x.
15. Esser N, Utzschneider KM, Kahn SE. Early beta cell dysfunction vs insulin hypersecretion as the primary event in the pathogenesis of dysglycaemia. Diabetologia. 2020;63(10):2007–21. https://doi.org/10.1007/s00125-020-05245-x.
16. Samuel VT, Petersen KF, Shulman GI. Lipid-induced insulin resistance: unravelling the mechanism. Lancet. 2010;375(9733):2267–77. https://doi.org/10.1016/S0140-6736(10)60408-4.
17. Taylor R. Type 2 diabetes: etiology and reversibility. Diabetes Care. 2013;36(4):1047–55. https://doi.org/10.2337/dc12-1805.
18. Popa S, Mot M. Beta-Cell Function and Failure in Type 2 Diabetes. In: Masuo K, editor. Type 2 Diabetes; 2013. Chapter 2.
19. Adiels M, Taskinen MR, Packard C, Caslake MJ, Soro-Paavonen A, Westerbacka J, Vehkavaara S, Häkkinen A, Olofsson SO, Yki-Järvinen H, Borén J. Overproduction of large VLDL particles is driven by increased liver fat content in man. Diabetologia. 2006;49(4):755–65. https://doi.org/10.1007/s00125-005-0125-z.
20. Taylor R, al-Mrabeh A, Zhyzhneuskaya S, Peters C, Barnes AC, Aribisala BS, Hollingsworth KG, Mathers JC, Sattar N, Lean MEJ. Remission of human type 2 diabetes requires decrease in liver and pancreas fat content but is dependent upon capacity for beta cell recovery. Cell Metab. 2018;28(4):547–56 e3. https://doi.org/10.1016/j.cmet.2018.07.003.
21. Schwartz SS, Epstein S, Corkey BE, Grant SFA, Gavin III JR, Aguilar RB, Herman ME. A unified pathophysiological construct of diabetes and its complications. Trends Endocrinol Metab. 2017;28(9):645–55. https://doi.org/10.1016/j.tem.2017.05.005.
22. Avery L, Flynn D, van Wersch A, Sniehotta FF, Trenell MI. Changing physical activity behavior in type 2 diabetes: a systematic review and meta-analysis of behavioral interventions. Diabetes Care. 2012;35(12):2681–9. https://doi.org/10.2337/dc11-2452.
23. Boule NG, et al. Effects of exercise on glycemic control and body mass in type 2 diabetes mellitus: a meta-analysis of controlled clinical trials. JAMA. 2001;286(10):1218–27. https://doi.org/10.1001/jama.286.10.1218.
24. Zhou YP, Grill V. Long term exposure to fatty acids and ketones inhibits B-cell functions in human pancreatic islets of Langerhans. J Clin Endocrinol Metab. 1995;80(5):1584–90.
25. Briaud I, Harmon JS, Kelpe CL, Segu VBG, Poitout V. Lipotoxicity of the pancreatic beta-cell is associated with glucose-dependent esterification of fatty acids into neutral lipids. Diabetes. 2001;50(2):315–21. https://doi.org/10.2337/diabetes.50.2.315.
26. Donath MY. Targeting inflammation in the treatment of type 2 diabetes: time to start. Nat Rev Drug Discov. 2014;13(6):465–76. https://doi.org/10.1038/nrd4275.
27. Donath MY, Shoelson SE. Type 2 diabetes as an inflammatory disease. Nat Rev Immunol. 2011;11(2):98–107. https://doi.org/10.1038/nri2925.
28. Buller CL, Loberg RD, Fan MH, Zhu Q, Park JL, Vesely E, Inoki K, Guan KL, Brosius FC III. A GSK-3/TSC2/mTOR pathway regulates glucose uptake and GLUT1 glucose transporter expression. Am J Physiol Cell Physiol. 2008;295(3):C836–43. https://doi.org/10.1152/ajpcell.00554.2007.
29. Larsen CM, Faulenbach M, Vaag A, Ehses JA, Donath MY, Mandrup-Poulsen T. Sustained effects of interleukin-1 receptor antagonist treatment in type 2 diabetes. Diabetes Care. 2009;32(9):1663–8. https://doi.org/10.2337/dc09-0533.
30. Johansson K, Neovius M, Hemmingsson E. Effects of anti-obesity drugs, diet, and exercise on weight-loss maintenance after a very-low-calorie diet or low-calorie diet: a systematic review and meta-analysis of randomized controlled trials. Am J Clin Nutr. 2013;99(1):14–23.
31. Lean MEJ, Leslie WS, Barnes AC, Brosnahan N, Thom G, McCombie L, Peters C, Zhyzhneuskaya S, al-Mrabeh A, Hollingsworth KG, Rodrigues AM, Rehackova L, Adamson AJ, Sniehotta FF, Mathers JC, Ross HM, McIlvenna Y, Welsh P, Kean S, Ford I, McConnachie A, Messow CM, Sattar N, Taylor R. Durability of a primary care-led weight-management intervention for remission of type 2 diabetes: 2-year results of the DiRECT open-label, cluster-randomised trial. Lancet Diabetes Endocrinol. 2019;7(5):344–55. https://doi.org/10.1016/S2213-8587(19)30068-3.
32. Karstoft K, Pedersen BK. Exercise and type 2 diabetes: focus on metabolism and inflammation. Immunol Cell Biol. 2016;94(2):146–50. https://doi.org/10.1038/icb.2015.101.
33. Wedell-Neergaard AS, et al. Exercise-induced changes in visceral adipose tissue mass are regulated by IL-6 signaling: a randomized controlled trial. Cell Metab. 2019;29(4):844–855.e3.
34. Sylow L, Kleinert M, Richter EA, Jensen TE. Exercise-stimulated glucose uptake - regulation and implications for glycaemic control. Nat Rev Endocrinol. 2017;13(3):133–48. https://doi.org/10.1038/nrendo.2016.162.
35. Ellingsgaard H, Hauselmann I, Schuler B, Habib AM, Baggio LL, Meier DT, Eppler E, Bouzakri K, Wueest S, Muller YD, Hansen AMK, Reinecke M, Konrad D, Gassmann M, Reimann F, Halban PA, Gromada J, Drucker DJ, Gribble FM, Ehses JA, Donath MY. Interleukin-6 enhances insulin secretion by increasing glucagon-like peptide-1 secretion from L cells and alpha cells. Nat Med. 2011;17(11):1481–9. https://doi.org/10.1038/nm.2513.
36. Ellingsgaard H, Seelig E, Timper K, Coslovsky M, Soederlund L, Lyngbaek MP, Wewer Albrechtsen NJ, Schmidt-Trucksäss A, Hanssen H, Frey WO, Karstoft K, Pedersen BK, Böni-Schnetzler M, Donath MY. GLP-1 secretion is regulated by IL-6 signalling: a randomised, placebo-controlled study. Diabetologia. 2020;63(2):362–73. https://doi.org/10.1007/s00125-019-05045-y.
37. Lang Lehrskov L, et al. Interleukin-6 delays gastric emptying in humans with direct effects on glycemic control. Cell Metab. 2018;27(6):1201–1211.e3.
38. Chaudhuri J, Bains Y, Guha S, Kahn A, Hall D, Bose N, Gugliucci A, Kapahi P. The role of advanced glycation end products in aging and metabolic diseases: bridging association and causality. Cell Metab. 2018;28(3):337–52. https://doi.org/10.1016/j.cmet.2018.08.014.
39. Fishman SL, Sonmez H, Basman C, Singh V, Poretsky L. The role of advanced glycation end-products in the development of coronary artery disease in patients with and without diabetes mellitus: a review. Mol Med. 2018;24(1):59. https://doi.org/10.1186/s10020-018-0060-3.
40. Santilli F, Vazzana N, Iodice P, Lattanzio S, Liani R, Bellomo R, Lessiani G, Perego F, Saggini R, Davì G. Effects of high-amount-high-intensity exercise on in vivo platelet activation: modulation by lipid peroxidation and AGE/RAGE axis. Thromb Haemost. 2013;110(6):1232–40. https://doi.org/10.1160/TH13-04-0295.
41. Dempsey PC, Blankenship JM, Larsen RN, Sacre JW, Sethi P, Straznicky NE, Cohen ND, Cerin E, Lambert GW, Owen N, Kingwell BA, Dunstan DW. Interrupting prolonged sitting in type 2 diabetes: nocturnal persistence of improved glycaemic control. Diabetologia. 2017;60(3):499–507. https://doi.org/10.1007/s00125-016-4169-z.

42. Karstoft K, Clark MA, Jakobsen I, Müller IA, Pedersen BK, Solomon TPJ, Ried-Larsen M. The effects of 2 weeks of interval vs continuous walking training on glycaemic control and whole-body oxidative stress in individuals with type 2 diabetes: a controlled, randomised, crossover trial. Diabetologia. 2017;60(3):508–17. https://doi.org/10.1007/s00125-016-4170-6.
43. Bennetsen SL, et al. The impact of physical activity on glycemic variability assessed by continuous glucose monitoring in patients with type 2 diabetes mellitus: a systematic review. Front Endocrinol (Lausanne). 2020;11:486. https://doi.org/10.3389/fendo.2020.00486. eCollection 2020.
44. Heiskanen MA, Motiani KK, Mari A, Saunavaara V, Eskelinen JJ, Virtanen KA, Koivumäki M, Löyttyniemi E, Nuutila P, Kalliokoski KK, Hannukainen JC. Exercise training decreases pancreatic fat content and improves beta cell function regardless of baseline glucose tolerance: a randomised controlled trial. Diabetologia. 2018;61(8):1817–28. https://doi.org/10.1007/s00125-018-4627-x.
45. Curran M, Drayson MT, Andrews RC, Zoppi C, Barlow JP, Solomon TPJ, Narendran P. The benefits of physical exercise for the health of the pancreatic beta-cell: a review of the evidence. Exp Physiol. 2020;105(4):579–89. https://doi.org/10.1113/EP088220.
46. Dela F, et al. Physical training may enhance beta-cell function in type 2 diabetes. Am J Physiol Endocrinol Metab. 2004;287(5):E1024–31. https://doi.org/10.1152/ajpendo.00056.2004.
47. Haus JM, Solomon TPJ, Kelly KR, Fealy CE, Kullman EL, Scelsi AR, Lu L, Pagadala MR, McCullough AJ, Flask CA, Kirwan JP. Improved hepatic lipid composition following short-term exercise in nonalcoholic fatty liver disease. J Clin Endocrinol Metab. 2013;98(7):E1181–8. https://doi.org/10.1210/jc.2013-1229.
48. Rabol R, Petersen KF, Dufour S, Flannery C, Shulman GI. Reversal of muscle insulin resistance with exercise reduces postprandial hepatic de novo lipogenesis in insulin resistant individuals. Proc Natl Acad Sci U S A. 2011;108(33):13705–9. https://doi.org/10.1073/pnas.1110105108.
49. Keating SE, Hackett DA, George J, Johnson NA. Exercise and non-alcoholic fatty liver disease: a systematic review and meta-analysis. J Hepatol. 2012;57(1):157–66. https://doi.org/10.1016/j.jhep.2012.02.023.
50. Sondergaard E, Rahbek I, Sørensen LP, Christiansen JS, Gormsen LC, Jensen MD, Nielsen S. Effects of exercise on VLDL-triglyceride oxidation and turnover. Am J Physiol Endocrinol Metab. 2011;300(5):E939–44. https://doi.org/10.1152/ajpendo.00031.2011.
51. van der Windt DJ, Sud V, Zhang H, Tsung A, Huang H. The effects of physical exercise on fatty liver disease. Gene Expr. 2018;18(2):89–101. https://doi.org/10.3727/105221617X15124844266408.
52. Way KL, Hackett DA, Baker MK, Johnson NA. The effect of regular exercise on insulin sensitivity in type 2 diabetes mellitus: a systematic review and meta-analysis. Diabetes Metab J. 2016;40(4):253–71. https://doi.org/10.4093/dmj.2016.40.4.253.
53. Sylow L, Richter EA. Current advances in our understanding of exercise as medicine in metabolic disease. Curr Opin Physiol. 2019;12:12–9. https://doi.org/10.1016/j.cophys.2019.04.008.
54. Bird SR, Hawley JA. Update on the effects of physical activity on insulin sensitivity in humans. BMJ Open Sport Exerc Med. 2017;2(1):e000143. https://doi.org/10.1136/bmjsem-2016-000143.
55. D'Souza DM, Al-Sajee D, Hawke TJ. Diabetic myopathy: impact of diabetes mellitus on skeletal muscle progenitor cells. Front Physiol. 2013;4:379.
56. Hernández-Ochoa EO, Vanegas C. Diabetic myopathy and mechanisms of disease. Biochem Pharmacol. 2015;4(5):e179.
57. Beyer I, Mets T, Bautmans I. Chronic low-grade inflammation and age-related sarcopenia. Curr Opin Clin Nutr Metab Care. 2012;15(1):12–22. https://doi.org/10.1097/MCO.0b013e32834dd297.
58. Mesinovic J, Zengin A, de Courten B, Ebeling PR, Scott D. Sarcopenia and type 2 diabetes mellitus: a bidirectional relationship. Diabetes Metab Syndr Obes. 2019;12:1057–72. https://doi.org/10.2147/DMSO.S186600.
59. L'Honoré A, et al. The role of Pitx2 and Pitx3 in muscle stem cells gives new insights into P38α MAP kinase and redox regulation of muscle regeneration. eLife. 2018;7:e32991. https://doi.org/10.7554/eLife.32991.
60. Christov C, Chrétien F, Abou-Khalil R, Bassez G, Vallet G, Authier FJ, Bassaglia Y, Shinin V, Tajbakhsh S, Chazaud B, Gherardi RK. Muscle satellite cells and endothelial cells: close neighbors and privileged partners. Mol Biol Cell. 2007;18(4):1397–409. https://doi.org/10.1091/mbc.e06-08-0693.
61. Arsic N, Zacchigna S, Zentilin L, Ramirez-Correa G, Pattarini L, Salvi A, Sinagra G, Giacca M. Vascular endothelial growth factor stimulates skeletal muscle regeneration in vivo. Mol Ther. 2004;10(5):844–54. https://doi.org/10.1016/j.ymthe.2004.08.007.
62. Nederveen JP, Joanisse S, Snijders T, Thomas ACQ, Kumbhare D, Parise G. The influence of capillarization on satellite cell pool expansion and activation following exercise-induced muscle damage in healthy young men. J Physiol. 2018;596(6):1063–78. https://doi.org/10.1113/JP275155.
63. Kargl CK, Nie Y, Evans S, Stout J, Shannahan JH, Kuang S, Gavin TP. Factors secreted from high glucose treated endothelial cells impair expansion and differentiation of human skeletal muscle satellite cells. J Physiol. 2019;597(20):5109–24. https://doi.org/10.1113/JP278165.
64. Boya P, Codogno P, Rodriguez-Muela N. Autophagy in stem cells: repair, remodelling and metabolic reprogramming. Development.2018;145(4):dev146506. https://doi.org/10.1242/dev.146506.
65. Henriksen TI, Wigge LV, Nielsen J, Pedersen BK, Sandri M, Scheele C. Dysregulated autophagy in muscle precursor cells from humans with type 2 diabetes. Sci Rep. 2019;9(1):8169. https://doi.org/10.1038/s41598-019-44535-2.
66. Brandt N, Gunnarsson TP, Bangsbo J, Pilegaard H. Exercise and exercise training-induced increase in autophagy markers in human skeletal muscle. Physiol Rep. 2018;6(7):e13651. https://doi.org/10.14814/phy2.13651.
67. Escobar KA, Cole NH, Mermier CM, VanDusseldorp TA. Autophagy and aging: maintaining the proteome through exercise and caloric restriction. Aging Cell. 2019;18(1):e12876. https://doi.org/10.1111/acel.12876.
68. Dort J, et al. Macrophages are key regulators of stem cells during skeletal muscle regeneration and diseases. Stem Cells Int. 2019;2019:4761427.
69. Fink LN, Oberbach A, Costford SR, Chan KL, Sams A, Blüher M, Klip A. Expression of anti-inflammatory macrophage genes within skeletal muscle correlates with insulin sensitivity in human obesity and type 2 diabetes. Diabetologia. 2013;56(7):1623–8. https://doi.org/10.1007/s00125-013-2897-x.
70. Sluik D, Buijsse B, Muckelbauer R, Kaaks R, Teucher B, Johnsen NF, Tjønneland A, Overvad K, Østergaard JN, Amiano P, Ardanaz E, Bendinelli B, Pala V, Tumino R, Ricceri F, Mattiello A, Spijkerman AMW, Monninkhof EM, May AM, Franks PW, Nilsson PM, Wennberg P, Rolandsson O, Fagherazzi G, Boutron-Ruault MC, Clavel-Chapelon F, Castaño JMH, Gallo V, Boeing H, Nöthlings U. Physical activity and mortality in individuals with diabetes mellitus: a prospective study and meta-analysis. Arch Intern Med. 2012;172(17):1285–95. https://doi.org/10.1001/archinternmed.2012.3130.
71. Karstoft K, Winding K, Knudsen SH, James NG, Scheel MM, Olesen J, Holst JJ, Pedersen BK, Solomon TPJ. Mechanisms behind the superior effects of interval vs continuous training on glycaemic control in individuals with type 2 diabetes: a randomised controlled trial. Diabetologia. 2014;57(10):2081–93. https://doi.org/10.1007/s00125-014-3334-5.
72. Rogers MA, Yamamoto C, King DS, Hagberg JM, Ehsani AA, Holloszy JO. Improvement in glucose tolerance after 1 wk of exercise in patients with mild NIDDM. Diabetes Care. 1988;11(8):613–8. https://doi.org/10.2337/diacare.11.8.613.
73. Krotkiewski M, Lönnroth P, Mandroukas K, Wroblewski Z, Rebuffe-Scrive M, Holm G, Smith U, Björntorp P. The effects of physical training on insulin secretion and effectiveness and on glucose metabolism in obesity and type 2 (non-insulin-dependent) diabetes mellitus. Diabetologia. 1985;28(12):881–90. https://doi.org/10.1007/BF00703130.
74. Eriksen L, Dahl-Petersen I, Haugaard SB, dela F. Comparison of the effect of multiple short-duration with single long-duration exercise sessions on glucose homeostasis in type 2 diabetes mellitus. Diabetologia. 2007;50(11):2245–53. https://doi.org/10.1007/s00125-007-0783-0.
75. Hannon TS, Kahn SE, Utzschneider KM, Buchanan TA, Nadeau KJ, Zeitler PS, Ehrmann DA, Arslanian SA, Caprio S, Edelstein SL, Savage PJ, Mather KJ, for the RISE Consortium. Review of methods for measuring beta-cell function: design considerations from the restoring insulin secretion (RISE) consortium. Diabetes Obes Metab. 2018;20(1):14–24. https://doi.org/10.1111/dom.13005.
76. Malin SK, Solomon TPJ, Blaszczak A, Finnegan S, Filion J, Kirwan JP. Pancreatic beta-cell function increases in a linear dose-response manner following exercise training in adults with prediabetes. Am J Physiol Endocrinol Metab. 2013;305(10):E1248–54. https://doi.org/10.1152/ajpendo.00260.2013.
77. Sigal RJ, Kenny GP, Boulé NG, Wells GA, Prud'homme D, Fortier M, Reid RD, Tulloch H, Coyle D, Phillips P, Jennings A, Jaffey J. Effects of aerobic training, resistance training, or both on glycemic control in type 2 diabetes: a randomized trial. Ann Intern Med. 2007;147(6):357–69. https://doi.org/10.7326/0003-4819-147-6-200709180-00005.
78. Church TS, Blair SN, Cocreham S, Johannsen N, Johnson W, Kramer K, Mikus CR, Myers V, Nauta M, Rodarte RQ, Sparks L, Thompson A, Earnest CP. Effects of aerobic and resistance training on hemoglobin A1c levels in patients with type 2 diabetes: a randomized controlled trial. JAMA. 2010;304(20):2253–62. https://doi.org/10.1001/jama.2010.1710.

79. Group, A.C, et al. Intensive blood glucose control and vascular outcomes in patients with type 2 diabetes. N Engl J Med. 2008;358(24):2560–72.
80. Duckworth W, Abraira C, Moritz T, Reda D, Emanuele N, Reaven PD, Zieve FJ, Marks J, Davis SN, Hayward R, Warren SR, Goldman S, McCarren M, Vitek ME, Henderson WG, Huang GD. Glucose control and vascular complications in veterans with type 2 diabetes. N Engl J Med. 2009;360(2):129–39. https://doi.org/10.1056/NEJMoa0808431.
81. Reusch JE, Manson JE. Management of Type 2 diabetes in 2017: getting to goal. JAMA. 2017;317(10):1015–6. https://doi.org/10.1001/jama.2017.0241.
82. Hemmingsen B, Lund SS, Gluud C, Vaag A, Almdal T, Hemmingsen C, Wetterslev J. Intensive glycaemic control for patients with type 2 diabetes: systematic review with meta-analysis and trial sequential analysis of randomised clinical trials. BMJ. 2011;343(nov24 1):d6898. https://doi.org/10.1136/bmj.d6898.
83. UK Prospective Diabetes Study (UKPDS) Group Intensive blood-glucose control with sulphonylureas or insulin compared with conventional treatment and risk of complications in patients with type 2 diabetes (UKPDS 33). UK Prospective Diabetes Study (UKPDS) Group. Lancet. 1998;352(9131): 837–53.
84. Lipska KJ, Krumholz HM. Is hemoglobin A1c the right outcome for studies of diabetes? JAMA. 2017;317(10):1017–8. https://doi.org/10.1001/jama.2017.0029.
85. Solomon TP, et al. Pancreatic beta-cell function is a stronger predictor of changes in glycemic control after an aerobic exercise intervention than insulin sensitivity. J Clin Endocrinol Metab. 2013;98(10):4176–86. https://doi.org/10.1210/jc.2013-2232.
86. Solomon TP, et al. The influence of hyperglycemia on the therapeutic effect of exercise on glycemic control in patients with type 2 diabetes mellitus. JAMA Intern Med. 2013;173(19):1834–6. https://doi.org/10.1001/jamainternmed.2013.7783.
87. Leslie WS, Ford I, Sattar N, Hollingsworth KG, Adamson A, Sniehotta FF, McCombie L, Brosnahan N, Ross H, Mathers JC, Peters C, Thom G, Barnes A, Kean S, McIlvenna Y, Rodrigues A, Rehackova L, Zhyzhneuskaya S, Taylor R, Lean MEJ. The diabetes remission clinical trial (DiRECT): protocol for a cluster randomised trial. BMC Fam Pract. 2016;17(1):20. https://doi.org/10.1186/s12875-016-0406-2.
88. Ryan DH, et al. Look AHEAD (action for health in diabetes): design and methods for a clinical trial of weight loss for the prevention of cardiovascular disease in type 2 diabetes. Control Clin Trials. 2003;24(5):610–28.
89. Ried-Larsen M, Christensen R, Hansen KB, Johansen MY, Pedersen M, Zacho M, Hansen LS, Kofoed K, Thomsen K, Jensen MS, Nielsen RO, MacDonald C, Langberg H, Vaag AA, Pedersen BK, Karstoft K. Head-to-head comparison of intensive lifestyle intervention (U-TURN) versus conventional multifactorial care in patients with type 2 diabetes: protocol and rationale for an assessor-blinded, parallel group and randomised trial. BMJ Open. 2015;5(12):e009764. https://doi.org/10.1136/bmjopen-2015-009764.
90. Johansen MY, et al. Effect of an intensive lifestyle intervention on glycemic control in patients with type 2 diabetes: the U-TURN randomized clinical trial. JAMA. 2017;318(7):637–46. https://doi.org/10.1001/jama.2017.10169.
91. Sievenpiper JL, Dworatzek PD. Food and dietary pattern-based recommendations: an emerging approach to clinical practice guidelines for nutrition therapy in diabetes. Can J Diabetes. 2013;37(1):51–7. https://doi.org/10.1016/j.jcjd.2012.11.001.
92. Thomas D, Elliott EJ. Low glycaemic index, or low glycaemic load, diets for diabetes mellitus. Cochrane Database Syst Rev. 2009;1:CD006296.
93. Thomas DE, Elliott EJ. The use of low-glycaemic index diets in diabetes control. Br J Nutr. 2010;104(6):797–802. https://doi.org/10.1017/S0007114510001534.
94. Jenkins DJ, et al. Effect of legumes as part of a low glycemic index diet on glycemic control and cardiovascular risk factors in type 2 diabetes mellitus: a randomized controlled trial. Arch Intern Med. 2012;172(21):1653–60. https://doi.org/10.1001/2013.jamainternmed.70.
95. Ajala O, English P, Pinkney J. Systematic review and meta-analysis of different dietary approaches to the management of type 2 diabetes. Am J Clin Nutr. 2013;97(3):505–16. https://doi.org/10.3945/ajcn.112.042457.
96. Ley SH, Hamdy O, Mohan V, Hu FB. Prevention and management of type 2 diabetes: dietary components and nutritional strategies. Lancet. 2014; 383(9933):1999–2007. https://doi.org/10.1016/S0140-6736(14)60613-9.
97. Evert AB, Boucher JL, Cypress M, Dunbar SA, Franz MJ, Mayer-Davis EJ, Neumiller JJ, Nwankwo R, Verdi CL, Urbanski P, Yancy WS. Nutrition therapy recommendations for the management of adults with diabetes. Diabetes Care. 2013;36(11):3821–42. https://doi.org/10.2337/dc13-2042.
98. Henry CJ. Basal metabolic rate studies in humans: measurement and development of new equations. Public Health Nutr. 2005;8(7A):1133–52. https://doi.org/10.1079/PHN2005801.
99. De Nardi AT, et al. High-intensity interval training versus continuous training on physiological and metabolic variables in prediabetes and type 2 diabetes: a meta-analysis. Diabetes Res Clin Pract. 2018;137:149–59. https://doi.org/10.1016/j.diabres.2017.12.017.
100. Jelleyman C, Yates T, O'Donovan G, Gray LJ, King JA, Khunti K, Davies MJ. The effects of high-intensity interval training on glucose regulation and insulin resistance: a meta-analysis. Obes Rev. 2015;16(11):942–61. https://doi.org/10.1111/obr.12317.
101. Liu Y, et al. Resistance exercise intensity is correlated with attenuation of HbA1c and insulin in patients with type 2 diabetes: a systematic review and meta-analysis. Int J Environ Res Public Health. 2019;16(1):140. https://doi.org/10.3390/ijerph16010140.
102. Helms ER, Cronin J, Storey A, Zourdos MC. Application of the repetitions in reserve-based rating of perceived exertion scale for resistance training. Strength Cond J. 2016;38(4):42–9. https://doi.org/10.1519/SSC.0000000000000218.
103. Zourdos MC. Novel resistance training–specific rating of perceived exertion scale measuring repetitions in reserve. J Strength Cond Res. 2016;30(1):267–75. https://doi.org/10.1519/JSC.0000000000001049.
104. Karlsen T, Aamot IL, Haykowsky M, Rognmo Ø. High intensity interval training for maximizing health outcomes. Prog Cardiovasc Dis. 2017;60(1): 67–77. https://doi.org/10.1016/j.pcad.2017.03.006.
105. Bueno AM, Pilgaard M, Hulme A, Forsberg P, Ramskov D, Damsted C, Nielsen RO. Injury prevalence across sports: a descriptive analysis on a representative sample of the Danish population. Inj Epidemiol. 2018;5(1):6. https://doi.org/10.1186/s40621-018-0136-0.
106. Rasmussen ST, Andersen JT, Nielsen TK, Cejvanovic V, Petersen KM, Henriksen T, Weimann A, Lykkesfeldt J, Poulsen HE. Simvastatin and oxidative stress in humans: a randomized, double-blinded, placebo-controlled clinical trial. Redox Biol. 2016;9:32–8. https://doi.org/10.1016/j.redox.2016.05.007.
107. Medhus AW, et al. Delay of gastric emptying by duodenal intubation: sensitive measurement of gastric emptying by the paracetamol absorption test. Aliment Pharmacol Ther. 1999;13(5):609–20. https://doi.org/10.1046/j.1365-2036.1999.00519.x.
108. Hannukainen JC, Borra R, Linderborg K, Kallio H, Kiss J, Lepomäki V, Kalliokoski KK, Kujala UM, Kaprio J, Heinonen OJ, Komu M, Parkkola R, Ahotupa M, Lehtimäki T, Huupponen R, Iozzo P, Nuutila P. Liver and pancreatic fat content and metabolism in healthy monozygotic twins with discordant physical activity. J Hepatol. 2011;54(3):545–52. https://doi.org/10.1016/j.jhep.2010.07.029.
109. American Thoracic Society; American College of Chest PhysiciansATS/ACCP Statement on Cardiopulmonary Exercise Testing. Am J Respir Crit Care Med. 2003;167(2):211–77.
110. Hackett DA, Johnson NA, Halaki M, Chow CM. A novel scale to assess resistance-exercise effort. J Sports Sci. 2012;30(13):1405–13. https://doi.org/10.1080/02640414.2012.710757.
111. McHorney CA, Ware JE Jr, Raczek AE. The MOS 36-item short-form health survey (SF-36): II. Psychometric and clinical tests of validity in measuring physical and mental health constructs. Med Care. 1993;31(3):247–63. https://doi.org/10.1097/00005650-199303000-00006.
112. White IR, Horton NJ, Carpenter J, Pocock SJ. Strategy for intention to treat analysis in randomised trials with missing outcome data. BMJ. 2011; 342(feb07 1):d40. https://doi.org/10.1136/bmj.d40.
113. Detry MA, Lewis RJ. The intention-to-treat principle: how to assess the true effect of choosing a medical treatment. JAMA. 2014;312(1):85–6. https://doi.org/10.1001/jama.2014.7523.
114. Atlas Collaboration. Reconstruction of hadronic decay products of tau leptons with the ATLAS experiment. Eur Phys J C Part Fields. 2016; 76(5):295.
115. Dmitrienko A, D'Agostino RB Sr. Multiplicity considerations in clinical trials. N Engl J Med. 2018;378(22):2115–22. https://doi.org/10.1056/NEJMra1709701.
116. Clinical Safety Data Management: Definitions and Standards for Expedited Reporting. European Medicines Agency. https://www.ema.europa.eu/en/documents/scientific-guideline/international-conference-harmonisation-technical-requirements-registration-pharmaceuticals-human-use_en-15.pdf. Date for coming into operation June 1995 https://www.ema.europa.eu/en/documents/scientific-guideline/international-conference-harmonisation-

technical-requirements-registration-pharmaceuticals-human-use_en-15.pdf. Accessed 16 Mar 2021.
117. Christensen RH, Wedell-Neergaard AS, Lehrskov LL, Legård GE, Dorph EB, Nymand S, Ball MK, Zacho M, Christensen R, Ellingsgaard H, Rosenmeier JB, Krogh-Madsen R, Pedersen BK, Karstoft K. The role of exercise combined with tocilizumab in visceral and epicardial adipose tissue and gastric emptying rate in abdominally obese participants: protocol for a randomised controlled trial. Trials. 2018;19(1):266. https://doi.org/10.1186/s13063-018-2637-0.
118. Johansen MY, MacDonald CS, Hansen KB, Karstoft K, Christensen R, Pedersen M, Hansen LS, Zacho M, Wedell-Neergaard AS, Nielsen ST, Iepsen UW, Langberg H, Vaag AA, Pedersen BK, Ried-Larsen M. Effect of an intensive lifestyle intervention on glycemic control in patients with type 2 diabetes: a randomized clinical trial. JAMA. 2017;318(7):637–46. https://doi.org/10.1001/jama.2017.10169.
119. Snorgaard O, et al., Farmakologisk behandling af type 2-diabetes. http://www.endocrinology.dk/PDF/FarmakologiskbehandlingDM2rev2014.pdf. Dansk Endokrinologisk Selskab og Dansk Selskab for Almen Medicin. 2014. Accessed 16 Mar 2021.
120. Schulz KF, Altman DG, Moher D, for the CONSORT Group. CONSORT 2010 statement: updated guidelines for reporting parallel group randomised trials. BMJ. 2010;340(mar23 1):c332. https://doi.org/10.1136/bmj.c332.
121. Recommendations for the Conduct, Reporting, Editing, and Publication of Scholarly work in Medical Journals. http://www.icmje.org/icmje-recommendations.pdf ICMJ. Updated 2019. Accessed 16 Mar 2021.

Publisher's Note

Springer Nature remains neutral with regard to jurisdictional claims in published maps and institutional affiliations.

Ready to submit your research? Choose BMC and benefit from:

- fast, convenient online submission
- thorough peer review by experienced researchers in your field
- rapid publication on acceptance
- support for research data, including large and complex data types
- gold Open Access which fosters wider collaboration and increased citations
- maximum visibility for your research: over 100M website views per year

At BMC, research is always in progress.

Learn more biomedcentral.com/submissions

BMC

Statistical analysis plan (SAP)

The effects of different doses of exercise on pancreatic β-cell function in patients with newly diagnosed type 2 diabetes

Version:	1.0
Version date:	Nov. 12th 2021
Trial registration:	www.clinicaltrials.gov
Trial registration number:	NCT03769883
Ethical committee:	Capital Region of Denmark
Approval number:	H-18038298
SAP authors:	Mathias Ried-Larsen, Grit E. Legaard, Mark P. P. Lyngbæk
Sponsor - Investigator:	Mathias Ried-Larsen, Senior Researcher, Centre for Physical Activity Research, Dept. 7641, Rigshospitalet, Denmark
Investigators:	Grit Elster Legaard, MD and Mark Preben Printz Lyngbæk, MD, Centre for Physical Activity Research, Dept. 7641, Rigshospitalet, Denmark
Statistical advisor:	Robin Christensen, MSc, PhD; Professor of Biostatistics and Clinical Epidemiology; Head of Musculoskeletal Statistics Unit.

Table of Contents

BACKGROUND AND RATIONAL....3

OBJECTIVES....4

HYPOTHESIS....5

TRIAL DESIGN, DATA COLLECTION AND OUTCOMES ASSESSMENT....6

OUTCOMES....6

STUDY POPULATION, ANALYSIS SET AND STATISTICAL PRINCIPLES....9

STATISTICAL METHODS....11

DEVIATIONS FROM THE ORIGINAL PROTOCOL....12

IMPLEMENTATION OF THE SAP....14

EXPECTED WRITING COMMITTEE....14

EXPECTED OUTLINE OF THE REPORT....15

OVERVIEW OF CONTENT (Unformatted tables with specific variables are placed at the end of the text)....15

TABLES (In paper)....15

FIGURES (In paper)....15

ONLINE ONLY (Tables)....16

ONLINE ONLY (Figures)....17

REFERENCES....19

UNFORMATTED TABLES WITH INTENDED CONTENT....21

BACKGROUND AND RATIONAL

The etiology, pathophysiology and treatment of type 2 diabetes (T2D) are undeniably multifactorial and the understanding of T2D is increasing rapidly, but reducing obesity remains essential to improve β-cell function. However, a residue β-cell capacity appears to be essential for remission emphasizing the need for lifestyle intervention early in the clinical management [1].

While exercise is less recognized as an efficient therapy for weight loss, dietary therapy is [2]. With the recent advantages in the role of very low-calorie diets on β-cell function [1, 3], it is important to study the role of exercise therapy in combination with dietary-induced weight loss to fully understand the implications for patient care. However, only a few studies have focused on the effects of exercise on pancreatic β-cell function in T2D and discrepancies regarding the effect exist [4-8]. The discrepancies may relate to the assessment of β-cell function [9], failure to correct for the change in peripheral insulin sensitivity, concomitant pharmacological therapy and the pre-trial insulin secretory capacity. Moreover, exercise intensity, volume and modality may play an essential role in the reduction of HbA1c [10-14]. Thus, current evidence suggests that physical activity may *directly* improve β-cell mass and β-cell function[15], and may also *indirectly* improve β-cell function and mass by inducing β-cell rest via reductions in systemic inflammation and metabolic stress (i.e. gluco- and lipotoxicity). However, evidence is limited from human studies investigating the relationship of exercise volume, intensity, frequency, and dose-dependency on β-cell function[15]. As a consequence, knowledge about the exercise training dose needed to reduce micro- and macrovascular complications in T2D is almost non-existing [12, 16-23]. As most clinical exercise interventions in T2D base their conclusions on HbA1c, the significance of exercise training in the clinical care of prevalent T2D is challenged [11, 23-25] and investigating β-cell function with different volumes of exercise in addition to a diet-induced weight loss is of clinical relevance. A full description of the rationale behind the study has been published elsewhere[26]. We propose that combining a moderate diet-induced weight loss with exercise training may dose-dependently improve pancreatic β-cell function.

OBJECTIVES

Primary aim: To investigate the effect of exercise training volume on pancreatic β-cell function after 16 weeks in patients with short standing T2D.
Secondary aims: To investigate the effect of exercise training volume on mechanisms underlying β–cell function.

Primary objective: To compare the effect of high (HED) *or* moderate (MED) volumes of exercise in combination with a dietary intervention, relative to the control (CON) *or* diet (DCON) comparator, on changes in the late-phase disposition index (DI) during the final 30 minutes of hyperglycemic phase of the hyperglycemic clamp from baseline to week 16, in patients with short standing T2D.

Major secondary objective: To compare the effect of high (HED) *or* moderate (MED) volumes of exercise in combination with a dietary intervention, relative to the control (CON) *or* diet (DCON) comparator, on changes in insulin secretion rate and insulin sensitivity derived from hyperglycemic clamp AND oral insulinogenic and insulin sensitivity index derived from the mixed meal tolerance test (MMTT) from baseline to week 16, in patients with short standing T2D.

Other objectives: To compare the effect of high (HED) *or* moderate (MED) volumes of exercise in combination with a dietary intervention, relative to the control (CON) *or* diet (DCON) comparator on changes in glucose disposal, postprandial glycemic control, GLP-1 and arginine stimulated insulin secretion, fasting blood glucose control, fasting blood lipids, blood pressure, physical function from baseline to week 16, in patients with short standing T2D.

HYPOTHESIS

Primary: The effect of exercise training on pancreatic β-cell function (assessed as late-phase disposition index) increases with increasing volumes of exercise in combination with a diet intervention across a 16-week intervention in patients with T2D of short duration. Specifically, it is expected that both moderate volume and high volumes of exercise in combination with a dietary intervention are superior to the control intervention in improving pancreatic β-cell function.

The hierarchy of the hypotheses and subsequent claims for the primary outcome are as follows;

1. High-volume exercise and diet group (HED) is superior to the control intervention (CON) in increasing the late phase disposition index from baseline (visit 1) to follow-up (visit 7). Superiority is claimed if the difference in change between the groups favours the HED group.

2. Medium-volume exercise and diet group (MED) is superior to the control intervention in increasing the late phase disposition index from baseline (visit 1) to follow-up (visit 7). Superiority is claimed if the difference in change between the groups favours the MED group.

3. The diet control group (DCON) is superior to the control intervention in increasing the late phase disposition index from baseline (visit 1) to follow-up (visit 7). Superiority is claimed if the difference in change between the groups favours the DCON group.

4. HED is superior to the DCON intervention in increasing the late phase disposition index from baseline (visit 1) to follow-up (visit 7). Superiority is claimed if the difference in change between the groups favours the HED group.

5. MED is superior to the DCON intervention in increasing the late phase disposition index from baseline (visit 1) to follow-up (visit 7). Superiority is claimed if the difference in change between the groups favours the MED group.

6. HED is superior to the MED intervention in increasing the late phase disposition index from baseline (visit 1) to follow-up (visit 7). Superiority is claimed if the difference in change between the groups favours the HED group.

TRIAL DESIGN, DATA COLLECTION AND OUTCOMES ASSESSMENT

All procedure and detailed information about the trial design, eligibility and methods, including a detailed description of the interventions has been published elsewhere [26]. Briefly, the study is a parallel-group, 4-arm assessor-blinded, randomised, clinical trial with 16 weeks of intervention. Participants are randomly allocated (1:1:1:1, stratified by sex) to four groups; 1) No intervention, 2) Dietary intervention, 3) Dietary intervention + moderate volume exercise (3 sessions/week), 4) Dietary intervention + high volume exercise (6 sessions/week). The study is registered at www.clinicaltrials.gov (NCT03769883) and approved by the Scientific Ethical Committee of the Capital Region of Denmark (approval number H-18038298) prior to commencement of any study procedures. Primary place of study execution and data collection is the Centre for Physical Activity Research (CFAS), Rigshospitalet, section 7641, Tagensvej 20, DK-2200 Copenhagen (visiting address); Blegdamsvej 9, DK-2100 Copenhagen (postal address), Telephone: (+45) 3545 7641.

It is expected that an exercise intervention will increase late-phase disposition index derived from a hyperglycemic clamp by 1.5 (au.) more than the control group, with a standard deviation of 1.5 (au.) of the change in the exercise and 1.0 (au.) in the control group[4]. For a contrast in a one-way ANOVA with four means (1.5, 1.0, 0.5, 0.0) and contrast coefficients (1, 0, 0, -1) using a two-sided significance level of 0.05, assuming an error standard deviation of 1.5 and a balanced design, a total sample size of 80 participants corresponds to an approximate statistical power of 87.7%. Thus, at least 20 participants are recruited per group.

OUTCOMES

The domains and measurements for this article as well the hierarchal structure of the hereof are based on the pre-specified designation located in the trial registration (published prior to recruitment initiation) and our published protocol published prior to last patient-last-visit (described in Table 5)[26].

Primary outcome (change, timeframe 0 to 16 weeks)

Domain: beta-cell function

Measurement: hyperglycemic clamp

- Late-phase disposition index during the last 30 minutes of the glucose infusion in the hyperglycemic clamp

Major outcomes (change, timeframe 0 to 16 weeks)

Domain: beta-cell function

Measurement: hyperglycemic clamp

- Late-phase insulin sensitivity index (mean Glucose infusion rate over last 30 min of the hyperglycemic clamp phase/ (mean insulin × glucose))
- Late-phase insulin secretion rate (mean deconvoluted C-peptide measurements over last 30 min of the hyperglycemic clamp phase/ mean glucose)

Domain: post-prandial glycemic control

Measurement: mixed meal tolerance test:

- Oral disposition index
- Oral insulin sensitivity index (Matsuda index)
- Oral insulin secretion index

Other secondary outcomes (change, timeframe 0 to 16 weeks).

Domain: β cell function

Measurement: hyperglycemic clamp

- GLP-1 stimulated insulin secretion rate and C-peptide
- Arginine stimulated insulin secretion rate and C-peptide
- First phase C-peptide and insulin secretion defined as the peak concentration during the initial 10 minutes of the hyperglycemic clamp
- Basal rate of glucose disappearance (R_d)
- Basal rate of endogenous glucose appearance (R_a)
- Rate of glucose disappearance (R_d) during steady-state hyperglycemia
- Rate of glucose endogenous appearance (R_a) during steady-state hyperglycemia

Domain: post-prandial glycemic control

Measurement: mixed meal tolerance test:

- iAUC of glucose, insulin, glucagon and C-peptide
- tAUC of glucose, insulin, glucagon and C-peptide
- Glucagon like peptide-1
- Gastric inhibitory peptide

- Gastric emptying

Domain: Body anthropometrics and composition

- Body weight
- Body mass index (BMI)

Domain: Clinical, functional markers of mechanism

Measurement: fasting blood samples (plasma)

- Glycated hemoglobin A1c
- Glucose
- C-peptide
- Insulin
- Triglyceride
- Low density lipoprotein

Domain: Blood pressure

Measurement: Home blood pressure monitoring

- Avg. home systolic blood pressure
- Avg. home diastolic blood pressure

Domain: Physical function

Measurements: VO2max test (indirect calorimetry and 1 repetition max in chest press and leg-extensions)

- Maximal oxygen consumption
- Maximal oxygen consumption relative to body weight
- Upper and lower body maximal strength

STUDY POPULATION, ANALYSIS SET AND STATISTICAL PRINCIPLES

Inclusion and exclusion criteria have been published elsewhere [26].The primary analysis will be based on the family of the intention-to-treat population, defined as the *as-observed population* (missing data will not be imputed in the primary analysis) [27, 28], and the set of participants who are as close as possible to the intended intervention protocol, i.e. per-protocol (criteria described in the box 1) as a sensitivity analysis. The 'Full Analysis Set' for the intention-to-treat will thus be derived from the set of all randomized participants by minimal and justified elimination of participants. Therefore, all participants allocated to a treatment group (CON, DCON, MED or HED) will be followed up, assessed and analysed as members of that group irrespective of their compliance to the planned course of treatment.

P-values and 95% confidence intervals will be presented for the between-group difference in change comparisons and only 95% confidence intervals will be presented for the within-group (0-16 weeks) differences. Statistical significance will be claimed if the null hypothesis is rejected at the alpha level of 0.05 (two-sided). No corrections for multiplicity will be performed. To maintain the family-wise type 1 error rate on the primary outcome, a hierarchical analytic approach is engaged [29]; if we fail to progress from any of the subsequent steps ($p > 0.05$) we will interpret p-values and CI's numerically as indicators of associations.

Between group comparisons for effect size estimation (difference in change from 0-16 weeks, based on a superiority assumption) will be completed for all outcomes in the following order:

1) CON vs. HED. If a difference is present ($p < 0.05$, 2-sided) then the next between group comparison is performed. If not – then sequence is terminated.
2) CON vs. MED. If a difference is present ($p < 0.05$, 2-sided) then the next between group comparison is performed. If not – then sequence is terminated.
3) CON vs. DCON. If a difference is present ($p < 0.05$, 2-sided) then the next between group comparison is performed. If not – then sequence is terminated.
4) DCON vs. HED. If a difference is present ($p < 0.05$, 2-sided) then the next between group comparison is performed. If not – then sequence is terminated.
5) DCON vs. MED. If a difference is present ($p < 0.05$, 2-sided) then the next between group comparison is performed. If not – then sequence is terminated.
6) MED vs. HED.

All non-hypothesis-based comparisons (i.e. on the secondary outcomes) are per definition considered exploratory and supportive to the interpretation of the primary outcome.

BOX 1 Per-protocol definition (all criteria present)

Control group:

- The primary outcome is assessed at both baseline and after 16 weeks follow-up (i.e. complete case).

Diet control group:

- The primary outcome is assessed at both baseline and after 16 weeks follow-up (i.e. complete case).
- Do not exceed +/- 30% of the prescribed energy intake as assessed by their dietary records (assessed as the mean energy intake across that latter 16 weeks, excluding 1-week vacation administered following week 2 of the intervention)

Exercise and diet groups:

- The primary outcome is assessed at both baseline and after 16 weeks follow-up (i.e. complete case).
- ≥ 70% of the prescribed exercise volume across the intervention period (excluding the initial two weeks of familization and potentially one week of vacation permitted after week two of the intervention). Exercise volume is calculated separately for aerobic and resistance training and ≥ 70% of the volume of each type should be achieved. For aerobic training ≥ 70% of prescribed training time (minutes) should be within the target heart rate zones. For resistance training, ≥ 70% of prescribed sets should be performed at or below the prescribed maximum RIR.
- Do not exceed +/- 30% of the prescribed energy intake as assessed by their dietary records (assessed as the mean energy intake across that latter 16 weeks, excluding 1-week vacation administered following week 2 of the intervention).

Harms and adverse events as defined in the protocol will be reported if the incidence is ≥ 5 % in any of the groups. All serious adverse events will be reported. Harms and adverse events will be reported as number and percentages of participants experiencing the event by system organ class and will be subject to null-hypothesis testing.

Sensitivity analyses will be performed using the potentially biased but conservative non-responder imputation (*baseline observation carried forward* technique) as well as the current best practice multiple imputation procedure[27]. Patterns of missing data will be investigated. *A priori*, the less restrictive missing at random (MAR) assumption is considered more reasonable than data missing completely at random (MCAR). Assuming that the data on potential dropouts are MAR, multiple imputation procedures will be applicable to handle missing data for all participants with baseline measurements.

STATISTICAL METHODS

The analyses of the primary outcome will be performed using a repeated measures analysis of covariance applied using mixed linear modelling [28, 30]. Mean change score of DI will be applied as the dependent outcome variable, whereas group, time, the interaction between time and group, sex, the baseline value of DI are included as independent (fixed) variables and participant identifier as random effect. The potentially biased *per-protocol* population analysis will be adjusted for putative confounders: sex, age, diabetes duration, baseline maximal oxygen consumption. If the global test indicates between-group differences ($H_{0,DCON} = H_{0,MED} = H_{0,HED} = H_{0,CON}$; $p \leq 0.1$), pairwise between-group differences, in the order described above, will be explored. The same statistical method will be applied to the other continues outcomes.

The assumptions for using the linear models will be checked to confirm normal distribution of the residuals and the homogeneity of the variance (standardized residuals vs. the predicted values). Variables not meeting the model assumptions will be transformed using appropriate transformations. If no suitable transformation is identified, the median change with interquartile ranges will be reported and testing will be performed using suitable non-parametric statistical tests (e.g. quantile regression).

Dichotomous outcomes (i.e. discontinuation, reduction or intensification of medications according to the predefined treatment algorithm) at 16 weeks follow-up compared to baseline) will be analyzed using logistic regression. If the dichotomous outcome data are sparse, the asymptotic results can be unreliable; therefore, Fisher's exact tests will be used to calculate the exact probability of the possible (2×4) tables allowing estimation of the Wald-test-associated variance, which corresponds to the ratio of its estimate (log-odds ratio [OR]) to its standard error. By default, no imputations will be used (statistical or otherwise) for the analysis, but robustness will be assessed via sensitivity analyses which evaluate missing data to explore the effect of departures from the assumption made in the main analysis (missing at random).

Statistical code for the primary analysis

```
*********************************************************************
mixed dDI i.group##i.time DI_0 i.sex ||ID:,
contrast i.group##i.time (note: omnibus test)
pwcompare i.group##i.time (note: pairwise comparisons if the omnibus test allows)
*********************************************************************
```

The variable *dDI* is the change in late-phase disposition index from baseline (0 weeks) to end follow-up (16 weeks). *Group* is the treatment variable; *sex* describes the sex of the participants and *DI_0* is the baseline late-phase disposition index. The model includes treatment (group, 4 levels), time (2 levels), sex (2 levels), and the possible interaction between treatment and time (8 levels) as fixed effects, with the baseline value of the relevant variable as a covariate.

DEVIATIONS FROM THE ORIGINAL PROTOCOL

Due to new data on the effects of medical discontinuation, following protocol changes were made prior to initiation of the study (Date for amendment Dec. 18th, 2018);

- Complete medical discontinuation upon inclusion was abandoned and excluded from the *per-protocol definition*.

Due to a slow recruitment rate and exclusion of a clinically relevant group of potential participants, we modified the following eligibility criteria (Date for amendment Sep. 2nd, 2019);

- “No known lung disease” was changed to “No lung disease, other than asthma that can be managed with beta2-agonists and does not exhibit seasonal variation.
- “No known thyroid disease” was changed to “No changes in hypothyroid disease treatment within the last 3 three months prior to enrolment”
- “No known liver disease” was changed to “No known liver disease - defined as ALAT or ASAT elevated three times above upper limit.”
- “No known autoimmune disease” was changed to “No psoriasis disease requiring systemic treatment or cutaneous elements bigger than a total area of 25 cm^2”
- “No diagnose of depression or treatment with anti-depressive medication, ongoing or within the last three months before enrolment” was changed to “No changes in symptoms or anti-depressive medication three months prior to enrolment.”
- ” Protein or glucose in the urine at pre-screening” was changed to “Macroalbuminuria at pre-screening”
- “No biochemical sign of other major diseases” was changed to “Biochemical sign of other major diseases”

The majority of participants were not able to attend the 4- and 12-week visits following an overnight fast, thus fasting blood sampling at these timepoint were abandoned.

Change from 2-hour to 1-hour hyperglycemia + GLP-1 infusion as we were not able to maintain hyperglycemia and with excessive high coefficients of variation.

In the event of malfunctioning heart rate monitoring, the participant was carefully instructed to train in accordance to the Borg scale corresponding to target heart rate zones (i.e. %HRmax) in harmony with the specific training program[31].

Six participants have had their intervention prolonged 3 weeks in order to ensure that they were no longer infected or infectious. The participant group distribution consisted of 1 CON, 2 DCON, 2 MED and 1 HED.

IMPLEMENTATION OF THE SAP

Upon SAP approval by and signatures of the writing committee, the statistical analysis plan will published at The Centre for Physical Activity website (www.aktivsundhed.dk) prior to commencing any statistical analyses.

EXPECTED WRITING COMMITTEE

Grit E. Legaard*, Mark P.P. Lyngbæk*, Kristian Karstoft, Nina S. Nielsen, Camilla Feineis, Sebastian L. Bennetsen, Ulrikke Nystrup, Benedikte Liebetrau, Katja Thomsen, Jan Christian Brønd, Martin Østergaard, Beckey Trinh, Thomas Solomon, Gerrit Van Hall, Jens J. Holst, Bolette Hartmann, Robin Christensen, Thomas P. Almdal, Bente. K. Pedersen, Mathias Ried-Larsen

*The authors contributed equally to the work.

Acknowledgements
We would like to thank the current and former CFAS and Center for Diabetes Municipality of Copenhagen employees; Villads Rasmussen, Cecilie F. Brinkløv, Anette Blom Nielsen, Katja Kofoed, Nana Møgelberg, Indzi Hamidovski for the technical and administrative support in the data collection and delivering the intervention.

EXPECTED OUTLINE OF THE REPORT

The study report will be aimed at a clinical journal, thus the report will contain 3500-4000 words and 4 to 6 main figures and tables depending on the journal.

OVERVIEW OF CONTENT (Unformatted tables with specific variables are placed at the end of the text)

TABLES (In paper)

- Table 1 Baseline characteristics
- Table 2 Within group changes in the primary and major secondary outcomes
- Table 3 Pairwise comparisons of the change in the primary outcome and major secondary outcomes
- Table 4 Within group changes in other outcomes reflecting underlying mechanisms of β cell function
- Table 5 Pairwise comparisons of the change in other outcomes reflecting underlaying mechanisms of β cell function

FIGURES (In paper)

- Figure 1: Table of graphs (2x3 panel) depicting the within-group change (baseline to 16 weeks) in the primary outcome and major secondary outcomes. Data are presented as least-squares-means (bar charts overlaid with individual values) with 95% confidence intervals.
    - Figure 2a: Change in late phase disposition index (primary outcome) by group
    - Figure 2b: Change in late phase insulin sensitivity index by group
    - Figure 2c: Change in late phase insulin secretion rate by group
    - Figure 2d: Change in oral disposition index by group
    - Figure 2e: Change in oral insulin sensitivity index by group
    - Figure 2f: Change in oral insulinogenic index by group

ONLINE ONLY (Tables)

- eTable 1 Self-reported adherence to diet
- eTabel 2 Self-reported adherence to pharmacological treatment and management
- eTable 3 Free-living physical activity
- eTable 4 Intensity and duration in aerobic training. Intensity measured as %HRmax in intervention in MED and HED group. Intensity is reported as duration in moderate intensity (60-79% HRmax) and duration in high intensity (80-100% HRmax).
- eTable 5 Resistance training in the large muscle groups. Intensity measured as repetitions in reserve in resistance training intervention in MED and HED group.
- eTable 6 Volume load (tonnage) in resistance training in the large muscle groups. Volume load measured as tonnage (kg x repetitions x sets).
- eTable 7 Exercise modification and causes in aerobic training in MED and HED group.
- eTable 8 Exercise modification and causes in resistance training in MED and HED group.
- eTabel 9 Adherence for aerobic and resistance training in MED and HED group.
- eTable 10 Coefficient of variation and precision during the hyperglycemic clamp
- eTable 11 Sensitivity analyses - Pairwise comparisons of the change in the primary and major outcomes
- eTable 12 Baseline values and within group changes (0-16 weeks) in the primary outcome and other secondary outcomes derived from the hyperglycemic clamp
- eTable 13 Other Pairwise comparisons of secondary outcomes derived from the mixed meal tolerance test
- eTable 14 Baseline values and within group changes (0-16 weeks) cardiometabolic, body composition and fitness
- eTable 15 Pairwise comparisons of the change in cardiometabolic, body composition and fitness
- eTable 16 Adverse events after randomization

ONLINE ONLY (Figures)

- eFigure 1: Flow of participants

- eFigure 2: Figure describing the pre-defined algorithms for pharmacological management of blood glucose, blood pressure and blood lipids including therapeutic targets.

- eFigure 3: Table of graphs describing the insulin secretion rates (least-squares-means, concentration on the y-axis) across the clamp (time in minutes on x-axis) by group (1a, 1b, 1c, 1d) with pre and post values and standard errors in the same graph (overlay graphs)

- eFigure 4: Table of graphs describing the glucose infusion rates (least-squares-means, concentration on the y-axis) across the clamp (time in minutes on x-axis) by group (1a, 1b, 1c, 1d) with pre and post values and standard errors in the same graph (overlay graphs)

- eFigure 5: Table of graphs describing the GLP-1 (least-squares-means, concentration on the y-axis) across the hyperglycemic clamp (time in minutes on x-axis) by group (1a, 1b, 1c, 1d) with pre and post values and standard errors in the same graph (overlay graphs)

- eFigure 6: Table of graphs describing the glucose (least-squares-means, concentration on the y-axis) response during the mixed meal tolerance test (time in minutes on x-axis) by group (1a, 1b, 1c, 1d) with pre and post values and standard errors in the same graph (overlay graphs)

- eFigure 7: Table of graphs describing the insulin (least-squares-means, concentration on the y-axis) response during the mixed meal tolerance test (time in minutes on x-axis) by group (1a, 1b, 1c, 1d) with pre and post values and standard errors in the same graph (overlay graphs)

- eFigure 8: Table of graphs describing the c-peptide (least-squares-means, concentration on the y-axis) response during the mixed meal tolerance test (time in minutes on x-axis) by group (1a, 1b, 1c, 1d) with pre and post values and standard errors in the same graph (overlay graphs)

- eFigure 9: Table of graphs describing the GLP-1 (least-squares-means, concentration on the y-axis) response during the mixed meal tolerance test (time in minutes on x-axis) by group (1a, 1b, 1c, 1d) with pre and post values and standard errors in the same graph (overlay graphs)

- eFigure 10: Table of graphs describing the GIP (least-squares-means, concentration on the y-axis) response during the mixed meal tolerance test (time in minutes on x-axis) by group (1a, 1b, 1c, 1d) with pre and post values and standard errors in the same graph (overlay graphs)

- eFigure 11: Table of graphs describing the paracetamol (least-squares-means, concentration on the y-axis) response during the mixed meal tolerance test (time in minutes on x-axis) by group (1a, 1b, 1c, 1d) with pre and post values and standard errors in the same graph (overlay graphs)

REFERENCES

1. Taylor, R., et al., *Remission of Human Type 2 Diabetes Requires Decrease in Liver and Pancreas Fat Content but Is Dependent upon Capacity for beta Cell Recovery.* Cell Metab, 2018. **28**(4): p. 547-556 e3.
2. Johansson, K., M. Neovius, and E. Hemmingsson, *Effects of anti-obesity drugs, diet, and exercise on weight-loss maintenance after a very-low-calorie diet or low-calorie diet: a systematic review and meta-analysis of randomized controlled trials.* The American Journal of Clinical Nutrition, 2013. **99**(1): p. 14-23.
3. Lean, M.E.J., et al., *Durability of a primary care-led weight-management intervention for remission of type 2 diabetes: 2-year results of the DiRECT open-label, cluster-randomised trial.* Lancet Diabetes Endocrinol, 2019. **7**(5): p. 344-355.
4. Karstoft, K., et al., *Mechanisms behind the superior effects of interval vs continuous training on glycaemic control in individuals with type 2 diabetes: a randomised controlled trial.* Diabetologia, 2014. **57**(10): p. 2081-93.
5. Dela, F., et al., *Physical training may enhance beta-cell function in type 2 diabetes.* Am J Physiol Endocrinol Metab, 2004. **287**(5): p. E1024-31.
6. Rogers, M.A., et al., *Improvement in glucose tolerance after 1 wk of exercise in patients with mild NIDDM.* Diabetes Care, 1988. **11**(8): p. 613-8.
7. Krotkiewski, M., et al., *The effects of physical training on insulin secretion and effectiveness and on glucose metabolism in obesity and type 2 (non-insulin-dependent) diabetes mellitus.* Diabetologia, 1985. **28**(12): p. 881-90.
8. Eriksen, L., et al., *Comparison of the effect of multiple short-duration with single long-duration exercise sessions on glucose homeostasis in type 2 diabetes mellitus.* Diabetologia, 2007. **50**(11): p. 2245-53.
9. Hannon, T.S., et al., *Review of methods for measuring beta-cell function: Design considerations from the Restoring Insulin Secretion (RISE) Consortium.* Diabetes Obes Metab, 2018. **20**(1): p. 14-24.
10. Malin, S.K., et al., *Pancreatic beta-cell function increases in a linear dose-response manner following exercise training in adults with prediabetes.* Am J Physiol Endocrinol Metab, 2013. **305**(10): p. E1248-54.
11. Umpierre, D., et al., *Volume of supervised exercise training impacts glycaemic control in patients with type 2 diabetes: a systematic review with meta-regression analysis.* Diabetologia, 2013. **56**(2): p. 242-51.
12. Boule, N.G., et al., *Meta-analysis of the effect of structured exercise training on cardiorespiratory fitness in Type 2 diabetes mellitus.* Diabetologia, 2003. **46**(8): p. 1071-81.
13. Sigal, R.J., et al., *Effects of aerobic training, resistance training, or both on glycemic control in type 2 diabetes: a randomized trial.* Ann Intern Med, 2007. **147**(6): p. 357-69.
14. Church, T.S., et al., *Effects of aerobic and resistance training on hemoglobin A1c levels in patients with type 2 diabetes: a randomized controlled trial.* JAMA, 2010. **304**(20): p. 2253-62.

15. Curran, M., et al., *The benefits of physical exercise for the health of the pancreatic beta-cell: a review of the evidence.* Exp Physiol, 2020.
16. Action to Control Cardiovascular Risk in Diabetes Study, G., et al., *Effects of intensive glucose lowering in type 2 diabetes.* N Engl J Med, 2008. **358**(24): p. 2545-59.
17. Group, A.C., et al., *Intensive blood glucose control and vascular outcomes in patients with type 2 diabetes.* N Engl J Med, 2008. **358**(24): p. 2560-72.
18. Duckworth, W., et al., *Glucose control and vascular complications in veterans with type 2 diabetes.* N Engl J Med, 2009. **360**(2): p. 129-39.
19. Reusch, J.E. and J.E. Manson, *Management of Type 2 Diabetes in 2017: Getting to Goal.* JAMA, 2017. **317**(10): p. 1015-1016.
20. Hemmingsen, B., et al., *Intensive glycaemic control for patients with type 2 diabetes: systematic review with meta-analysis and trial sequential analysis of randomised clinical trials.* BMJ, 2011. **343**: p. d6898.
21. *Intensive blood-glucose control with sulphonylureas or insulin compared with conventional treatment and risk of complications in patients with type 2 diabetes (UKPDS 33). UK Prospective Diabetes Study (UKPDS) Group.* Lancet, 1998. **352**(9131): p. 837-53.
22. Lipska, K.J. and H.M. Krumholz, *Is Hemoglobin A1c the Right Outcome for Studies of Diabetes?* JAMA, 2017. **317**(10): p. 1017-1018.
23. Umpierre, D., et al., *Physical activity advice only or structured exercise training and association with HbA1c levels in type 2 diabetes: a systematic review and meta-analysis.* JAMA, 2011. **305**(17): p. 1790-9.
24. Avery, L., et al., *Changing physical activity behavior in type 2 diabetes: a systematic review and meta-analysis of behavioral interventions.* Diabetes Care, 2012. **35**(12): p. 2681-9.
25. Boule, N.G., et al., *Effects of exercise on glycemic control and body mass in type 2 diabetes mellitus: a meta-analysis of controlled clinical trials.* JAMA, 2001. **286**(10): p. 1218-27.
26. Lyngbaek, M.P.P., et al., *The effects of different doses of exercise on pancreatic beta-cell function in patients with newly diagnosed type 2 diabetes: study protocol for and rationale behind the "DOSE-EX" multi-arm parallel-group randomised clinical trial.* Trials, 2021. **22**(1): p. 244.
27. White, I.R., et al., *Strategy for intention to treat analysis in randomised trials with missing outcome data.* BMJ, 2011. **342**: p. d40.
28. Detry, M.A. and R.J. Lewis, *The intention-to-treat principle: how to assess the true effect of choosing a medical treatment.* JAMA, 2014. **312**(1): p. 85-6.
29. Dmitrienko, A. and R.B. D'Agostino, Sr., *Multiplicity Considerations in Clinical Trials.* N Engl J Med, 2018. **378**(22): p. 2115-2122.
30. Atlas, C., et al., *Reconstruction of hadronic decay products of tau leptons with the ATLAS experiment.* Eur Phys J C Part Fields, 2016. **76**(5): p. 295.
31. Scherr, J., et al., *Associations between Borg's rating of perceived exertion and physiological measures of exercise intensity.* Eur J Appl Physiol, 2013. **113**(1): p. 147-55.

UNFORMATTED TABLES WITH INTENDED CONTENT

Table 1 Baseline characteristics

	CON	DCON	MED	HED	Total
Age (years)					
Sex (N (%) female)					
Type 2 diabetes duration (years)					
Glycemic control					
HbA1c (mmol/mol)					
HbA1c (%)					
Fasting glucose (mmol/l)					
Fasting insulin (pmol/l)					
Fasting C-peptide (pmol/l)					
Lipids					
Low Density lipoprotein					
Fasting triglycerides					
Blood pressure					
Systolic (mmHg)					
Diastolic (mmHg)					
Glucose-lowering medication, N (%)					
None					
Biguanide					
Biguanide + SGLT2i **or** DPP4i					
Biguanide + SGLT2i + DPP4i					
Lipid-lowering medication, No (%)					
None					
Statin					
Blood pressure lowering medication, No (%)					
None					
ARB **or** ACEi					
ARB **or** ACEi + Thiazide **or** CCB					
ARB **or** ACEi + Thiazide + CCB					
Physical function					
Absolute VO_2 max (ml/min)					
Relative VO_2 max (ml/kg/min)					
Watt max (W/kg)					
1 RM chest press (kg)					
1 RM leg extension (kg)					
Body composition					
Body weight (kg)					
BMI (kg/m^2)					
Diet					
Energy intake (kcal/day)					
Physical activity level					
Moderate and vigorous physical activity (hours/day)					
Stepping (steps/day)					
Sitting (hours/day)					
Hyperglycemic clamp					
Basal					
Mean insulin secretion rate					
Glucose R_a (mg * kg^{-1} * min^{-1})					
Glucose R_d (mg * kg^{-1} * min^{-1})					
Early phase hyperglycemia					
Mean GIR (mg * kg^{-1} * min^{-1})					
Mean insulin secretion rate					
Peak insulin secretion rate					

**Table 1 cont’d**					
Steady state hyperglycemia					
Late phase disposition index					
Late phase insulin sensitivity index					
Late phase insulin secretion rate					
Mean GIR ($mg * kg^{-1} * min^{-1}$)					
Peak insulin secretion rate					
Glucose R_a ($mg * kg^{-1} * min^{-1}$)					
Glucose R_d ($mg * kg^{-1} * min^{-1}$)					
Hyperglycemia and GLP-1					
Mean GIR ($mg * kg^{-1} * min^{-1}$)					
Mean insulin secretion rate					
Peak insulin secretion rate					
Hyperglycemia, GLP-1 and Arginine					
Mean insulin secretion rate					
Peak insulin secretion rate					
Mixed meal tolerance test					
0-30 min					
tAUC glucose					
tAUC C-peptide					
tAUC insulin					
tAUC $GLP\text{-}1_{total}$					
tAUC $GLP\text{-}1_{active}$					
tAUC GIP_{total}					
tAUC paracetamol					
0-120 min					
Oral disposition index					
Oral insulin sensitivity index					
tAUC glucose					
tAUC C-peptide					
tAUC insulin					
tAUC $GLP\text{-}1_{total}$					
tAUC $GLP\text{-}1_{active}$					
tAUC GIP_{total}					
tAUC paracetamol					

Data are presented as mean (SD) or median (IQR). CON, control group, DCON: Diet control group: MED: Moderate volume exercise, HED: High volume exercise, HbA1c: glycated hemoglobin A1c, LDL: low-density lipoprotein, BMI: body mass index (calculated as weight in kilograms divided by height in meters squared). SLGT2i: selective sodium glucose co-transporter 2 inhibitors, DPP4i*:* dipeptidyl peptidase 4 inhibitors, ARB: angiotensin II receptor blockers, ACEi: angiotensin converting enzyme inhibitors, CCB: calcium channel blockers. Ra: Rate of appearance, Rd: Rate of disappearance, GIR: Glucose infusion rate

Table 2 Within-group changes from baseline to 16-week follow-up in the primary and major secondary outcomes								
	CON		DCON		MED		HED	
	Change	95% CI	Change	95% CI	Change	95% CI	Change	95% CI
Primary outcome								
Late-phase Disposition index								
Major Secondary outcomes								
Late-phase insulin secretion rate								
Late-phase insulin sensitivity								
Oral disposition index								
Oral insulin sensitivity index								
Oral insulinogenic index								

Data are least-squares means. CI: confidence intervals, CON: control group, DCON: Dietary control group, MED: Moderate volume exercise, HED: High volume exercise,

Table 3 Pairwise comparisons of the change in the primary outcome and major secondary outcomes

	HED vs. CON			MED vs. CON			DCON vs. CON			HED vs. DCON			MED vs. DCON			HED vs. MED		P
	MD	95% CI		MD	95% CI		MD	95% CI		MD	95% CI		MD	95% CI		MD	95% CI	
Primary outcome																		
Late-phase Disposition index																		
Major Secondary outcomes																		
Insulin secretion rate																		
Insulin sensitivity																		
Oral disposition index																		
Oral insulin sensitivity index																		
Oral insulinogenic index																		

MD: Mean difference, CI: confidence intervals. CON: control group, DCON: Dietary control group, MED: Moderate volume exercise, HED: High volume exercise

Table 4 Within-group changes from baseline to 16-week follow-up in in other outcomes reflecting underlaying mechanisms of beta-cell function

	CON		DCON		MED		HED	
	Change	95% CI	Change	95% CI	Change	95% CI	Change	95% CI
Basal								
Mean insulin secretion rate								
Glucose R_a (mg * kg^{-1} * min^{-1})								
Glucose R_d (mg * kg^{-1} * min^{-1})								
Early state hyperglycemia								
Mean GIR (mg * kg^{-1} * min^{-1})								
Mean insulin secretion rate								
Peak insulin secretion rate								
Steady state hyperglycemia								
Mean GIR (mg * kg^{-1} * min^{-1})								
Peak insulin secretion rate								
Glucose R_a (mg * kg^{-1} * min^{-1})								
Glucose R_d (mg * kg^{-1} * min^{-1})								
Hyperglycemia and GLP-1								
Mean GIR (mg * kg^{-1} * min^{-1})								
Mean insulin secretion rate								
Peak insulin secretion rate								
Hyperglycemia, GLP-1 and Arginine								
Mean insulin secretion rate								
Peak insulin secretion rate								
0-30 min								
tAUC glucose								
tAUC C-peptide								
tAUC insulin								
tAUC $GLP\text{-}1_{total}$								
tAUC $GLP\text{-}1_{active}$								
tAUC GIP_{total}								
tAUC paracetamol								
0-120 min								
tAUC glucose								
tAUC C-peptide								
tAUC insulin								
tAUC $GLP\text{-}1_{total}$								
tAUC $GLP\text{-}1_{active}$								
tAUC GIP_{total}								
tAUC paracetamol								

Data are least-squares means. CI: confidence intervals, CON: control group, DCON: Dietary control group, MED: Moderate volume exercise, HED: High volume exercise, GIR: glucose infusion rate, Ra: Rate of appearance, Rd: Rate of disappearance: GLP-1: Glucagon-like-peptide 1, GIP: Gastric inhibitory polypeptide, tAUC: Total area under the curve

Table 5 Pairwise comparisons of the change in other outcomes reflecting underlying mechanisms of beta-cell function

	HED vs. CON		MED vs. CON		DCON vs. CON		HED vs. DCON		MED vs. DCON		HED vs. MED	
	MD	95% CI	MD	95% CI	MD	95% CI	MD	95% CI	MD	95% CI	MD	95% CI
	Hyperglycemic clamp											
Basal												
Mean insulin secretion rate												
Glucose R_a ($mg * kg^{-1} * min^{-1}$)												
Glucose R_d ($mg * kg^{-1} * min^{-1}$)												
Early state hyperglycemia												
Mean GIR ($mg * kg^{-1} * min^{-1}$)												
Mean insulin secretion rate												
Peak insulin secretion rate												
Steady state hyperglycemia												
Mean GIR ($mg * kg^{-1} * min^{-1}$)												
Peak insulin secretion rate												
Glucose R_a ($mg * kg^{-1} * min^{-1}$)												
Glucose R_d ($mg * kg^{-1} * min^{-1}$)												
Hyperglycemia and GLP-1												
Mean GIR ($mg * kg^{-1} * min^{-1}$)												
Mean insulin secretion rate												
Peak insulin secretion rate												
Hyperglycemia, GLP-1 and Arginine												
Mean insulin secretion rate												
Peak insulin secretion rate												
	Mixed meal tolerance test											
0-30 min												
tAUC glucose												
tAUC C-peptide												
tAUC insulin												
tAUC $GLP\text{-}1_{total}$												
tAUC $GLP\text{-}1_{active}$												
tAUC GIP_{total}												
tAUC paracetamol												
0-120 min												
tAUC glucose												
tAUC C-peptide												
tAUC insulin												
tAUC $GLP\text{-}1_{total}$												
tAUC $GLP\text{-}1_{active}$												
tAUC GIP_{total}												
tAUC paracetamol												

MD: Mean difference, CI: confidence intervals. CON: control group, DCON: Dietary control group, MED: Moderate volume exercise, HED: High volume exercise, GIR: glucose infusion rate, Ra: Rate of appearance, Rd: Rate of disappearance: GLP-1: Glucagon-like-peptide 1, GIP: Gastric inhibitory polypeptide, tAUC: Total area under the curve

ONLINE ONLY

eTable 1 Adherence to diet									
	Baseline (N=)	Week 4 (N=)	% adherence	Week 12 (N=)	% adherence	Week 16 (N=)	% adherence	% adherence after randomization	Mean reduction after randomization (% from baseline)
Total energy intake (Kcal/kg/day)									
CON									
DCON									
MED									
HED									
Total carbohydrate (% of total energy intake)									
CON									
DCON									
MED									
HED									
Fiber (% of total energy intake)									
CON									
DCON									
MED									
HED									
Total fat (% of total energy intake)									
CON									
DCON									
MED									
HED									
Saturated fat (% of total energy intake)									
CON									
DCON									
MED									
HED									
Protein (% of total energy intake)									
CON									
DCON									
MED									
HED									
Alcohol (% of total energy intake)									
CON									
DCON									
MED									
HED									

Data are mean and standard deviation or median and interquartile range. CON: control group, DCON: Dietary control group, MED: Moderate volume exercise, HED: High volume exercise

eTabel 2 self-reported adherence to pharmacological treatment[a] and management																
	Baseline				Week 4				Week 12				Week 16			
	CON	DCON	MED	HED	CON	DCON	MED	HED	CON	DCON	MED	HED	CON	DCON	MED	HED
Proportion of participants attending consultation																
Self-reported adherence to Glucose-lowering medication																
Several times per week																
Once a week																
Several times per month																
Once a month																
Never																
Not relevant																
Does not take prescribed medication																
Missing values																
Self-reported adherence to blood pressure-lowering medication																
Several times per week																
Once a week																
Several times per month																
Once a month																
Never																
Not relevant																
Does not take prescribed medication																
Missing values																
Self-reported adherence to lipid-lowering medication																
Several times per week																
Once a week																
Several times per month																
Once a month																
Never																
Not relevant																
Does not take prescribed medication																
Missing values																
Glucose-lowering medication, N (%)																
None																
Biguanide																
Biguanide + SGLT2i **or** DPP4i																
Biguanide + SGLT2i **+** DPP4i																
Lipid-lowering medication, No (%)																
None																
Statin																
Blood pressure lowering medication, No (%)																
None																
ARB **or** ACEi																
ARB **or** ACEi + Thiazide **or** CCB																
ARB **or** ACEi + Thiazide + CCB																

Data presented as N (%)
There were five adherence categories in relation to how often the participants would forget to take their medicine: 1) several times per week 2) once a week 3) several times per month 4) once a month 5) never.
Adherence (%) in these categories is calculated as follows: Total N - (does not take the prescribed medicine + numbers of participants with no medication + missing values) since adherence is calculated based on the participants that are prescribed medication and taking the medication. Not the total number of participants (N).
[a]How often does the participant forget the medication

eTable 3 Free-living physical activity				
	Baseline (N=)	Week 4 (N=)	Week 12 (N=)	Week 16 (N=)
Valid days (N)				
CON				
DCON				
MED				
HED				
Wear time (hours/day)				
CON				
DCON				
MED				
HED				
Total physical activity (counts per minute)				
CON				
DCON				
MED				
HED				
MVPA (min/day)				
CON				
DCON				
MED				
HED				
Sitting time (min/day)				
CON				
DCON				
MED				
HED				
Stepping (steps/day)				
CON				
DCON				
MED				
HED				
Data are mean and standard deviation or median and interquartile range. CON: control group, DCON: Dietary control group, MED: Moderate volume exercise, HED: High volume exercise, MVPA: Moderate and vigorous physical activity				

eTable 4 Intensity and duration in aerobic training						
Intensity (internal and external load) in aerobic training						
Familiarization week 1-2	Average %HRmax, N (%)	Number of minutes 60-79% HRmax, (N=)	Number of minutes 80-100% HRmax, (N=)	Minutes spent in 80-100% HRmax, N (%)	Average watt, (N=)	
MED						
HED						
Week 3-10	Average %HRmax, N (%)	Number of minutes 60-79% HRmax, (N=)	Number of minutes 80-100% HRmax, (N=)	Minutes spent in 80-100% HRmax, N (%)	Average watt, (N=)	Increase in average watt from week 1-2 to 3-10 N (%)
MED						
HED						
Week 11-16	Average %HRmax, N (%)	Number of minutes 60-79% HRmax, (N=)	Number of minutes 80-100% HRmax, (N=)	Minutes spent in 80-100% HRmax, N (%)	Average watt, (N=)	Increase in average watt from week 3-10 to week 11-16 N (%)
MED						
HED						
Week 3-16	Number of minutes 60-79% HRmax, (N=)	Number of minutes 80-100% HRmax, (N=)	Number of minutes within target %HRmax, N (%)	Minutes spent in 80-100% HRmax, N (%)	Average watt, (N=)	Increase in average watt from week 3 to week 16, N (%)
MED						
HED						
Duration of aerobic training						
Familiarization week 1-2	Number of minutes prescribed pr. week, (N=)	Number of minutes performed pr week, (N=)	Number of minutes completed from prescribed, N (%)	Number of minutes performed within target %HRmax, N (%)	Number of minutes pr. sessions, (N=)	Number of sessions pr. week, (N=)
MED						
HED						
Week 3-10	Number of minutes prescribed pr. week, (N=)	Number of minutes performed pr week, (N=)	Number of minutes completed from prescribed, N (%)	Number of minutes performed within target %HRmax, N (%)	Number of minutes pr. sessions (N=)	Number of sessions pr. week, (N=)
MED						
HED						
Week 11-16	Number of minutes prescribed pr. week, (N=)	Number of minutes performed pr week, (N=)	Number of minutes completed from prescribed, N (%)	Number of minutes performed within target %HRmax, N (%)	Number of minutes pr. sessions (N=)	Number of sessions pr. week, (N=)
MED						
HED						
Week 3-16	Number of minutes prescribed pr. week, (N=)	Number of minutes performed pr week, (N=)	Number of minutes completed from prescribed, N (%)	Number of minutes performed within target %HRmax, N (%)	Number of minutes pr. sessions (N=)	Number of sessions pr. week, (N=)
MED						
HED						
Data are mean and standard deviation or median and interquartile range. HRmax: Maximum heart rate, MED: Moderate volume exercise, HED: High volume exercise						

eTable 5 Resistance training in the large muscle groups				
Familiarization week 1-2	Number of sets prescribed pr. week, (N=)	Number of sets performed pr. week, (N=)	Number of sets completed from prescribed, N (%)	Number of sets performed within target RIR, N (%)
MED				
Leg press				
Leg extension				
Leg curl				
Chest press				
Back row				
Total				
HED				
Leg press				
Leg extension				
Leg curl				
Chest press				
Back row				
Total				
Week 3-10	Number of sets prescribed pr. week, (N=)	Number of sets performed pr. week, (N=)	Number of sets completed from prescribed, N (%)	Number of sets performed within target RIR, N (%)
MED				
Leg press				
Leg extension				
Leg curl				
Chest press				
Back row				
Total				
HED				
Leg press				
Leg extension				
Leg curl				
Chest press				
Back row				
Total				
Week 11-16	Number of sets prescribed pr. week, (N=)	Number of sets performed pr. week, (N=)	Number of sets completed from prescribed, N (%)	Number of sets performed within target RIR, N (%)
MED				
Leg press				
Leg extension				
Leg curl				
Chest press				
Back row				
Total				
HED				
Leg press				
Leg extension				
Leg curl				
Chest press				
Back row				
Total				
Week 3-16	Number of sets prescribed pr. week, (N=)	Number of sets performed pr. week, (N=)	Number of sets completed from prescribed, N (%)	Number of sets performed within target RIR, N (%)
MED				
Leg press				
Leg extension				
Leg curl				
Chest press				
Back row				
Total				
HED				
Leg press				
Leg extension				
Leg curl				
Chest press				
Back row				
Total				
Data are mean and standard deviation or median and interquartile range. RIR: repetitions in reserve, MED: Moderate volume exercise, HED: High volume exercise				

eTable 6 Volume load (tonnage) in resistance training in the large muscle groups						
Familiarization week 1-2	Number of repetitions pr week, (N=)	Number of repetitions pr. set, (N=)	Average kilogram lifted pr. set, (N=)	Number of sets performed pr week, (N=)	Tonnage pr week, (N=)	
MED						
Leg press						
Leg extension						
Leg curl						
Chest press						
Back row						
Total						
HED						
Leg press						
Leg extension						
Leg curl						
Chest press						
Back row						
Total						
Week 3-10	Number of repetitions pr week, (N=)	Number of repetitions pr. set, (N=)	Average kilogram lifted pr. set, (N=)	Number of sets performed, (N=)	Tonnage pr week, (N=)	Tonnage increase from week 1-2 to week 3-10, N (%)
MED						
Leg press						
Leg extension						
Leg curl						
Chest press						
Back row						
Total						
HED						
Leg press						
Leg extension						
Leg curl						
Chest press						
Back row						
Total						
Week 11-16	Number of repetitions pr week, (N=)	Number of repetitions pr. set, (N=)	Average kilogram lifted pr. set, (N=)	Number of sets performed, (N=)	Tonnage pr week, (N=)	Tonnage increase from week 3-10 to week 11-16, N (%)
MED						
Leg press						
Leg extension						
Leg curl						
Chest press						
Back row						
Total						
HED						
Leg press						
Leg extension						
Leg curl						
Chest press						
Back row						
Total						
Week 3-16	Number of repetitions pr week, (N=)	Number of repetitions pr. set, (N=)	Average kilogram lifted pr. set, (N=)	Number of sets performed, (N=)	Tonnage, (N=)	Tonnage increase from week 3 to week 16, N (%)
MED						
Leg press						
Leg extension						
Leg curl						
Chest press						
Back row						
Total						
HED						
Leg press						
Leg extension						
Leg curl						
Chest press						
Back row						
Total						
Data are mean and standard deviation or median and interquartile range. Tonnage: weight (kg) x repetitions x sets, MED: Moderate volume exercise, HED: High volume exercise						

eTable 7 Exercise modification and causes in aerobic training							
Familiarization week 1-2	Fatigue, (N=)	Musculoskeletal discomfort, (N=)	Motivational, (N=)	Other reasons, (N=)	Missed exercises, (N=)	Number of participants with ≥1 modification (%)	Number of sessions with ≥1 modification (%)
MED							
HED							
Week 3-10	Fatigue, (N=)	Musculoskeletal discomfort, (N=)	Motivational, (N=)	Other reasons, (N=)	Missed exercises, (N=)	Number of participants with ≥1 modification (%)	Number of sessions with ≥1 modification (%)
MED							
HED							
Week 11-16	Fatigue, (N=)	Musculoskeletal discomfort, (N=)	Motivational, (N=)	Other reasons, (N=)	Missed exercises, (N=)	Number of participants with ≥1 modification (%)	Number of sessions with ≥1 modification (%)
MED							
HED							
Week 3-16	Fatigue, (N=)	Musculoskeletal discomfort, (N=)	Motivational, (N=)	Other reasons, (N=)	Missed exercises, (N=)	Number of participants with ≥1 modification (%)	Number of sessions with ≥1 modification (%)
MED							
HED							
Data are mean and standard deviation or median and interquartile range. MED: Moderate volume exercise, HED: High volume exercise							

eTable 8 Exercise modification and causes in resistance training							
Familiarization week 1-2	Fatigue, (N=)	Musculoskeletal discomfort, (N=)	Motivational, (N=)	Other reasons, (N=)	Missed exercises, (N=)	Number of participants with ≥1 modification (%)	Number of sessions with ≥1 modification (%)
MED							
Leg press							
Leg extension							
Leg curl							
Chest press							
Back row							
Total							
HED							
Leg press							
Leg extension							
Leg curl							
Chest press							
Back row							
Total							
Week 3-10	Fatigue, (N=)	Musculoskeletal discomfort, (N=)	Motivational, (N=)	Other reasons, (N=)	Missed exercises, (N=)	Number of participants with ≥1 modification (%)	Number of sessions with ≥1 modification (%)
MED							
Leg press							
Leg extension							
Leg curl							
Chest press							
Back row							
Total							
HED							
Leg press							
Leg extension							
Leg curl							
Chest press							
Back row							
Total							
Week 11-16	Fatigue, (N=)	Musculoskeletal discomfort, (N=)	Motivational, (N=)	Other reasons, (N=)	Missed exercises, (N=)	Number of participants with ≥1 modification (%)	Number of sessions with ≥1 modification (%)
MED							
Leg press							
Leg extension							
Leg curl							
Chest press							
Back row							
Total							
HED							
Leg press							
Leg extension							
Leg curl							
Chest press							
Back row							
Total							
Week 3-16	Fatigue, (N=)	Musculoskeletal discomfort, (N=)	Motivational, (N=)	Other reasons, (N=)	Missed exercises, (N=)	Number of participants with ≥1 modification (%)	Number of sessions with ≥1 modification (%)
MED							
Leg press							
Leg extension							
Leg curl							
Chest press							
Back row							
Total							
HED							
Leg press							
Leg extension							
Leg curl							
Chest press							
Back row							
Total							
Data are mean and standard deviation or median and interquartile range. MED: Moderate volume exercise, HED: High volume exercise							

eTabel 9 Adherence for aerobic and resistance training			
Familiarization week 1-2	Aerobic training, N (%)	Resistance training, N (%)	Total training, N (%)
MED			
HED			
Week 3-10	Aerobic training, N (%)	Resistance training, N (%)	Total training, N (%)
MED			
HED			
Week 11-16	Aerobic training, N (%)	Resistance training, N (%)	Total training, N (%)
MED			
HED			
Week 3-16	Aerobic training, N (%)	Resistance training, N (%)	Total training, N (%)
MED			
HED			Total training, N (%)
Total	Aerobic training, N (%)	Resistance training, N (%)	
MED			
HED			

Data are mean and standard deviation or median and interquartile range. RIR: repetitions in reserve, MED: Moderate volume exercise, HED: High volume exercise. Adherence: For prescribed aerobic training ≥ 70% of minutes should be within the target heart rate zones. For prescribed resistance training, ≥ 70% of the sets should be performed at or below the maximum RIR.

eTable 10 Coefficient of variation and precision during the hyperglycemic clamp											
	CON			DCON			MED			HED	
	0 weeks (SD or IQR)	16 weeks (SD or IQR)		0 weeks (SD or IQR)	16 weeks (SD or IQR)		0 weeks (SD or IQR)	16 weeks (SD or IQR)		0 weeks (SD or IQR)	16 weeks (SD or IQR)
Coefficient of variance (%)											
Basal											
Early phase hyperglycemia											
Steady phase hyperglycemia											
Hyperglycemia + GLP-1											
Hyperglycemia + GLP-1 + Arginine											
Off-target											
Steady phase hyperglycemia											
Hyperglycemia + GLP-1											
Data are means and standard deviations/median or interquartile ranges at baseline or follow-up or estimated within-group difference in change from baseline to follow-up with 95% confidence intervals. CON: control group, DCON: Dietary control group, MED: Moderate volume exercise, HED: High volume exercise, GIR: glucose infusion rate, Ra: Rate of appearance, Rd: Rate of disappearance: GLP-1: Glucagon-like-peptide 1											

eTable 11 Sensitivity analyses - Pairwise comparisons of the change in the primary outcome and indices of beta-cell function and insulin sensitivity

	HED vs. CON	P-value		MED vs. CON	P-value		DCON vs. CON	P-value		HED vs. DCON	P-value		MED vs. DCON	P-value		HED vs. MED	P-value
Per protocol#																	
Primary outcome																	
Late-phase Disposition index (hyperglycemic clamp)																	
Major Secondary outcomes																	
Late-phase insulin sensitivity (hyperglycemic clamp)																	
Late-phase insulin secretion rate (hyperglycemic clamp)																	
Oral disposition index (MMTT)																	
Oral insulin sensitivity (MMTT)																	
Oral insulinogenic index (MMTT)																	
Imputation																	
Primary outcome																	
Late-phase Disposition index (hyperglycemic clamp)																	
Secondary outcomes																	
Late-phase insulin sensitivity (hyperglycemic clamp)																	
Late-phase insulin secretion rate (hyperglycemic clamp)																	
Oral disposition index (MMTT)																	
Oral insulin sensitivity (MMTT)																	
Oral insulinogenic index (MMTT)																	

Data are estimated mean difference in changes between groups with 95% confidence intervals. CON: control group, DCON: Dietary control group, MED: Moderate volume exercise, HED: High volume exercise, MMTT: Mixed meal tolerance test

Adjusted for sex, age, diabetes duration, baseline maximal oxygen consumption

eTable 12 Baseline values and within group changes (0-16 weeks) for other outcomes from the mixed meal tolerance test derived outcomes

	CON			DCON			MED			HED	
	0 weeks (SD or IQR)	Change (95% CI)		0 weeks (SD or IQR)	Change (95% CI)		0 weeks (SD or IQR)	Change (95% CI)		0 weeks (SD or IQR)	Change (95% CI)
0-15 min											
Incremental AUC											
iAUC glucose											
iAUC C-peptide											
iAUC insulin											
iAUC GLP-1$_{total}$											
iAUC GLP-1$_{active}$											
iAUC GIP$_{total}$											
iAUC paracetamol											
Total AUC											
tAUC glucose											
tAUC C-peptide											
tAUC insulin											
tAUC GLP-1$_{total}$											
tAUC GLP-1$_{active}$											
tAUC GIP$_{total}$											
tAUC paracetamol											
0-30 min											
Incremental AUC											
iAUC glucose											
iAUC C-peptide											
iAUC insulin											
iAUC GLP-1$_{total}$											
iAUC GLP-1$_{active}$											
iAUC GIP$_{total}$											
iAUC paracetamol											
Total AUC											
tAUC glucose											
tAUC C-peptide											
tAUC insulin											
tAUC GLP-1$_{total}$											
tAUC GLP-1$_{active}$											
tAUC GIP$_{total}$											
tAUC paracetamol											
0-60 min											
Incremental AUC											
iAUC glucose											
iAUC C-peptide											
iAUC insulin											
iAUC GLP-1$_{total}$											
iAUC GLP-1$_{active}$											
iAUC GIP$_{total}$											
iAUC paracetamol											
Total AUC											
tAUC glucose											
tAUC C-peptide											
tAUC insulin											
tAUC GLP-1$_{total}$											
tAUC GLP-1$_{active}$											
tAUC GIP$_{total}$											
tAUC paracetamol											
0-180 min											
Incremental AUC											
iAUC glucose											
iAUC C-peptide											
iAUC insulin											
iAUC GLP-1$_{total}$											

iAUC GLP-1$_{active}$										
iAUC paracetamol										
Total AUC										
tAUC glucose										
tAUC C-peptide										
tAUC insulin										
tAUC GLP-1$_{total}$										
tAUC GLP-1$_{active}$										
tAUC paracetamol										

Data are means and standard deviations/median or interquartile ranges at baseline or follow-up or estimated within-group difference in change from baseline to follow-up with 95% confidence intervals. CON: control group, DCON: Dietary control group, MED: Moderate volume exercise, HED: High volume exercise, GLP-1: Glucagon-like-peptide 1, GIP: Gastric inhibitory polypeptide, tAUC: total area under the curve, iAUC: incremental area under the curve.

eTable 13 Other Pairwise comparisons of secondary outcomes derived from the mixed meal tolerance test

	HED vs. CON	P-value		MED vs. CON	P-value		DCON vs. CON	P-value		HED vs. DCON	P-value		MED vs. DCON	P-value		HED vs. MED	P-value
Total AUC																		
0-15 min																		
tAUC C-peptide																		
tAUC insulin																		
tAUC GLP-1_{total}																		
tAUC GLP-1_{active}																		
tAUC GIP_{total}																		
tAUC paracetamol																		
0-60 min																		
tAUC glucose																		
tAUC C-peptide																		
tAUC insulin																		
tAUC GLP-1_{total}																		
tAUC GLP-1_{active}																		
tAUC GIP_{total}																		
tAUC paracetamol																		
0-180 min																		
tAUC glucose																		
tAUC C-peptide																		
tAUC insulin																		
tAUC GLP-1_{total}																		
tAUC GLP-1_{active}																		
tAUC GIP_{total}																		
tAUC paracetamol																		
Incremental AUC																		
0-15 min																		
iAUC glucose																		
iAUC C-peptide																		
iAUC insulin																		
iAUC GLP-1_{total}																		
iAUC GLP-1_{active}																		
iAUC GIP_{total}																		
iAUC paracetamol																		
0-30 min																		
iAUC glucose																		
iAUC C-peptide																		
iAUC insulin																		
iAUC GLP-1_{total}																		
iAUC GLP-1_{active}																		
iAUC GIP_{total}																		
iAUC paracetamol																		
0-60 min																		
iAUC glucose																		
iAUC C-peptide																		
iAUC insulin																		
iAUC GLP-1_{total}																		
iAUC GLP-1_{active}																		
iAUC GIP_{total}																		
iAUC paracetamol																		
0-180 min																		
iAUC glucose																		
iAUC C-peptide																		
iAUC insulin																		
iAUC GLP-1_{total}																		
iAUC GLP-1_{active}																		
iAUC paracetamol																		

Data are estimated mean difference in changes between groups with 95% confidence intervals. CON: control group, DCON: Dietary control group, MED: Moderate volume exercise, HED: High volume exercise, GLP-1: Glucagon-like-peptide 1, GIP: Gastric inhibitory polypeptide, tAUC: total area under the curve, iAUC: incremental area under the curve

eTable 14 Within-group changes (0-16 weeks) cardiometabolic, body composition and fitness								
	CON		DCON		MED		HED	
	Change	95% CI	Change	95% CI	Change	95% CI	Change	95% CI
Glycemic control								
HbA1c (mmol/mol)								
HbA1c (%)								
Fasting glucose (mmol/l)								
Fasting insulin (pmol/l)								
Fasting C-peptide (pmol/l)								
Glucose-lowering medication, No (%)								
Reduction[a]								
Discontinuation[b]								
Intensification[c]								
Lipid-lowering medication, No (%)								
Reduction[a]								
Discontinuation[b]								
Intensification[c]								
Blood pressure lowering medication, No (%)								
Reduction[a]								
Discontinuation[b]								
Intensification[c]								
Lipids								
LDL cholesterol (mmol/l)								
Fasting triglycerides (mmol/l)								
Blood pressure								
Systolic (mmHg)								
Diastolic (mmHg)								
Fitness								
Absolute VO_2 max (ml/min)								
Relative VO_2 max (ml/kg/min)								
Watt max (W/kg)								
1 RM chest press (kg)								
1 RM leg extension (kg)								
Body composition								
Body weight (kg)								
BMI (kg/m^2)								

Data are least-squares means. CI: confidence intervals, CON: control group, DCON: Dietary control group, MED: Moderate volume exercise, HED: High volume exercise

[a]Reduction defined as at least one step down on the pre-defined algorithm.

[b]Discontinuation defined as, discontinuation of all drugs when therapeutic target was met.

[c]Intensification defined as at least one step up on the pre-defined algorithm.

eTable 15 Pairwise comparisons of the change in cardiometabolic, body composition and fitness

	HED vs. CON	P-value		MED vs. CON	P-value		DCON vs. CON	P-value		HED vs. DCON	P-value		MED vs. DCON	P-value		HED vs. MED	P-value
Glycemic control																	
HbA1c (mmol/mol)																	
HbA1c (%)																	
Fasting glucose (mmol/l)																	
Fasting insulin (pmol/l)																	
Fasting C-peptide (pmol/l)																	
Glucose-lowering medication, No (%)																	
Reduction																	
Discontinuation																	
Intensification																	
Lipid-lowering medication, No (%)																	
Reduction																	
Discontinuation																	
Intensification																	
Blood pressure lowering medication, No (%)																	
Reduction																	
Discontinuation																	
Intensification																	
Lipids																	
Total cholesterol (mmol/l)																	
LDL cholesterol (mmol/l)																	
Fasting triglycerides (mmol/l)[a]																	
Blood pressure																	
Systolic (mmHg)																	
Diastolic (mmHg)																	
Fitness																	
Absolute VO_2 max (ml/min)																	
Relative VO_2 max (ml/kg/min)																	
Watt max (W/kg)																	
1 RM chest press (kg)																	
1 RM leg extension (kg)																	
Body composition																	
Body weight (kg)																	
BMI (kg/m^2)																	

Data are estimated mean difference in changes between groups with 95% confidence intervals. CON: control group, DCON: Dietary control group, MED: Moderate volume exercise, HED: High volume exercise, HbA1c: Glycated hemoglobin 1Ac, GLP-1: Glucagon-like-peptide 1, GIP: Gastric inhibitory polypeptide

eTable 16 Adverse events after randomization

Event	All n (%)	CON n (%)	DCON n (%)	MED n (%)	HED n (%)
Serious AE					
All AE					
Gastrointestinal					
Nausea					
Vomiting					
Diarrhea					
Constipation					
Dyspepsia					
Flatulens					
Abdominal distension					
Abdominal pain					
Other					
Infections					
Musculoskeletal pain and discomfort					
Back pain					
Lower extremities					
Upper extremities					
other					
Musculoskeletal injury, defined as pain or discomfort resulting in inability to exercise for ≥7days					
Back pain					
Lower extremities					
Upper extremities					
other					
Complications associated with clinical or experimental procedures					
Metabolism and nutrition disorders					
Decreased appetite					
Increased appetite					
Hunger					
Other					
Nervous system disorders					
Headache					
Dizziness					
Other					
Events related to dysglycemia					
Events related to blood pressure management					
Other					

Values are number and percentage (%) of participants with adverse event for each group. All events are self-reported to reported to the study nurse, dietitian or trainers and occurred after randomization.

Mathias Ried-Larsen

Title

The effects of different doses of exercise on pancreatic β-cell function in patients with newly diagnosed type 2 diabetes (DOSE-EX): A randomized clinical trial

Trial registration

Intended registry: www.clinicaltrials.gov (Awaiting approval from the Research Ethics Committee, The Capital Region of Denmark)

Protocol version

4.0

Protocol date

October 16st 2019

Funding statement

This project is funded by a grant from Trygfonden (9M DKr). The grant covers salary, running costs and equipment. MR-L is employed at the Centre for Physical Activity Research (CFAS), Rigshospitalet, Denmark. CFAS is funded by Trygfonden (40M DKr). Funding (400.000 DKr) is also achieved from Svend Andersen fonden. The investigators declare no conflicts of interest. No private companies are involved in the study. If additional funding is obtained, the Research Ethical Committee and the participants will be notified.

Trial sponsor

Center for Aktiv Sundhed – Rigshospitalet
Mathias Ried-Larsen
Blegdamsvej 9
2100, Copenhagen
Denmark
Phone: +4535457641
Contact: Mathias Ried-Larsen

Content

Roles and responsibilities 4
Introduction 6
Pancreatic β-cell function and non-pharmacological interventions in T2D. 6
The role of exercise in restoring pancreatic β-cell function 7
The patient population and concomitant care 8
The role of exercise in mechanisms leading to the development of T2D complications. 8
Study objectives and hypotheses 9
Objectives 9
Research hypotheses: 9
Methods 10
Design: 10
Eligibility criteria 11
Interventions 12
Increased exercise doses and dietary intervention 12
Control intervention: 17
Concomitant care (all participants) 18
Outcomes 21
Primary outcome 21
Key secondary outcome 21
Secondary outcomes (difference in changes between groups from baseline to 16 weeks follow-up) 21
Sample size considerations 23
Recruitment 23
Allocation 24
Blinding 24
Emergency un-blinding 25
Data collection, management and analysis 25
Data collection methods 26
Mixed meal tolerance test 27
Continuous glucose monitoring (CGM) 27
Biopsies 27
Diet record 29
Retention 29
Data management 30

Statistical methods 30
Harms, risks and discomforts 31
Ethics and dissemination 33
Protocol amendments 34
Consent or assent 34
Confidentiality 34
Declaration of interests 35
Access to data 35
Ancillary and post-trial care 35
Dissemination policy 35
Informed consent materials: 35

Roles and responsibilities

Principal Investigators
Mathias Ried-Larsen, PhD, Group leader

Co-Investigators
Bente Klarlund Pedersen (BKP), MD, DMsc, professor
Grit Elster Legård (GEL) – *research physician*, MD[1]
Mark Preben Lyngbæk (MPL) – *research physician*, MD
Regitse Højgaard (RH) – *research physician,* MD
Kristian Karstoft (KK), MD, Ph.D. [1,2]
Thomas Peter Almdal (TPA) – *research physician*, MD, DMSc, Senior physician/Consultant Diabetologist
Thomas Solomon (TS), MSc., Ph.D.
Helga Ellingsgaard (HE), MSc., Ph.D., Group leader
Charlotte Glümer (CG), Ph.D., professor
Søren Nielsen (SN), MSc., Ph.D., Group leader
Robin Christensen (RC), Ph.D., professor[8]

Steering committee
Mathias Ried-Larsen, Thomas Peter Almdal, Bente Klarlund Pedersen

Organizational structure and responsibilities

Principal investigators and research physicians

- Design and conduct of DOSE-EX
- Preparation of protocol and revisions
- Preparation and submission of registration to the Danish Data Authorities
- Clinical trials registration
- Preparation of Standard Operation Procedures brochures (SOP)
- Preparation of case report forms (CRF)
- Organizing steering committee meetings
- Publication of study reports
- Report serious adverse events (SAE) to the research ethics committee (REC)
- Members of trial management committee

Steering committee (see title page for members)

- Agreement of final protocol
- Reviewing progress of study
- Reviewing and Agreeing to changes to the protocol and/or SOP's
- Reviewing and agreeing to sub-study proposals

Trial management committee

- PI, Research Physicians, Administration
- Study planning
- Organization of steering committee meetings
- Organization recruitment and biweekly progress reports on recruitment status
- Provide annual report to the REC
- Responsible for trial master file
- Budget administration and purchases of equipment
- Advice for lead investigators
- Intervention organization
- Organization of data collection
- Assistance with international review, board/independent ethics committee applications
- Data verification
- Allocation

Introduction

Lifestyle intervention including exercise therapy is a cornerstone in clinical care of type 2 diabetes (T2D). The current exercise recommendation for T2D is 150 min/week of moderate to vigorous intensity aerobic exercise, supplemented with resistance training 2-3 days/week [1,2]. The rationale for the recommendations are primarily based on reductions in HbA1c, whereas evidence supporting an effect on central T2D pathophysiological mechanisms e.g. pancreatic β-cell function is scarce[1,2]. As exercise induces health effects that most likely are not exclusively mediated via HbA1c or other known surrogate markers, this study aims at understanding exercise dosing beyond HbA1c in order to approach minimal exercise dose to reduce the risk of micro and macro vascular complications. In fact, epidemiological studies suggest that the vigorous leisure time physical activity should be above 4 and as high as 10 hours per week to reduce the risk of vascular complications [3-6]. Thus, it may in this perspective very well be that the most efficient exercise dose is higher than the current recommendations. With the aim of developing "exercise as medicine" for patients with T2D, efficacy in relation to clinically relevant endpoints needs to be demonstrated. Moreover, dose-response efficiency of exercise on these endpoints needs to be established – i.e., establishing the minimum dose necessary to affect these outcomes. As targeting a reduction in HbA1c might not lead to a reduction in complications, knowledge about the exercise dose needed to reduce micro- and macrovascular complications in T2D is largely unknown [7-15]. In essence, as the most clinical exercise interventions in T2D base their conclusions on HbA1c, the significance of exercise in the clinical care of prevalent T2D is challenged [14,16-18].

Pancreatic β-cell function and non-pharmacological interventions in T2D.

Although T2D is in general regarded as a treatable yet chronic condition, several non-pharmacological therapies have been demonstrated to introduce remission and reversal of pancreatic β-cell function (normal/non-diabetic glycemic control without the use of glucose lowering therapy)[19-21]. These are weight reduction, dietary or surgical, and exercise. In a recent trial we observed that an exercise-based (6 sessions of exercise/week) lifestyle intervention with a hypocaloric diet component eliminated the need for glucose lowering medications in 56% of the intervention group following the 12-month intervention[22,23]. Thus, there is evidence supporting that T2D may be a reversible disease.

Glycemic control is maintained through a relationship between insulin sensitivity and insulin secretion. This relationship is inversely and proportionally related, where glucose homeostasis is tightly regulated by a feedback loop with crosstalk between pancreatic β-cells and insulin-sensitive tissue[24]. Whereas insulin resistance is the earliest detectable abnormality in T2D[25], dysfunction in the insulin secretory capacity determines the onset of hyperglycemia and concomitant treatment[26]. By the time of T2D diagnosis the insulin secretory capacity of the β-cell may be reduced by up to 50 percent[27]. This β-cell dysfunction may be caused by lipo- and glucotoxity[28,29]. The presence of high FFA availability in presence of hyperglycemia is detrimental to β-cell survival and is believed to cause β-cell apoptosis. The pancreatic β-cell in T2D is subject to oxidative stress, endoplasmic reticulum (ER) stress and amyloid deposition. Moreover, ER stress caused by an abundance of FFA, may cause a depletion of Ca^{2+} stores and prevent the release of insulin[30,31]. As previously proposed, an increased hepatic insulin resistance will increase *de novo lipogeneses*, and thereby increase delivery of lipids from the liver to other tissues[32], amongst those the pancreas where they will accumulate[33]. Excess circulating FFA may be taken up by the β-cells and stored as intracellular lipids [33]. Due to peripheral (skeletal muscle and adipose tissue) and central (liver) insulin resistance, increased levels of portal insulin develops and may stimulate the storage of lipids in β-cells[33]. Finally, local inflammation, namely glucose induced IL1-β, may cause oxidative stress by activation of the NFκB pathway and activation of infiltrated macrophages[30]. Indeed, pharmacological inhibition of IL1-β by subcutaneous injections of IL1-ra increased

the β-cell secretory function following 13 weeks of treatment in T2D patients[34]. This supports that inflammation plays an important role in the etiology of T2D and may be causally related to pancreatic β-cell dysfunction in T2D.

Bariatric surgery and extreme dietary calorie restriction along with a rapid weight loss have shown to improve β-cell function within days to weeks[35]. This coincides with a decrease in hepatic lipid deposition, followed by a decrease in pancreatic lipid deposition[33]. It has thus been proposed that secondary to the restoration of hepatic insulin sensitivity by depletion of intracellular lipid accumulation from weight loss, the delivery of TAG to other tissues are decreased and thus the function of the β-cell is restored through a depletion of TAG/FFA[33,35,36]. Indeed, to accomplish the massive weight loss needed to restore function (11-20 kg of body weight) a maintained and extreme calorie restriction or bariatric surgery is needed. Thus, it is evident that diet-induced weight loss is pivotal in reversing β-cell function in a dose-dependent manner[19,37,38], and should be considered in concert with exercise intervention in the clinical care of T2D.

The role of exercise in restoring pancreatic β-cell function

It is well established that exercise improves insulin sensitivity in the peripheral tissue[39,40] and thus may induce pancreatic β-cell rest. Only a few studies have focused on the effects of exercise on pancreatic β-cell function in T2D and discrepancies regarding the effect exists[41-45]. The discrepancies may relate to the assessment of β-cell function[46], concomitant pharmacological therapy and the pre-trial insulin secretory capacity. Moreover, exercise intensity, volume and modality may play a role[15,17,47-49].

Exercise may, independently of weight loss, relieve hepatic insulin resistance[50] and decrease *de novo lipogeneses*[51] and lower plasma TAG[52]. Accordingly, in a recent study from Heiskanen et al., it was observed that only 14 weeks of exercise decreased pancreatic ectopic lipid accumulation while improving β-cell function in both participants with and without T2D[53], lending support to the so-called twin-cycle hypothesis. The latter hypothesis postulates that chronic calorie excess leads to accumulation of liver fat with eventual spill over into the pancreas. These self-reinforcing cycles between liver and pancreas eventually cause metabolic inhibition of insulin secretion after meals and onset of hyperglycemia. However, in our recent trial, we observed that the oral disposition index (DI - a marker of β-cell function) was improved despite only a modest weight loss (unpublished data). This improvement was due to an improved insulin secretion rate as well as an improved whole body insulin sensitivity. In fact, in a post hoc analyses we found that in relation to weight change only a non-significant 3 kg difference in weight loss ($p=0.1$) and a <0.4 kg difference in loss of abdominal fat mass ($p=0.1$) were observed between participants achieving T2D remission and participants that did not, following the 12-month intervention (unpublished data). This suggests that adding exercise to weight loss may work through different mechanisms than proposed by the twin cycle hypothesis. In post hoc analyses (in preparation) we observed that IL1-ra was significantly reduced following the intervention. This reduction in IL1-ra probably reflects a reduction in IL1-β which is causatively related to β-cell function[31]. Indeed, the reduction in IL1-ra explained >60% of the improvement in DI observed in the study (in preparation). However, the methodology did not allow us to understand the mechanism behind this observation nor did it allow us to investigate the causative role of manipulating the exercise dose in the context of lifestyle intervention.

It is well recognized that the muscle is an endocrine organ and that exercise elicits an anti-inflammatory effects in a cross-talk with multiple organs[54]. Moreover, it has previously been suggested that the anti-inflammatory effects of exercise may be mechanistically linked to improved β-cell function in T2D [55], through mechanisms that are distinct different from diet induced weight loss. It is thus evident that diet induced weight loss and exercise may complement each other, and including exercise to diet-induced weight loss provide additive effects in relation the re-establish the pancreatic β-cell function. Thus, we propose that

combining a moderate diet-induced weight loss with exercise may re-establish pancreatic β-cell function through decreased metabolic stress and an anti-inflammatory effect of exercise in T2D patients with no concomitant glucose- and lipid lowering pharmacological therapy. Moreover, as the volume of exercise needed to re-establish β-cell function is unknown this needs to be established in order to gain knowledge about the role of exercise-based lifestyle intervention as a first-line therapy for T2D patients.

The patient population and concomitant care

Poor glycaemic control and poor β-cell function prior to training predict less benefit from training[56,57]. Indeed, Dela et al. showed that T2D patients with remaining insulin secretory function improved the insulin secretory capacity with exercise, whereas participants with low secretory capacity prior to the intervention did not[42]. This is in line with observations from other lifestyle interventions in T2D[58,59]. Moreover, to avoid any risk of drug-induced signs of hypoglycemia or hypotension, previous trials with an expected decrease in body weight in other populations of T2D patients have adjusted the concomitant glucose-and BP lowering medications according to symptomatology and/or standard care guidelines without any adverse effects[1959].

To use lifestyle as a first-line monotherapy it is thus sensible **a)** to focus on T2D patients with remaining β-cell function prior to the intervention and **b)** to adjust concomitant pharmacological therapy according to symptomatology and existing guidelines when investigating the effects of lifestyle intervention on pancreatic β-cell function.

The role of exercise in mechanisms leading to the development of T2D complications.

Both a physically inactive lifestyle and abdominal adiposity create a state of chronic low-grade inflammation [60]. Together reactive oxygen species (ROS) and inflammation contribute to the development of micro- and macro-vascular complications [60,61]. The recent finding that anti-inflammatory therapy leads to a significantly lower rate of recurrent cardiovascular events in patients at high risk [62] suggests that the anti-inflammatory actions of exercise may also influence macrovascular complications to diabetes. Glycaemic variability, defined as the mean amplitude of glycaemic excursions (MAGE), is associated with coronary artery disease, vascular endothelial dysfunction, possibly through increased oxidative stress independent of HbA1c, and fasting plasma glucose [63-65]. High glycaemic variability and hyperglycemia triggers the production of ROS which may drive local and systemic low-grad inflammation[60,66,67]. It has been proposed that the accelerated formation of advanced glycation end-products (AGEs) and their interaction with the receptor for AGEs (RAGE) cause ROS and low-grade inflammation [60,68]. AGEs bind to AGE receptors that can be broadly classified into those that degrade or detoxify AGEs, and those that signal to increase oxidative stress and inflammation upon ligand binding, namely RAGE as well as other AGE receptors[60]. The activation of RAGE by AGEs results in the initiation of various signal transduction cascades and transcription factors such as nuclear factor (NF)-κB, which ultimately cause the generation of ROS and subsequently inflammation. Thus, AGE and ROS seem to represent an important, interconnected pathogenic mechanism involved in all microvascular complications [60]. As exercise represents a natural, strong anti-inflammatory strategy [55,69], exercise may reduce the risk of complications. Thus, we propose that, secondary to a reduction in glycemic variability, exercise may reduce inflammation between systemic ROS and inflammation through the AGE/RAGE axis.

Study objectives and hypotheses

Objectives

Primary objective: To investigate the dose-dependency of exercise on pancreatic β-cell function in patients with short standing T2D.

Secondary objectives: To investigate the dosing effect of exercise on markers of T2D pathogenesis beyond glucose tolerance and mechanisms that may lead to restoration of pancreatic β–cell function, vascular complications and elucidate the causality and time relation between these various causes and consequences of the disease.

Research hypotheses:

Primary: The effect of exercise on pancreatic β-cell function (disposition index) increases with increasing volumes of exercise in combination with a diet across a 4-month intervention in patients with T2D of short duration. It is expected that both moderate volume and high volumes of exercise in combination with a dietary intervention is superior to the control intervention for improving pancreatic β-cell function. It is moreover expected that high volumes of exercise are superior to moderate volumes of exercise and to dietary intervention alone in improving pancreatic β-cell function.

Secondary: Exercise reduces glycaemic variability in a dose-dependent manner followed by reductions in systemic ROS and inflammation mediated by alterations in the AGE/RAGE axis.

Mathias Ried-Larsen

Methods

Design:

A parallel-group 4-arm assessor-blinded randomised clinical trial (Figure 1). Participants will be randomly allocated (1:1:1:1) stratified by sex to;

1) No intervention (CON)
2) Dietary intervention (DCON)
3) Dietary intervention + moderate volume exercise (MED)
4) Dietary intervention + high volume exercise (HED)

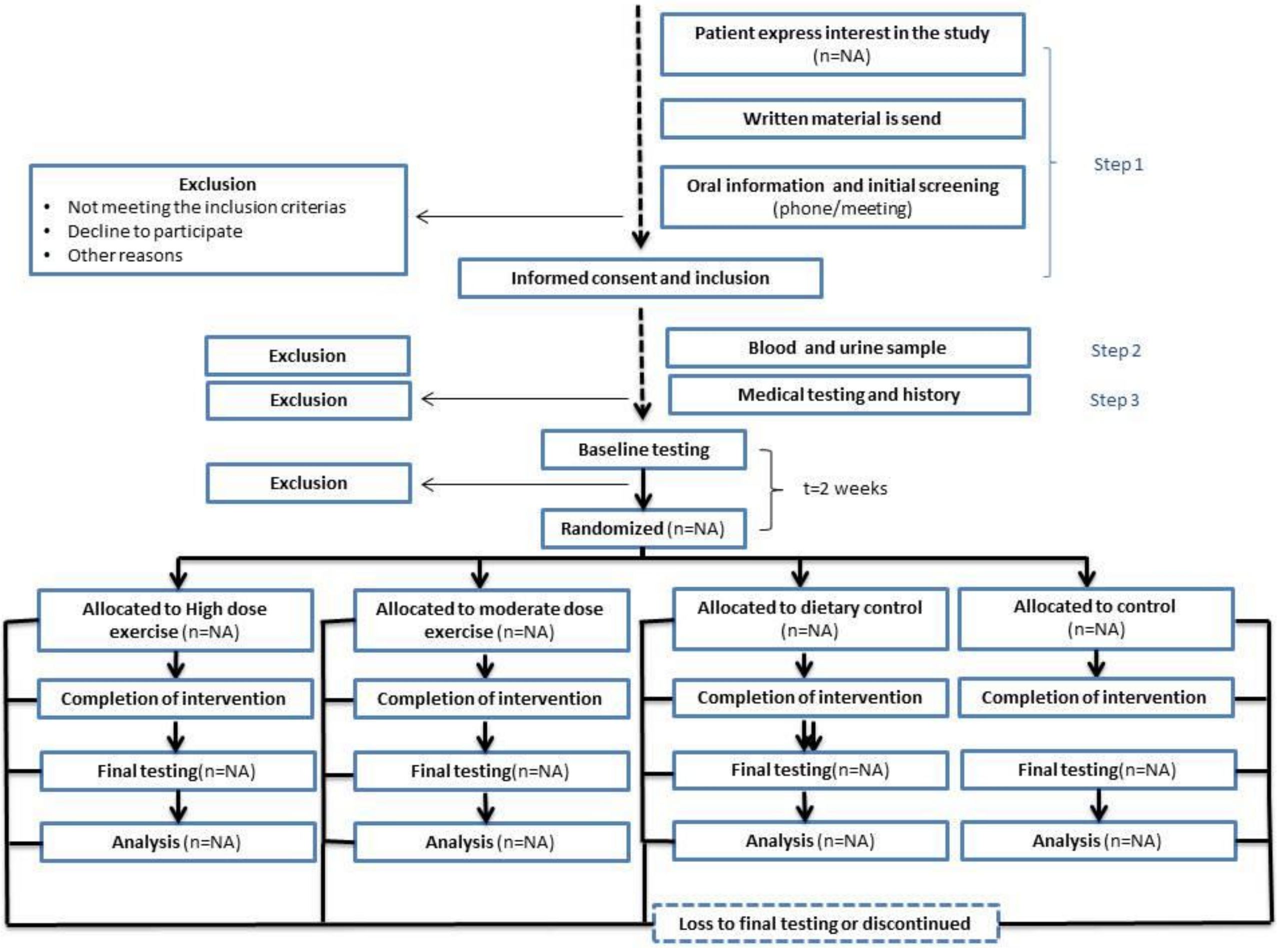

Figure 1: Flow of participants.

Primary place of study execution and data collection

Centre for Physical Activity Research (CFAS)
Rigshospitalet, section M7641
Tagensvej 20, DK-2200 Copenhagen (study address)
Blegdamsvej 9, DK-2100 Copenhagen (postal address)
Telephone: (+45) 3545 7641

All data will be collected and analysed in Denmark

Eligibility criteria

Inclusion criteria

1. Men and women aged 18-80 years
2. Diagnosed with diabetes type 2 and/or HbA1c ≥ 48 mmol/mol if no treatment with anti-diabetic medication and/or use of antidiabetic medication
3. Caucasian
4. No diagnose of Type 1 diabetes, MODY-diabetes, Type 1½ diabetes or LADA-diabetes
5. T2D 0-6 years of duration
6. No treatment with insulin
7. No use of sulphonylurea based drugs
8. Body Mass Index (BMI) >27 kg/m2 and <40 kg/m2
9. No known or signs of intermediate or severe microvascular complications to diabetes (retino-, neuro- or nephropathy)
10. No known cancer
11. No lung disease, other than asthma that can be managed with beta2-agonists and does not exhibit seasonal variation .
12. No known cardiovascular disease
13. No known hyperthyroid disease
14. No changes in hypothyroid disease treatment within the last 3 three months prior to enrolment
15. No known liver disease - defined as ALAT or ASAT elevated three times above upper limit.
16. No known autoimmune disease
17. No psoriasis disease requiring systemic treatment or cutan elements bigger than a total area of 25 cm^2
18. No other endocrine disorder causing obesity
19. No current treatment with anti-obesity medication
20. No current treatment with anti-inflammatory medication
21. No weight loss of > 5kg within the last 6 months
22. No changes in symptoms or anti-depressive medication three months prior to enrolment.
23. No diagnose of psychiatric disorder or treatment with anti-psychotic medication
24. No history of suicidal behavior or ideations within the last three months **prior** enrolment
25. No previous surgical treatment for obesity (excluding liposuction > 1 year prior to enrolment)
26. Not pregnant/considering pregnancy
27. No functional impairments that prevents the performance of intensive exercise
28. Accept of medical regulation by the DOSE-EX endocrinologist
29. Inactivity, defined as < 1,5 hours of structured physical activity pr. week at moderate intensity and cycling < 30 minutes/5 km pr. day at moderate intensity (moderate intensity = out of breath but able to speak)
30. No participation in other research intervention studies

Exclusion criteria

1. HbA1c: >=75 mmol/mol with no glucose lowering medications

2. HbA1c: >=64 mmol/mol with mono glucose lowering therapy (if compliant with the prescription)
3. HbA1c: >=57 mmol/mol with >=dual glucose lowering therapy (if compliant with the prescription)
4. eGFR<60mL/min
5. Macroalbuminuria at pre-screening
6. Clinical or biochemical signs of hypothyroid disease
7. Biochemical sign of other major diseases
8. Presence of circulating glutamatdecarboxylase anti body (GAD) 65
9. Objective findings that contraindicates participation in intensive exercise
10. Anamnestic findings that contraindicates participation in the study
11. Unable to allocate the needed time to fulfill the intervention
12. Language barrier, mental incapacity, unwillingness or inability to understand and be able to complete the interventions

Interventions

General description

The general intervention is based on a previous trial and adapted to the aim of this study[23,70]. The active interventions will consist of two main components; 1) increased physical activity and structured exercise and/or 2) a dietary intervention aiming at a weight loss. Whereas there will be no differences in the dietary intervention between the lifestyle groups, the volume of physical activity and structured exercise will be varied according to the frequencies of the structured exercise sessions. The study groups are prescribed:

1) **Control group (CON):** No intervention
2) **Dietary control (DCON):** Dietary intervention (see below)
3) **Moderate Exercise Dose (MED):** Two aerobic training sessions per week of 45-60 min duration and one session per week with combined aerobic (30-35 min) and resistance (30 min) training and a dietary intervention (described below)
4) **High Exercise Dose (HED):** Four aerobic training sessions per week of 45-60 min duration and two sessions per week with combined aerobic (30-35 min) and resistance (30 min) training and a dietary intervention (described below)

Increased exercise doses and dietary intervention

Detailed description of the intervention components.

Dietary intervention and intended weight loss (DCON, MED and HED)

The dietary intervention will be based on the recommendations from the American Diabetes Association (ADA) with increased on macronutrient quality [71]. The macro-nutrient distributions are in line with the current guidelines from the national Diabetes Association and Canadian guidelines, where individualization in macronutrient distribution should lie within the range of 45-60E% carbohydrate, 15-20E% protein and 20-35E% fat [72]. Based on recent reviews with eleven and twelve randomized controlled trials of > four weeks duration[73-75], the diet will include food items with low glycaemic index (GI) or load (GL) diets as they are

associated with improved glycaemic control as compared with high GI or GL food items. Additionally, low GI diets are effective in T2D management [76]. Thus, the dietary intervention emphasis will be on low GI and low GL in shape of non-processed foods. Since T2DM is associated with co-morbidities like cardiovascular disease and due to the general consensus that saturated fat intake is related to cardiovascular disease risk [77] thus the dietary plan will aim at reducing saturated fat intake <7E% as proposed by ADA[71]. Both prevention and a successful management of type 2 diabetes are highly related to diets rich in whole grains, fruits, vegetables, nuts and legumes and lower on refined grains, red or processed meat and sugar sweetened beverages[77], why these will be targeted throughout the study. The broad range of macro-nutrient composition allows for individual preferences.

Procedure: A dietician will prepare individual meal plans based on individual counselling (1 session/month) with proposed recipes (see appendix 1 for an example). The implementation and potential adjustments will be performed continuously based on self-report dietary records and discussed during group sessions (monthly) and at the individual sessions. The meal plans will cover three main meals and three snack meals per day. The content recipes may be adapted to individual participant preferences and recipes will be changed continuously throughout the intervention. Energy requirement will be based on the age-adjusted Oxford equations[78], aiming for a weight loss The participants' body weight is used for calculation of the energy requirement if the body mass index (BMI) is 25 kg/m2. If the BMI is >25, the body weight in the equation is adjusted to equal a BMI=25 kg/m2. On days with training sessions, 200 kcal/day will be added to the energy intake. In case of hypoglycemic events, energy intake will be reassessed. In order to reduce the risk of mild hypoglycaemia, a snack meal just before (100– 200 kcal) and after (200 kcal), and a main meal 2–3 h before a training session will be advised. In case of subjective signs of low blood glucose (hunger, sweating, increased heart rate, feeling uncomfortable, dizziness and confusion), the participants are instructed to eat either one piece of fruit, drink a glass of juice in combination with a piece of rye bread or crisp bread. To facilitate adherence, the participants are allowed to contact the clinical dietician by email once/ week in case of any issues regarding implementation of or concerns about the meal plan.

Table 1 Principles of the dietary intervention. Adapted from Ried-Larsen et al 2015[23].

**Principle**	**Additional comment**
Homemade food	Recipes will be available
Limit processed food items	
Include seasonal greens and fruits (minimum 600 g/day)	
Maximum 2 pieces of fruits per day	
Limit the amount of sodium	
Include fish (350 g/week)	200 g should be 'fat' fish e.g. salmon or mackerel
Fibre rich food items (3g/MJ)	
Hot meals should include fish once per week, one vegan meal per week	
Minced meat maximum twice per week	
Hot meals should contain minimum 200g vegetables per meal, max. ¼ of the plate should be meat, max. ¼ of the plate should be high glycaemic index/load food items.	

Ad libitum intake water and tea is allowed	
No sugar sweetened beverages (including soda pops, juice or artificial sweetened beverages	Juice is allowed in case of subjective signs of mild hypoglycaemia in relation to training
Alcohol is discouraged throughout the intervention period	

Increased physical activity and structured exercise
The training protocol will be adapted based on a previous study where the T2D participants were prescribed 6 weekly sessions of aerobic alone or combined aerobic and resistance training (averaging 360-420 min of exercise per week)[23]. Although mean adherence was high (82% of the planned session were completed), variation was high and imperfect[70]. Thus, a lower dose may be expected and thus a moderate dose exercise groups is formed based on the original protocol[23].
Procedure: As in our previous protocol, the target aerobic training intensity span in will be **60-88% of HR_{max}**, which is in line with current guidelines [79,23]. The intensity of the resistance training will also be in line with current guidelines, i.e. 3 bouts per muscle group with intensities varying between 6-15 repetitions maximum[79]. Thus, the exercise program will be adapted as previously described[23]. As previous analyses suggest that there may be an inverse dose-response relationship between reductions in HbA1c and aerobic exercise volume, this parameter will be used to adapt the training protocol[15,17]. As the effect of exercise on HbA1c is closer related to the number of training sessions[17], we will reduce the number of sessions by 50%, to three sessions/week in the moderate exercise dose group and maintain the original session frequency in the high dose exercise group (six sessions/week).

All training sessions will be completed at local fitness centres. All exercise programs are administered by the trainers and individualized on site based on preferences and risk of injuries. The target intensity of the exercise is not individualized based on preferences. An example of a weekly training program (high dose group) is found in Table 2. The initial two weeks of the intervention, a familiarization to the specific exercises will be prioritized to facilitate the training quality (i.e. to meet the prescribed training intensity) in the remaining part of the intervention. During this period the participants will be thoroughly introduced and to the Polar HR watch, training diaries and the concepts of *repetitions in reserve/rate of perceived exertion*.

To ascertain compliance to the intervention and quality of the training, all exercise sessions will be supervised by educated trainers with a medical training, physiotherapist training or exercise physiology training. The educated and experienced trainers will form individual exercise plans on a weekly basis to accommodate individual participant preferences in terms of exercise modality and to avoid potential overuse injuries. Due to the high risk of injuries with running[80], running will not be allowed as an exercise modality. All exercise sessions will be monitored and recorded (intensity: heart rate/resistance/repetitions/modality. Aerobic exercise duration: time see below).

Training will be supervised to ensure intensity and compliance. Furthermore, heart rate, training duration, numbers of repetitions and perceived exhaustion will be monitored at every bout of exercise which will enable us to precisely evaluate the amount of exercise performed by each of the participants.

Table 2 Example of a weekly training program for the high dose group. Adapted from Ried-Larsen et al 2015[23]

**Week day**	**Aerobic training**	**Resistance training**	**Notes to the trainers**
Monday	Duration: 40 min<br>1. 5 min warm up at 60-65 % of HR max<br>2. 20 min at 70-78 % of HR max<br>3. 15 min at 76-83 % of HR max	Duration: 30 min<br>1. 5 primary target exercises: Anterior chain (thigh), posterior chain (thigh), chest, back and shoulders.<br>2. Each target exercise is performed in 3 sets of 10-12 repetition max (RM)<br>3. You can use machines, free weights, barbells, body weight etc.<br>4. Active breaks containing core exercise are performed between each set. This means that the pause between each set is replaced with a core training exercise<br>5. The 5 core exercises should include 3 dynamic abdominal exercises and 2 lower back exercises	Make sure to inform the participant which muscle groups are activated and with time expand their “box” of different exercises. This will help them in the long run and create variety in relation to motivation but also to minimize injuries.
Tuesday	Duration: 60 min<br>1. 5 min warm up at 60-65 % of HR max<br>2. 5 min at 70-75 % of HR max<br>3. 20 min at 74-79 % of HR max<br>4. 10 min at 80-88 % of HR max<br>5. 5 min at 70-75 % of HR max<br>6. 15 min consisting of 2 HR min at 76-80 % of HR max, 2 min at 83-90 % of HR max and 1 min active recovery. Repeat 3 times.		
Wednesday	Duration: 60 min.<br>1. 5 min warm up at 60-65 % of HR max<br>2. 10 min at 68-73 % of HR max.<br>3. 15 min at 75-80 % of HR max 20 min at 77-84 % of HR max.<br>4. 10 min consisting of 30 sec max effort and 30 sec active recovery.		
Thursday	Duration: 30 min<br>1. 5 min warm up at 60-65 % of HR max<br>2. 25 min at 73-83 % of HR max	Duration: 30 min<br>1. 5 primary target exercises: Anterior chain (thigh), posterior chain (thigh), chest, back and shoulders.<br>2. Each target exercise is performed in 3 sets of 10-12 RM<br>3. You can use machines, free weights, barbells, body weight etc.<br>4. Active breaks containing core exercise are performed between each set. This means that the pause between each set is replaced with a core training exercise	

		5. The 5 core exercises should include 3 dynamic abdominal exercises and 2 lower back exercises
Friday	Duration: 60 min<br>1. 5 min warm up at 60-65 % of HR max<br>2. 15 min at 73-83 % of HR max 40 min consisting of 5 min at 76-82 % of HR max, 3 min towards max and 2 min active recovery. Repeat 4 times.	
Saturday	Rest day	
Sunday	Duration: 60 min<br>1. 45 min walking<br>2. 15 min walking/jogging in hills or on stairs.	Duration: 15 min<br>1. Core training. Free of choice.

Training of Intervention personnel: 4-6 trainers with minimum a bachelor degree in sports science or educated physiotherapists will be recruited for the training intervention and a dietician will be recruited for the dietary intervention. The primary responsibility and working tasks for the intervention staff is to assure compliance with the protocol and prevent loss-to-follow-up. As they will be responsible for motivating participants and making individual adjustments to the participants' training programs in order to reduce the risk of excessive load or injuries previous experience is required. All intervention personnel will attend a adapted (to T2D) certification course based on the ambassador program at provided to health care professional by the sponsor (see http://aktivsundhed.dk/da/ambassadorer). The course is led by the lead investigators, clinicians and psychologists and will contain the following:

- The research protocol
- Disease pathology (type 2 diabetes)
- The intervention: Organizations, exercise, physical activity, diet, sleep and coaching
- Motivation
- Medical issues during intervention (including a course in cardiopulmonary resuscitation)

Mandatory biweekly meetings for all persons (management (clinical and research) and intervention personal) will be enforced to discuss all issues related to the intervention (medical issues, challenges in relation to recruitment, motivation and drop-outs etc.)

Modifications and strategy to maintain and improve compliance

Exercise intervention: For participants in the exercise intervention group, compliance is monitored continuously through the study by the trainers. If any participants complete less than 80% of the training volume prescribed over a 1-week period, procedures to prevent drop-out are initiated. A 1-week vacation will be allowed, where the participants will receive exercise programs that will be feasible to complete at the vacation location. The programs will closely mimic the assigned intervention. In case that the exercise volume is unfeasible during vacation, the volume will be reduced. A specific plan to reach the overall exercise volume (across 16 weeks) will be drafted in collaboration with the participant (e.g. to increase the existing exercise sessions by 10 min over a period until the target volume has been reached).

The drop-out prevention procedures for the exercise session include;

1. The participant is offered an interview with an exercise trainer to help handling the worries and to help manage the time. If the lacking compliance relates to injuries, pain or resistance to exercise modality, the exercise modality may be altered, whereas the exercise intensity will be maintained.
2. If compliance is not corrected/maintain based on action 1 within a week, a temporary adjusted plan is made in collaboration between the trainers and participant with the aim of maintaining the weekly training volume by reducing the sessions of exercise per week but increase duration (unchanged intensity).
3. If this is not sufficient to correct/maintain compliance, the training volume will be reduced for a short period of time by retracting 1/3 of the exercise sessions per week for 2 weeks. During this procedure a plan to restore the training volume is formed in collaboration between the trainer and the participant.

Dietary intervention: If participants exceed +/- 30% of the prescribed energy intake as assessed by their dietary records the procedures below are initiated. Moreover, if the participant contacts any intervention staff and expresses concerns about satiety, food preferences or food preparation techniques the procedures, described below are initiated. A 1-week vacation will be allowed, where the participants will receive dietary guidance that will be feasible to complete at the vacation location. Moreover, the participant will be asked to complete at dietary record during the vacation, in order to adjust the following dietary program to reach the pre-specified energy intake.

The drop-out prevention procedures for the dietary intervention include;

1) An interview regarding compliance to the meal plan is performed, and the participant is provided with specific guidelines to practical changes in the plan by the clinical dieticians. E.g. to increase adherence to food items increasing satiety or exchange some food items match preferences
2) If action 1 is in sufficient then the energy intake is increased in steps of 100 Kcal/day until the level of satiety is acceptable by the participant.

Adherence assessment

Posture allocation and physical activity behaviours are measured using three axial accelerometer-based physical activity monitors (Axivity AX3, Newcastle, UK). See procedures below. All heart rate profiles are recorded during in the intervention group (not control) (Polar V800, Polar, Holte, Dk). As multiple modalities are allowed to target the muscle groups described in the training plan (Table 2), all participants in the active group receive a sheet with pictograms of possible exercises available in the training center (see appendix 2 for an example). The final sheets are formed when final training locations are identified. The participant will note the resistance, rate of perceived exertion and number for repetitions for each exercise and general notes about the quality and resistance modifications. Moreover, if the participant does not complete or partially complete the session as prescribed, the reasons for that is noted on the sheet. The trainers will randomly check the validity of the self-report during the sessions. At the end of the sessions the sheets are collected by the trainers and stored at CFAS for later data management. The participants are moreover asked to complete weighed dietary records during the intervention. See procedures below.

Control intervention:

The control group will be instructed to maintain regular physical activity and diet behaviors throughout the study. No additional intervention is introduced.

Concomitant care (all participants)

Pharmacological treatment procedures and algorithm:
Prior (48 hours) to visit 1, 2, 6 and 7 all glucose lowering medication and exercise will be discontinued (all groups). Participants will go through screenings (on site) of HbA1c levels, cholesterol levels and home blood pressure monitoring during the intervention. Glucose-lowering and antihypertensive may be reduced or discontinued to avoid hypotensive or hypoglycemic events (see procedures below). The clinical responsibility of the pharmacological T2D treatment will be transferred from the patient general practitioner (GP) to the study endocrinologist (Consultant diabetologist Thomas Peter Almdal) throughout the study period. The participant GP will be informed about study participation and procedure and encouraged to contact the study nurse in case of questions (see appendix 3 for letter to GP). The clinical endocrinologist will perform medical regulations based on clinical parameters and symptoms (see procedures below). The endocrinologist will be blinded to group allocation, but all necessary information about intervention, medical history and adverse events will be provided through the study nurse. If considered necessary, the blinding will be repealed and the participant will be contacted directly by the endocrinologist. During the intervention (at 0, 4 and 12 weeks follow-up) the study nurse presents the anonymized data (HbA1c, triglyceride, low density lipoprotein and total cholesterol, home-based diastolic and systolic blood pressure (18 home-based resting measurements across three days).

In case of premature termination (due to any reason), the participant will be offered a consultation aiming at initiating the needed pharmacological therapy and the responsibility for the pharmacological therapy will be transferred back to the GP. The participant will be advised to contact their GP. Participants completing the trial, will have their medications readjusted their pharmacological therapy based on the current guidelines following completion and the responsibility for the pharmacological therapy will be transferred back to the GP[81]. The participants will be strongly encouraged to contact their GP upon completion of the trial.

Safety criteria and rescue medication: An experienced endocrinologist will be in charge of the regulation of medication in accordance with the pre-defined algorithms mentioned below. Participants will in details be informed about side effects as well as subjective signs of increased hypo- or hyperglycaemia (thirst, fatigue, polyuria, confusion) and urged to contact the study nurse in case of any adverse symptoms. Safety criteria include adverse events, health related outcomes (for instance episodes of angina or signs of atrial fibrillation) and participant-reported hypoglycemic episodes (plasma glucose < 4mmol/l). Major hypoglycemic periods are reported to the study nurse. Minor hypoglycemic episodes are defined as those that can be self-treated; major episodes are defined as plasma glucose <3mmol/l or episodes requiring third-party assistance or medical intervention. In case of adverse effects, medication is changed according to titration described below.

In case of patient-reported (see above) minor symptoms of hypo, or hyper glycemia or hypo- or hypertensions, the patients are asked to complete glucose (3 consecutive days: morning (fasting), before evening meal and 2 hours after evening meal (postprandial)) or BP (18 home-based resting measurements across three days) profiles. The profiles will be reviewed by the nurse and presented to the study endocrinologist in a blinded and stepwise manner (see algorithm below). The endocrinologist will adjust the medications based on the algorithm below. All events are registered in the database.

Rescue pharmacological algorithm and titration:

According to Danish national guidelines the goal in relation to glucose, blood pressure, and LDL cholesterol are as follows:

	Glucose	Blood pressure	Lipids – LDL Cholesterol
Goal	HbAc < 48 mmol/mol	130 / 80 mmHg	2,5 mmol/l

These goals will also apply to patient included in the DOSE-EX Trial, as it did in the U-Turn trial[22].

Based on the results reported from the U-TURN and Direct trial[58], is expected that a HbA1c1 in the HED and possibly in the MED group will decreased by 5 – 7 mmol/mol in the period from week 0 – 12, and in the same period a modest reduction in blood pressure is expected. In the DIRECT trial the number of prescribed antihypertensive decreased from 1,1 to 0,5 in the intervention group. At the planned assessments of the glucose and blood pressure control will be performed at weeks -2, 4 and 12, the HbA1c goals for the treatment are given below.

**Medication**	Treatment goals	Intensification of treatment at inclusion and follow-ups	Reduction in medication at inclusion and follow-ups
Glucose lowering /Antidiabetics	HbA1c = 48 mmol/mol<br><br>*Fasting glucose < 7 mmol/l*<br>*Postprandial glucose < 7,8 mmol/l*	HbAc = 53 mmol/mol<br><br>*Fasting BG > 7,0 mmol/l*<br>*Postprandial PG > 11 mmol/l*	HbA1c < 48 mmol/mol<br><br>*Fasting BG < 7 mmol/l and*<br>*Postprandial BG < 7,8 mmol/l*
Antihypertensive	BP ≤130/80 mmHg	BP >140/85 mmHg	BP < 125 / 75 mmHg

Algorithm for reduction in glucose lowering treatment:

It is anticipated that the patient at inclusion will be treated in one of the following 3 ways

1. Metformin 1000 mg x 2 + 2 two more glucose lowering drugs (Empagliflozin 25 mg x 1, and Sitagliptin 100 mg. Patients treated with sulfonylureas at inclusion be changed to one of the two above mentioned drugs)
2. Metformin 1000 mg x 2 + 1 or two more glucose lowering drugs (First line drug will be Empagliflozin 25 mg x 1, 2nd line drug will be Sitagliptin 100 mg. Patients treated with sulfonyl ureas at inclusion be changed to one of the two above mentioned drugs)
3. Metformin 1000 mg x 2
4. Metformin 500 mg x 2
5. Stop glucose lowering medication

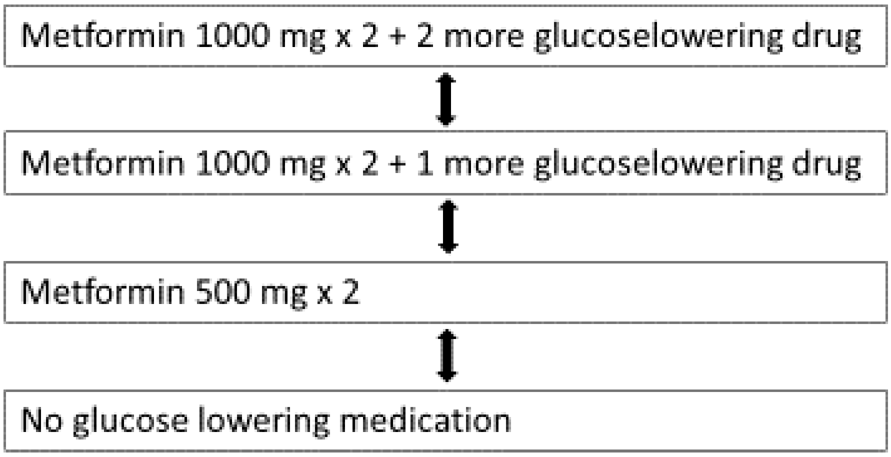

<u>Algorithm for reduction/intensification of antihypertensive treatment:</u>

It is anticipated that the patient at inclusion will be treated with 1–3 agents from the following classes of antihypertensives; AT-2 or ACE inhibitors, thiazide diuretics, and Calcium Channel blockers. If this is not the case the medication will be adjusted to comprise the 3 agents. If only 1 antihypertensive drug is given this must be AT-2 or ACE inhibitors. Based on BP measured at 18 home-based measurements done immediately before weeks - 2, 4 and 12, antihypertensive medication can be continued, can be reduced or intensified in a stepwise manner corresponding to the steps showed for glucose lowering drugs. The steps are:

1. Losartan / Enalapril 100 mg / 20 mg or equivalent dosages of any other AT-2 antagonist or ACE inhibitor + 2 other antihypertensive (a Calcium Antagonist or a thiazide diuretic)

2. Losartan / Enalapril 100 mg / 20 mg or equivalent dosages of any other AT-2 antagonist or ACE inhibitor + 1 other antihypertensive drug (can be either a Calcium Antagonist or a thiazide diuretic)

3. Losartan / Enalapril 100 mg / 20 mg or equivalent dosages of any other AT-2 antagonist or ACE inhibitor
4. Losartan / Enalapril 50 mg / 10 mg or equivalent dosages of any other AT-2 antagonist or ACE inhibitor

Prohibited medications
Biological medicine, beta blockers, anti-psychotic medicine, lithium or metabolism disorder treatment. Anti-cancer treatment, daily use of systemic-, inhalation-, or local glucocorticoids. Furthermore, no regular or daily use of non-steroid anti-inflammatory drugs. Also, no daily use of proton pump inhibitors. The day prior to visit 1 no intake of paracetamol is allowed.

Outcomes

Primary outcome

- The change in the late-phase disposition index (DI) from baseline (0 weeks) to follow-up (16 weeks) during the final 30 minutes of hyperglycemic phase of the hyperglycemic clamp. The late-phase DI is defined as the insulin secretion rate (ISR) divided by blood glucose concentration multiplied by the insulin sensitivity index (S_I) ((ISR/glucose)×S_I). The DI will form basis for hierarchical co-primary outcomes assessment based on per protocol as the differences in change of the DI between study groups;
    1. CON vs. HED
    2. CON vs. MED
    3. DCON vs. CON
    4. DCON vs. HED
    5. DCON vs. MED
    6. MED vs. HED
- The data collection in the *per-protocol population* is independent of group allocation. The per protocol population is defined as participants (all criteria present):
    1. CON
        - The primary outcome is assessed at both baseline and after 16 weeks follow-up should be available for analysis for both groups (i.e. complete case).
    2. DCON
        - The primary outcome is assessed at both baseline and after 16 weeks follow-up should be available for analysis for both groups (i.e. complete case).
        - Do not exceed +/- 30% of the prescribed energy intake as assessed by their dietary records (assessed as the mean energy intake mean across that latter 12 weeks, excluding 1-week vacation administered following week 2 of the intervention)
    3. MED and HED
        - The primary outcome is assessed at both baseline and after 16 weeks follow-up should be available for analysis for both groups (i.e. complete case).
        - >=70% of the prescribed exercise volume across the intervention period (excluding weeks 1, 2 + 1-week vacation administered following week 2 of the intervention).
        - Do not exceed +/- 30% of the prescribed energy intake as assessed by their dietary records (assessed as the mean energy intake across that latter 12 weeks, excluding 1-week vacation administered following week 2 of the intervention)

Key secondary outcome

- Intention-to-treat analyses of the primary outcome.

Secondary outcomes (difference in changes between groups from baseline to 16 weeks follow-up)

- Secondary measures of pancreatic α- and β-cell function
    - GLP-1 stimulated insulin, C-peptide and glucagon secretion
    - Arginine stimulated insulin, C-peptide and glucagon secretion
    - 1st phase C-peptide and insulin secretion defined as the peak concentration during the initial 10 minutes of the hyperglycaemic clamp
    - Late-phase S_I (mean Glucose infusion rate over last 30 min of clamp phase/(mean insulin×glucose))

  - Early phase DI (DI from 0-30 minutes)
  - Rate of glucose appearance (R_a)
  - Rate of glucose disappearance (R_d)
- Glycaemic control parameters derived from 4-7 days free-living continuous glucose monitoring
  - Mean amplitude of glycemic excursions will be calculated based on min 3 days sensor glucose profiles. MAGE is calculated by taking the arithmetic mean of the blood glucose increases or decreases (from blood glucose nadirs to peaks or vice versa) when both ascending and descending segments exceed the value of 1 SD of the blood glucose during a 24-hour measurement period[82,83]
  - Coefficient of variation defined as (mean glucose/the standard deviation (SD)) of min 3 days sensor glucose profiles
  - The SD of the glucose from min 3 days sensor glucose profiles
  - The mean glucose from min 3 days sensor glucose profiles
  - Time in hyperglycaemia defined
  - Time in hypoglycaemia defined
- Body composition and lipid deposition
  - Pancreatic fat deposition (MRS)
  - Hepatic fat deposition (MRS)
  - Visceral abdominal fat mass
  - Body composition (body weight, body mass index, lean body mass, total fat mass)
- Clinical, functional, metabolic and urinary metabolic markers of mechanisms
  - Urinary systemic markers of oxidative stress (8-oxo-Guo and 8-oxo-dG)
  - Circulating advanced glycation end-products (AGE),
  - Circulating receptor for AGE (sRAGE)
  - Circulating inflammatory markers (high sensitive C-reactive protein, interferon-ϒ, IL-10, IL-8, IL-6, IL1, TNFα).
  - Haemoglobin 1Ac (HbA1c)
  - Lipoedema (total cholesterol, Triglyceride, low and high density lipoprotein)
  - Blood pressure (Resting systolic and diastolic blood pressure)
  - Glycemic control during mixed meal tolerance test (iAUC, tAUC of glucose, insulin, glucagon and C-peptide)
  - Circulating markers of appetite regulation
  - Rate of gastric emptying
- Physical fitness
  - Maximal aerobic capacity (VO_2 peak)
  - One repetition maximum (RM) strength
- Patient reported outcomes
  - Physical and mental well-being
  - Quality of life
  - Satiety

Exploratory outcomes

- Outcomes from muscle and fat biopsies
  - Mitochondrial density
  - Mitochondrial function

  - mRNA
  - Protein expression
  - Inflammation
  - Metabolic signalling
- Metabolomics and proteomics from urine and blood samples
- Circulating biomarkers of organ and/or arterial function

Sample size considerations

Based on a previous study with an aerobic exercise volume similar to the guidelines (moderate dose group) in a T2D with short T2D duration, it is expected that an exercise intervention will increase late-phase disposition index derived from a hyperglycemic clamp by 1.5 (au.) more than the control group, with a standard deviation of 1.5 (au.) of the change in the exercise and 1.0 (au.) in the control group[41]. For a contrast in a one-way ANOVA with four means (1.5, 1, 0.5, 0) and contrast coefficients (1, 0, 0, -1) using a two-sided significance level of 0.05, assuming an error standard deviation of 1.5 and a balanced design, a total sample size of 80 yield a power of 0.877. Thus, to obtain a sufficient statistical power up to 20 participants are recruited per group (N=80 in total).

Recruitment

Recruitment is initiated upon approval from the Regional Scientific Committee and is open until N=80 T2D patients are randomly allocated or until 01-12-2021, whichever is reached first. To prevent screen failures, participants are recruited and screened for eligibility in three separate steps (described below).

Step 1 Individuals will be recruited in collaboration with Center for Diabetes, Municipality of Copenhagen, Steno Diabetes Center Sealand (contact person Director Lise Tarnow), The Danish Diabetes Association (Diabetesforeningen) and through advertisements in media (e.g. newspaper, television, radio), at social media (e.g. facebook), and by the internet, posters, and flyers in local areas. Interested persons will contact the project investigators by either the CFAS/CIM website, email, mail or by phone. Moreover, persons who have voluntarily registered in the MediCollect database and meet an overall set of inclusion criteria (according to the protocol inclusions criteria regarding diagnosis and age. Additionally potential participants greater Copenhagen area will be selected as test and training will be conducted in Copenhagen) will receive a questionnaire (see appendix 6) to pre-screen them for eligibility. Briefly about the Medicollect screening process; persons in the Medicollect database have voluntarily registered in the database and have consented to receive information about research projects with the prospect of enrolment and that their contact information is forwarded to the research project upon a match. Please see an elaborate method description of the Medicollect screening procedures in the appendix 7. Contact information from persons who complete the questionnaire, who is interested in participation in the study and fit the pre-screen criteria will be forwarded to the project investigators and will undergo the screening procedures outlined below.

For all persons will then receive "information for participants" by mail with an encouraging note to read the material within two days. The project investigators will, by phone, give oral information about the study, ask whether the subject has any question to the written or oral information, and perform a screening of the eligibility criteria. All possible participants will be informed about the possibility of inviting a private counsellor to an information meeting and all possible participants have the possibility to reconsider participation for at least 24 hours before signing the informed consent. If the interested person is eligible for inclusion and oral and written informed consent is provided, the person is included and a blood and urine screening is booked at the laboratory (step 2) and the baseline screening visits (visit 1-3) are planned. The

person will be informed about the possibility to contact the responsible study personnel in case of questions and information from the phone screening will not be used for later evaluation in the project.
If inclusion criteria are not met, the person is informed hereof and the process is discontinued.
If an information meeting is inquired it will take place in a closed room with only the project investigator, the potential participant and potentially the private counsellor will be present. Once again, the interested persons will be given the chance to consider engagement in the study before signing the informed consent. Following this procedure, the person is included in the study.

Step 2 Included participants are invited to Rigshospitalet (department of clinical biochemistry, 5001) for an initial blood test and urine screening. The blood- and urine test are performed to identify potential exclusion criteria not identified in step 1. In theory, this screening can also identify unknown disease (e.g. kidney, liver or thyroid disease). In such case the participant will be excluded and referred to his/her GP.

Step 3 Medical history and medical examination will be conducted at the beginning of the first baseline screening visit at CFAS (visit 1). Medical history will be obtained based on a relevant screening program and physical examination including electrocardiogram (ECG), blood pressure measurement, cardio-pulmonary stethoscopy and foot examination will be performed by a MD. If the medical history or examination reveals contraindications for further participation the participant will be inform hereof and excluded. Following this step, the participant will undergo baseline screenings and receive allocation.

Allocation

Participants are randomly allocated following successful completion of the baseline measurements. An independent statistician generates a computer-generated randomization schedule in a ratio of 1:1:1:1 stratified by sex. In order to ascertain concealment, the block sizes will not be disclosed. The schedule is forwarded to a secretary not involved in any study procedures and stored on a password protected computer. Following the baseline measurements, the participants are given consecutive numbers which is forwarded to the secretary who subsequently returns the corresponding allocation to a study nurse not involved with any testing procedures. The study nurse will then assign the allocation to the participant in person.

Blinding

Participants are blinded for treatment allocation until group assignment. However, following the baseline assessment blinding of the participants is no longer possible. All study personal involved with data collection will be blinded throughout the study. Participants are not allowed to discuss group allocation while participating in the assessments. The endocrinologist assessing the need for medication and safety will be blinded to allocation. The clinical values will be presented to the endocrinologist by a study nurse without disclosing treatment allocation.

To obtain a blinded outcome assessment the following procedure is enforced. Upon completion of the study and prior to breaking the allocation code, a data-collection form is generated by a statistician (RC) and the PI (MR-L). The data-analyst breaks the allocation code and labels the participants according to the assigned treatment and analyzes the outcomes. Following the analyses, group allocation will be concealed in all data outputs and the N per group and present the data to the writing committee in a blinded fashion. Then the writing committee will provide their blinded interpretations.

Emergency un-blinding

As all necessary information about intervention, medical history and adverse events can be provided to the endocrinologist by the study nurse, the blinding of the endocrinologist can be repealed if considered necessary.

Data collection, management and analysis

The participant timeline for outcomes assessment are described in Table 3 and the procedures are described below.

**Table 3 Participant timeline**										
	Week	-3	-2	-1	-1	4	12	17	18	18
	Appropriate staff	Visit 0	Visit 1	Visit 2	Visit 3	Visit 4	Visit 5	Visit 6	Visit 7	Visit 8
**Primary outcome**										
Hyperglycemic clamp	Study MD			X					X	
**Secondary and exploratory outcomes**										
Clinical examination and history	Study MD		X					X		
Clinical blood sampling	Study nurse	X	X			X	X	X		
Urine sampling	All team members	X	X			X	X	X		
Mixed meal tolerance test	Study nurse		X					X		
Continous glucose monitoring	All team members		X					X		
Muscle and fat biopsies	Study MD			X					X	
Cardio-vascular procedures										
Home blood pressure	All team members		X			X	X	X		
Body composition										
Magnetic resonsance imaging	Radiologist				X					X
Magnetic resonsance spectroscopy	Radiologist				X					X
Dual-energy x-ray absorptiometry	All team members		X					X		
Physical function										
Cardio-respiratory fitness	All team members		X	X				X		
Muscular strengt	All team members		X					X		
Physical activity behavior	All team members		X			X	X	X		
Patient reported outcomes										
Mental and physical well-being	All team members			X					X	

Quality of life	All team members			X					X	
Satiety	Study nurse		X					X		
Dietary records	All team members		X			X	X	X		

MD: Medical doctor, CO: Carbon mono-oxide

Data collection methods

Training and certification plans: All test personal will receive extensive training in all relevant standard operating procedures. Moreover, relevant staff will receive training and certification in DXA scanning. The training will be organized and executed by expert staff members.

Primary outcome

The hyperglycemic clamp

For the 3-stage hyperglycemic clamp an antecubital venous catheter is placed for infusion. For blood sampling a retrograde venous catheter is placed in the contralateral hand and kept warm with a heating blanket. After baseline blood sampling (t=-120 min.) a primed, continuous [6,6-^{2}H2]glucose infusion and a primed, continuous palmitate and glycerol infusion is started and maintained throughout the duration of the clamp in order to assess rate of appearance and disappearance of glucose and FFA during the procedure.

At t=0 the hyperglycaemic stage is initiated. We aim to increase baseline glucose concentration by 5.4mM above fasting level by a square-ware glucose infusion lasting 15 min. After this, glucose concentration will be kept constant and glucose infusion rates (GIR) will be adjusted based on blood glucose measurements (ABL 8 series, Radiometer, Denmark) obtained every 5th min according to an automated algorithm based on[41]. At t=120 min, the hyperglycemic+GLP1 stage is commenced by infusing a primed (0.2 pmol/kg), continuous GLP-1 infusion mimicking postprandial levels in healthy individuals. At t=240 the arginine stage is commenced with injection of arginine hydrochloride (5g given over 30 s) to assess maximal insulin secretory capacity. At t=280 min, the clamp is terminated. Following the termination, participants will be monitored with blood glucose measurements for 30-60 min into to safe recovery.

Secondary and explorative outcomes

Clinical examination, clinical blood, and urine sampling

A medical history and examination (vital parameters (HR, BP), weight, height, waist and hip circumference, ECG) will be measured by standard procedures. Moreover, socio-demographic information; education, age, ethnicity, civil status, occupation, smoking status and alcohol consumption will be collected. Blood sampling will be conducted at all visits by standard procedures. The blood samples (25 ml) will be analysed immediately for serum concentration of cholesterols, triglycerides, glucose, C-peptide, insulin and HbA1c, haematology, electrolytes, metabolites, liver- and renal status, endocrinology (including HCG if relevant) at Clinical Biochemical department 3011 (Rigshospitalet). Participants are asked to collect urine samples throughout the study. They will be provided a spot urine-sampling kit to use at home prior to visit 1, 4, 6 and 7. The samples should be obtained no more than 2 days before the visit and frozen immediately after collection. Urine will be used to measure 8-oxo-Guo and 8-oxo-dG using a validated method of ultra-performance liquid chromatography and tandem mass spectrometry[84].

Mixed meal tolerance test
To evaluate the postprandial glucose metabolism and gastric emptying time a standard 3-hour mixed meal tolerance test (MMTT) with the addition of 1.5 g paracetamol will be performed at baseline and after the training intervention. Serial blood samples will be drawn at baseline, at 0, 15, 30, 60, 90, 120, 150, 180 min after intake of 360 ml of a liquid meal (E%: 55/30/15, respectively carbohydrate/fat/protein). Blood samples will be collected in relevant tubes and analyzed for GLP-1 (active and total), glucagon, GIP, Leptin, Ghrelin, PYY, insulin, pro-insulin, C-peptide, glucose, and rate of gastric emptying. The rate of gastric emptying will be calculated as previously reported[85].

Continuous glucose monitoring (CGM)
A blinded FreeStyle Libre (Abbott, United Kingdom) will be inserted in the subcutaneous adipose tissue on the lower part of the abdomen (under the umbilicus). Blinded CGM devices are considered minimally invasive. Enzyme-coated electrodes are used to measure interstitial glucose concentrations. Using an applicator device, a thin plastic sensor is inserted just under the skin of the upper arm. The information stored in the receiver is converted into estimated profiles of glucose. The receiver can store information for up to 14 days. The values are downloaded in the laboratory and stored for later analyses and calculation of outcome measures.

Biopsies
The muscle biopsy (approx. 2-300 mg, will be obtained with Bergstrøm needle from m. vastus lateralis. 5 ml of lidocaine (20 mg/ml) will be administrated as local anaesthetic before the biopsy. The wound will be closed with patches and is expected to heal within days. Immediately after the biopsy, we will isolate muscle progenitor cells. After isolation we will use immunomagnetic sorting (MACS) of CD56+ cells to ensure that the cell population is a pure fraction of myoblasts. Single cell capture, specific reverse transcription of miRNAs and cDNA pre-amplification will be performed using the Fluidigm® C1™ System.

The abdominal subcutaneous fat biopsies (approx. 1-200 mg), will also be obtained by Begstrøms needle from the subcutaneous adipose tissue on the abdomen after administrating 2 ml lidocain (20 mg/ml) as local anaesthetic. The wounds will be closed with patches and is expected to heal within days. Qualified health professionals will take the biopsies under sterile conditions.
Subjects are only allowed to receive acetylsalicylic acid (e.g. Magnyl) or dipyridamol as antiplatelet therapy and do not have to pause their treatment prior to biopsy.
Thus, subjects taking anticoagulants or other blood thinners will not be offered muscle – or fat biopsies due to risk of bleeding. On the day that the biopsies are taken, the subjects will be handed a physical – and easy-read pamphlet where a plan of action is described should there be signs of infection or bleeding.

Body composition
Abdominal Magnetic Resonance Imaging (MRI) and Magnetic Resonance Spectroscopy (MRS)
MRI and MRS were performed using a Siemens Magnetom Prisma 3 Tesla matrix magnetic resonance scanner (Erlangen, Germany) at 3 mm intervals. All adipose tissue located from the diaphragm to pelvic floor inside the peritoneum is traced manually as the visceral fat region of interest. MRS to assess liver and pancreatic fat is performed based on the MRI and analyzed as described elsewhere[86]. The MRI will be performed at Rigshospitalet (dept. 3024) by a trained radiologist. Relevant software will be used for post-processing. All MR scans will be analysed in a blinded manner.

Body composition
Dual x-ray absorptiometry will be used to assess body fat and lean mass before, during and after the

intervention. To distinguish between subcutaneous adipose tissue and visceral adipose tissue in the android region, additional software (enCORE software version 13.6.) will be used.

Physical function

Maximal aerobic capacity
The participants will undergo a maximal graded exercise test on a bicycle ergometer for evaluation of cardiovascular function and determination of the peak oxygen uptake (VO2peak). The test starts with a 5 min warm up at 70 watts (the watts may have to be adjusted to the individual fitness level). Warm up is immediately followed by a 15 watt increase every 1 min until exhaustion. Ventilation and expired gases will be measured during the test via an indirect calorimetric system, and heart rate will be assessed simultaneously. From watt-max the maximal aerobic work capacity will be calculated using ACSM standard metabolic calculation. The test will be continued until the following criteria were met: plateauing of heart rate and VO_2 with incremental workloads, respiratory exchange ratio > 1.1 [87]. Oxygen consumption will be assessed using continuous indirect calorimetric measurements (CPET, Cosmed, Italy) and heart rate monitoring (Polar Electro, Holte, DK).

Muscle strength
Maximum muscle strength will be assessed in four functional exercises performed in resistance training machines (leg press, chest press, leg extension, and seated row) by 1-repetition maximum (1-RM). Participants will be positioned correctly in machine and will perform a light warm-up (8 repetitions on a light load). Following a short rest, a progressive loading procedure is performed (i.e. a higher load is selected and 1 repetition is performed, after another short rest, an even higher load is tested for 1 repetition) until the single repetition cannot be completed in good form. The load of the last successful repetition will be recorded as the 1-RM.

Physical activity
All participants will be equipped with two accelerometers (AX3, Axivity, Newcastle, UK) for 4-6 consecutive days. One accelerometer placed on the right thigh, and one accelerometer placed on the right side of the lower back. Both accelerometers will be attached on the participant with a patch (Fixomull stretch, BSN medical, Germany). Posture allocation will be determined by orientation of the thigh-mounted accelerometer, while intensity of physical activity will be determined by the accelerometer counts of the hip mounted accelerometer. For a given measurement sequence, it will be possible to determine time stamps on posture allocation, duration of sedentary periods, breaks in sedentary time, and the intensity of physical activity during breaks in sedentary time. Using the AX3 to monitor posture allocation and basic physical activity behaviour will take place four times (before, during (week 4 and 12) and after the intervention).

Patient reported outcomes
Mental and physical well-being from the Short Form 36 (SF-36)
The SF-36 is a short-form health survey with 36 questions. It yields an 8-scale profile of functional health and well-being scores as well as psychometrically-based physical and mental health summary measures and a preference-based health utility index [88].

Diet record
A self-reported three-day record of their total dietary intake will be obtained at baseline, during (at week 3, 6, 9 and 12) in the intervention period, and after the intervention. On the basis of these records the energy intake of each participant will be estimated and used to adjust the dietary intervention.

Satiety
Satiety is recorded during the MMTT (immediately before, 60 min into the test and immediately after the test) using a 5 item visual analogue scale (VAS). The participants are asked to rate immediate sensation of hunger, sensation of thirst, desire to eat, nausea and fullness.

Biological specimens and research biobank
Separate blood samples (20 mL) from the clinical examinations will be aliquoted, frozen at and stored at -80°C a project specific biobank at (CFAS) 7641 (Rigshospitalet) for future analyses of measure AGE, sRAGE, inflammatory markers, hormones and biomarkers of disease progression and organ function.
During the hyperglycemic clamp separate blood samples (232 mL mL) are aliquoted, frozen at and stored at -80°C a project specific biobank at (CFAS) 7641 (Rigshospitalet) for future analyses of for future analyses of inflammatory markers, C-peptide, insulin, glucose, paracetamol, cholesterols, triglycerides, free fatty acids, glucagon, incretins, metabolomics and proteomics. Total blood volume loss during the hyperglycemic clamp from blood samples and blood waste is estimated to be approximately 280 mL

During the MMTT separate blood samples (140 mL) are aliquoted, frozen at and stored at -80°C a project specific biobank at (CFAS) 7641 (Rigshospitalet) for future analyses of inflammatory markers, C-peptide, insulin, glucose, paracetamol, cholesterols, triglycerides, free fatty acids, glucagon, incretins, metabolomics and proteomics.

Urine samples (15 mL) are frozen at -80°C until analysis. Remaining muscle tissue (~250 mg) is immediately frozen in liquid nitrogen and stored for batch isolation of mRNA and protein. Gene expression analyses will be conducted for regulation of protein degeneration and regeneration signaling, as well as muscle specific pathways. Protein expression analyses will be performed to elucidate related pathways in muscle degeneration, regeneration and oxidative stress. Adipose tissue biopsies (100-200 mg) are immediately frozen in liquid nitrogen and stored for batch isolation of mRNA and protein. Gene expression analyses will be conducted for markers of immune cell infiltration, inflammatory cytokines, adipose tissue browning, and regulation of cellular metabolism. Similarly, protein expression analyses will be performed to elucidate browning, inflammatory and metabolic signaling.

Finally, we will ask participants to give separate consent a blood sample (10 ml) which will be frozen and stored for non-specified future research until termination of the study (2028). Before future analyses are performed of these samples, we will apply local ethical committee for approval. Any excess biological material will be stored for 20 years (01.11.2038) and then be transferred to CFAS' biobank registered with Datatilsynet (RH-2017-60 I-suite nr: 05329). Biological material will not be exchanged with researchers in other countries unless reported to and approved by the ethical committee.

Retention
All participants will receive up to DKr. 4.000 Dkr (€530) to cover lost earnings, transport and discomfort. The transaction is completed upon completion of the study (all four full laboratory days (v1, v2, v6 and v7) or upon withdrawal). For every completed full day of laboratory testing, participants will receive 1.000 DKr. Moreover, 500 Dkr. in compensation will be added per biopsy (up to 4 in total).

To prevent loss-to-follow-up amongst participants in the CON and the participants are NOT believed to benefit from the control intervention more than expect from standard care, they are offered an exercise program based on the DOSE-EX program. It will consist of supervised aerobic exercise with the same intensity and duration as described for the MOD group. It also includes a 4-month membership for Fitness World with a value of 930 kr. Moreover, they will receive a 1-hour introduction and help (group based) to initiate the training session (5 minutes for each session). This constitutes a total of 5 hours of group-based counseling (1-hour introduction, 5 min intro * 3 x week * 4 weeks * 4 months) with a value of 895 kr. (derived from Fitness Worlds listing price for group-based counseling). If the control group participants do not wish to receive this offer, they will be referred to the municipality-based rehabilitation in their resident area. All participants are allowed to contact the study nurse by phone in case of project related questions (e.g. pharmacological treatment, sports injuries etc.). All compensation is subject to taxation including the value of the Fitness World membership.

Data management

Data forms, entry, transmission, entry and editing

The web-based Clinical Trial Management System EasyTrial is used for data entry and management (EasyTrial ApS). EasyTrial has been approved by the Danish Data Protection Board. Electronic case reports forms (eCRF) and questionnaires have been generated by the sponsor in EasyTrial. Fields have been programmed with acceptable ranges for data entry. EasyTrial is also used to send reminders to the participants prior to visits and to remind participants in the exercise intervention group to upload heart rate data for supervision. During the study, data are entered directly into the system by the investigators, and after study completion data will be extracted directly from the system by the sponsor/investigators and stored behind a firewall on a secure served with logging of activity retrospectively for 6 months. The servers are backed up continuously.

All paper material (CRF, blood screen results, questionnaires and dietary records) is collected and store in a locked cabinet on CFAS, Rigshospitalet Denmark. All information from the paper material is entered (twice by in non-consecutive order) into the electronic back-end system. Consistency is checked using appropriate software. In case of discrepancies between the entries, the original paper record is consulted. Upon completion of the study all paper material is scanned and store on the secured hospital server in an electronic form. All paper material, except for the consent form, is the destroyed. Data management is performed using appropriate statistical software.

Statistical methods

Analysis of the primary outcome

The primary analysis will be based on the set of participants that is as close as possible to the intended intervention protocol (i.e. per protocol – see definition above).

The analyses of the primary outcome will be performed using mixed linear modeling with the mean change score of DI as dependent variable and group (2 levels), sex (2 levels) and the baseline value of DI as independent variables. The assumptions for using the linear models will be checked to confirm normal distribution of the residual and the homogeneity of the variance (standardized residuals vs. the predicted values).

The primary analysis is based on a hierarchical analytic approach in order to maintain the type 1 error rate. Between group comparisons for effect size estimation ((difference in change from 0-16 weeks, based on a superiority assumption) is completed in the following order;

1) CON vs. HED. If a difference is present ($p<0.05$, 2-sidet) then the next between group comparison is performed. If not – then sequence is terminated
2) CON vs. MED. If a difference is present ($p<0.05$, 2-sidet) then the next between group comparison is performed. If not – then sequence is terminated
3) CON vs. DCON. If a difference is present ($p<0.05$, 2-sidet) then the next between group comparison is performed. If not – then sequence is terminated
4) DCON vs. HED. If a difference is present ($p<0.05$, 2-sidet) then the next between group comparison is performed. If not – then sequence is terminated
5) DCON vs. MED. If a difference is present ($p<0.05$, 2-sidet) then the next between group comparison is performed. If not – then sequence is terminated
6) MED vs. HED.

The assumptions for using the linear models will be checked to confirm normal distribution of the residual and the homogeneity of the variance (standardized residuals vs. the predicted values).

Analyses of the secondary outcomes
The ITT analysis of DI (key secondary outcome) will be based on the *as-observed population* (missing data will not be imputed in the primary analysis)[89]. Thus, the participants are analyzed according to the planned treatment regimen rather than the actual treatment, i.e. irrespective of their compliance with the planned course of treatment. The 'Full Analysis Set' will thus be derived from the set of all randomized participants by minimal and justified elimination of participants. Therefore, all participants allocated to a treatment group (CON, MED or HED) will be followed up, assessed and analyzed as members of that group irrespective of their compliance to the planned course of treatment. Sensitivity analyses will be performed using *baseline observation carried forward* and multiple imputation procedures[89]. Patterns of missing data will be investigated. *A priori*, the less restrictive missing at random (MAR) assumption is considered more reasonable than the missing data be missing completely at random (MCAR). Assuming that the data on potential drop-outs are missing at random multiple imputation procedures would be applicable to handle missing data for all participants with baseline measurements.

Other continuous secondary outcomes assessed pre- and post-intervention will be analyzed analysis of covariance with the mean change score of the variable as dependent variable and group (3 levels), sex (2 levels) and the baseline value of the variable as independent variables. Continuous variables, additionally assessed during the intervention period, are analyzed within the framework of repeated-measures linear mixed models, an analysis of covariance model will be used to analyze mean changes in continuous end points. The model includes treatment (4 levels), time (3 levels), sex (2 levels), and the possible interaction between treatment (group) and time (weeks) as fixed effects, with the baseline value of the relevant variable as a covariate and participant ID as random effect. The assumptions will be investigated as described above. Variables not meeting the model assumptions will be transformed using appropriate transformations. If no suitable transformation is identified, the median change with interquartile ranges will reported and testing is performed using suitable non-parametric statistical tests (e.g. wilcoxons signed rank tests). Binary outcomes are reported as numbers and proportions and tested using a X^2 test or exact statistics.

Harms, risks and discomforts

Adverse events (AE) and safety evaluation
In this study, we have adopted the ICH definition of adverse event (AE) (E2A).

An AE is thus defined as; "*An adverse event (AE) can therefore be any unfavourable and unintended sign (including an abnormal laboratory finding, for example), symptom, or disease temporally associated with the use of a medicinal product, whether or not considered related to the medicinal product"*[90].

Serious AE (SAE) is defined as;*"[...] any untoward medical occurrence that at any dose: * results in death, * is life-threatening, NOTE: The term "life-threatening" in the definition of "serious" refers to an event in which the patient was at risk of death at the time of the event; it does not refer to an event which hypothetically might have caused death if it were more severe. * requires inpatient hospitalisation or prolongation of existing hospitalisation, * results in persistent or significant disability/incapacity, or * is a congenital anomaly/birth defect"*[90].

AEs/SAEs (anticipated and unanticipated) will be recorded on adverse event forms. These forms will include a description and classification of the event, date of onset, date resolved, whether the event was serious or not (ICH criteria), relationship of the event to the study (1=none, 2=unlikely, 3=possible, 4=probable, 5=definitely), action taken, and whether the study was suspended or not. All SAEs, will be reported to the Regional Ethical Committee by the sponsor. AEs observed by any investigator and/or reported by the participant must be reported in the source data and case report form from the first (= signature of informed consent) to the last protocol-specific procedure allocation[91].

VO_2-max test and 1RM: Physical fitness and strength tests, where subjects must put in maximal effort. The tests can cause some degree of breathlessness and exhaustion, but is standard methods used for scientific purpose at the CFAS laboratory.

DXA scan: Is not expected to cause any discomfort and involves very little radiation (0.0004mSv) corresponding to approximately 1/10 of the radiation acquired for a thoracic x-ray. The dose is smaller than received when flying in a commercial jet from (11-12 hours) (SST.dk - Strålingsguiden). The DXA scan is considered safe and is part of standard scientific testing at CFAS.

Hyperglycemic clamp: The hyperglycemic clamp combined with GLP-1 infusion and arginine bolus may cause hypoglycaemic symptoms (dizziness, headache and fatigue) following the trial. However, blood glucose will be monitored for up to 1 hour after the test and a meal will be provided when testing has finished. Furthermore, there is a minor risk of infection or hematoma due to blood lines being placed. All researchers are experts in these procedures so the risks are minimal. The hyperglycaemic clamp with GLP-1 and arginine has previously been used for scientific purpose in our laboratory. The introduction of arginine may give the participants a transient metallic taste which is short and fully reversible.
Stable isotope tracers will be used to obtain knowledge about glucose and lipid distribution. The isotopes are not radioactive and are considered safe to use. They have previously been used in the CFAS laboratory and we do not expect any additional discomfort than the ones already described according to the clamp.

Blood sampling: A small peripheral venous catheter (PVS) will be placed and can cause slight discomfort and a small risk of local infection and edema. The blood volume collected (maximum 965 ml/4 months) is considered too small to cause any symptoms.

Biopsies: Prior to the muscle biopsy local anaesthesia will be administered, which is associated with short-lasting discomfort. After a few minutes, the area is numbed and a small skin incision is made. The Bergstrom cannula is inserted in M. vastus lateralis and the biopsy is taken. This part of the procedure might be associated with slight pain. The procedure takes a few minutes and after the biopsy is taken, the wound will be closed with Steri-Strips, which must be kept on for five days. There will be an additional patch, which can be removed after two days. Subjects might experience some degree of muscle pain after the biopsy. Paracetamol (1000 mg) max. four times a day will be recommended as pain killers. The fat biopsy (subcutaneous adipose tissue from the abdomen) is taken following the same procedure; these biopsies in general cause much less discomfort. Complications are rare and usually mild. The procedures can leave small bruises, but generally heals nicely. Temporary decreased sensation at the incision area or where the local anaesthetic has been injected can be seen. Usually, nerve lesions and altered sensation at the incision area heals within months. In theory, infections can appear when piercing the skin. This occurs in 1 out of 25,000 times and can in some cases require treatment with antibiotics. In order to avoid infection all participants will be informed to abstain from swimming in seawater or swimming pools within the first five days. Furthermore, they will be informed to contact a project doctor in case of any signs of infection (heat, redness, swelling or fever).

MRI/MRS: The measurement is pain free. It is based on radio waves and thus the participant is not exposed to x-rays or other sources of radiation. The scan is performed in a tight cylinder which may cause claustrophobia with some participants. The scans are not performed with a specific clinical purpose but rather for quantification of site-specific ectopic adipose tissue. It cannot and will not be used for diagnostic purposes. However, all scans will be screened by a trained radiologist and therefore unexpected abnormalities may be detected. If deemed necessary, the department of radiology (Rigshospitalet, dept. 3024) will help perform further diagnostics if the participant so wishes (according to the consent form).

Continuous glucose monitoring (CGM): The method is safe and routinely used by diabetic patients to continuously monitor the blood glucose level. A tiny electrode (glucose sensor) will be inserted to the subcutaneous tissue at the upper arm. To this end, there is a small risk of infection and the study participants will be informed and instructed to take action in case of symptoms of infection.

Ethics and dissemination

The study is expected to result in minimal discomfort and risk and the participants can discontinue their engagement at any time and without any reason. The study examines the effects of various volumes of physical activity and exercise on glucose levels and pancreatic β-cell function in participants with shorter term T2D. Both a rapid weight loss through diet and diet/physical activity may induce subjective signs of hypo-glycemia. Thus, procedures to adjust medications in both the Look AHEAD and direct DIRECT studies are described[58,92]. It is thus reasonable to adjust glucose and BP lowering agents with a safety mechanism in a blinded standardized algorithm in all groups. Based on a previous study a large proportion of the exercise/diet groups are expected to discontinue their glucose-lowering and BP lowering medications within the study period without any adverse events[22]. Immediately following the study the clinical parameters are reviewed by the research physicians and the participants are asked to contact their GP with the purpose of continuing their treatment based on the clinical guidelines[81]. The participants receiving DCON/MED/HED groups will benefit from the study in terms of thorough medical examination and increased physical capacity and increased T2D management. Based on previous research from our group, it is expected that a large proportion will maintain or even improve T2D control with this intervention[70]. Moreover, in contrast to the previous study, all exercise

session are fully supervised, thus it is expected that compliance to the lifestyle intervention is even higher than previously reported (82%)[70]. The control group will also benefit from an extensive health checkup and achieve insight to basic anti-diabetic lifestyle alterations. After the project has finished all participants will be re-referred to the various activities for patients with type 2 diabetes in their local municipalities . If participants in the control group do not wish or is unable to attend the rehabilitation program, then a dietary plan and an extensive individualized training program (based on the intervention provided in the (DCON/MED/HED groups) will be provided.

The study is important, valuable, sound, and will contribute to the essential knowledge of possible T2D remission induced by non-surgical and non-pharmacological lifestyle intervention. Specifically, this study will improve knowledge about if and how exercise intervention, when administered in concert with diet-induced weight loss may revert of pancreatic β-cell function in patients with T2D and possibly provide a sound alternative to conventional high-risk procedures. Moreover, the study will elucidate the time dependency and causality between the pathophysiological processes of cardiovascular damage and add to the knowledge about how and if exercise intervention will decrease the risk of the micro- and macro vascular complications induced by T2D. In the end the project will be a crucial stepping stone in the process of developing efficient lifestyle interventions with both a curative and secondary prevention purposes in the clinical care of T2D

Participants may withdraw with no obligation to provide a reason. Participants may be withdrawn from the study prior to the expected completion of the study if steering committee decides to discontinue the study (possible at any time for any reason) or the clinical condition of the participant is such that the investigator recommends withdrawal.

Ethical approval will be applied at the Scientific Ethical Committee at the Capital Region of Denmark and the study will be conducted in accordance with the Declaration of Helsinki (1964) with its subsequent revisions. No study procedures (including recruitment procedures) will be initiated until ethical approval has been obtained.

Protocol amendments

Changes to the protocol can be induced by scientific rationale or evidence of potential harms/risks of the intervention or data collection methods. Potential amendments have to be accepted by the DOSE-EX scientific committee before submission to The Scientific Ethical Committee of the Capitol Region of Copenhagen. No amendments to the protocol will be implemented until approval by the ethical committee.

Consent or assent

Trained research personal will provide oral and written information about the project to all possible participants. On basis of an informed discussion the research personal will obtain written and oral informed consent from the participants willing to be part of the trial.

Confidentiality

To anonymize data all participants will be ascribed a unique participant identification (ID) number. The identification key (ID number to personal information) on a password protected computer separate from the unique ID number and the database. All local databases will be secured with passwords and logged. Printed data will be kept in a separate locked area with limited access. All patient-related information obtained during the study will be handled in accordance with the Danish law for protection of personal data (“lov om behandling af personoplysninger”) and the Danish health law (“sundhedsloven”). Information will be obtained

from patient journals in relation to blood samples that are drawn as part of this study and analyzed by the biochemical department at Rigshospitalet. Moreover, information about medications prescriptions and redemption from inclusion and until completion or discontinuation of the study will be obtained from the patient journals. The blood samples include screening for side effects during the study and a broad medical blood screening in the beginning and end of the study. The blood samples will be registered from the hospital blood sample portal (Labka) and para-clinical observations will be obtained through "Sundhedsportalen". Upon ethical approval, the study will be submitted to the Danish Data Protection Agency ("Region H's paraplyanmeldelse") for approval.

Declaration of interests

The authors declare no conflicts of interest. The funding agency (Trygfonden) has not taken part in protocol drafting and will not take part in completion of the study, data collection nor interpretation or publishing of the data from this trial.

Access to data

All data is the property of CFAS and access to data is overseen by the DOSE-EX steering committee. All members of the DOSE-EX study group have access to the anonymized cleaned dataset upon completion of the final post-intervention testing upon approval from the steering committee (based on an approved proposal or with specific reason provided). Following publication of the primary outcome, other research may request access to data following an approved (by the steering committee) research proposal.

Ancillary and post-trial care

Participants enrolled in the study will be covered by Patienterstatningsordningen.

Dissemination policy

The data from the DOSE-EX study will be published in international peer-reviewed journals. All results will be reported according to the CONSORT guidelines [93]. Positive, negative and inconclusive data will all be disseminated and published.

Principal investigators and other senior members of the steering committee are considered lead authors of the material derived from the project. All authors must comply with the "Uniform Requirements for Manuscripts Submitted to Biomedical Journals" [94].

Informed consent materials:

Prior to inclusion in the study written and oral informed consent. Please see ***Appendix 5 for the consent form.***

1. Colberg SR, Sigal RJ, Fernhall B, et al. Exercise and type 2 diabetes: the American College of Sports Medicine and the American Diabetes Association: joint position statement. *Diabetes care.* 2010;33(12):e147-167.
2. Colberg SR, Sigal RJ, Yardley JE, et al. Physical Activity/Exercise and Diabetes: A Position Statement of the American Diabetes Association. *Diabetes care.* 2016;39(11):2065-2079.

3. Sluik D, Buijsse B, Muckelbauer R, et al. Physical Activity and Mortality in Individuals With Diabetes Mellitus: A Prospective Study and Meta-analysis. *Archives of internal medicine.* 2012;172(17):1285-1295.
4. Tanasescu M, Leitzmann MF, Rimm EB, Hu FB. Physical activity in relation to cardiovascular disease and total mortality among men with type 2 diabetes. *Circulation.* 2003;107(19):2435-2439.
5. Hu FB, Stampfer MJ, Solomon C, et al. Physical activity and risk for cardiovascular events in diabetic women. *Annals of internal medicine.* 2001;134(2):96-105.
6. Sone H, Tanaka S, Tanaka S, et al. Leisure-time physical activity is a significant predictor of stroke and total mortality in Japanese patients with type 2 diabetes: analysis from the Japan Diabetes Complications Study (JDCS). *Diabetologia.* 2013;56(5):1021-1030.
7. Action to Control Cardiovascular Risk in Diabetes Study G, Gerstein HC, Miller ME, et al. Effects of intensive glucose lowering in type 2 diabetes. *The New England journal of medicine.* 2008;358(24):2545-2559.
8. Group AC, Patel A, MacMahon S, et al. Intensive blood glucose control and vascular outcomes in patients with type 2 diabetes. *The New England journal of medicine.* 2008;358(24):2560-2572.
9. Duckworth W, Abraira C, Moritz T, et al. Glucose control and vascular complications in veterans with type 2 diabetes. *The New England journal of medicine.* 2009;360(2):129-139.
10. Reusch JE, Manson JE. Management of Type 2 Diabetes in 2017: Getting to Goal. *JAMA : the journal of the American Medical Association.* 2017;317(10):1015-1016.
11. Hemmingsen B, Lund SS, Gluud C, et al. Intensive glycaemic control for patients with type 2 diabetes: systematic review with meta-analysis and trial sequential analysis of randomised clinical trials. *BMJ (Clinical research ed.).* 2011;343:d6898.
12. Intensive blood-glucose control with sulphonylureas or insulin compared with conventional treatment and risk of complications in patients with type 2 diabetes (UKPDS 33). UK Prospective Diabetes Study (UKPDS) Group. *Lancet.* 1998;352(9131):837-853.
13. Lipska KJ, Krumholz HM. Is Hemoglobin A1c the Right Outcome for Studies of Diabetes? *JAMA : the journal of the American Medical Association.* 2017;317(10):1017-1018.
14. Umpierre D, Ribeiro PA, Kramer CK, et al. Physical activity advice only or structured exercise training and association with HbA1c levels in type 2 diabetes: a systematic review and meta-analysis. *JAMA : the journal of the American Medical Association.* 2011;305(17):1790-1799.
15. Boule NG, Kenny GP, Haddad E, Wells GA, Sigal RJ. Meta-analysis of the effect of structured exercise training on cardiorespiratory fitness in Type 2 diabetes mellitus. *Diabetologia.* 2003;46(8):1071-1081.
16. Avery L, Flynn D, van Wersch A, Sniehotta FF, Trenell MI. Changing physical activity behavior in type 2 diabetes: a systematic review and meta-analysis of behavioral interventions. *Diabetes care.* 2012;35(12):2681-2689.
17. Umpierre D, Ribeiro PA, Schaan BD, Ribeiro JP. Volume of supervised exercise training impacts glycaemic control in patients with type 2 diabetes: a systematic review with meta-regression analysis. *Diabetologia.* 2013;56(2):242-251.
18. Boule NG, Haddad E, Kenny GP, Wells GA, Sigal RJ. Effects of exercise on glycemic control and body mass in type 2 diabetes mellitus: a meta-analysis of controlled clinical trials. *JAMA : the journal of the American Medical Association.* 2001;286(10):1218-1227.
19. Lean ME, Leslie WS, Barnes AC, et al. Primary care-led weight management for remission of type 2 diabetes (DiRECT): an open-label, cluster-randomised trial. *Lancet.* 2018;391(10120):541-551.
20. Gregg EW, Chen H, Wagenknecht LE, et al. Association of an intensive lifestyle intervention with remission of type 2 diabetes. *JAMA : the journal of the American Medical Association.* 2012;308(23):2489-2496.
21. Courcoulas AP, Belle SH, Neiberg RH, et al. Three-Year Outcomes of Bariatric Surgery vs Lifestyle Intervention for Type 2 Diabetes Mellitus Treatment: A Randomized Clinical Trial. *JAMA Surg.* 2015;150(10):931-940.

22. Johansen MY, MacDonald CS, Hansen KB, et al. Effect of an Intensive Lifestyle Intervention on Glycemic Control in Patients With Type 2 Diabetes: A Randomized Clinical Trial. *JAMA : the journal of the American Medical Association.* 2017;318(7):637-646.
23. Ried-Larsen M, Christensen R, Hansen KB, et al. Head-to-head comparison of intensive lifestyle intervention (U-TURN) versus conventional multifactorial care in patients with type 2 diabetes: protocol and rationale for an assessor-blinded, parallel group and randomised trial. *BMJ open.* 2015;5(12):e009764.
24. Kahn SE, Prigeon RL, McCulloch DK, et al. Quantification of the relationship between insulin sensitivity and beta-cell function in human subjects. Evidence for a hyperbolic function. *Diabetes.* 1993;42(11):1663-1672.
25. Taylor R. Insulin resistance and type 2 diabetes. *Diabetes.* 2012;61(4):778-779.
26. Defronzo RA. Banting Lecture. From the triumvirate to the ominous octet: a new paradigm for the treatment of type 2 diabetes mellitus. *Diabetes.* 2009;58(4):773-795.
27. Rudenski AS, Hadden DR, Atkinson AB, et al. Natural history of pancreatic islet B-cell function in type 2 diabetes mellitus studied over six years by homeostasis model assessment. *Diabetic medicine : a journal of the British Diabetic Association.* 1988;5(1):36-41.
28. Zhou YP, Grill V. Long term exposure to fatty acids and ketones inhibits B-cell functions in human pancreatic islets of Langerhans. *The Journal of clinical endocrinology and metabolism.* 1995;80(5):1584-1590.
29. Briaud I, Harmon JS, Kelpe CL, Segu VB, Poitout V. Lipotoxicity of the pancreatic beta-cell is associated with glucose-dependent esterification of fatty acids into neutral lipids. *Diabetes.* 2001;50(2):315-321.
30. Donath MY. Targeting inflammation in the treatment of type 2 diabetes: time to start. *Nat Rev Drug Discov.* 2014;13(6):465-476.
31. Donath MY, Shoelson SE. Type 2 diabetes as an inflammatory disease. *Nat Rev Immunol.* 2011;11(2):98-107.
32. Adiels M, Taskinen MR, Packard C, et al. Overproduction of large VLDL particles is driven by increased liver fat content in man. *Diabetologia.* 2006;49(4):755-765.
33. Taylor R. Type 2 diabetes: etiology and reversibility. *Diabetes care.* 2013;36(4):1047-1055.
34. Larsen CM, Faulenbach M, Vaag A, Ehses JA, Donath MY, Mandrup-Poulsen T. Sustained effects of interleukin-1 receptor antagonist treatment in type 2 diabetes. *Diabetes care.* 2009;32(9):1663-1668.
35. Lim EL, Hollingsworth KG, Aribisala BS, Chen MJ, Mathers JC, Taylor R. Reversal of type 2 diabetes: normalisation of beta cell function in association with decreased pancreas and liver triacylglycerol. *Diabetologia.* 2011;54(10):2506-2514.
36. White MG, Shaw JA, Taylor R. Type 2 Diabetes: The Pathologic Basis of Reversible beta-Cell Dysfunction. *Diabetes care.* 2016;39(11):2080-2088.
37. Taylor R. Pathogenesis of type 2 diabetes: tracing the reverse route from cure to cause. *Diabetologia.* 2008;51(10):1781-1789.
38. Taylor R, Leslie WS, Barnes AC, et al. Clinical and metabolic features of the randomised controlled Diabetes Remission Clinical Trial (DiRECT) cohort. *Diabetologia.* 2018;61(3):589-598.
39. Sylow L, Kleinert M, Richter EA, Jensen TE. Exercise-stimulated glucose uptake - regulation and implications for glycaemic control. *Nat Rev Endocrinol.* 2017;13(3):133-148.
40. Way KL, Hackett DA, Baker MK, Johnson NA. The Effect of Regular Exercise on Insulin Sensitivity in Type 2 Diabetes Mellitus: A Systematic Review and Meta-Analysis. *Diabetes Metab J.* 2016;40(4):253-271.
41. Karstoft K, Winding K, Knudsen SH, et al. Mechanisms behind the superior effects of interval vs continuous training on glycaemic control in individuals with type 2 diabetes: a randomised controlled trial. *Diabetologia.* 2014;57(10):2081-2093.
42. Dela F, von Linstow ME, Mikines KJ, Galbo H. Physical training may enhance beta-cell function in type 2 diabetes. *American journal of physiology. Endocrinology and metabolism.* 2004;287(5):E1024-1031.

43. Rogers MA, Yamamoto C, King DS, Hagberg JM, Ehsani AA, Holloszy JO. Improvement in glucose tolerance after 1 wk of exercise in patients with mild NIDDM. *Diabetes care.* 1988;11(8):613-618.
44. Krotkiewski M, Lonnroth P, Mandroukas K, et al. The effects of physical training on insulin secretion and effectiveness and on glucose metabolism in obesity and type 2 (non-insulin-dependent) diabetes mellitus. *Diabetologia.* 1985;28(12):881-890.
45. Eriksen L, Dahl-Petersen I, Haugaard SB, Dela F. Comparison of the effect of multiple short-duration with single long-duration exercise sessions on glucose homeostasis in type 2 diabetes mellitus. *Diabetologia.* 2007;50(11):2245-2253.
46. Hannon TS, Kahn SE, Utzschneider KM, et al. Review of methods for measuring beta-cell function: Design considerations from the Restoring Insulin Secretion (RISE) Consortium. *Diabetes, obesity & metabolism.* 2018;20(1):14-24.
47. Malin SK, Solomon TP, Blaszczak A, Finnegan S, Filion J, Kirwan JP. Pancreatic beta-cell function increases in a linear dose-response manner following exercise training in adults with prediabetes. *American journal of physiology. Endocrinology and metabolism.* 2013;305(10):E1248-1254.
48. Sigal RJ, Kenny GP, Boule NG, et al. Effects of aerobic training, resistance training, or both on glycemic control in type 2 diabetes: a randomized trial. *Annals of internal medicine.* 2007;147(6):357-369.
49. Church TS, Blair SN, Cocreham S, et al. Effects of aerobic and resistance training on hemoglobin A1c levels in patients with type 2 diabetes: a randomized controlled trial. *JAMA : the journal of the American Medical Association.* 2010;304(20):2253-2262.
50. Haus JM, Solomon TP, Kelly KR, et al. Improved hepatic lipid composition following short-term exercise in nonalcoholic fatty liver disease. *The Journal of clinical endocrinology and metabolism.* 2013;98(7):E1181-1188.
51. Rabol R, Petersen KF, Dufour S, Flannery C, Shulman GI. Reversal of muscle insulin resistance with exercise reduces postprandial hepatic de novo lipogenesis in insulin resistant individuals. *Proceedings of the National Academy of Sciences of the United States of America.* 2011;108(33):13705-13709.
52. Sondergaard E, Rahbek I, Sorensen LP, et al. Effects of exercise on VLDL-triglyceride oxidation and turnover. *American journal of physiology. Endocrinology and metabolism.* 2011;300(5):E939-944.
53. Heiskanen MA, Motiani KK, Mari A, et al. Exercise training decreases pancreatic fat content and improves beta cell function regardless of baseline glucose tolerance: a randomised controlled trial. *Diabetologia.* 2018.
54. Benatti FB, Pedersen BK. Exercise as an anti-inflammatory therapy for rheumatic diseases-myokine regulation. *Nat Rev Rheumatol.* 2015;11(2):86-97.
55. Karstoft K, Pedersen BK. Exercise and type 2 diabetes: focus on metabolism and inflammation. *Immunol Cell Biol.* 2016;94(2):146-150.
56. Solomon TP, Malin SK, Karstoft K, Kashyap SR, Haus JM, Kirwan JP. Pancreatic beta-cell function is a stronger predictor of changes in glycemic control after an aerobic exercise intervention than insulin sensitivity. *The Journal of clinical endocrinology and metabolism.* 2013;98(10):4176-4186.
57. Solomon TP, Malin SK, Karstoft K, Haus JM, Kirwan JP. The influence of hyperglycemia on the therapeutic effect of exercise on glycemic control in patients with type 2 diabetes mellitus. *JAMA internal medicine.* 2013;173(19):1834-1836.
58. Leslie WS, Ford I, Sattar N, et al. The Diabetes Remission Clinical Trial (DiRECT): protocol for a cluster randomised trial. *BMC Fam Pract.* 2016;17:20.
59. Ryan DH, Espeland MA, Foster GD, et al. Look AHEAD (Action for Health in Diabetes): design and methods for a clinical trial of weight loss for the prevention of cardiovascular disease in type 2 diabetes. *Controlled clinical trials.* 2003;24(5):610-628.
60. Manigrasso MB, Juranek J, Ramasamy R, Schmidt AM. Unlocking the biology of RAGE in diabetic microvascular complications. *Trends Endocrinol Metab.* 2014;25(1):15-22.

61. Broedbaek K, Siersma V, Henriksen T, et al. Association between urinary markers of nucleic acid oxidation and mortality in type 2 diabetes: a population-based cohort study. *Diabetes care.* 2013;36(3):669-676.
62. Ridker PM, Everett BM, Thuren T, et al. Antiinflammatory Therapy with Canakinumab for Atherosclerotic Disease. *The New England journal of medicine.* 2017;377(12):1119-1131.
63. Monnier L, Mas E, Ginet C, et al. Activation of oxidative stress by acute glucose fluctuations compared with sustained chronic hyperglycemia in patients with type 2 diabetes. *JAMA : the journal of the American Medical Association.* 2006;295(14):1681-1687.
64. Ceriello A, Esposito K, Piconi L, et al. Oscillating glucose is more deleterious to endothelial function and oxidative stress than mean glucose in normal and type 2 diabetic patients. *Diabetes.* 2008;57(5):1349-1354.
65. Karstoft K, Clark MA, Jakobsen I, et al. The effects of 2 weeks of interval vs continuous walking training on glycaemic control and whole-body oxidative stress in individuals with type 2 diabetes: a controlled, randomised, crossover trial. *Diabetologia.* 2017;60(3):508-517.
66. Brownlee M. The pathobiology of diabetic complications: a unifying mechanism. *Diabetes.* 2005;54(6):1615-1625.
67. Ceriello A, Ihnat M. Oxidative stress is, convincingly, the mediator of the dangerous effects of glucose variability. *Diabetic medicine : a journal of the British Diabetic Association.* 2010;27(8):968.
68. Chilelli NC, Burlina S, Lapolla A. AGEs, rather than hyperglycemia, are responsible for microvascular complications in diabetes: a "glycoxidation-centric" point of view. *Nutrition, metabolism, and cardiovascular diseases : NMCD.* 2013;23(10):913-919.
69. Pedersen BK. Anti-inflammatory effects of exercise: role in diabetes and cardiovascular disease. *European journal of clinical investigation.* 2017;47(8):600-611.
70. Johansen MY, MacDonald C, Hansen KB, et al. Effect of an intensive lifestyle intervention on glycemic control in patients with type 2 diabetes: the U-TURN randomized clinical trial *JAMA - Journal of the American Medical Association.* 2017.
71. Evert AB, Boucher JL, Cypress M, et al. Nutrition therapy recommendations for the management of adults with diabetes. *Diabetes care.* 2013;36(11):3821-3842.
72. Sievenpiper JL, Dworatzek PD. Food and dietary pattern-based recommendations: an emerging approach to clinical practice guidelines for nutrition therapy in diabetes. *Can J Diabetes.* 2013;37(1):51-57.
73. Thomas D, Elliott EJ. Low glycaemic index, or low glycaemic load, diets for diabetes mellitus. *The Cochrane database of systematic reviews.* 2009(1):CD006296.
74. Thomas DE, Elliott EJ. The use of low-glycaemic index diets in diabetes control. *The British journal of nutrition.* 2010;104(6):797-802.
75. Jenkins DJ, Kendall CW, Augustin LS, et al. Effect of legumes as part of a low glycemic index diet on glycemic control and cardiovascular risk factors in type 2 diabetes mellitus: a randomized controlled trial. *Archives of internal medicine.* 2012;172(21):1653-1660.
76. Ajala O, English P, Pinkney J. Systematic review and meta-analysis of different dietary approaches to the management of type 2 diabetes. *The American journal of clinical nutrition.* 2013;97(3):505-516.
77. Ley SH, Hamdy O, Mohan V, Hu FB. Prevention and management of type 2 diabetes: dietary components and nutritional strategies. *Lancet.* 2014;383(9933):1999-2007.
78. Henry CJ. Basal metabolic rate studies in humans: measurement and development of new equations. *Public health nutrition.* 2005;8(7A):1133-1152.
79. Colberg SR, Sigal RJ, Fernhall B, et al. Exercise and type 2 diabetes: the American College of Sports Medicine and the American Diabetes Association: joint position statement. *Diabetes care.* 2010;33(12):e147-167.
80. Bueno AM, Pilgaard M, Hulme A, et al. Injury prevalence across sports: a descriptive analysis on a representative sample of the Danish population. *Inj Epidemiol.* 2018;5(1):6.

81. Snorgaard O, Drivsholm TB, Christiansen JS, et al. *Farmakologisk behandling af type 2-diabetes.* Dansk Endokrinologisk Selskab og Dansk Selskab for Almen Medicin;2014.
82. Færch K, Amadid H, Nielsen LB, et al. The PRE-D Trial: Protocol for a randomised controlled trial of the effect of dapagliflozin, metformin and physical activity on glucose variability, body composition and cardiovascular risk in pre-diabetes. *BMJ open.* 2016.
83. Service FJ, Molnar GD, Rosevear JW, Ackerman E, Gatewood LC, Taylor WF. Mean amplitude of glycemic excursions, a measure of diabetic instability. *Diabetes.* 1970;19(9):644-655.
84. Henriksen T, Hillestrom PR, Poulsen HE, Weimann A. Automated method for the direct analysis of 8-oxo-guanosine and 8-oxo-2'-deoxyguanosine in human urine using ultraperformance liquid chromatography and tandem mass spectrometry. *Free radical biology & medicine.* 2009;47(5):629-635.
85. Medhus AW, Sandstad O, Bredesen J, Husebye E. Delay of gastric emptying by duodenal intubation: sensitive measurement of gastric emptying by the paracetamol absorption test. *Aliment Pharmacol Ther.* 1999;13(5):609-620.
86. Hannukainen JC, Borra R, Linderborg K, et al. Liver and pancreatic fat content and metabolism in healthy monozygotic twins with discordant physical activity. *J Hepatol.* 2011;54(3):545-552.
87. Karstoft K, Winding K, Knudsen SH, et al. The effects of free-living interval-walking training on glycemic control, body composition, and physical fitness in type 2 diabetic patients: a randomized, controlled trial. *Diabetes care.* 2013;36(2):228-236.
88. McHorney CA, Ware JE, Jr., Raczek AE. The MOS 36-Item Short-Form Health Survey (SF-36): II. Psychometric and clinical tests of validity in measuring physical and mental health constructs. *Medical care.* 1993;31(3):247-263.
89. White IR, Horton NJ, Carpenter J, Pocock SJ. Strategy for intention to treat analysis in randomised trials with missing outcome data. *BMJ (Clinical research ed.).* 2011;342:d40.
90. USE ICOHOTRFROPFH. ICH HARMONISED TRIPARTITE GUIDELINE CLINICAL SAFETY DATA MANAGEMENT: DEFINITIONS AND STANDARDS FOR EXPEDITED REPORTING E2A 1994; http://www.ich.org/fileadmin/Public_Web_Site/ICH_Products/Guidelines/Efficacy/E2A/Step4/E2A_Guideline.pdf. Accessed 18/6, 2018.
91. Christensen RH, Wedell-Neergaard AS, Lehrskov LL, et al. The role of exercise combined with tocilizumab in visceral and epicardial adipose tissue and gastric emptying rate in abdominally obese participants: protocol for a randomised controlled trial. *Trials.* 2018;19(1):266.
92. Ryan DH, Espeland MA, Foster GD, et al. Look AHEAD (Action for Health in Diabetes): design and methods for a clinical trial of weight loss for the prevention of cardiovascular disease in type 2 diabetes. *Controlled clinical trials.* 2003;24(5):610-628.
93. Schulz KF, Altman DG, Moher D. CONSORT 2010 statement: updated guidelines for reporting parallel group randomised trials. *BMJ (Clinical research ed.).* 2010;340:c332.
94. (ICMJE) ICoMJE. Recommendations for the Conduct, Reporting, Editing, and Publication of Scholarly Work in Medical Journals. 2017. Accessed June 18, 2018.
