# Supplementary material for: Effects of different doses of exercise and diet-induced weight loss on beta-cell function in type 2 diabetes (DOSE-EX): a randomized clinical trial
Source: Nat Metab. 2023 May 1;5(5):880–95. doi: 10.1038/s42255-023-00799-7 (PMC10229430; doi:10.1038/s42255-023-00799-7)
Supplement: Supplementary file 2 — Reporting Summary [file 42255_2023_799_MOESM2_ESM.pdf]

nature portfolio

Corresponding author(s): Mathias Ried-Larsen

Last updated by author(s): 2023-27-03

Reporting Summary

Nature Portfolio wishes to improve the reproducibility of the work that we publish. This form provides structure for consistency and transparency in reporting. For further information on Nature Portfolio policies, see our Editorial Policies and the Editorial Policy Checklist.

Statistics

For all statistical analyses, confirm that the following items are present in the figure legend, table legend, main text, or Methods section.

n/a	Confirmed	
☐	☒	The exact sample size (*n*) for each experimental group/condition, given as a discrete number and unit of measurement
☐	☒	A statement on whether measurements were taken from distinct samples or whether the same sample was measured repeatedly
☐	☒	The statistical test(s) used AND whether they are one- or two-sided<br>*Only common tests should be described solely by name; describe more complex techniques in the Methods section.*
☐	☒	A description of all covariates tested
☐	☒	A description of any assumptions or corrections, such as tests of normality and adjustment for multiple comparisons
☐	☒	A full description of the statistical parameters including central tendency (e.g. means) or other basic estimates (e.g. regression coefficient) AND variation (e.g. standard deviation) or associated estimates of uncertainty (e.g. confidence intervals)
☐	☒	For null hypothesis testing, the test statistic (e.g. *F*, *t*, *r*) with confidence intervals, effect sizes, degrees of freedom and *P* value noted<br>*Give P values as exact values whenever suitable.*
☒	☐	For Bayesian analysis, information on the choice of priors and Markov chain Monte Carlo settings
☐	☒	For hierarchical and complex designs, identification of the appropriate level for tests and full reporting of outcomes
☒	☐	Estimates of effect sizes (e.g. Cohen's *d*, Pearson's *r*), indicating how they were calculated

Our web collection on statistics for biologists contains articles on many of the points above.

Software and code

Policy information about availability of computer code

Data collection	No software was used
Data analysis	Data was analyzed usign STATA/SE (StataCorp, College Station, Texas), version 17.1.

For manuscripts utilizing custom algorithms or software that are central to the research but not yet described in published literature, software must be made available to editors and reviewers. We strongly encourage code deposition in a community repository (e.g. GitHub). See the Nature Portfolio guidelines for submitting code & software for further information.

Data

Policy information about availability of data

All manuscripts must include a data availability statement. This statement should provide the following information, where applicable:

- Accession codes, unique identifiers, or web links for publicly available datasets
- A description of any restrictions on data availability
- For clinical datasets or third party data, please ensure that the statement adheres to our policy

Data are not available for download due to privacy/ethical restrictions under the EU GDPR. Specific requests for access to the trial and unique biological data as well as code may be sent to mathias.ried-larsen@regionh.dk. Based on the request access may be provided to a named individual in agreement with the rules and regulations of the Danish Data Protection Agency and the National Committee on Health Research Ethics.

Human research participants

Policy information about studies involving human research participants and Sex and Gender in Research.

Reporting on sex and gender	Randomization was stratified by sex to ensure a balanced design. Accordingly the analyses were adjusted for sex.
Population characteristics	Adult (age 58 years, 35% female) persons with type 2 diabetes (4 years from diagnosis)
Recruitment	Participants were recruited through the media, municipalities, and the Danish Health Data Authorities. As participants self-referred to the study, this may have caused a selection bias towards healthier persons. Consequently, the study results may not be generalized to the entire population living with type 2 diabetes
Ethics oversight	Scientific Ethical Committee of the Capital Region of Denmark (approval number H-18038298)

Note that full information on the approval of the study protocol must also be provided in the manuscript.

Field-specific reporting

Please select the one below that is the best fit for your research. If you are not sure, read the appropriate sections before making your selection.

☐ Life sciences ☒ Behavioural & social sciences ☐ Ecological, evolutionary & environmental sciences

For a reference copy of the document with all sections, see nature.com/documents/nr-reporting-summary-flat.pdf

Behavioural & social sciences study design

All studies must disclose on these points even when the disclosure is negative.

Study description	The study was a 16-week parallel-group, 4-arm, assessor-blinded, randomized clinical trial using quantitative data
Research sample	Danish Men and women aged 18–80 years, diagnosed with T2D within <7 years, no current treatment with insulin, BMI >27 kg/m2 and <40 kg/m2. The sample was recruited due to increased risk of morbidities where diet and exercise may alleviate these. Prior studies have indicated that to reestablish beta-cell function, the diabetes should be of a relative short duration and remaining beta-cell function should be present. Thus short diabetes duration was considered and we included persons without the need of exogenous insulin. The sample is likely not representative of the entire population living with type 2 diabetes
Sampling strategy	This was a convenience sample and the final sample size was determined by the N participants needed to reach a statistical power of >80%. Sample size was calculated based on a previous exercise study in persons with type 2 diabetes (DOI: 10.1007/s00125-014-3334-5). For a contrast in a one-way ANOVA with four means (1.5, 1.0, 0.5, 0.0) and contrast coefficients (1, 0, 0, -1) using a two-sided significance level of 0.05, assuming an error standard deviation of 1.5 and a balanced design, a total sample size of 80 participants in the per-protocol population (approximately 20 participants in each group) would yield statistical power of 87.7%.
Data collection	The web-based Clinical Trial Management System EasyTrial was used for data entry and management (EasyTrial ApS). EasyTrial has been approved by the Danish Data Protection Board. Electronic case report forms (eCRF) and questionnaires will be generated by the sponsor in EasyTrial. Fields have been programmed with acceptable ranges for data entry. All paper material (CRF, blood screen results, questionnaires and dietary records) was collected and stored in a locked cabinet at CFAS, Rigshospitalet Denmark. All information from the paper material was entered twice by in non-consecutive order into the electronic back-end system. In case of discrepancies between the entries, the original paper record was consulted. Upon completion of the study, all paper material have been scanned and stored on the secured hospital server in an electronic form. Data management will be performed using appropriate statistical software Excel and STATA.<br>To enable pseudonymised data, all participants was ascribed a unique participant identification (ID) number. The identification key (ID number to personal information) was stored on a password-protected computer, separate from the unique ID number and the database. Printed data was kept in a separate locked area with limited access. All patient-related information obtained during the study was handled in accordance with the Danish law for protection of personal data ("lov om behandling af personoplysninger") and the Danish health law ("sundhedsloven"). The blood samples were registered from the hospital blood sample portal (Labka) and para-clinical observations will be obtained through "Sundhedsportalen". The study was reported to the Danish Data Protection Agency ("Region H's paraplyanmeldelse") VD-2018-516/ I-suite no. 6768.<br>The researchers were blinded for allocation.<br>No other persons were present during data collection other than the participants and the researchers.
Timing	February 2019 and October 2021
Data exclusions	Pre-defined exclusion criteria were defined. No participants were excluded after randomization.
Non-participation	Five participants were lost-to-follow-up; one due to malignancy, one was dissatisfied with group allocation, one refrained from study testing due to COVID19, and two due to musculoskeletal injuries.

Randomization	The participants were randomly allocated to the 4 intervention arms upon successful completion of the baseline measurements. An independent statistician prepared a computer-generated randomization schedule in a ratio of 1:1:1:1, stratified by sex. To ensure concealment, the (permuted) block sizes were not disclosed. The schedule was forwarded to a secretary not involved in any study procedures and stored on a password-protected computer. Sequentially numbered opaque, sealed envelopes were prepared and stored in a locked cabinet prior to commencing the recruitment. The envelopes were lined with aluminum foil to render the envelope impermeable to intense light. Following the conclusion of the hyperglycemic clamp, the appropriate envelope was opened by a study nurse and the participant was informed about the allocation stated on the card inside the envelope. The participant received the allocation in a closed room. As such, the participants were blinded for treatment allocation until after the completion of the hyperglycemic clamp.

Reporting for specific materials, systems and methods

We require information from authors about some types of materials, experimental systems and methods used in many studies. Here, indicate whether each material, system or method listed is relevant to your study. If you are not sure if a list item applies to your research, read the appropriate section before selecting a response.

Materials & experimental systems

n/a	Involved in the study	
☒	☐	Antibodies
☒	☐	Eukaryotic cell lines
☒	☐	Palaeontology and archaeology
☒	☐	Animals and other organisms
☐	☒	Clinical data
☒	☐	Dual use research of concern

Methods

n/a	Involved in the study	
☒	☐	ChIP-seq
☒	☐	Flow cytometry
☒	☐	MRI-based neuroimaging

Clinical data

Policy information about clinical studies

All manuscripts should comply with the ICMJE guidelines for publication of clinical research and a completed CONSORT checklist must be included with all submissions.

Clinical trial registration	NCT03769883
Study protocol	Submitted with the manuscript
Data collection	Conducted between February 2019 and October 2021 at the Centre for Physical Activity Research (CFAS), Rigshospitalet, Copenhagen, Denmark
Outcomes	The primary outcome was assessed using a hyperglycemic clamp method. The secondary outcomes where assessed using a hyperglycemic clamp method and a mixed meal tolerance test. Blood samples were drawn as outlined in the protocol and manuscript by blinded research staff. The samples were sent do a central lab at Rigshospitalet, where they were analyzed by staff unaware of allocation.
